# Cellular mechanism of action of forsythiaside for the treatment of diabetic kidney disease

**DOI:** 10.3389/fphar.2022.1096536

**Published:** 2023-01-13

**Authors:** Chunmei Xu, Huikai Miao, Xiaoxuan Chen, Haiqing Zhang

**Affiliations:** ^1^ Department of Endocrinology, Shandong Provincial Hospital Affiliated to Shandong First Medical University, Jinan, China; ^2^ Shandong Key Laboratory of Endocrinology and Lipid Metabolism, Shandong Provincial Hospital, Jinan, China; ^3^ Department of Endocrinology, Shandong Provincial Hospital, Shandong University, Jinan, China; ^4^ Shandong Provincial Institute of Dermatology and Venereology, Shandong University, Jinan, China

**Keywords:** forsythiaside, diabetic kidney disease, network analysis, oxidative stress, podocytopathy, traditional Chinese medicine

## Abstract

**Background:** Diabetic kidney disease (DKD) becomes the leading cause of death for end-stage renal disease, whereas the potential mechanism is unclear and effective therapy is still rare. Our study was designed to investigate the cellular mechanism of Forsythiaside against DKD.

**Materials and Methods:** The targets of Forsythiaside and the DKD-related targets were obtained from databases. The overlapping targets in these two sets were regarded as potential targets for alleviation of DKD by Forsythiaside. The targets of diabetic podocytopathy and tubulopathy were also detected to clarify the mechanism of Forsythiaside ameliorating DKD from the cellular level.

**Results:** Our results explored that PRKCA and RHOA were regarded as key therapeutic targets of Forsythiaside with excellent binding affinity for treating DKD podocytopathy. Enrichment analysis suggested the underlying mechanism was mainly focused on the oxidative stress and mTOR signaling pathway. The alleviated effects of Forsythiaside on the reactive oxidative species accumulation and PRKCA and RHOA proteins upregulation in podocytes were also confirmed.

**Conclusion:** The present study elucidates that Forsythiaside exerts potential treatment against DKD which may act directly RHOA and PRKCA target by suppressing the oxidative stress pathway in podocytes. And Forsythiaside could be regarded as one of the candidate drugs dealing with DKD in future experimental or clinical researches.

## 1 Introduction

As the population who suffers from diabetes surges rapidly, the prevalence rate of diabetic kidney disease (DKD) significantly increases and occurs in approximately 40% of patients with type 2 diabetes mellitus (de Boer et al., 2011). DKD as one of the most deleterious complications of diabetes, has become the leading cause of end-stage renal disease (ESRD), which considerably does harm for the life quality of patients and lays serious economic burden in the society and families (Doshi and Friedman, 2017). A sufficient understanding the underlying mechanisms of renal injury under the condition of high glucose facilitates the advancement of prevention and treatment of DKD.

A variety of cells were involved in the pathogenesis of DKD and the clinical effects and mechanism of DKD occurrence and development have been preliminarily elucidated. Studies showed that glomerular changes were undoubtedly major characteristics of DKD (Gilbert, 2017). Traditional study revealed that glomerular podocyte injury played an important role in the process of DKD development (Chen et al., 2020). Furthermore, the role of tubular abnormality in promoting DKD progression has been gradually illustrated. High glucose can do harm to the kidney through various pathways, including renal glomerular (Ren et al., 2017), tubular (Zhao et al., 2021), vascular (Zhan et al., 2021) and renal interstitial injuries (Cheng et al., 2020). Intensive glucose control in DKD could slow down but not significantly prevent the progression of DKD (MacIsaac et al., 2017). Other conventional therapy which was restricted to renin-angiotensin aldosterone system inhibitor also showed limited benefit for delaying the disease progression (Coleman et al., 2020). Thus, conventional western therapies fail to provide favorable utility for DKD.

Traditional Chinese Medicine (TCM) shows remarkable therapeutic effect on chronic diseases, and has been widely supplemented to intervene DKD in China and other Asian countries (Lovisa et al., 2016; Chen D. Q. et al., 2018; Chen H. et al., 2018; Wang et al., 2018). Chinese botanical drug medicine as a treasure trove of TCM is rich in lots of natural compounds, which are effective in treating diseases for their achievement of the multi-target, and multi-mechanism system regulation. However, there is less research of natural compounds from the Chinese botanical drug medicine against renal injury in DKD (Zhang et al., 2019).

According to the theory of TCM, Forsythiaside, as a main constituent of *Forsythia suspensa* (Thunb.) Vahl, has long been thought to have benefits involving clearing heat and detoxifying, dispelling carbuncle and is commonly used in treating infections and pyrexia (Guo et al., 2007; Lu et al., 2010). Previous pharmacologic studies indicated that the Forsythiaside has multiple biological functions such as anti-inflammatory (Wang et al., 2016), antioxidant (Lu et al., 2010), and hepatoprotection (Zhang et al., 2018). Nowadays, Forsythiaside has a wide range of applications in various diseases, for instance, inflammation (Zeng et al., 2017), virus infection (Law et al., 2017), neurodegeneration (Chen L. et al., 2019), oxidative stress (Huang et al., 2015), and liver injury (Gong et al., 2021). Recently, study revealed that Forsythiaside A showed protective effects against adriamycin-induced nephropathy in rat by reducing proteinuria, serum creatinine and urea nitrogen levels and apoptotic cells (Lu et al., 2020). Moreover, Forsythiaside alleviated adriamycin-triggered renal injury and enhanced superoxide dismutase activities, inhibited malondialdehyde and lactate dehydrogenase productions (Lu et al., 2020). However, the influence and underlying mechanism of Forsythiaside on the alleviation of DKD is yet to be completely elucidated. Therefore, the nephro-protective activities of Forsythiaside in DKD were further investigated in this study.

With the continuous progress and rapid development of various high-throughput technologies, a large number of scientific researches have surged, which further contribute to an enormous and novel bioinformation network. Network analysis as an emerging bioinformation approach for drug discovery and development shows the marked advantages of convenience of acquiring information, low cost, and a flood of valuable resource. Network analysis emerges as the new mode of large-scale data processing develops and investigates the relationship between compounds and medicines, diseases and targets by calculating the synergistic probability of multi-compound, multi-target and multi-pathway (Hopkins, 2007). And network analysis is useful to explain the mechanism of diseases from the systemic view and guides the discovery of new medicines for elucidating the potential interaction of the body and medicines. It has gradually become a new trend to apply the methods of network analysis to clarify the possibility and mechanism of Chinese botanical drug medicine and compound in treating disease.

Nowadays, there have been major advances in the understanding and treatment of DKD, whereas few researches about natural compounds of Chinese botanical drug medicine relieving the development of DKD from evidence-based single and specific cellular facet were available. Therefore, the objective of this study was to reveal the effects of Forsythiaside on the DKD alleviation and its mechanism in the treatment of DKD from the specific cellular level. The target proteins of Forsythiaside and DKD, diabetic podocytopathy and diabetic tubulopathy were retrieved and established respectively by public database. Then the enrichment analyses were further carried out. The molecular docking simulation based on “lock-key principle” and energy matching was performed to calculate the binding affinity between macromolecule and ligand for virtually prediction of drug targets. Finally, the potential mechanism of Forsythiaside alleviating renal damage was preliminarily confirmed and Forsythiaside-targets-pathways network was constructed and visualized by Cytoscape 3.8.2 software (Pang et al., 2018). Our research could provide preliminarily theoretical basis for the curative effects of natural compounds of TCM on the treatments of DKD and broaden the horizon for clinical integration of traditional Chinese and Western medicine on DKD treatment in future.

## 2 Materials and methods

### 2.1 Analysis of predicted targets of forsythiaside

The drug-like properties of Forsythiaside are summarized in Table 1. The potential intervention targets of Forsythiaside were extracted from the ChEMBL database (Gaulton et al., 2017) (https://www.ebi.ac.uk/chembl/g/), the PubChem database (Kim et al., 2019) (http://pubchem.ncbi.nlm.nih.gov), the Swiss Target Prediction database (Gfeller et al., 2014) (http://www.swisstargetprediction.ch/), the Binding database (Chen et al., 2001) (https://www.bindingdb.org/bind/index.jsp), and the PharmMapper database (Wang et al., 2017) (http://lilab-ecust.cn/pharmmapper/index.html). All potential targets were summarized and their names standardized using the universal Protein Resource site (RRID: SCR_002380) (http://www.uniprot.org/).

**TABLE 1:** Pharmacological and molecular properties data of Forsythiaside.

MW	AlogP	Hdon	Hacc	OB (%)	Caco-2	BBB	DL	FASA	TPSA	RBN
624.65	.38	9	15	3.05	−1.92	−2.95	.61	.35	245.29	11

Abbreviation: MW, molecular weight; AlogP, partition coefficient; Hdon, hydrogen bond donors; Hacc, hydrogen bond acceptors; OB, oral bioavailability; Caco-2: Caco-2 cell permeability; BBB: blood brain barrier; DL, drug-likeness; FASA, fractional negative accessible surface area; TPSA, topological polar surface area; RBN, number of rotatable bonds.

### 2.2 Prediction of diabetic kidney disease-related target proteins

The target proteins involved in the pathogenesis of DKD were predicted and acquired from a variety of databases. The terms “Diabetic kidney disease” and “Diabetic nephropathy” were used as keywords across multiple databases. We used Therapeutic Target (Wang et al., 2020) (http://bidd.nus.edu.sg/group/cjttd/), GeneCards (Rebhan et al., 1998) (https://www.genecards.org/), DisGeNET (RRID: SCR_006178) (https://www.disgenet.org/) (Piñero et al., 2020), DrugBank (Wishart et al., 2008) (https://go.drugbank.com/) and Online Mendelian Inheritance in Man (OMIM, RRID: SCR_006437) (https://omim.org/) (Boyadjiev and Jabs, 2000) databases to screen and identify potential targets of DKD.

### 2.3 Identification of overlapping targets of forsythiaside and diabetic kidney disease

#### 2.3.1 Acquiring candidate target molecules by target mapping

To clarify the candidate target molecules *via* which Forsythiaside could intervene in DKD, the predicted targets of Forsythiaside were mapped onto the DKD-related proteins and overlapping targets were extracted as key genes. Overlapping targets were visualized using a Venn diagram.

#### 2.3.2 Construction of a protein-protein interaction network

Once mapped, overlapping target molecules between Forsythiaside and DKD were inserted into the STRING website (Szklarczyk et al., 2017) (https://string-db.org/), a useful tool for the discovery of functional protein interactions and further generation of a PPI network.

#### 2.3.3 Network construction based on overlapping targets of forsythiaside and diabetic kidney disease

Cytoscape software (CluePedia Cytoscape plugin, RRID: SCR_015784) (https://cytoscape.org/), an open source software platform for visualizing intricate networks and integrating networks with attribute data (Shannon et al., 2003), was applied to establish the networks of Forsythiaside and DKD’s respective targets, the PPI network of overlapping targets between Forsythiaside and DKD, and overlapping target-pathway networks. The properties of each PPI network were calculated and analyzed using the Cytoscape plug-in Network Analyzer. Higher parameter values indicate that a node is more significant.

#### 2.3.4 KEGG enrichment analysis based on overlapping targets between forsythiaside and diabetic kidney disease

To further clarify the potential role of Forsythiaside in alleviating DKD, overlapping targets were inserted into the Database for Annotation, Visualization and Integrated Discovery site (Huang, et al., 2009) (version 6.8; https://david.ncifcrf.gov/) to carry out KEGG enrichment analysis. Bubble diagrams of KEGG enrichment analysis were drawn using R Project for Statistical Computing (RRID: SCR_001905) (https://www.r-project.org/).

### 2.4 Analysis of diabetic podocytopathy and tubulopathy-related target molecules

In order to specify the cellular mechanism of Forsythiaside in the treatment of DKD, target molecules which were related to diabetic podocytopathy and diabetic tubulopathy were predicted and acquired from multiple databases. The terms “Diabetic podocytopathy” or “Diabetic tubulopathy” were inserted into the GeneCards and OMIM databases as keywords.

### 2.5 Establishment of a protein-protein interaction network, and network analysis based on overlapping targets of forsythiaside and diabetic podocytopathy or tubulopathy

Overlapping targets between Forsythiaside and diabetic podocytopathy were acquired by drawing a Venn diagram, and candidate diabetic tubulopathy target genes acted on by Forsythiaside were obtained by using similar methods.

Upon target mapping, overlapping target molecules between Forsythiaside and diabetic podocytopathy or tubulopathy were used to generate a PPI network using the STRING database (RRID: SCR_005223) (https://cn.string-db.org/) following the methods described in Section 2.3.2.

Furthermore, PPI networks were developed for the following: diabetic podocytopathy and targets; diabetic tubulopathy and targets; overlapping targets between Forsythiaside and diabetic podocytopathy; overlapping targets between Forsythiaside and diabetic tubulopathy; and overlapping target-pathway network were constructed using Cytoscape software (RRID: SCR_003032) (https://cytoscape.org/). The values of degree were calculated using the Cytoscape plug-in Network Analyzer, and the nodes were ranked by these values.

### 2.6 GO pathway and KEGG enrichment based on overlapping proteins between forsythiaside and diabetic podocytopathy or tubulopathy

In order to elucidate the pathways through which Forsythiaside may intervene in diabetic podocytopathy or tubulopathy, the overlapping targets of Forsythiaside and diabetic podocytopathy or tubulopathy were inserted into the Database for Annotation, Visualization and Integrated Discovery (version 6.8; https://david.ncifcrf.gov/) and the Metascape database (RRID: SCR_016620) (http://metascape.org/) (Zhou et al., 2019). The top 10 pathways were displayed.

Upon three enrichment analyses, the key pathways through which Forsythiaside alleviates DKD, diabetic podocytopathy, and tubulopathy, respectively were identified. The same pathways were extracted as key ones that Forsythiaside might alter to treat DKD, by ameliorating targets in diabetic podocytopathy or tubulopathy. The overlapping Forsythiaside and diabetic podocytopathy or tubulopathy target proteins that were identified as critical proteins through which Forsythiaside may intervene in order to treat DKD.

### 2.7 Molecular docking simulation

#### 2.7.1 Acquirement and preparation of protein receptors

The crystal structures of critical proteins were acquired from RCSB Protein Data Bank database (Goodsell et al., 2020) (http://www.pdb.org/), and PDB codes of the protein receptors were summarized in Table 2. PyMOL software (version 2.5.0) was used to edit the protein complexes by removing the original ligand and water molecules from them, and the modified protein was saved in pdb format (Lu, 2020). Autodock software (version 4.2.6) (Morris et al., 2009), a suite of automated docking tools, and Autodock Tools (version 1.5.6) (http://mgltools.scripps.edu/documentation/links/autodock) were used to prepare the appropriate protein receptor by adding hydrogen and computing charges. The protein macromolecule was saved in pdbqt format.

**TABLE 2:** Results of 9 hub genes and Forsythiaside molecular docking.

Number	Hub gene	PDB code	Docking affinity (kcal/mol)
1	PPARG	6fzp	.91
2	INSR	2hr7	2.64
3	PRKCA	4ra4	−.15
4	SOD2	2adq	.78
5	GCK	1v4s	1.38
6	ACE	1o86	1.0
7	RHOA	6kx2	−1.14
8	HDAC1	7sme	−.01
9	CCR5	4mbs	2.3

#### 2.7.2 Ligand preparation

Before docking, the structure of Forsythiaside was acquired using PubChem and the Traditional Chinese Medicine System Pharmacology Database (http://lsp.nwu.edu.cn/tcmsp.php) (Ru et al., 2014), which was saved in Mol2 format and further analyzed. The conformation of Forsythiaside (Pubchem CID: 5281773) was displayed in Supplementary Figure S1. Then, Autodock software (version 4.2.6) was applied to transfer the structure in Mol2 format to pdbqt format to allow energy minimization for the docking.

#### 2.7.3 Molecular docking

Upon the preparation of ligand and receptor, Autodock software (version 4.2.6) was used to evaluate and confirm the binding affinity between ligand and receptor and further construct the binding model. Binding models were visualized using PyMOL software (RRID: SCR_000305) (https://pymol.org/2/), and Discovery Studio Visualizer software (version 21.1.0.20298) was applied to transform the 3D structure of the complex to 2D.

### 2.8 Reagent, cell culture and treatment

Forsythiaside A powder (FA, the main component of Forsythiaside, purity: 99.43%) was purchased from MedChemExpress (cat. HY-N0028; Monmouth Junction, NJ, United States), and dissolved in DMSO at various concentrations (2.5, 5, and 10 μg/mL). Conditionally immortalized human podocytes were kindly supplied by Dr. Yi Fan (Department of Pharmacology, Shandong University School of Medicine, Jinan, China) and cultured in RPMI-1640 medium (cat no. 11875093; Gibco, Grand Island, NY, United States) supplemented with 10% fetal bovine serum (cat no. 10099141; Gibco), 100 units/mL penicillin and 100 mg/mL streptomycin (1% P/S concentration, cat no. 10378016; Gibco) until cells became confluent.

Podocytes were incubated in various media containing 5.5 mM D-glucose (NG), 5.5 mM D-glucose +34.5 mM D-mannitol (MG) (Chen Y. et al., 2019), 40 mM D-glucose (HG), or 40 mM D-glucose + Forsythiaside at various concentrations (HG + FA 2.5, 5, or 10 μg/mL). D-glucose and D-mannitol were purchased from Sigma-Aldrich Canada Co. (cat nos. 50-99-7 and #69-65-8; Oakville, ON, Canada). After treatment, podocytes were harvested for protein extraction.

### 2.9 Cell viability assessment by Cell Counting Kit-8 assay

Human podocytes were seeded at a density 3×10^4^ cells/cm^2^ in RPMI 1640 medium on 96-well plates. After cell adherence, RPMI-1640 medium was replaced with other media, including NG, MG, HG, or HG + FA at concentrations of 2.5, 5, or 10 μg/mL. Cells were subsequently incubated for 48 h. Cell viability was detected using the CCK-8 assay kit (cat no. CK04-11; Dojindo Molecular Technologies, Inc., Kumamoto, Japan) according to the manufacturer’s instructions. The absorbance of each sample was measured at a wavelength of 450 nm.

### 2.10 Western blotting

Proteins were isolated from cultured podocytes after various treatments. Protein levels were detected using a BCA Protein Assay Kit (cat no. P0012; Beyotime, Shanghai, China). Proteins were subsequently separated by SDS-PAGE electrophoresis. Antibodies against protein kinase C alpha (PRKCA) (cat no. 21991-1-AP; Proteintech Group Inc., Wuhan, China, 1:1,000) and RHOA (cat no. 10749-1-AP; Proteintech, 1:1,000) were used for western blotting. An antibody against *β*-actin (cat no. 60008-1-Ig; Proteintech, 1:5,000) was used as a reference.

### 2.11 Reactive oxidative species measurement

ROS were measured in podocytes using a ROS detection kit (cat no. S0033S; Beyotime, Shanghai, China) according to the manufacturer’s instructions. Briefly, podocytes were seeded on sterile glass coverslips in a 6-well plate and incubated with different media for 48 h. Thereafter, slides were incubated with a 10 μM DCFH-DA working solution for 30 min at 37°C. After washing three times with warm PBS, ROS intensity was detected under a fluorescent microscope (Olympus FSX100).

### 2.12 Statistical analysis

Technical and biological replicates were performed three times. Data were processed using SPSS software (RRID: SCR_002865) (https://www.ibm.com/products/spss-statistics) and presented as means ± standard error of the mean. Student’s t-test was used to evaluate the significance of difference between two groups. One-way ANOVA and two-way ANOVA tests were used for multiple statistic comparisons. *p* < .05 was considered as statistically significant.

## 3 Results

### 3.1 Forsythiaside-target network and diabetic kidney disease-target network construction

**FIGURE 1:**
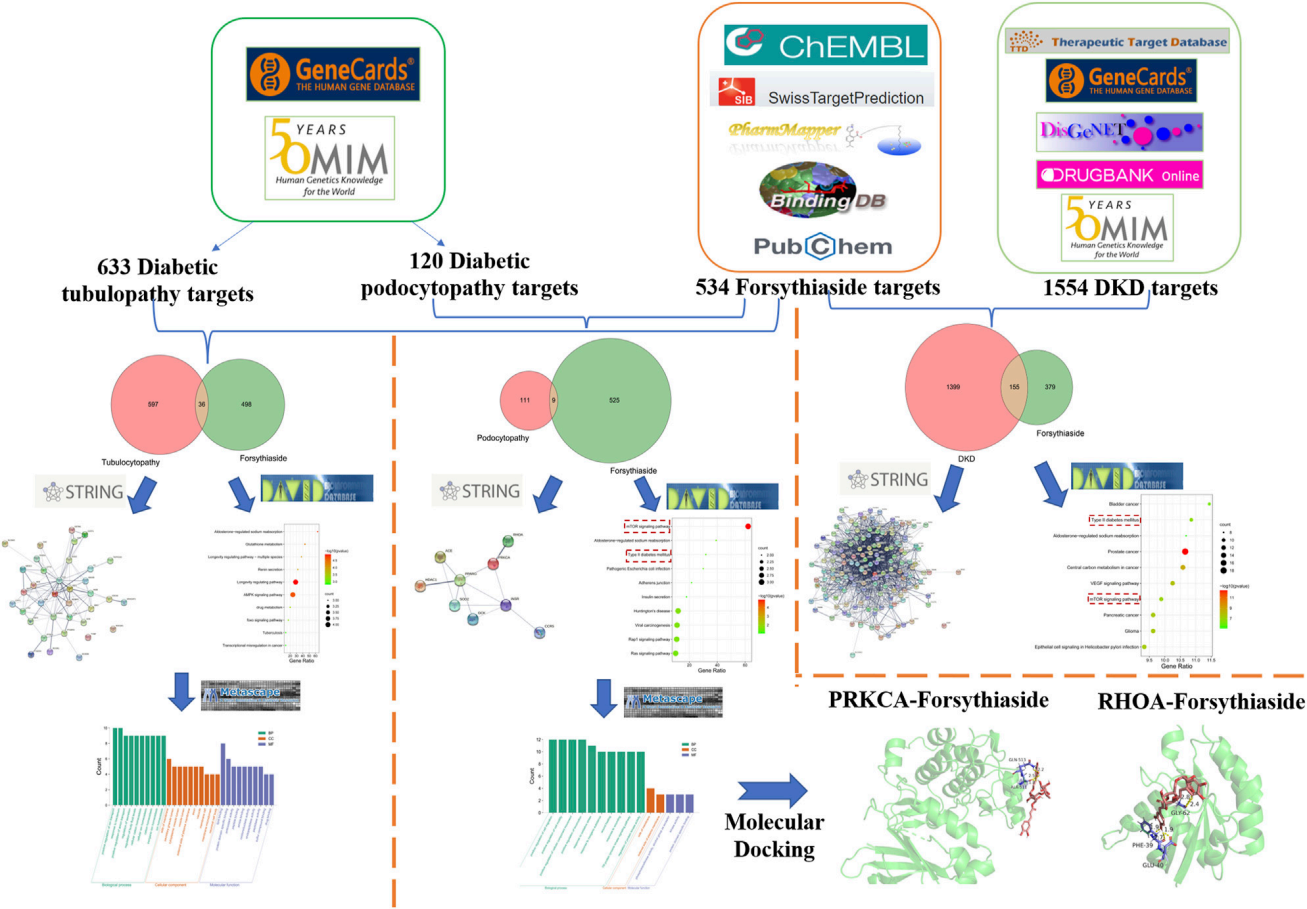
Flowchart of exploring the mechanisms of Forsythiaside in DKD from the specific cellular level.

**FIGURE 2:**
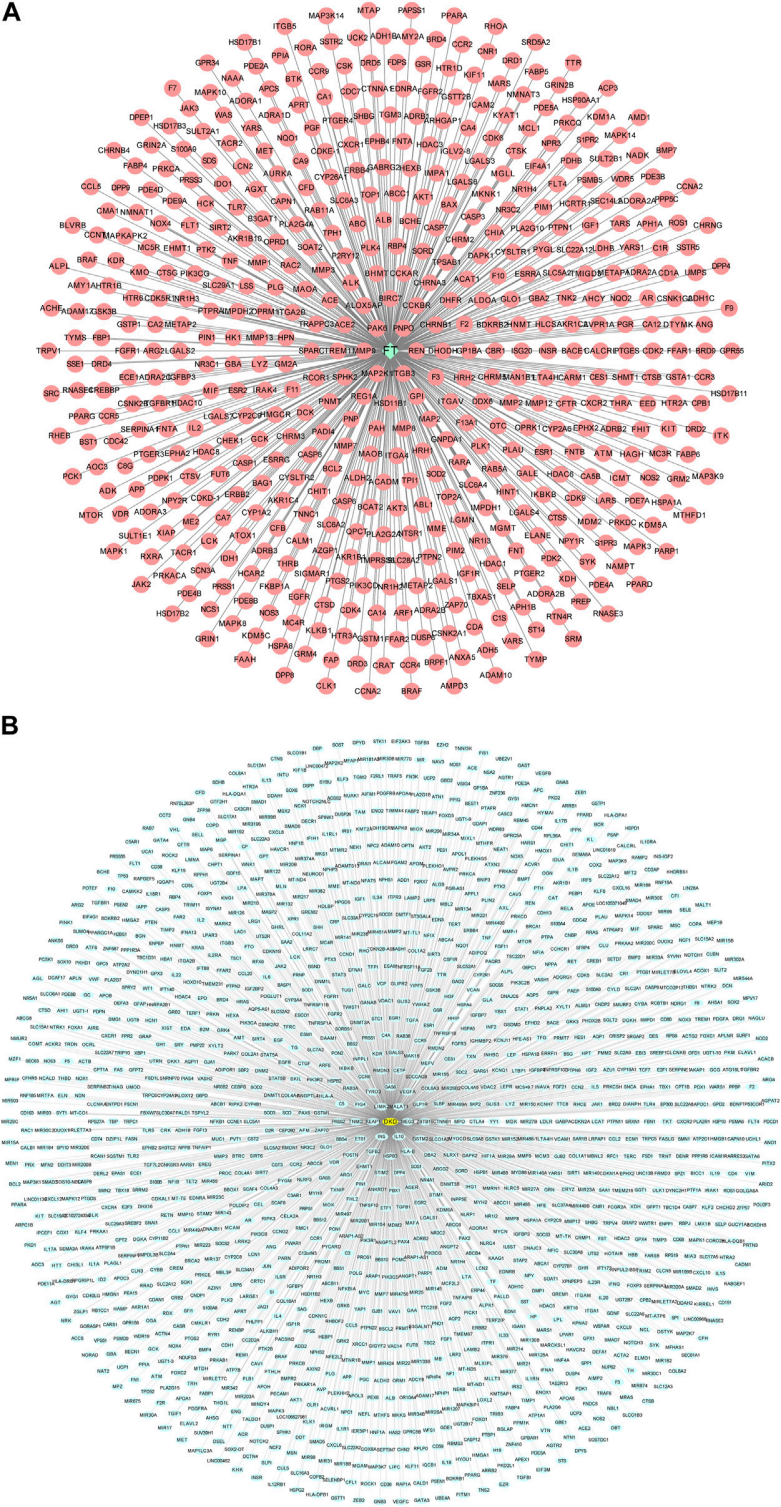
Compound-target network and disease-target network. **(A)** Forsythiaside-target network. Green diamond node represents Forsythiaside and red circular nodes represent corresponding targets. **(B)** DKD-target network. Yellow circular node represents DKD and green circular nodes represent corresponding targets. FT: Forsythiaside; DKD: diabetic kidney disease.

A flowchart summarizing the results of the network analysis study is shown in Figure 1. We used the ChEMBL, PubChem, Swiss Target Prediction, Binding, and PharmMapper databases to screen and acquire the putative targets of Forsythiaside. 534 predicted targets were identified as the potential target proteins of Forsythiaside (Figure 2A). The target proteins were standardized using universal Protein Resource site, and detailed information is given in Supplementary Table S1.

By using the Therapeutic Target, GeneCards, DisGeNET, DrugBank, and OMIM databases, a total of 1,554 target proteins were identified as potential targets of DKD (Figure 2B). Information on target proteins is given in Supplementary Table S2.

### 3.2 Protein-protein interaction network of Forsythiaside-diabetic kidney disease targets

**FIGURE 3:**
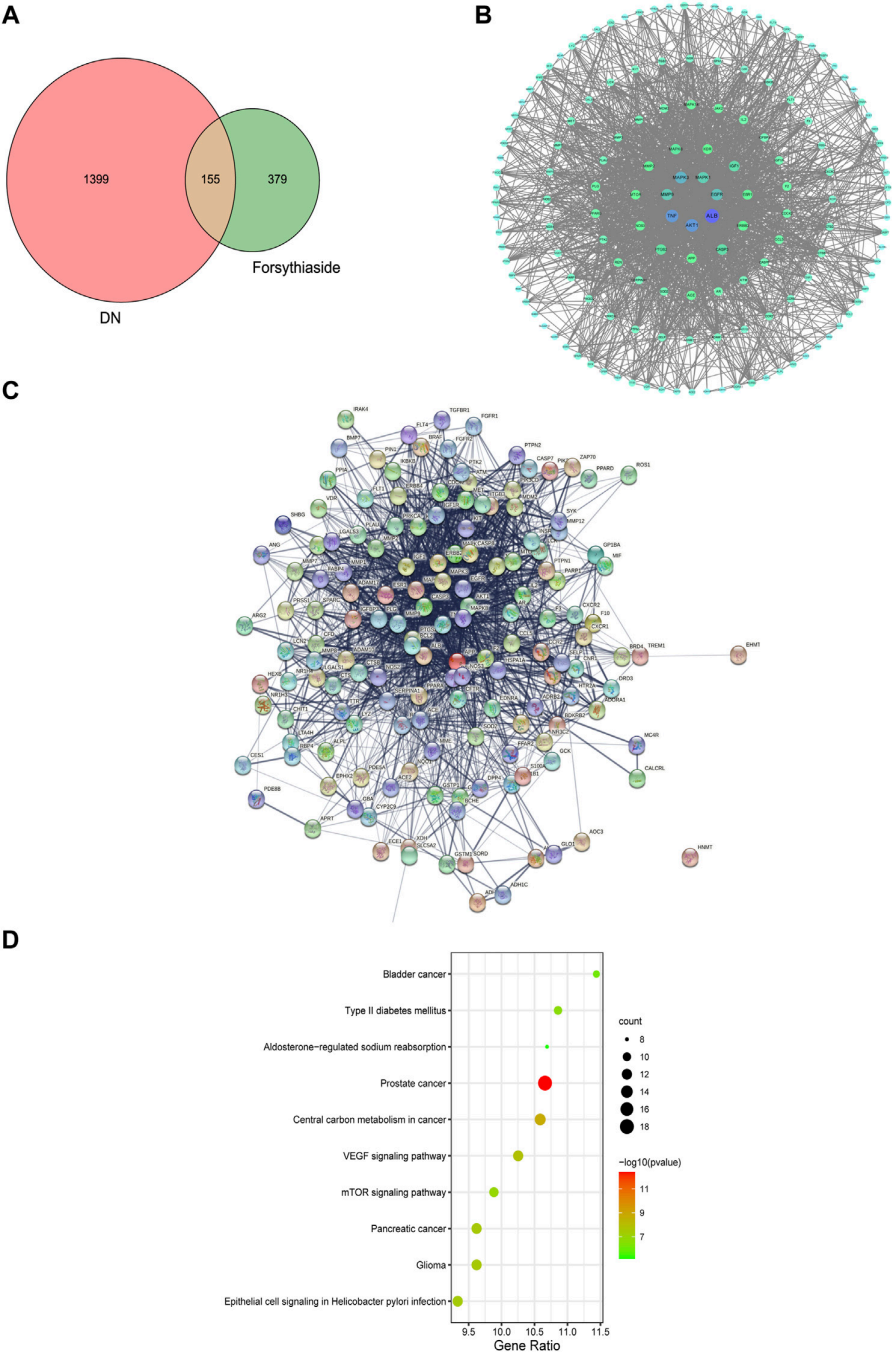
Venn diagram, PPI network and KEGG enrichment of Forsythiaside-DKD targets. **(A)** Venn diagram of overlapping targets of Forsythiaside and DKD. **(B)** PPI network of Forsythiaside-DKD targets. A total of 155 overlapping targets were presented as circle and ordered by the degree level. The hub targets were presented in the innermost center circle, and the core targets were AKT1, ALB, TNF, MAPK3, MAPK1, MMP9, and EGFR. **(C)** The interactive relationship was analyzed and displayed as PPI network by STRING website. **(D)** KEGG enrichment analysis of potential targets that Forsythiaside acted for alleviating DKD. DKD: diabetic kidney disease.

To acquire the targets through which Forsythiaside acted on DKD, the 534 putative targets of Forsythiaside were mapped to the 1554 DKD-related targets to obtain overlapping targets of Forsythiaside and DKD. The overlapping targets were visualized using a Venn diagram shown in Figure 3A. Consequently, 155 targets were identified as candidate targets through which Forsythiaside might alleviate DKD. Cytoscape software (version 3.8.2) was applied to construct the PPI network, which could evaluate the action of the target proteins in disease, and reveal their potential interactions. The PPI network of overlapping proteins between Forsythiaside and DKD was established, and 155 targets were sorted in descending order by degree. As shown in Figure 3B, targets were presented as circles and ordered by the degree. Hub targets were presented in the innermost center circle, and the core targets were AKT1, ALB, TNF, MAPK3, MAPK1, MMP9, and EGFR. The interactive relationship was analyzed using the STRING tool; results are displayed in Figure 3C.

### 3.3 KEGG pathway enrichment analysis

To systematically elucidate the mechanism of Forsythiaside treatment, KEGG enrichment analysis of 155 overlapping genes between Forsythiaside and DKD was performed. The top 10 pathways were selected and drawn according to counts of hub genes and *p* values (Figure 3D). Several pathways were selected as the key action mechanism underlying DKD treatment with Forsythiaside, including Type II diabetes mellitus and mTOR signaling pathways.

### 3.4 Diabetic podocytopathy and tubulopathy target network

**FIGURE 4:**
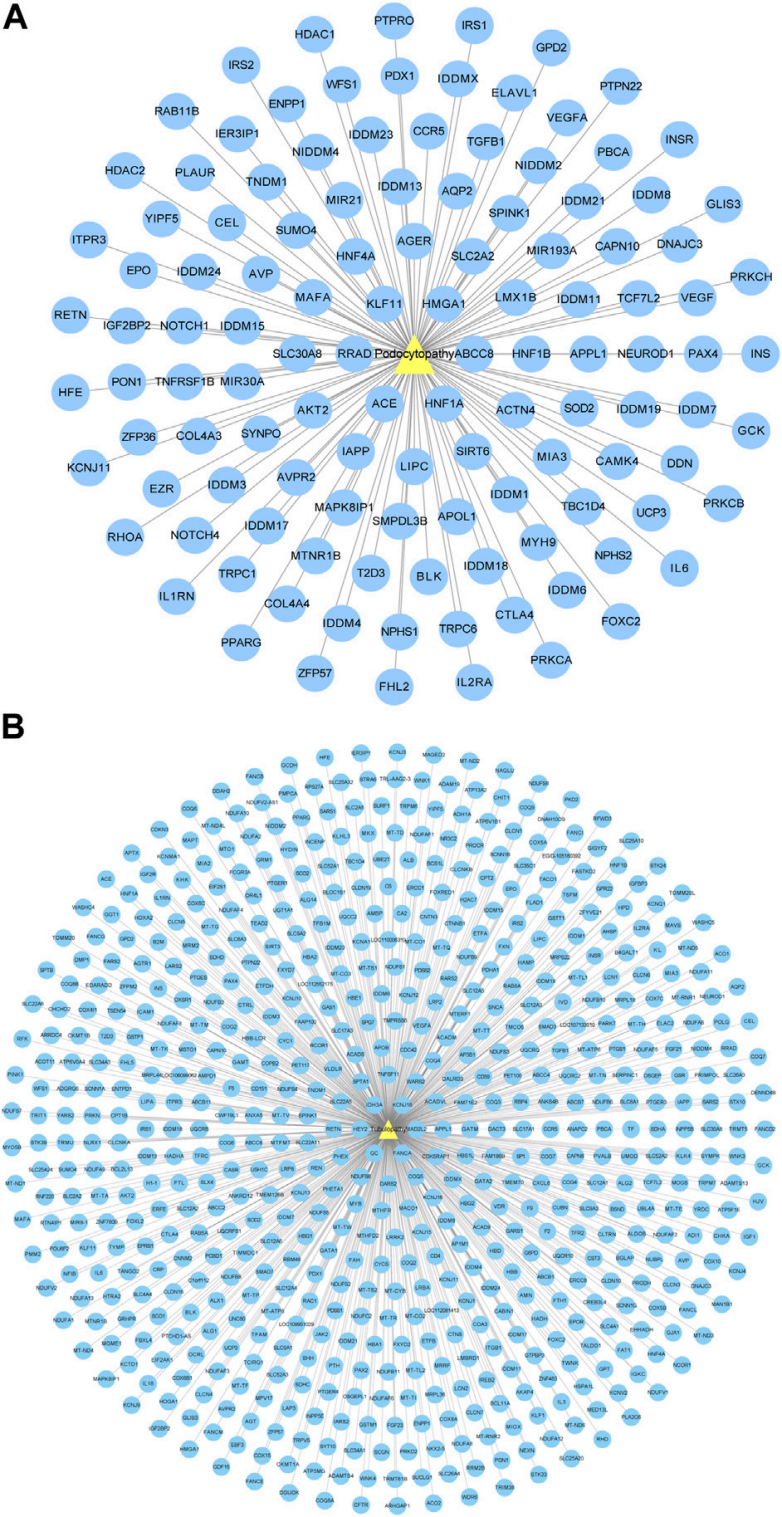
Disease-target network. **(A)** Diabetic podocytopathy-target network. Yellow triangle node represents diabetic podocytopathy and blue circular nodes represent corresponding targets. A total of 120 proteins were documented as the potential targets of diabetic podocytopathy. **(B)** Diabetic tubulopathy-target network. Yellow triangle node represents diabetic tubulopathy and blue circular nodes represent corresponding targets. Total 633 predicted target proteins of diabetic tubulopathy were identified.

The GeneCards and OMIM databases were used to acquire the targets of diabetic podocytopathy. A total of 120 proteins were documented as potential targets of diabetic podocytopathy (Figure 4A). Detailed information on these proteins was recorded in Supplementary Table S3. The same analysis was conducted, and 633 predicted target proteins of diabetic tubulopathy were identified from the databases (Figure 4B). Relative information of proteins is presented in Supplementary Table S4.

### 3.5 Protein-protein interaction networks of forsythiaside-diabetic podocytopathy and tubulopathy targets

**FIGURE 5:**
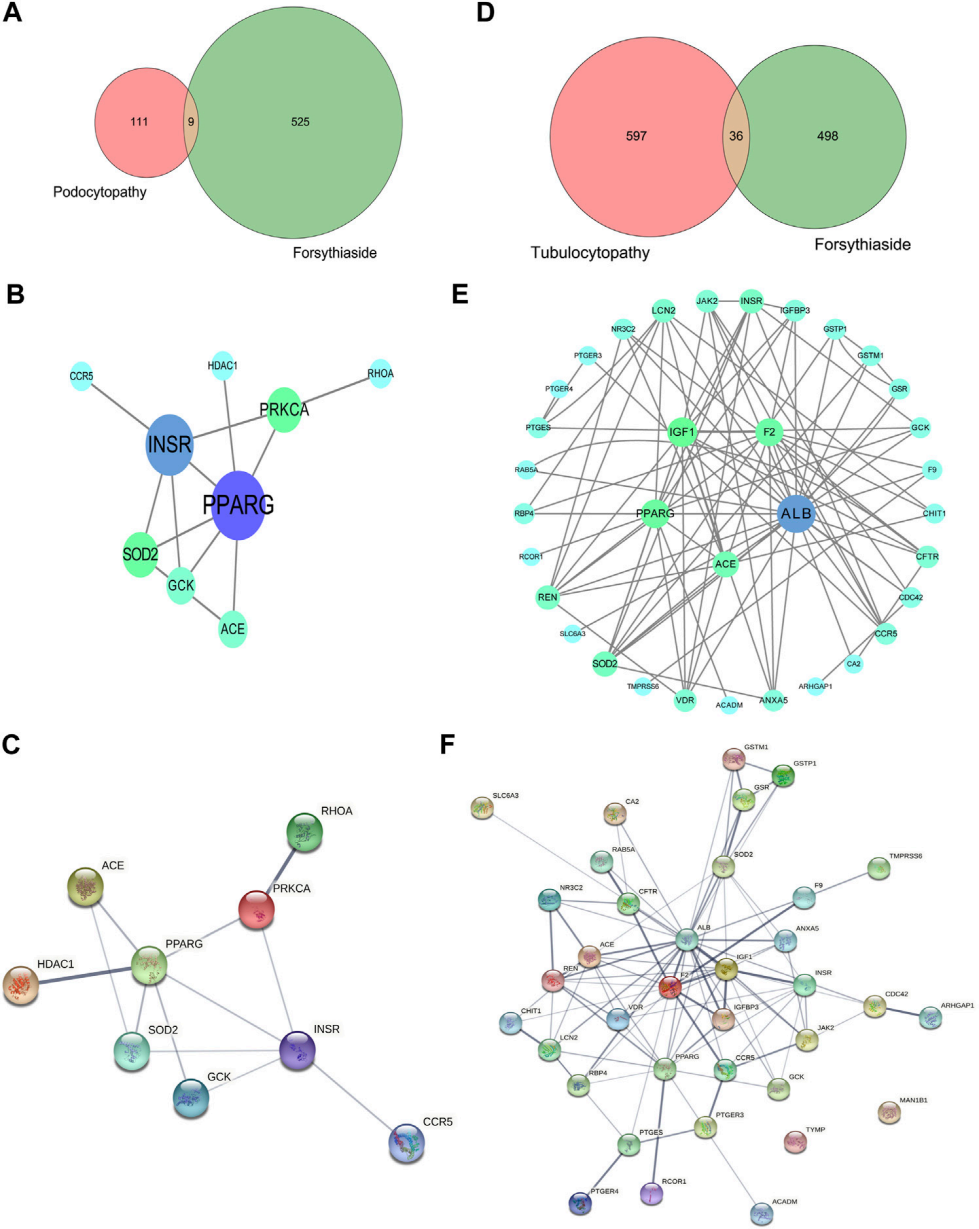
Venn diagram and PPI network of Forsythiaside-disease targets. **(A)** Venn diagram of overlapping targets of Forsythiaside and diabetic podocytopathy. **(B)** PPI network of Forsythiaside-diabetic podocytopathy targets. Nine overlapping targets were presented as circle and ordered by the degree level. **(C)** The PPI network which reflected the interaction between nine overlapping targets was displayed by STRING website. **(D)** Venn diagram of overlapping targets of Forsythiaside and diabetic tubulopathy. **(E)** PPI network of Forsythiaside-diabetic tubulopathy targets. Thirty-six overlapping targets were presented as circle and ordered by the degree level. Five genes were of significance in the network, including ALB, PPARG, IGF1, F2 and ACE. **(F)** The PPI network analysis of 36 overlapping targets between Forsythiaside and diabetic tubulopathy was performed by STRING website.

A Venn diagram was drawn to identify the overlapping targets between Forsythiaside and diabetic podocytopathy (Figure 5A). Nine overlapping targets were identified and arranged based on the degree level, including PPARG, INSR, PRKCA, SOD2, GCK, ACE, RHOA, HDAC1, and CCR5 (Figure 5B). Additionally, the interaction relationship between nine overlapping targets were further investigated. And the PPI network which reflected the interaction between these targets was displayed in Figure 5C.

Similarly, the overlapping genes between Forsythiaside and diabetic tubulopathy were selected. A total of 36 genes were identified, as shown in a Venn diagram (Figure 5D). A circle network was drawn and genes were ordered by their degree levels. Five genes were of significance in this network, including ALB, PPARG, IGF1, F2, and ACE (Figure 5E). PPI network analysis was also performed; the result is shown in Figure 5F.

### 3.6 GO and KEGG pathway enrichment analysis of overlapping genes of forsythiaside-diabetic podocytopathy and forsythiaside-diabetic tubulopathy

**FIGURE 6:**
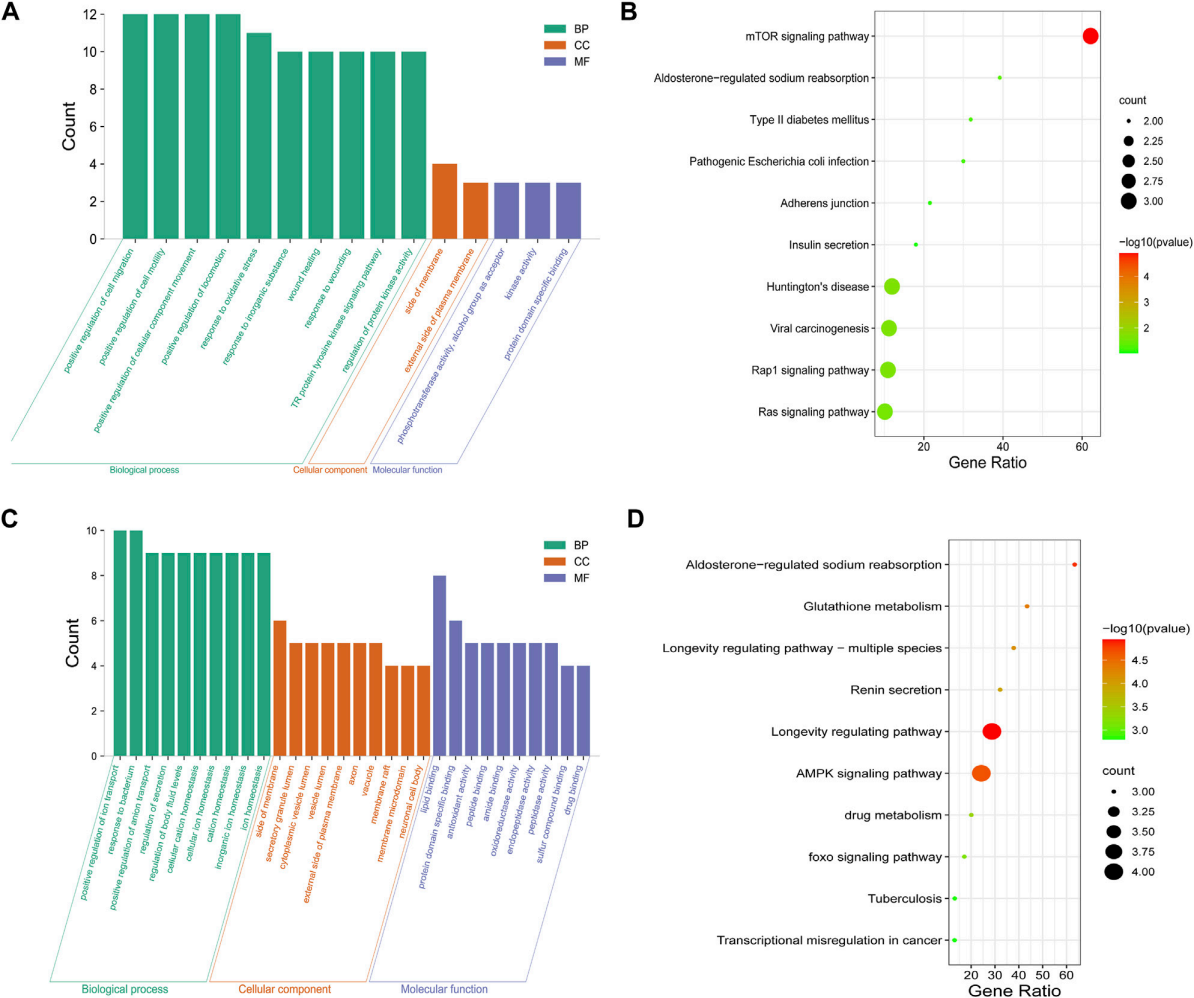
GO and KEGG enrichment of Forsythiaside-disease targets. **(A)** GO and **(B)** KEGG enrichment analysis of potential targets that Forsythiaside acting on diabetic podocytopathy. **(C)** GO and **(D)** KEGG enrichment analysis of potential targets that Forsythiaside acting on diabetic tubulopathy.

For clarifying the potential mechanism of Forsythiaside on diabetic podocytopathy and tubulopathy systematically, enrichment analyses were conducted with the 9 and 36 discovered overlapping genes, respectively. For diabetic podocytopathy, the top GO items were the response to oxidative stress, cellular components of side of membrane, and molecular function of kinase activity (Figure 6A). The top KEGG pathways were the mTOR signaling pathway, Type II diabetes mellitus, and Rap1 and Ras signaling pathway (Figure 6B). For diabetic tubulopathy, the overlapping targets were primarily enriched in the positive regulation of ion transport, cellular components of side of membrane, and molecular function of lipid binding (Figure 6C). Additionally, the top KEGG enrichments were associated with the longevity-regulating and AMPK signaling pathways (Figure 6D).

### 3.7 Molecular docking analysis

**FIGURE 7:**
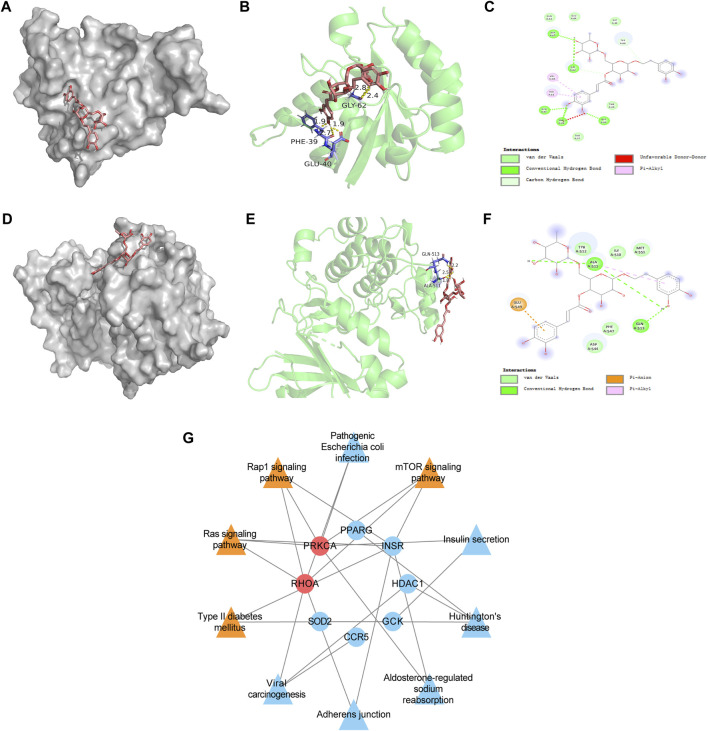
Molecular docking data of RHOA target and PRKCA target and compound-target-pathway network of Forsythiaside and diabetic podocytopathy. **(A)** Schematics (3D) showed that molecular model of Forsythiaside fitting in the binding pocket of the RHOA protein. **(B)** Schematics (3D) represented the interactions between Forsythiaside and surrounding residues of RHOA. The yellow dashed lines showed hydrogen bonds and the interaction distances were indicated. **(C)** Schematics (2D) revealed the interaction forces between Forsythiaside and RHOA. **(D)** Schematics (3D) represented that molecular model of Forsythiaside fitting in the binding pocket of the PRKCA protein. **(E)** Schematics (3D) showed the interactions between Forsythiaside and surrounding residues of PRKCA. The yellow dashed lines showed hydrogen bonds and the interaction distances were indicated. **(F)** Schematics (2D) revealed the interaction forces between Forsythiaside and PRKCA. **(G)** The compound-target-pathway network was established to explore the potential mechanisms of Forsythiaside treating diabetic podocytopathy.

After conducting enrichment analysis, we found that the enrichment pathways of the overlapping targets of Forsythiaside and DKD were consistent with those of Forsythiaside and diabetic podocytopathy, suggesting that Forsythiaside alleviated DKD mainly through its action on podocytes. Thus, molecular docking simulation was performed to verify the possibility of Forsythiaside binding to the targets in diabetic podocytopathy. The potential interaction between 9 overlapping genes and Forsythiaside was explored with molecular docking verification. And the complexes of retrieved targets and Forsythiaside were filtered by the docking affinity values which were reported by AutoDock. The greater absolute value of minus value of the docking affinity showed the stronger binding affinity of Forsythiaside and the targets. Total nine docking complexes were analyzed and the models of binding complexes were displayed. The number of conformations analyzed for docking was 50. And the corresponding free energy of binding and the putative affinity were summarized in Table 2. Docking data suggested strong binding affinity of RHOA- Forsythiaside docking (−1.14 kcal/mol, Figures 7A–C) and PRKCA- Forsythiaside docking (−.15 kcal/mol, Figures 7D–F), while other 7 targets showed weak binding affinities with Forsythiaside. To be specific, for RHOA- Forsythiaside docking complex, small molecule ligand Forsythiaside could interact with the residue of RHOA protein *via* a wide range of interaction forces. As shown in Figure 7A, Forsythiaside could insert into the interface pocket of RHOA protein. Docking complex displayed that five hydrogen bonds were formed between Forsythiaside and RHOA’s residues including PHE-39, GLU-40, and GLY-62 residue of RHOA. And the distances of hydrogen bonds and Forsythiaside were 2.8, 2.4, and 2.7, 1.9 and 1.9 Å, respectively (Figure 7B). Other interaction forces were also found in the Forsythiaside -RHOA docking complex. ALA-61 and VAL-38 residue contributed to form pi-alkyl interactions with Forsythiaside. GLN-63, GLU-64, GLY-14, THR-37, and THR-19 residue interacted with Forsythiaside *via* van der Waals forces. The carbon hydrogen bond and unfavorable donor-donor forces were also found in the interaction of Forsythiaside with TYR-66 and PHE-39 residue, respectively (Figure 7C).

In the PRKCA-Forsythiaside docking complex, Forsythiaside could potentially fit into the interface pocket of the PRKCA protein (Figure 7D). The complex showed that three hydrogen bonds were formed between Forsythiaside and GLN-513 and ALA-511 of PRKCA. Interactions between the three hydrogen bonds are shown in Figure 7E with a distance of 2.5, 2.2, and 1.8 Å, respectively. Moreover, the GLU-543 residue contributed to form pi-anion, and the ALA-511 residue contributed to form pi-alkyl, interactions with Forsythiaside. The other residues, such as TYR-512, ILE-510 and MET-551, PHE-547, and ASP-544 interacted with Forsythiaside *via* van der Waals forces (Figure 7F). Consequently, Forsythiaside stably bound to PRKCA and RHOA proteins through multiple interactive forces.

### 3.8 Compound-target-pathway network

As shown in Figure 7G compound-target-pathway network was established to explore the potential mechanism of Forsythiaside treatment of diabetic podocytopathy. Two targets, PRKCA and RHOA proteins, and four signaling pathways, including the mTOR signaling pathway, Type II diabetes mellitus, and Ras and Rap1 signaling pathway, were predicted and considered as key nodes and pathways through which Forsythiaside ameliorated DKD podocytopathy.

### 3.9 Forsythiaside relieved impaired viability of podocytes induced by high glucose treatment

**FIGURE 8:**
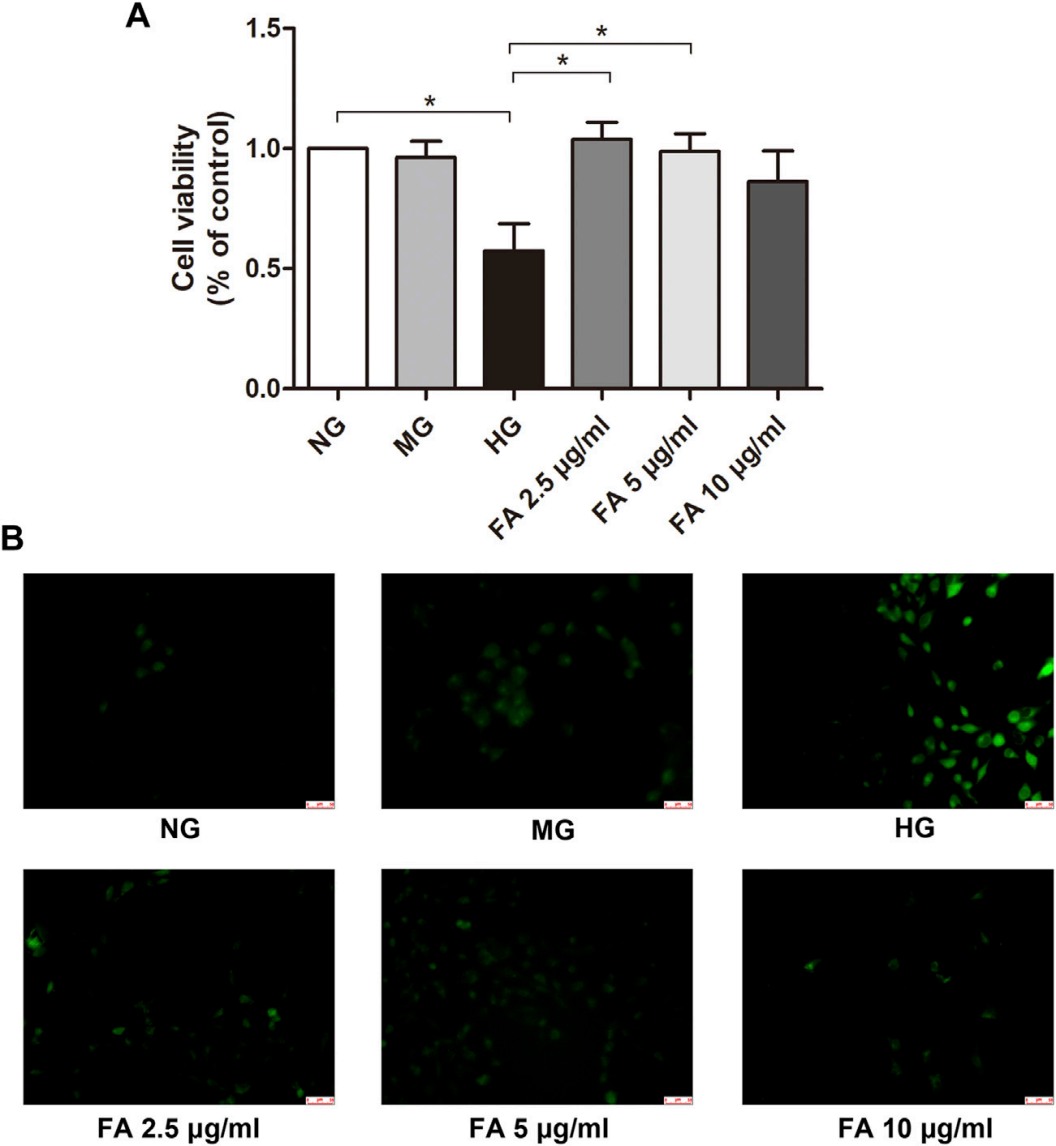
Assessment of podocyte viability by Cell Counting Kit-8 assay and reactive oxidative species measurement by fluorescence. **(A)** Podocyte viability was examined by Cell Counting Kit-8 assay after various treatments for 48 h. **(B)** Reactive oxidative species which were labeled with green fluorescence were detected after podocytes were exposed to normal glucose (NG), isosmotic mannitol (MG), or high glucose (HG) for 48 h with or without Forsythiaside (FA 2.5 μg/mL, FA 5 μg/mL, and FA 10 μg/mL) treatment. Magnification: ×200. Scale bar = 50 µm. Data are mean ± S.E.M. *p < .05. FA: Forsythiaside.

To investigate the direct effect and drug toxicity of Forsythiaside on podocytes, we performed a CCK-8 assay. Firstly, cell viability of podocytes was examined after high glucose stimulation. As shown in Figure 8A, cell viability declined in the HG podocyte group compared to the NG podocyte group (*p* < .05). Cell viability was further assessed in the HG + FA group. Results showed that the viability of podocytes in this group were not significantly different when compared with podocytes in the NG group (*p* > .05, Figure 8A), indicating that the concentrations of Forsythiaside used in the experiment were non-toxic. Additionally, we found that there was a significant increase in viability in media with 2.5 and 5 μg/mL Forsythiaside compared with the HG group (*p* < .05). However, treatment with 10 μg/mL Forsythiaside did not show significant increase of cell viability when compared with HG group (*p* > .05).

### 3.10 Forsythiaside significantly inhibited ROS production in podocytes under high glucose conditions

To clarify a potential mechanism for the renal protective effect of Forsythiaside, potential changes in ROS were measured with a reagent solution, DCFH-DA, which fluoresced upon oxidation by ROS. As shown in Figure 8B, increased ROS production was observed in podocytes in the HG group compared to those in the NG and MG groups, manifesting as green fluorescence. However, Forsythiaside significantly reduced the HG-induced increase in ROS. These results suggest a potential nephroprotective effect of Forsythiaside related to the inhibition of oxidative stress inhibition in podocytes, consistent with our network analysis; findings are described in Section 3.6.

### 3.11 Forsythiaside suppressed the expression of PRKCA and RHOA in podocytes treated with high glucose

**FIGURE 9:**
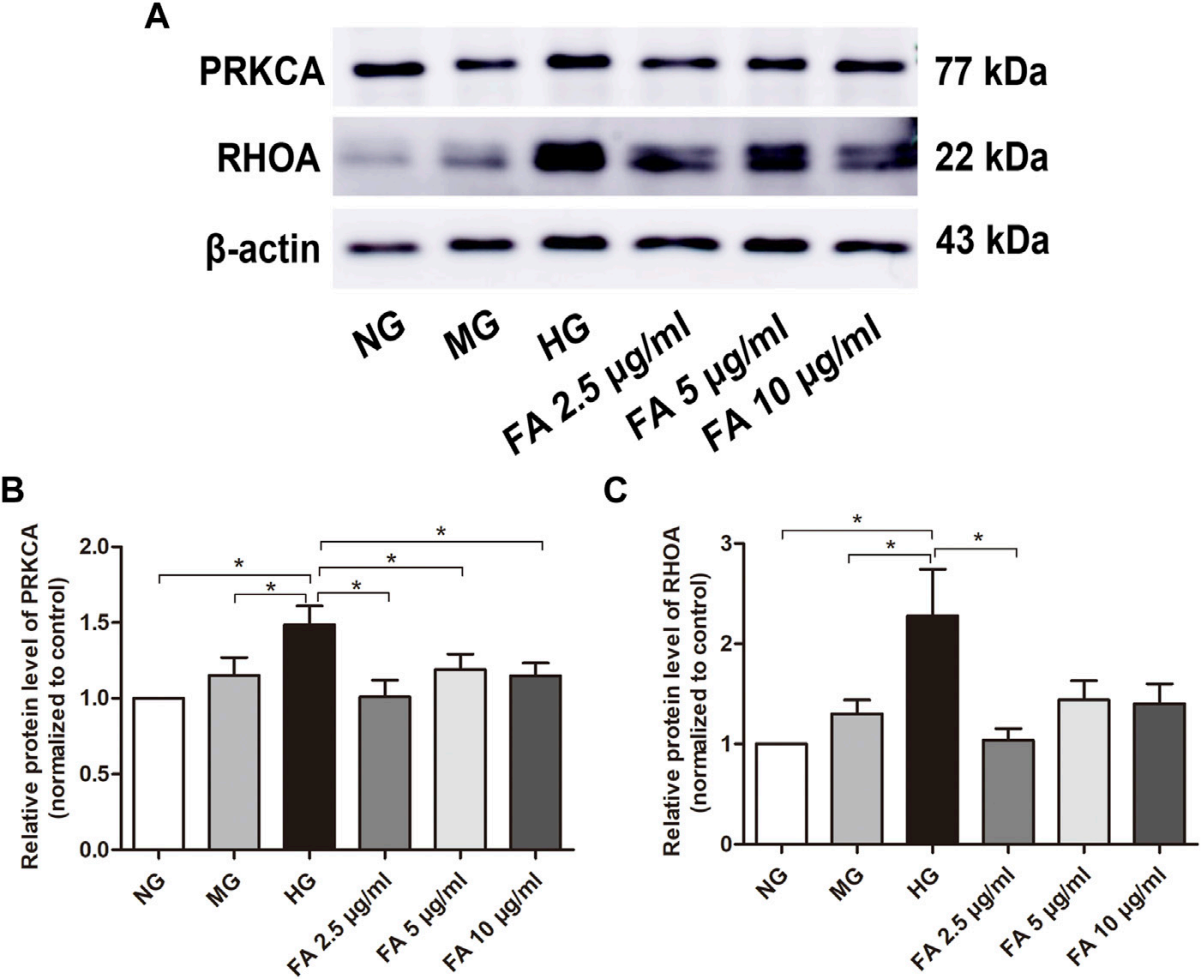
Examination of PRKCA and RHOA protein expression levels after Forsythiaside treatment. **(A)** The protein levels of PRKCA and RHOA in podocytes were examined by Western blotting. **(B)** Quantification of PRKCA protein expression in podocytes after various treatments. **(C)** Quantification of RHOA protein expression in podocytes after different treatments. Values are expressed as mean ± S.E.M. *p < .05. NG: normal glucose; MG: isosmotic mannitol; HG: high glucose; FA: Forsythiaside.

As shown in Figures 9A,B marked increase in expression of PRKCA protein in podocytes was detected in the HG group when compared to the NG and MG groups, as detected by western blotting (*p* < .05), while treatment with Forsythiaside in HG medium visibly suppressed the expression of PRKCA (*p* < .05). Additionally, western blotting results showed that the expression of RHOA was significantly upregulated in podocytes in the HG group compared with those in the NG and MG groups (*p* < .05), which was in accordance with the results of PRKCA protein. It is worth noting that 2.5 μg/mL Forsythiaside treatment markedly inhibited the expression of RHOA protein compared with the HG group (*p* < .05, Figures 9A,C). However, there was no significant decrease of RHOA protein expression in 5 μg/mL and 10 μg/mL Forsythiaside groups (Figure 9C). The results of western blotting confirmed the network analysis and molecular docking findings described in Sections 3.5, 3.7. Taken together, our results demonstrate the potential protective mechanism of Forsythiaside under high glucose conditions may be attributed to the suppression of a PRKCA and RHOA-related pathway.

## 4 Discussion

Of the common chronic complications of diabetes, DKD in one of the heaviest burdens for patients (Thomas et al., 2015). Conventional therapies such as intensive control of blood glucose, pressure, and lipids have not effectively inhibited DKD progression (Ahlqvist et al., 2015). Additional factors including oxidative stress, inflammation, and metabolic alteration have also been verified to influence the occurrence and development of DKD (Lehtonen, 2020). Current advances in treatment for DKD show few benefits in patient prognosis and survival. Thus, key pathways that may be involved in the progression of DKD must be investigated in order to identify effective treatments for DKD.

Bioactive constituents extracted from a variety of medicinal plants are considered more effective than synthetic medications for the management of multiple chronic diseases (Gorji et al., 2018; Mao et al., 2019). Therefore, natural products are regarded to have potential applications in the treatment of DKD. Forsythiaside has been shown to be an effective compound for the prevention and treatment of chronic disease (Cheng et al., 2015). In recent years, studies on the pharmacological effects of Forsythiaside have gradually increased. These show that Forsythiaside has anti-oxidation (Huang et al., 2015), anti-inflammation (Chen L. et al., 2018), and immunoregulatory (Cheng et al., 2014) properties. Studies have shown that one of the core mechanisms of DKD development is the accumulation of ROS, inflammatory factors, and immune complexes in the kidney, which further activate cytotoxic signaling in the kidney, leading to renal dysfunction (Huang et al., 2020). Forsythiaside may protect against DKD by intervening these mechanisms. However, to our knowledge there are few studies on the material basis and mechanism of the prevention and treatment of DKD by Forsythiaside.

In this study, we found that Forsythiaside was effective in preventing and treating DKD by performing network analysis. The pathways of Forsythiaside in the treatment of DKD were mTOR signal pathway and Type II diabetes mellitus. Our findings revealed that Forsythiaside could not only intervene various targets to regulate the same pathway, but also show the ability to act on the same target to control the multiple pathways for management of DKD, preliminarily indicating that the natural compound exerts multi-target and multi-pathway regulating effect on the improvement of the chronic and complicated disease.

A great number of cell types are involved in the pathogenesis of DKD. Deciphering the molecular mechanisms that regulate the occurrence and development of DKD, and exploring effective therapeutic measures for delaying renal impairment are essential. As the two inherent components of the kidney, the renal tubule and glomerular podocytes are the most frequently studied biological units in renal dysfunction. The proximal renal tubule is sensitive to various pathogenic factors, including metabolic and hemodynamic stimuli which induce the apoptosis of tubular cells and further lead to the progression of tubulo-interstitial lesions in DKD (Habib, 2013). Glomerular podocytes are the primary mediators of the integrity and normal function of the glomerular filtration barrier. Damage to podocytes causes detachment and dysfunction, which further promotes DKD progression (Pagtalunan et al., 1997; Steffes et al., 2001).

For clarifying the exact mechanism from the cellular aspect, the genes and pathways that Forsythiaside intervened were mapped to the targets and pathways of diabetic podocytopathy and tubulopathy by construction of network. For exploring how Forsythiaside treated DKD from the cellular level, we analyzed the overlapping pathways and related genes that Forsythiaside played an important role in the pathogenesis of DKD and diabetic podocytopathy or tubulopathy simultaneously. And the overlapping pathways, including mTOR signaling pathway and Type II diabetes mellitus were identified as the key pathways that Forsythiaside ameliorated DKD by improving podocytopathy. Few overlapping pathways were observed between DKD and diabetic tubulopathy in which Forsythiaside worked, indicating the mechanism the Forsythiaside alleviated DKD was mainly involved in the improvement of podocytopathy other than tubulopathy.

Further molecular docking simulation was performed to elucidate the specific target protein in podocytopathy that Forsythiaside worked to intervene for the amelioration of DKD, and we found that Forsythiaside could play a key role in the treatment of DKD podocytopathy by targeting RHOA and PRKCA protein.

Ras homolog family member A (RHOA) belongs to the family of Small GTPase. The primary function of RHOA is mainly related with cytoskeleton organization when binding to a variety of effector proteins, and then is responsible for control of cellular responses (Kloc et al., 2019). Researches revealed that RHOA exerted the detrimental effects on the progression of DKD. The suppression of the RHOA/ROCK pathway also possessed a protective capability for glomerular inflammation and fibrosis in diabetic rats (Hirose et al., 2010). Another research indicated that catalpol rescued disrupted cytoskeleton in podocytes under high glucose, which was mainly attributable to the inhibition of RHOA activities (Rao et al., 2017). Consistent with previous studies, our research revealed that Forsythiaside inhibited the expression of RHOA protein in podocytes. Therefore, we inferred that RHOA may be involved in the dysfunction of podocytes in DKD pathogenesis and Forsythiaside could target and intervene RHOA for management of diabetic podocytopathy.

Protein kinase C alpha (PRKCA), another target of Forsythiaside we acquired from network analysis and molecular docking in this study, is a serine/threonine-protein kinase which is involved in the regulation of a variety of cellular processes by activating signaling cascade involving mTOR pathway (Bhalla et al., 2017). Previous studies revealed that hyperglycemia induced the activation of PKC system which played a vital role in DKD glucotoxicity (Craven and DeRubertis, 1989; Lee et al., 1989) and the abnormal modulation of renal function (Chen et al., 2004). Activation of PRKCA has been implicated as a central mediator in the pathogenesis of DKD and the development of albuminuria (Kang et al., 1999). The inhibition of PRKCA exerted the beneficial effect in DKD (Parameswaran et al., 2000; Koya, 2014). Combined with our findings, Forsythiaside ameliorated the detrimental effects of podocyte damage under HG condition possibly by preventing PRKCA upregulation.

The targets that Forsythiaside bound and intervened were primarily identified, whereas the underlying mechanism is still unclear. Further Go enrichment analysis was performed and the results showed that Forsythiaside could relieve oxidative stress and improve kinase activity in DKD podocytes. Dysfunction of podocytes has been verified as a critical driving factor for DKD occurrence and development (Bid et al., 2013; Reiser and Sever, 2013). Podocyte loss which induced glomerular damage and glomerulosclerosis may result from excess generation of ROS which further caused podocyte apoptosis (Susztak et al., 2006). Thus, prevention of redox reactions is probably beneficial for podocyte survival in DKD. Forsythiaside as the active component of could inhibit the increased ROS induced by hydrogen peroxide (Huang et al., 2015). RHOA was reported to play a vital role of induing excess oxidative stress (Bhalla et al., 2017) and Sanggenon C alleviated cerebral injury by suppressing oxidative stress *via* controlling RHOA signaling pathway (Shimokawa et al., 2016). Additionally, the role for PRKCA which controlled oxidative phosphorylation was identified in nephrotoxic nephritis mice (Kvirkvelia et al., 2018), suggesting that PRKCA was essential in the regulation of oxidative stress. Combined with the findings which Forsythiaside inhibited the expressions of RHOA and PRKCA proteins, and markedly alleviated the ROS accumulation, we supposed that Forsythiaside could act on the renal podocytes and suppress oxidative stress in diabetic podocytopathy *via* intervening RHOA and PRKCA.

Furthermore, our study clarified that Forsythiaside alleviated the detrimental effect of hyperglycemia on podocytes in diabetic podocytopathy possibly by inhibiting the mTOR signaling pathway. Research revealed that Everolimus, an inhibitor of mTOR remodeled aberrant podocyte behavior by substantial increase of RHOA activity and enhanced actin stress fibers (Jeruschke et al., 2013), suggesting that RHOA was involved in the regulation of mTOR signaling pathway, and the potential roles in DKD pathogenesis was preliminarily elucidated in our network analysis study. And PRKCA could also modulate a wide range of cellular processes by activating mTOR signaling pathways. These studies suggested that the pretreatment of Forsythiaside could efficiently act on the kidney to keep podocytes functioning normally through targeting RHOA and PRKCA and could be developed as a potential natural antioxidant and therapy for DKD podocyte injury by controlling mTOR signaling pathways.

In conclusion, by using network analysis, we found that Forsythiaside intervened DKD by mainly targeting diabetic podocytopathy. Furthermore, our study elucidated that Forsythiaside exerted potential treatment against DKD which may act directly RHOA and PRKCA target by suppressing the oxidative stress pathway in podocytes, which preliminarily reflected the characteristics of multi-target and multi-action pathway of Forsythiaside in treating DKD from the point of view of cellular precision therapy. Our findings could offer a reference for investigation of therapeutic benefits of Forsythiaside in DKD from the cellular facet. However, systematic studies of Forsythiaside intervention on DKD are still insufficient. Furthermore, limitations existed in the study. The lack of comprehensiveness in current network information, and the delayed update of database may become the inevitable limitations. The explanation of data should be more careful. Therefore, it is of great clinical significance to study the exact mechanism of Forsythiaside against DKD podocytopathy and to further develop related pharmaceutical preparations in future, and future research study should focus on the therapeutic interventions of Forsythiaside for DKD patients in clinic.

## Data Availability

The datasets presented in this study can be found in online repositories. The names of the repository/repositories and accession number(s) can be found in the article/Materials and methods section.

## Supplementary material

SUPPLEMENTARY FIGURE S1 The conformation of Forsythiaside which was acquired from PubChem database (Pubchem CID: 5281773).

## Nomenclature

DKD: diabetic kidney disease
FA: Forsythiaside A powder
HG: podocytes incubated in media with 40 mM D-glucose for 48 h
HG + FA: podocytes incubated in media with 40 mM D-glucose and varying concentrations of Forsythiaside (2.5, 5, and 10 μg/mL) for 48 h
MG: podocytes incubated in media with 5.5 mM D-glucose and 34.5 mM D-mannitol for 48 h
NG: podocytes incubated in media with 5.5 mM D-glucose for 48 h
OMIM: Online Mendelian Inheritance in Man database
PKC: protein-kinase C
PPI: protein-protein interaction
PRKCA: protein kinase C alpha
ROS: reactive oxygen species
TCM: traditional Chinese medicine
