# Supplementary material for: Cellular mechanism of action of forsythiaside for the treatment of diabetic kidney disease
Source: Front Pharmacol. 2023 Jan 13;13:1096536. doi: 10.3389/fphar.2022.1096536 (PMC9880420; doi:10.3389/fphar.2022.1096536)
Supplement: Supplementary file 1 [file Table1.DOCX]

**Table S1.** The putative targets of Forsythiaside.

| **Number** | **Gene name** | **Protein name** | **Database 1** | **Database 2** | **Database 3** | **Database 4** |
| --- | --- | --- | --- | --- | --- | --- |
| 1 | ABCC1 | Multidrug resistance-associated protein 1 | ChEMBL |  |  |  |
| 2 | ABL1 | Tyrosine-protein kinase ABL | SwissTargetPrediction |  |  |  |
| 3 | ABO | Histo-blood group ABO system transferase | PharmMapper |  |  |  |
| 4 | ACADM | Medium-chain specific acyl-CoA dehydrogenase, mitochondrial | PharmMapper |  |  |  |
| 5 | ACAT1 | Acetyl-CoA acetyltransferase, mitochondrial | PharmMapper |  |  |  |
| 6 | ACE | Angiotensin-converting enzyme | SwissTargetPrediction | ChEMBL |  |  |
| 7 | ACE2 | Angiotensin-converting enzyme 2 | PharmMapper |  |  |  |
| 8 | ACHE | Acetylcholinesterase | ChEMBL |  |  |  |
| 9 | ACP3 | Prostatic acid phosphatase | PharmMapper |  |  |  |
| 10 | ADAM10 | Disintegrin and metalloproteinase domain-containing protein 10 | ChEMBL |  |  |  |
| 11 | ADAM17 | Disintegrin and metalloproteinase domain-containing protein 17 | SwissTargetPrediction | ChEMBL | PharmMapper |  |
| 12 | ADH1B | Alcohol dehydrogenase 1B | PharmMapper |  |  |  |
| 13 | ADH1C | Alcohol dehydrogenase 1C | PharmMapper |  |  |  |
| 14 | ADH5 | Alcohol dehydrogenase class-5 | PharmMapper |  |  |  |
| 15 | ADK | Adenosine kinase | SwissTargetPrediction | ChEMBL | PharmMapper |  |
| 16 | ADORA1 | Adenosine A1 receptor | ChEMBL |  |  |  |
| 17 | ADORA2A | Adenosine A2a receptor | SwissTargetPrediction | ChEMBL | PharmMapper |  |
| 18 | ADORA2B | Adenosine A2b receptor | SwissTargetPrediction | ChEMBL |  |  |
| 19 | ADORA3 | Adenosine A3 receptor | SwissTargetPrediction | ChEMBL |  |  |
| 20 | ADRA1D | Alpha-1d adrenergic receptor | ChEMBL |  |  |  |
| 21 | ADRA2A | Alpha-2a adrenergic receptor | ChEMBL |  |  |  |
| 22 | ADRA2B | Alpha-2b adrenergic receptor | ChEMBL |  |  |  |
| 23 | ADRA2C | Alpha-2c adrenergic receptor | ChEMBL |  |  |  |
| 24 | ADRB1 | Beta-1 adrenergic receptor | ChEMBL |  |  |  |
| 25 | ADRB2 | Beta-2 adrenergic receptor | ChEMBL |  |  |  |
| 26 | ADRB3 | Beta-3 adrenergic receptor | ChEMBL |  |  |  |
| 27 | AGXT | Serine--pyruvate aminotransferase | PharmMapper |  |  |  |
| 28 | AHCY | Adenosylhomocysteinase | PharmMapper |  |  |  |
| 29 | AKR1B1 | Aldose reductase | SwissTargetPrediction | ChEMBL | PharmMapper |  |
| 30 | AKR1B10 | Aldo-keto reductase family 1 member B10 | SwissTargetPrediction | ChEMBL |  |  |
| 31 | AKR1C2 | Aldo-keto reductase family 1 member C2 | SwissTargetPrediction | PharmMapper |  |  |
| 32 | AKR1C4 | Aldo-keto reductase family 1 member C4 | SwissTargetPrediction |  |  |  |
| 33 | AKT1 | Serine/threonine-protein kinase AKT | ChEMBL | PharmMapper |  |  |
| 34 | AKT3 | Serine/threonine-protein kinase AKT3 | ChEMBL |  |  |  |
| 35 | ALB | Serum albumin | SwissTargetPrediction | PharmMapper |  |  |
| 36 | ALDH2 | Aldehyde dehydrogenase | SwissTargetPrediction | PharmMapper |  |  |
| 37 | ALDOA | Fructose-bisphosphate aldolase A | PharmMapper |  |  |  |
| 38 | ALK | ALK tyrosine kinase receptor | ChEMBL |  |  |  |
| 39 | ALOX5AP | 5-lipoxygenase activating protein | ChEMBL |  |  |  |
| 40 | ALPL | Alkaline phosphatase, tissue-nonspecific isozyme | ChEMBL |  |  |  |
| 41 | AMD1 | S-adenosylmethionine decarboxylase proenzyme | PharmMapper |  |  |  |
| 42 | AMPD3 | AMP deaminase 3 | SwissTargetPrediction |  |  |  |
| 43 | AMY1A | Alpha-amylase 1 | PharmMapper |  |  |  |
| 44 | AMY2A | Pancreatic alpha-amylase | PharmMapper |  |  |  |
| 45 | ANG | Angiogenin | PharmMapper |  |  |  |
| 46 | ANXA5 | Annexin A5 | PharmMapper |  |  |  |
| 47 | AOC3 | Amine oxidase, copper containing | ChEMBL |  |  |  |
| 48 | APCS | Serum amyloid P-component | PharmMapper |  |  |  |
| 49 | APH1A | Gamma-secretase subunit APH-1A | SwissTargetPrediction |  |  |  |
| 50 | APH1B | Gamma-secretase | ChEMBL |  |  |  |
| 51 | APP | Beta amyloid A4 protein | SwissTargetPrediction | ChEMBL |  |  |
| 52 | APRT | Adenine phosphoribosyltransferase | PharmMapper |  |  |  |
| 53 | AR | Androgen Receptor | ChEMBL | PharmMapper |  |  |
| 54 | ARF1 | ADP-ribosylation factor 1 | PharmMapper |  |  |  |
| 55 | ARG2 | Arginase-2, mitochondrial | PharmMapper |  |  |  |
| 56 | ARHGAP1 | Rho GTPase-activating protein 1 | PharmMapper |  |  |  |
| 57 | ATM | Serine-protein kinase ATM | ChEMBL |  |  |  |
| 58 | ATOX1 | Copper transport protein ATOX1 | PharmMapper |  |  |  |
| 59 | AURKA | Serine/threonine-protein kinase Aurora-A | SwissTargetPrediction |  |  |  |
| 60 | AVPR1A | Vasopressin V1a receptor | ChEMBL |  |  |  |
| 61 | AZGP1 | Zinc-alpha-2-glycoprotein | PharmMapper |  |  |  |
| 62 | B3GAT1 | Galactosylgalactosylxylosylprotein 3-beta-glucuronosyltransferase 1 | PharmMapper |  |  |  |
| 63 | BACE1 | Beta-secretase 1 | ChEMBL | PharmMapper |  |  |
| 64 | BAG1 | BAG family molecular chaperone regulator 1 | PharmMapper |  |  |  |
| 65 | BAX | Apoptosis regulator BAX | PUBCHEM |  |  |  |
| 66 | BCAT2 | Branched-chain-amino-acid aminotransferase, mitochondrial | ChEMBL | PharmMapper |  |  |
| 67 | BCHE | Butyrylcholinesterase | ChEMBL | PharmMapper |  |  |
| 68 | BCL2 | Apoptosis regulator Bcl-2 | PUBCHEM | ChemBL |  |  |
| 69 | BDKRB2 | Bradykinin B2 receptor | ChEMBL |  |  |  |
| 70 | BHMT | Betaine--homocysteine S-methyltransferase 1 | PharmMapper |  |  |  |
| 71 | BIRC7 | Baculoviral IAP repeat-containing protein 7 | PharmMapper |  |  |  |
| 72 | BLVRB | Flavin reductase | PharmMapper |  |  |  |
| 73 | BMP7 | Bone morphogenetic protein 7 | PharmMapper |  |  |  |
| 74 | BRAF | Serine/threonine-protein kinase B-raf | ChEMBL |  |  |  |
| 75 | BRAF | B-Raf proto-oncogene serine/threonine-protein kinase | PharmMapper |  |  |  |
| 76 | BRD4 | Bromodomain-containing protein 4 | ChEMBL |  |  |  |
| 77 | BRD9 | Bromodomain-containing protein 9 | ChEMBL |  |  |  |
| 78 | BRPF1 | Peregrin | ChEMBL |  |  |  |
| 79 | BST1 | ADP-ribosyl cyclase 2 | PharmMapper |  |  |  |
| 80 | BTK | Tyrosine-protein kinase BTK | SwissTargetPrediction |  |  |  |
| 81 | C1R | Complement C1r subcomponent | PharmMapper |  |  |  |
| 82 | C1S | Complement C1s subcomponent | PharmMapper |  |  |  |
| 83 | C8G | Complement component C8 gamma chain | PharmMapper |  |  |  |
| 84 | CA1 | Carbonic anhydrase I | SwissTargetPrediction | ChEMBL | PharmMapper |  |
| 85 | CA12 | Carbonic anhydrase XII | SwissTargetPrediction | ChEMBL | PharmMapper |  |
| 86 | CA14 | Carbonic anhydrase XIV | SwissTargetPrediction |  |  |  |
| 87 | CA2 | Carbonic anhydrase II | SwissTargetPrediction | ChEMBL | PharmMapper |  |
| 88 | CA4 | Carbonic anhydrase IV | SwissTargetPrediction |  |  |  |
| 89 | CA5B | Carbonic anhydrase VB | ChEMBL |  |  |  |
| 90 | CA7 | Carbonic anhydrase VII | SwissTargetPrediction | ChEMBL |  |  |
| 91 | CA9 | Carbonic anhydrase IX | SwissTargetPrediction | ChEMBL |  |  |
| 92 | CALCRL | Calcitonin gene-related peptide type 1 receptor | ChEMBL |  |  |  |
| 93 | CALM1 | Calmodulin | PharmMapper |  |  |  |
| 94 | CAPN1 | Calpain 1 | ChEMBL |  |  |  |
| 95 | CARM1 | Histone-arginine methyltransferase CARM1 | ChEMBL |  |  |  |
| 96 | CASP1 | Caspase-1 | SwissTargetPrediction | ChEMBL |  |  |
| 97 | CASP3 | Caspase-3 | PUBCHEM | Swifftarget prediction | ChemBL | PharmMapper |
| 98 | CASP6 | Caspase-6 | SwissTargetPrediction |  |  |  |
| 99 | CASP7 | Caspase-7 | SwissTargetPrediction | PharmMapper |  |  |
| 100 | CASP8 | Caspase-8 | SwissTargetPrediction | ChEMBL |  |  |
| 101 | CBR1 | Carbonyl reductase [NADPH] 1 | PharmMapper |  |  |  |
| 102 | CCKAR | Cholecystokinin A receptor | ChEMBL |  |  |  |
| 103 | CCKBR | Cholecystokinin B receptor | ChEMBL |  |  |  |
| 104 | CCL5 | C-C motif chemokine 5 | PharmMapper |  |  |  |
| 105 | CCNA2 | Cyclin-A2 | PharmMapper |  |  |  |
| 106 | CCNA2 | Cyclin-A2 | SwissTargetPrediction |  |  |  |
| 107 | CCNT1 | Cyclin-T1 | ChEMBL | PharmMapper |  |  |
| 108 | CCR2 | C-C chemokine receptor type 2 | ChEMBL |  |  |  |
| 109 | CCR3 | C-C chemokine receptor type 3 | ChEMBL |  |  |  |
| 110 | CCR4 | C-C chemokine receptor type 4 | ChEMBL |  |  |  |
| 111 | CCR5 | C-C chemokine receptor type 5 | ChEMBL |  |  |  |
| 112 | CCR9 | C-C chemokine receptor type 9 | ChEMBL |  |  |  |
| 113 | CD1A | T-cell surface glycoprotein CD1a | PharmMapper |  |  |  |
| 114 | CDA | Cytidine deaminase | PharmMapper |  |  |  |
| 115 | CDC42 | Cell division control protein 42 homolog | PharmMapper |  |  |  |
| 116 | CDC7 | Cell division cycle 7-related protein kinase | ChEMBL |  |  |  |
| 117 | CDK2 | Cyclin-dependent kinase 2 | SwissTargetPrediction | ChEMBL | PharmMapper |  |
| 118 | CDK4 | Cyclin-dependent kinase 4 | ChEMBL |  |  |  |
| 119 | CDK5R1 | Cyclin-dependent kinase 5 activator 1 | PharmMapper |  |  |  |
| 120 | CDK6 | Cell division protein kinase 6 | PharmMapper |  |  |  |
| 121 | CDK9 | CDK9/cyclin T1 | ChEMBL |  |  |  |
| 122 | CDKD-1 | Cyclin-dependent kinase 4/cyclin D1 | SwissTargetPrediction |  |  |  |
| 123 | CDKE-1 | Cyclin-dependent kinase 2/cyclin E1 | SwissTargetPrediction |  |  |  |
| 124 | CES1 | Liver carboxylesterase 1 | PharmMapper |  |  |  |
| 125 | CFB | Complement factor B | PharmMapper |  |  |  |
| 126 | CFD | Complement factor D | PharmMapper |  |  |  |
| 127 | CFTR | Cystic fibrosis transmembrane conductance regulator | ChEMBL |  |  |  |
| 128 | CHEK1 | Serine/threonine-protein kinase Chk1 | PharmMapper |  |  |  |
| 129 | CHIA | Acidic mammalian chitinase | SwissTargetPrediction |  |  |  |
| 130 | CHIT1 | Chitotriosidase-1 | PharmMapper |  |  |  |
| 131 | CHRM2 | Muscarinic acetylcholine receptor M2 | ChEMBL |  |  |  |
| 132 | CHRM3 | Muscarinic acetylcholine receptor M3 | ChEMBL |  |  |  |
| 133 | CHRM5 | Muscarinic acetylcholine receptor M5 | ChEMBL |  |  |  |
| 134 | CHRNA3 | Neuronal acetylcholine receptor subunit alpha-3 | ChEMBL |  |  |  |
| 135 | CHRNB1 | Acetylcholine receptor subunit beta | ChEMBL |  |  |  |
| 136 | CHRNB4 | Neuronal acetylcholine receptor subunit beta-4 | ChEMBL |  |  |  |
| 137 | CHRNG | Acetylcholine receptor; alpha1/beta1/delta/gamma | ChEMBL |  |  |  |
| 138 | CLK1 | Dual specificity protein kinase CLK1 | PharmMapper |  |  |  |
| 139 | CMA1 | Chymase | PharmMapper |  |  |  |
| 140 | CNR1 | Cannabinoid CB1 receptor | ChEMBL |  |  |  |
| 141 | CPB1 | Carboxypeptidase B | PharmMapper |  |  |  |
| 142 | CRAT | Carnitine O-acetyltransferase | PharmMapper |  |  |  |
| 143 | CREBBP | CREB-binding protein | ChEMBL |  |  |  |
| 144 | CSK | Tyrosine-protein kinase CSK | PharmMapper |  |  |  |
| 145 | CSNK1G2 | Casein kinase I isoform gamma-2 | PharmMapper |  |  |  |
| 146 | CSNK2A1 | Casein kinase II alpha | ChEMBL | PharmMapper |  |  |
| 147 | CSNK2B | Casein kinase II subunit beta | SwissTargetPrediction |  |  |  |
| 148 | CTNNA1 | Catenin alpha-1 | PharmMapper |  |  |  |
| 149 | CTSB | Cathepsin B | ChEMBL | PharmMapper |  |  |
| 150 | CTSD | Cathepsin D | PharmMapper |  |  |  |
| 151 | CTSG | Cathepsin G | PharmMapper |  |  |  |
| 152 | CTSK | Cathepsin K | ChEMBL | PharmMapper |  |  |
| 153 | CTSS | Cathepsin S | ChEMBL | PharmMapper |  |  |
| 154 | CTSV | Cathepsin L2 | PharmMapper |  |  |  |
| 155 | CXCR1 | Interleukin-8 receptor A | ChEMBL |  |  |  |
| 156 | CXCR2 | Interleukin-8 receptor B | ChEMBL |  |  |  |
| 157 | CYP1A2 | Cytochrome P450 1A2 | ChEMBL |  |  |  |
| 158 | CYP26A1 | Cytochrome P450 26A1 | ChEMBL |  |  |  |
| 159 | CYP2A6 | Cytochrome P450 2A6 | ChEMBL |  |  |  |
| 160 | CYP2C9 | Cytochrome P450 2C9 | PharmMapper |  |  |  |
| 161 | CYSLTR1 | Cysteinyl leukotriene receptor 1 | ChEMBL |  |  |  |
| 162 | CYSLTR2 | Cysteinyl leukotriene receptor 2 | ChEMBL |  |  |  |
| 163 | DAPK1 | Death-associated protein kinase 1 | PharmMapper |  |  |  |
| 164 | DCK | Deoxycytidine kinase | PharmMapper |  |  |  |
| 165 | DDX6 | Probable ATP-dependent RNA helicase DDX6 | PharmMapper |  |  |  |
| 166 | DHFR | Dihydrofolate reductase | SwissTargetPrediction | PharmMapper |  |  |
| 167 | DHODH | Dihydroorotate dehydrogenase, mitochondrial | PharmMapper |  |  |  |
| 168 | DPEP1 | Dipeptidase 1 | PharmMapper |  |  |  |
| 169 | DPP4 | Dipeptidyl peptidase IV | ChEMBL | PharmMapper |  |  |
| 170 | DPP8 | Dipeptidyl peptidase VIII | ChEMBL |  |  |  |
| 171 | DPP9 | Dipeptidyl peptidase IX | ChEMBL |  |  |  |
| 172 | DRD1 | Dopamine D1 receptor | ChEMBL |  |  |  |
| 173 | DRD2 | Dopamine D2 receptor | ChEMBL |  |  |  |
| 174 | DRD3 | Dopamine D3 receptor | ChEMBL |  |  |  |
| 175 | DRD4 | Dopamine D4 receptor | ChEMBL |  |  |  |
| 176 | DRD5 | Dopamine D5 receptor | ChEMBL |  |  |  |
| 177 | DTYMK | Thymidylate kinase | PharmMapper |  |  |  |
| 178 | DUSP6 | Dual specificity protein phosphatase 6 | PharmMapper |  |  |  |
| 179 | ECE1 | Endothelin-converting enzyme 1 | SwissTargetPrediction |  |  |  |
| 180 | EDNRA | Endothelin receptor ET-A | ChEMBL |  |  |  |
| 181 | EED | Polycomb protein EED | ChEMBL |  |  |  |
| 182 | EGFR | Epidermal growth factor receptor erbB1 | ChEMBL | PharmMapper |  |  |
| 183 | EHMT1 | Histone-lysine N-methyltransferase, H3 lysine-9 specific 5 | ChEMBL |  |  |  |
| 184 | EIF4A1 | Eukaryotic initiation factor 4A-I | SwissTargetPrediction |  |  |  |
| 185 | ELANE | Leukocyte elastase | ChEMBL | PharmMapper |  |  |
| 186 | EPHA2 | Ephrin type-A receptor 2 | SwissTargetPrediction | PharmMapper |  |  |
| 187 | EPHB4 | Ephrin type-B receptor 4 | PharmMapper |  |  |  |
| 188 | EPHX2 | Epoxide hydratase | SwissTargetPrediction | PharmMapper |  |  |
| 189 | ERBB2 | Receptor protein-tyrosine kinase erbB-2 | ChEMBL |  |  |  |
| 190 | ERBB4 | Receptor tyrosine-protein kinase erbB-4 | PharmMapper |  |  |  |
| 191 | ESR1 | Estrogen receptor alpha | SwissTargetPrediction | ChEMBL | PharmMapper |  |
| 192 | ESR2 | Estrogen receptor beta | ChEMBL | PharmMapper |  |  |
| 193 | ESRRA | Steroid hormone receptor ERR1 | PharmMapper |  |  |  |
| 194 | ESRRG | Estrogen-related receptor gamma | PharmMapper |  |  |  |
| 195 | F10 | Coagulation factor X | ChEMBL | PharmMapper |  |  |
| 196 | F11 | Coagulation factor XI | ChEMBL | PharmMapper |  |  |
| 197 | F13A1 | Coagulation factor XIII | ChEMBL |  |  |  |
| 198 | F2 | Thrombin | SwissTargetPrediction | PharmMapper |  |  |
| 199 | F3 | Coagulation factor VII/tissue factor | SwissTargetPrediction | ChEMBL |  |  |
| 200 | F7 | Coagulation factor VII | SwissTargetPrediction | ChEMBL | PharmMapper |  |
| 201 | F9 | Coagulation factor IX | ChEMBL |  |  |  |
| 202 | FAAH | Anandamide amidohydrolase | ChEMBL |  |  |  |
| 203 | FABP4 | Fatty acid-binding protein, adipocyte | PharmMapper |  |  |  |
| 204 | FABP5 | Fatty acid-binding protein, epidermal | PharmMapper |  |  |  |
| 205 | FABP6 | Gastrotropin | PharmMapper |  |  |  |
| 206 | FAP | Seprase | PharmMapper |  |  |  |
| 207 | FBP1 | Fructose-1,6-bisphosphatase | ChEMBL |  |  |  |
| 208 | FDPS | Farnesyl diphosphate synthase | ChEMBL | PharmMapper |  |  |
| 209 | FFAR1 | Free fatty acid receptor 1 | ChEMBL |  |  |  |
| 210 | FFAR2 | Free fatty acid receptor 2 | ChEMBL |  |  |  |
| 211 | FGFR1 | Fibroblast growth factor receptor 1 | SwissTargetPrediction | ChEMBL | PharmMapper |  |
| 212 | FGFR2 | Fibroblast growth factor receptor 2 | PharmMapper |  |  |  |
| 213 | FHIT | Bis(5'-adenosyl)-triphosphatase | SwissTargetPrediction | PharmMapper |  |  |
| 214 | FKBP1A | Peptidyl-prolyl cis-trans isomerase FKBP1A | PharmMapper |  |  |  |
| 215 | FLT1 | Vascular endothelial growth factor receptor 1 | ChEMBL |  |  |  |
| 216 | FLT4 | Vascular endothelial growth factor receptor 3 | SwissTargetPrediction |  |  |  |
| 217 | FNT | Protein farnesyltransferase | ChEMBL |  |  |  |
| 218 | FNTA | Protein farnesyltransferase/geranylgeranyltransferase type-1 subunit alpha | PharmMapper |  |  |  |
| 219 | FNTA | Protein farnesyltransferase/geranylgeranyltransferase type-1 subunit alpha | SwissTargetPrediction |  |  |  |
| 220 | FNTB | Protein farnesyltransferase subunit beta | SwissTargetPrediction |  |  |  |
| 221 | FUT6 | Fucosyltransferase 6 | SwissTargetPrediction |  |  |  |
| 222 | GABRG2 | GABA-A receptor; alpha-1/beta-2/gamma-2 | ChEMBL |  |  |  |
| 223 | GALE | UDP-glucose 4-epimerase | PharmMapper |  |  |  |
| 224 | GBA | Beta-glucocerebrosidase | SwissTargetPrediction | PharmMapper |  |  |
| 225 | GBA2 | Beta-glucosidase | SwissTargetPrediction |  |  |  |
| 226 | GCK | Glucokinase | PharmMapper |  |  |  |
| 227 | GLO1 | Lactoylglutathione lyase | PharmMapper |  |  |  |
| 228 | GM2A | Ganglioside GM2 activator | PharmMapper |  |  |  |
| 229 | GNPDA1 | Glucosamine-6-phosphate isomerase | PharmMapper |  |  |  |
| 230 | GP1BA | Platelet glycoprotein Ib alpha chain | PharmMapper |  |  |  |
| 231 | GPI | Glucose-6-phosphate isomerase | PharmMapper |  |  |  |
| 232 | GPR34 | Probable G-protein coupled receptor 34 (by homology) | SwissTargetPrediction |  |  |  |
| 233 | GPR55 | G-protein coupled receptor 55 | SwissTargetPrediction |  |  |  |
| 234 | GRIN1 | Glutamate NMDA receptor; GRIN1/GRIN2B | ChEMBL |  |  |  |
| 235 | GRIN2A | Glutamate receptor ionotropic,NMDA2A | SwissTargetPrediction |  |  |  |
| 236 | GRIN2B | Glutamate receptor ionotropic,NMDA2B | SwissTargetPrediction |  |  |  |
| 237 | GRM2 | Metabotropic glutamate receptor 2 | ChEMBL |  |  |  |
| 238 | GRM4 | Metabotropic glutamate receptor 4 | ChEMBL |  |  |  |
| 239 | GSK3B | Glycogen synthase kinase-3 beta | ChEMBL | PharmMapper |  |  |
| 240 | GSR | Glutathione reductase, mitochondrial | PharmMapper |  |  |  |
| 241 | GSTA1 | Glutathione S-transferase A1 | PharmMapper |  |  |  |
| 242 | GSTM1 | Glutathione S-transferase Mu 1 | PharmMapper |  |  |  |
| 243 | GSTP1 | Glutathione S-transferase P | PharmMapper |  |  |  |
| 244 | GSTT2B | Glutathione S-transferase theta-2 | PharmMapper |  |  |  |
| 245 | HAGH | Hydroxyacylglutathione hydrolase, mitochondrial | PharmMapper |  |  |  |
| 246 | HCAR2 | Hydroxycarboxylic acid receptor 2 | SwissTargetPrediction |  |  |  |
| 247 | HCK | Tyrosine-protein kinase HCK | PharmMapper |  |  |  |
| 248 | HCRTR1 | Orexin receptor 1 | ChEMBL |  |  |  |
| 249 | HDAC1 | Histone deacetylase 1 | SwissTargetPrediction | ChEMBL |  |  |
| 250 | HDAC10 | Histone deacetylase 10 | ChEMBL |  |  |  |
| 251 | HDAC3 | Histone deacetylase 3 | ChEMBL |  |  |  |
| 252 | HDAC6 | Histone deacetylase 6 | ChEMBL |  |  |  |
| 253 | HDAC8 | Histone deacetylase 8 | PharmMapper |  |  |  |
| 254 | HEXB | Beta-hexosaminidase subunit beta | PharmMapper |  |  |  |
| 255 | HINT1 | Histidine triad nucleotide-binding protein 1 | PharmMapper |  |  |  |
| 256 | HK1 | Hexokinase-1 | PharmMapper |  |  |  |
| 257 | HLCS | Biotin--protein ligase | SwissTargetPrediction |  |  |  |
| 258 | HMGCR | HMG-CoA reductase | ChEMBL | PharmMapper |  |  |
| 259 | HNMT | Histamine N-methyltransferase | PharmMapper |  |  |  |
| 260 | HPN | Serine protease hepsin | PharmMapper |  |  |  |
| 261 | HRH1 | Histamine H1 receptor | ChEMBL |  |  |  |
| 262 | HRH2 | Histamine H2 receptor | ChEMBL |  |  |  |
| 263 | HSD11B1 | Corticosteroid 11-beta-dehydrogenase isozyme 1 | PharmMapper |  |  |  |
| 264 | HSD17B1 | Estradiol 17-beta-dehydrogenase 1 | PharmMapper |  |  |  |
| 265 | HSD17B11 | Estradiol 17-beta-dehydrogenase 11 | PharmMapper |  |  |  |
| 266 | HSD17B2 | Estradiol 17-beta-dehydrogenase 2 | ChEMBL |  |  |  |
| 267 | HSD17B3 | Estradiol 17-beta-dehydrogenase 3 | ChEMBL |  |  |  |
| 268 | HSP90AA1 | Heat shock protein HSP 90-alpha | SwissTargetPrediction | ChEMBL | PharmMapper |  |
| 269 | HSPA1A | Heat shock 70 kDa protein 1 | PharmMapper |  |  |  |
| 270 | HSPA8 | Heat shock cognate 71 kDa protein | PharmMapper |  |  |  |
| 271 | HTR1B | Serotonin 1b (5-HT1b) receptor | ChEMBL |  |  |  |
| 272 | HTR1D | Serotonin 1d (5-HT1d) receptor | ChEMBL |  |  |  |
| 273 | HTR2A | Serotonin 2a (5-HT2a) receptor | ChEMBL |  |  |  |
| 274 | HTR3A | Serotonin 3a (5-HT3a) receptor | ChEMBL |  |  |  |
| 275 | HTR6 | Serotonin 6 (5-HT6) receptor | ChEMBL |  |  |  |
| 276 | ICAM2 | Intercellular adhesion molecule 2 | PharmMapper |  |  |  |
| 277 | ICMT | Isoprenylcysteine carboxyl methyltransferase | ChEMBL |  |  |  |
| 278 | IDH1 | Isocitrate dehydrogenase [NADP] cytoplasmic | ChEMBL |  |  |  |
| 279 | IDO1 | Indoleamine 2,3-dioxygenase | ChEMBL |  |  |  |
| 280 | IGF1 | Insulin-like growth factor IA | PharmMapper |  |  |  |
| 281 | IGF1R | Insulin-like growth factor 1 receptor | PharmMapper |  |  |  |
| 282 | IGFBP3 | Insulin-like growth factor binding protein 3 | SwissTargetPrediction |  |  |  |
| 283 | IGLV2-8 | Ig lambda chain V-II region MGC | PharmMapper |  |  |  |
| 284 | IKBKB | Inhibitor of nuclear factor kappa B kinase beta subunit | ChEMBL |  |  |  |
| 285 | IL2 | Interleukin-2 | PharmMapper |  |  |  |
| 286 | IMPA1 | Inositol monophosphatase | PharmMapper |  |  |  |
| 287 | IMPDH1 | Inosine-5'-monophosphate dehydrogenase 1 | SwissTargetPrediction | ChEMBL | PharmMapper |  |
| 288 | IMPDH2 | Inosine-5'-monophosphate dehydrogenase 2 | SwissTargetPrediction | ChEMBL | PharmMapper |  |
| 289 | INSR | Insulin receptor | PharmMapper |  |  |  |
| 290 | IRAK4 | Interleukin-1 receptor-associated kinase 4 | SwissTargetPrediction |  |  |  |
| 291 | ISG20 | Interferon-stimulated gene 20 kDa protein | PharmMapper |  |  |  |
| 292 | ITGA2B | Integrin alpha-IIb/beta-3 | SwissTargetPrediction |  |  |  |
| 293 | ITGA4 | Integrin alpha-4 | ChEMBL |  |  |  |
| 294 | ITGAV | Integrin alpha-V/beta-3 | SwissTargetPrediction |  |  |  |
| 295 | ITGB3 | Integrin beta-3 | SwissTargetPrediction |  |  |  |
| 296 | ITGB5 | Integrin alpha-V/beta-5 | ChEMBL |  |  |  |
| 297 | ITK | Tyrosine-protein kinase ITK/TSK | ChEMBL | PharmMapper |  |  |
| 298 | JAK2 | Tyrosine-protein kinase JAK2 | PharmMapper |  |  |  |
| 299 | JAK3 | Tyrosine-protein kinase JAK3 | PharmMapper |  |  |  |
| 300 | KDM1A | Lysine-specific histone demethylase 1 | ChEMBL |  |  |  |
| 301 | KDM5A | Lysine-specific demethylase 5A | ChEMBL |  |  |  |
| 302 | KDM5C | Lysine-specific demethylase 5C | ChEMBL |  |  |  |
| 303 | KDR | Vascular endothelial growth factor receptor 2 | SwissTargetPrediction | PharmMapper |  |  |
| 304 | KIF11 | Kinesin-like protein KIF11 | PharmMapper |  |  |  |
| 305 | KIT | Mast/stem cell growth factor receptor | PharmMapper |  |  |  |
| 306 | KLKB1 | Plasma kallikrein | ChEMBL |  |  |  |
| 307 | KMO | Kynurenine 3-monooxygenase | ChEMBL |  |  |  |
| 308 | KYAT1 | Kynurenine--oxoglutarate transaminase 1 | PharmMapper |  |  |  |
| 309 | LARS | Leucyl-tRNA synthetase | SwissTargetPrediction |  |  |  |
| 310 | LCK | Tyrosine-protein kinase LCK | ChEMBL | PharmMapper |  |  |
| 311 | LCN2 | Neutrophil gelatinase-associated lipocalin | PharmMapper |  |  |  |
| 312 | LDHB | L-lactate dehydrogenase B chain | PharmMapper |  |  |  |
| 313 | LGALS1 | Galectin-1 | SwissTargetPrediction |  |  |  |
| 314 | LGALS2 | Galectin-2 | PharmMapper |  |  |  |
| 315 | LGALS3 | Galectin-3 | PharmMapper |  |  |  |
| 316 | LGALS4 | Galectin-4 | SwissTargetPrediction |  |  |  |
| 317 | LGALS7 | Galectin-7 | SwissTargetPrediction | PharmMapper |  |  |
| 318 | LGALS8 | Galectin-8 | SwissTargetPrediction |  |  |  |
| 319 | LGMN | Legumain | ChEMBL |  |  |  |
| 320 | LSS | Lanosterol synthase | PharmMapper |  |  |  |
| 321 | LTA4H | Leukotriene A4 hydrolase | ChEMBL |  |  |  |
| 322 | LYZ | Lysozyme C | PharmMapper |  |  |  |
| 323 | MAN1B1 | Endoplasmic reticulum mannosyl-oligosaccharide 1,2-alpha-mannosidase | PharmMapper |  |  |  |
| 324 | MAOA | Monoamine oxidase A | ChEMBL |  |  |  |
| 325 | MAOB | Amine oxidase [flavin-containing] B | PharmMapper |  |  |  |
| 326 | MAP2 | Microtubule-associated protein 2 | ChEMBL |  |  |  |
| 327 | MAP2K1 | Dual specificity mitogen-activated protein kinase kinase 1 | SwissTargetPrediction |  |  |  |
| 328 | MAP3K14 | Mitogen-activated protein kinase kinase kinase 14 | ChEMBL |  |  |  |
| 329 | MAP3K9 | Mitogen-activated protein kinase kinase kinase 9 | SwissTargetPrediction |  |  |  |
| 330 | MAPK1 | MAP kinase ERK2 | ChEMBL | PharmMapper |  |  |
| 331 | MAPK10 | Mitogen-activated protein kinase 10 | PharmMapper |  |  |  |
| 332 | MAPK14 | MAP kinase p38 alpha | SwissTargetPrediction | ChEMBL | PharmMapper |  |
| 333 | MAPK3 | MAP kinase ERK1 | ChEMBL |  |  |  |
| 334 | MAPK8 | Mitogen-activated protein kinase 8 | PharmMapper |  |  |  |
| 335 | MAPKAPK2 | MAP kinase-activated protein kinase 2 | ChEMBL | PharmMapper |  |  |
| 336 | MARS | Methionyl-tRNA synthetase | SwissTargetPrediction |  |  |  |
| 337 | MC3R | Melanocortin receptor 3 | ChEMBL |  |  |  |
| 338 | MC4R | Melanocortin receptor 4 | ChEMBL |  |  |  |
| 339 | MC5R | Melanocortin receptor 5 | ChEMBL |  |  |  |
| 340 | MCL1 | Induced myeloid leukemia cell differentiation protein Mcl-1 | ChEMBL |  |  |  |
| 341 | MDM2 | p53-binding protein Mdm-2 | ChEMBL | PharmMapper |  |  |
| 342 | ME2 | NAD-dependent malic enzyme, mitochondrial | PharmMapper |  |  |  |
| 343 | MET | Hepatocyte growth factor receptor | ChEMBL | PharmMapper |  |  |
| 344 | METAP1 | Methionine aminopeptidase 1 | PharmMapper |  |  |  |
| 345 | METAP2 | Methionine aminopeptidase 2 | ChEMBL |  |  |  |
| 346 | METAP2 | Methionine aminopeptidase 2 | PharmMapper |  |  |  |
| 347 | MGLL | Monoglyceride lipase | PUBCHEM | ChemBL |  |  |
| 348 | MGMT | 6-O-methylguanine-DNA methyltransferase | SwissTargetPrediction |  |  |  |
| 349 | MIF | Macrophage migration inhibitory factor | PharmMapper |  |  |  |
| 350 | MKNK1 | MAP kinase-interacting serine/threonine-protein kinase MNK1 | ChEMBL |  |  |  |
| 351 | MME | Neprilysin | SwissTargetPrediction | ChEMBL |  |  |
| 352 | MMP1 | Matrix metalloproteinase 1 | SwissTargetPrediction | ChEMBL |  |  |
| 353 | MMP12 | Matrix metalloproteinase-12 | SwissTargetPrediction | PharmMapper |  |  |
| 354 | MMP13 | Matrix metalloproteinase 13 | SwissTargetPrediction | PharmMapper |  |  |
| 355 | MMP2 | Matrix metalloproteinase-2 | SwissTargetPrediction | ChEMBL | PharmMapper |  |
| 356 | MMP3 | Stromelysin-1 | PharmMapper |  |  |  |
| 357 | MMP7 | Matrix metalloproteinase 7 | SwissTargetPrediction | PharmMapper |  |  |
| 358 | MMP8 | Matrix metalloproteinase 8 | SwissTargetPrediction | PharmMapper |  |  |
| 359 | MMP9 | Matrix metalloproteinase 9 | SwissTargetPrediction | ChEMBL | PharmMapper |  |
| 360 | MTAP | S-methyl-5-thioadenosine phosphorylase | PharmMapper |  |  |  |
| 361 | MTHFD1 | C-1-tetrahydrofolate synthase, cytoplasmic | PharmMapper |  |  |  |
| 362 | MTOR | Serine/threonine-protein kinase mTOR | SwissTargetPrediction |  |  |  |
| 363 | NAAA | N-acylsphingosine-amidohydrolase | ChEMBL |  |  |  |
| 364 | NADK | NAD kinase | SwissTargetPrediction |  |  |  |
| 365 | NAMPT | Nicotinamide phosphoribosyltransferase | ChEMBL |  |  |  |
| 366 | NCS1 | Neuronal calcium sensor 1 | PharmMapper |  |  |  |
| 367 | NMNAT1 | Nicotinamide mononucleotide adenylyltransferase 1 | PharmMapper |  |  |  |
| 368 | NMNAT3 | Nicotinamide mononucleotide adenylyltransferase 3 | PharmMapper |  |  |  |
| 369 | NOS2 | Nitric oxide synthase, inducible | PharmMapper |  |  |  |
| 370 | NOS3 | Nitric oxide synthase, endothelial | PharmMapper |  |  |  |
| 371 | NOX4 | NADPH oxidase 4 | ChEMBL |  |  |  |
| 372 | NPR3 | Atrial natriuretic peptide clearance receptor | PharmMapper |  |  |  |
| 373 | NPY1R | Neuropeptide Y receptor type 1 | ChEMBL |  |  |  |
| 374 | NPY2R | Neuropeptide Y receptor type 2 | ChEMBL |  |  |  |
| 375 | NQO1 | Quinone reductase 1) | ChEMBL | PharmMapper |  |  |
| 376 | NQO2 | Quinone reductase 2 | ChEMBL | PharmMapper |  |  |
| 377 | NR1H2 | Oxysterols receptor LXR-beta | PharmMapper |  |  |  |
| 378 | NR1H3 | Oxysterols receptor LXR-alpha | PharmMapper |  |  |  |
| 379 | NR1H4 | Bile acid receptor | PharmMapper |  |  |  |
| 380 | NR1I3 | Nuclear receptor subfamily 1 group I member 3 | PharmMapper |  |  |  |
| 381 | NR3C1 | Glucocorticoid receptor | ChEMBL |  |  |  |
| 382 | NR3C2 | Mineralocorticoid receptor | PharmMapper |  |  |  |
| 383 | NTSR1 | Neurotensin receptor 1 | ChEMBL |  |  |  |
| 384 | OPRD1 | Delta opioid receptor | ChEMBL |  |  |  |
| 385 | OPRK1 | Kappa opioid receptor | ChEMBL |  |  |  |
| 386 | OPRM1 | Mu opioid receptor | ChEMBL |  |  |  |
| 387 | OTC | Ornithine carbamoyltransferase, mitochondrial | PharmMapper |  |  |  |
| 388 | P2RY12 | Purinergic receptor P2Y12 (by homology) | SwissTargetPrediction |  |  |  |
| 389 | PADI4 | Protein-arginine deiminase type-4 | PharmMapper |  |  |  |
| 390 | PAH | Phenylalanine-4-hydroxylase | PharmMapper |  |  |  |
| 391 | PAK6 | Serine/threonine-protein kinase PAK 6 | PharmMapper |  |  |  |
| 392 | PAPSS1 | Bifunctional 3-phosphoadenosine 5-phosphosulfate synthetase 1 | PharmMapper |  |  |  |
| 393 | PARP1 | Poly [ADP-ribose] polymerase-1 | SwissTargetPrediction | ChEMBL | PharmMapper |  |
| 394 | PCK1 | Phosphoenolpyruvate carboxykinase, cytosolic [GTP] | PharmMapper |  |  |  |
| 395 | PDE2A | Phosphodiesterase 2A | ChEMBL |  |  |  |
| 396 | PDE3B | cGMP-inhibited 3,5-cyclic phosphodiesterase B | PharmMapper |  |  |  |
| 397 | PDE4A | Phosphodiesterase 4A | ChEMBL |  |  |  |
| 398 | PDE4B | Phosphodiesterase 4B | ChEMBL | PharmMapper |  |  |
| 399 | PDE4D | Phosphodiesterase 4D | ChEMBL | PharmMapper |  |  |
| 400 | PDE5A | Phosphodiesterase 5A | ChEMBL | PharmMapper |  |  |
| 401 | PDE7A | Phosphodiesterase 7A | ChEMBL |  |  |  |
| 402 | PDE8B | Phosphodiesterase 8B | ChEMBL |  |  |  |
| 403 | PDE9A | Phosphodiesterase 9A | ChEMBL |  |  |  |
| 404 | PDHB | Pyruvate dehydrogenase E1 component subunit beta, mitochondrial | PharmMapper |  |  |  |
| 405 | PDK2 | [Pyruvate dehydrogenase [lipoamide]] kinase isozyme 2, mitochondrial | PharmMapper |  |  |  |
| 406 | PDPK1 | 3-phosphoinositide-dependent protein kinase 1 | PharmMapper |  |  |  |
| 407 | PGF | Placenta growth factor | SwissTargetPrediction | PharmMapper |  |  |
| 408 | PGR | Progesterone receptor | ChEMBL | PharmMapper |  |  |
| 409 | PIK3CD | PI3-kinase p110-delta subunit | ChEMBL |  |  |  |
| 410 | PIK3CG | PI3-kinase p110-gamma subunit | SwissTargetPrediction | ChEMBL | PharmMapper |  |
| 411 | PIM1 | Serine/threonine-protein kinase PIM1 | ChEMBL | PharmMapper |  |  |
| 412 | PIM2 | Serine/threonine-protein kinase PIM2 | ChEMBL |  |  |  |
| 413 | PIN1 | Peptidyl-prolyl cis-trans isomerase NIMA-interacting 1 | ChEMBL |  |  |  |
| 414 | PLA2G10 | Group X secretory phospholipase A2 | ChEMBL | PharmMapper |  |  |
| 415 | PLA2G2A | Phospholipase A2 group IIA | ChEMBL | PharmMapper |  |  |
| 416 | PLA2G4A | Cytosolic phospholipase A2 | ChEMBL |  |  |  |
| 417 | PLAU | Urokinase-type plasminogen activator | ChEMBL | PharmMapper |  |  |
| 418 | PLG | Plasminogen (by homology) | SwissTargetPrediction |  |  |  |
| 419 | PLK1 | Serine/threonine-protein kinase PLK1 | ChEMBL | PharmMapper |  |  |
| 420 | PLK4 | Serine/threonine-protein kinase PLK4 | ChEMBL |  |  |  |
| 421 | PNMT | Phenylethanolamine N-methyltransferase | PharmMapper |  |  |  |
| 422 | PNP | Purine nucleoside phosphorylase | PharmMapper |  |  |  |
| 423 | PNPO | Pyridoxine-5-phosphate oxidase | PharmMapper |  |  |  |
| 424 | PPARA | Peroxisome proliferator-activated receptor alpha | PharmMapper |  |  |  |
| 425 | PPARD | Peroxisome proliferator-activated receptor delta | ChEMBL | PharmMapper |  |  |
| 426 | PPARG | Peroxisome proliferator-activated receptor gamma | ChEMBL | PharmMapper |  |  |
| 427 | PPIA | Cyclophilin A | ChEMBL | PharmMapper |  |  |
| 428 | PPP5C | Serine/threonine-protein phosphatase 5 | PharmMapper |  |  |  |
| 429 | PREP | Prolyl endopeptidase | ChEMBL |  |  |  |
| 430 | PRKACA | cAMP-dependent protein kinase catalytic subunit alpha | PharmMapper |  |  |  |
| 431 | PRKCA | Protein kinase C alpha type | SwissTargetPrediction | BindingDB | ChEMBL |  |
| 432 | PRKCQ | Protein kinase C theta type | PharmMapper |  |  |  |
| 433 | PRKDC | DNA-dependent protein kinase | ChEMBL |  |  |  |
| 434 | PRSS1 | Trypsin I (by homology) | SwissTargetPrediction |  |  |  |
| 435 | PRSS3 | Trypsin III (by homology) | SwissTargetPrediction |  |  |  |
| 436 | PSMB5 | Proteasome Macropain subunit MB1 | ChEMBL |  |  |  |
| 437 | PTGER2 | Prostanoid EP2 receptor | ChEMBL |  |  |  |
| 438 | PTGER3 | Prostanoid EP3 receptor | ChEMBL |  |  |  |
| 439 | PTGER4 | Prostanoid EP4 receptor | ChEMBL |  |  |  |
| 440 | PTGES | Prostaglandin E synthase | ChEMBL |  |  |  |
| 441 | PTGS2 | Prostaglandin G/H synthase 2 | PubChem |  |  |  |
| 442 | PTK2 | Focal adhesion kinase 1 | ChEMBL |  |  |  |
| 443 | PTPN1 | Protein-tyrosine phosphatase 1B | ChEMBL | PharmMapper |  |  |
| 444 | PTPN2 | T-cell protein-tyrosine phosphatase | ChEMBL |  |  |  |
| 445 | PTPRA | Receptor-type tyrosine-protein phosphatase alpha | SwissTargetPrediction |  |  |  |
| 446 | PYGL | Glycogen phosphorylase, liver form | PharmMapper |  |  |  |
| 447 | QPCT | Glutaminyl-peptide cyclotransferase | PharmMapper |  |  |  |
| 448 | RAB11A | Ras-related protein Rab-11A | PharmMapper |  |  |  |
| 449 | RAB5A | Ras-related protein Rab-5A | PharmMapper |  |  |  |
| 450 | RAC2 | Ras-related C3 botulinum toxin substrate 2 | PharmMapper |  |  |  |
| 451 | RARA | Retinoic acid receptor alpha | PharmMapper |  |  |  |
| 452 | RBP4 | Retinol-binding protein 4 | PharmMapper |  |  |  |
| 453 | RCOR1 | LSD1/CoREST complex | ChEMBL |  |  |  |
| 454 | REG1A | Lithostathine-1-alpha | PharmMapper |  |  |  |
| 455 | REN | Renin | ChEMBL | PharmMapper |  |  |
| 456 | RHEB | GTP-binding protein Rheb | PharmMapper |  |  |  |
| 457 | RHOA | Transforming protein RhoA | PharmMapper |  |  |  |
| 458 | RNASE3 | Eosinophil cationic protein | PharmMapper |  |  |  |
| 459 | RNASE4 | Ribonuclease 4 | PharmMapper |  |  |  |
| 460 | RORA | Nuclear receptor ROR-alpha | PharmMapper |  |  |  |
| 461 | ROS1 | Proto-oncogene tyrosine-protein kinase ROS | ChEMBL |  |  |  |
| 462 | RTN4R | Reticulon-4 receptor | PharmMapper |  |  |  |
| 463 | RXRA | Retinoic acid receptor RXR-alpha | PharmMapper |  |  |  |
| 464 | S100A9 | Protein S100-A9 | PharmMapper |  |  |  |
| 465 | S1PR2 | Sphingosine 1-phosphate receptor Edg-5 | ChEMBL |  |  |  |
| 466 | S1PR3 | Sphingosine 1-phosphate receptor Edg-3 | ChEMBL |  |  |  |
| 467 | SCN3A | Sodium channel protein type III alpha subunit | ChEMBL |  |  |  |
| 468 | SDS | L-serine dehydratase | PharmMapper |  |  |  |
| 469 | SEC14L2 | SEC14-like protein 2 | PharmMapper |  |  |  |
| 470 | SELP | P-selectin | PharmMapper |  |  |  |
| 471 | SERPINA1 | Alpha-1-antitrypsin | PharmMapper |  |  |  |
| 472 | SHBG | Sex hormone-binding globulin | PharmMapper |  |  |  |
| 473 | SHMT1 | Serine hydroxymethyltransferase, cytosolic | PharmMapper |  |  |  |
| 474 | SIGMAR1 | Sigma opioid receptor | ChEMBL |  |  |  |
| 475 | SIRT2 | NAD-dependent deacetylase sirtuin 2 | ChEMBL |  |  |  |
| 476 | SLC22A12 | Solute carrier family 22 member 12 | ChEMBL |  |  |  |
| 477 | SLC28A2 | Sodium/nucleoside cotransporter 2 | SwissTargetPrediction |  |  |  |
| 478 | SLC29A1 | Equilibrative nucleoside transporter 1 | SwissTargetPrediction |  |  |  |
| 479 | SLC5A2 | Sodium/glucose cotransporter 2 | ChEMBL |  |  |  |
| 480 | SLC6A2 | Norepinephrine transporter | SwissTargetPrediction | ChEMBL |  |  |
| 481 | SLC6A3 | Dopamine transporter | ChEMBL |  |  |  |
| 482 | SLC6A4 | Serotonin transporter | ChEMBL |  |  |  |
| 483 | SOAT2 | Acyl coenzyme A:cholesterol acyltransferase 2 | ChEMBL |  |  |  |
| 484 | SOD2 | Superoxide dismutase [Mn], mitochondrial | PharmMapper |  |  |  |
| 485 | SORD | Sorbitol dehydrogenase | PharmMapper |  |  |  |
| 486 | SPARC | SPARC | PharmMapper |  |  |  |
| 487 | SPHK2 | Sphingosine kinase 2 | ChEMBL |  |  |  |
| 488 | SRC | Tyrosine-protein kinase SRC | SwissTargetPrediction | PharmMapper |  |  |
| 489 | SRD5A2 | Steroid 5-alpha-reductase 2 | ChEMBL |  |  |  |
| 490 | SRM | Spermidine synthase | PharmMapper |  |  |  |
| 491 | SSE1 | Heat shock protein homolog SSE1 | PharmMapper |  |  |  |
| 492 | SSTR2 | Somatostatin receptor 2 | ChEMBL |  |  |  |
| 493 | SSTR5 | Somatostatin receptor 5 | ChEMBL |  |  |  |
| 494 | ST14 | Suppressor of tumorigenicity protein 14 | PharmMapper |  |  |  |
| 495 | SULT1E1 | Estrogen sulfotransferase | PharmMapper |  |  |  |
| 496 | SULT2A1 | Bile salt sulfotransferase | PharmMapper |  |  |  |
| 497 | SULT2B1 | Sulfotransferase family cytosolic 2B member 1 | PharmMapper |  |  |  |
| 498 | SYK | Tyrosine-protein kinase SYK | PharmMapper |  |  |  |
| 499 | TACR1 | Neurokinin 1 receptor | ChEMBL |  |  |  |
| 500 | TACR2 | Neurokinin 2 receptor | SwissTargetPrediction | ChEMBL |  |  |
| 501 | TARS | Threonyl-tRNA synthetase | SwissTargetPrediction |  |  |  |
| 502 | TBXAS1 | Thromboxane-A synthase | ChEMBL |  |  |  |
| 503 | TGFBR1 | TGF-beta receptor type I | ChEMBL | PharmMapper |  |  |
| 504 | TGM3 | Protein-glutamine gamma-glutamyltransferase E | PharmMapper |  |  |  |
| 505 | THRA | Thyroid hormone receptor alpha | ChEMBL | PharmMapper |  |  |
| 506 | THRB | Thyroid hormone receptor beta-1 | ChEMBL |  |  |  |
| 507 | TLR7 | Toll-like receptor 7 | ChEMBL |  |  |  |
| 508 | TMIGD3 | Transmembrane domain-containing protein TMIGD3 | ChEMBL |  |  |  |
| 509 | TMPRSS6 | Transmembrane protease serine 6 | ChEMBL |  |  |  |
| 510 | TNF | Tumor necrosis factor | PubChem | SwissTargetPrediction | SwissTargetPrediction |  |
| 511 | TNK2 | Activated CDC42 kinase 1 | PharmMapper |  |  |  |
| 512 | TNNC1 | Troponin C, slow skeletal and cardiac muscles | PharmMapper |  |  |  |
| 513 | TOP1 | DNA topoisomerase I | ChEMBL |  |  |  |
| 514 | TOP2A | DNA topoisomerase II alpha | SwissTargetPrediction |  |  |  |
| 515 | TPH1 | Tryptophan 5-hydroxylase 1 | ChEMBL | PharmMapper |  |  |
| 516 | TPI1 | Triosephosphate isomerase | PharmMapper |  |  |  |
| 517 | TPSAB1 | Tryptase beta-1 | ChEMBL |  |  |  |
| 518 | TRAPPC3 | Trafficking protein particle complex subunit 3 | PharmMapper |  |  |  |
| 519 | TREM1 | Triggering receptor expressed on myeloid cells 1 | PharmMapper |  |  |  |
| 520 | TRPV1 | Vanilloid receptor | ChEMBL |  |  |  |
| 521 | TTR | Transthyretin | PharmMapper |  |  |  |
| 522 | TYMP | Thymidine phosphorylase | SwissTargetPrediction | PharmMapper |  |  |
| 523 | TYMS | Thymidylate synthase | PharmMapper |  |  |  |
| 524 | UCK2 | Uridine-cytidine kinase 2 | PharmMapper |  |  |  |
| 525 | UMPS | Uridine 5-monophosphate synthase | PharmMapper |  |  |  |
| 526 | VARS | Valyl-tRNA synthetase 2 | SwissTargetPrediction |  |  |  |
| 527 | VDR | Vitamin D receptor | ChEMBL |  |  |  |
| 528 | WAS | Wiskott-Aldrich syndrome protein | PharmMapper |  |  |  |
| 529 | WDR5 | WD repeat-containing protein 5 | ChEMBL |  |  |  |
| 530 | XDH | Xanthine dehydrogenase | ChEMBL |  |  |  |
| 531 | XIAP | Inhibitor of apoptosis protein 3 | ChEMBL | PharmMapper |  |  |
| 532 | YARS | Tyrosyl-tRNA synthetase | SwissTargetPrediction |  |  |  |
| 533 | YARS1 | Tyrosyl-tRNA synthetase, cytoplasmic | PharmMapper |  |  |  |
| 534 | ZAP70 | Tyrosine-protein kinase ZAP-70 | PharmMapper |  |  |  |
