# Supplementary figures and images for: Cellular mechanism of action of forsythiaside for the treatment of diabetic kidney disease
Source: Front Pharmacol. 2023 Jan 13;13:1096536. doi: 10.3389/fphar.2022.1096536 (PMC9880420; doi:10.3389/fphar.2022.1096536)

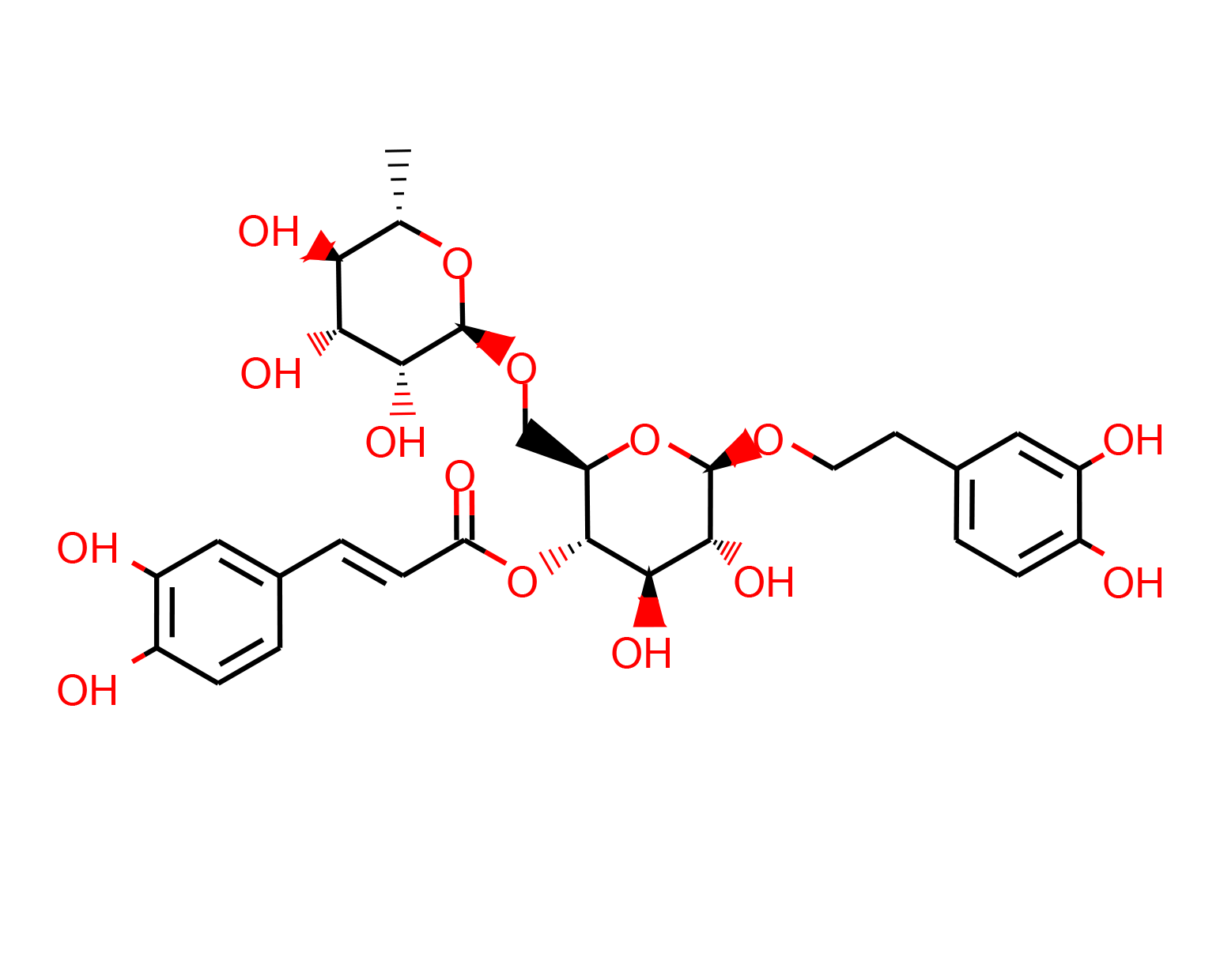

Supplement: Supplementary file 2 [file Image1.tif]
