# Supplementary material for: Cellular mechanism of action of forsythiaside for the treatment of diabetic kidney disease
Source: Front Pharmacol. 2023 Jan 13;13:1096536. doi: 10.3389/fphar.2022.1096536 (PMC9880420; doi:10.3389/fphar.2022.1096536)
Supplement: Supplementary file 3 [file Table2.DOCX]

**Table S2**. DKD related targets.

| **Number** | **Gene name** | **Protein name** | **Database 1** | **Database 2** | **Database 3** | **Database 4** |
| --- | --- | --- | --- | --- | --- | --- |
| 1 | AARS1 | Alanyl-TRNA Synthetase 1 | GeneCards |  |  |  |
| 2 | ABCA1 | ATP Binding Cassette Subfamily A Member 1 | GeneCards | DisGeNET |  |  |
| 3 | ABCA4 | ATP Binding Cassette Subfamily A Member 4 | GeneCards | OMIM |  |  |
| 4 | ABCB1 | ATP Binding Cassette Subfamily B Member 1 | GeneCards | OMIM |  |  |
| 5 | ABCB11 | Bile salt export pump | DrugBank |  |  |  |
| 6 | ABCB4 | Phosphatidylcholine translocator ABCB4 | OMIM |  |  |  |
| 7 | ABCC11 | ATP binding cassette subfamily C member 11 | DisGeNET |  |  |  |
| 8 | ABCC6 | ATP binding cassette subfamily C member 6 | DisGeNET |  |  |  |
| 9 | ABCC8 | ATP Binding Cassette Subfamily C Member 8 | GeneCards | DisGeNET | OMIM |  |
| 10 | ABCG2 | ATP-binding cassette sub-family G member 2 | DrugBank |  |  |  |
| 11 | ABCG8 | ATP-binding cassette sub-family G member 8 | OMIM |  |  |  |
| 12 | ACACB | Acetyl-CoA carboxylase beta | DisGeNET |  |  |  |
| 13 | ACCS | 1-aminocyclopropane-1-carboxylate synthase homolog (inactive) | DisGeNET |  |  |  |
| 14 | ACE | Angiotensin I Converting Enzyme | GeneCards | DisGeNET | DrugBank | OMIM |
| 15 | ACE2 | Angiotensin I converting enzyme 2 | DisGeNET |  |  |  |
| 16 | ACKR2 | Atypical chemokine receptor 2 | DisGeNET |  |  |  |
| 17 | ACOX1 | Acyl-CoA oxidase 1 | DisGeNET |  |  |  |
| 18 | ACR | Acrosin | DisGeNET |  |  |  |
| 19 | ACSS2 | Acyl-CoA synthetase short chain family member 2 | DisGeNET |  |  |  |
| 20 | ACTA2 | Actin alpha 2, smooth muscle | DisGeNET |  |  |  |
| 21 | ACTB | Actin beta | DisGeNET |  |  |  |
| 22 | ACTG2 | Actin gamma 2, smooth muscle | DisGeNET |  |  |  |
| 23 | ACTN4 | actinin alpha 4 | GeneCards | DisGeNET |  |  |
| 24 | ACVR1 | Activin A receptor type 1 | DisGeNET |  |  |  |
| 25 | ACVRL1 | Activin A receptor like type 1 | DisGeNET |  |  |  |
| 26 | ADAM10 | ADAM metallopeptidase domain 10 | DisGeNET |  |  |  |
| 27 | ADAM17 | ADAM metallopeptidase domain 17 | DisGeNET | OMIM |  |  |
| 28 | ADAMTS13 | ADAM metallopeptidase with thrombospondin type 1 motif 13 | DisGeNET |  |  |  |
| 29 | ADCY8 | Adenylate cyclase 8 | DisGeNET |  |  |  |
| 30 | ADD1 | Adducin 1 | DisGeNET |  |  |  |
| 31 | ADD2 | Adducin 2 | DisGeNET |  |  |  |
| 32 | ADGRG1 | Adhesion G protein-coupled receptor G1 | DisGeNET |  |  |  |
| 33 | ADH1B | Alcohol dehydrogenase 1B (class I), beta polypeptide | DisGeNET |  |  |  |
| 34 | ADH1C | Alcohol dehydrogenase 1C | OMIM |  |  |  |
| 35 | ADIPOQ | Adiponectin, C1Q and collagen domain containing | DisGeNET | GeneCards |  |  |
| 36 | ADIPOR1 | Adiponectin receptor 1 | DisGeNET |  |  |  |
| 37 | ADIPOR2 | Adiponectin receptor 2 | DisGeNET |  |  |  |
| 38 | ADM | Adrenomedullin | DisGeNET |  |  |  |
| 39 | ADORA1 | Adenosine A1 receptor | DisGeNET |  |  |  |
| 40 | ADRB2 | Adrenoceptor Beta 2 | GeneCards |  |  |  |
| 41 | AFM | Afamin | DisGeNET |  |  |  |
| 42 | AGER | Advanced Glycosylation End-Product Specific Receptor | GeneCards | DisGeNET |  |  |
| 43 | AGL | Amylo-Alpha-1, 6-Glucosidase, 4-Alpha-Glucanotransferase | GeneCards | OMIM |  |  |
| 44 | AGPAT2 | 1-Acylglycerol-3-Phosphate O-Acyltransferase 2 | GeneCards |  |  |  |
| 45 | AGT | Angiotensinogen | GeneCards | DisGeNET |  |  |
| 46 | AGTR1 | Angiotensin II Receptor Type 1 | GeneCards | DisGeNET | DrugBank |  |
| 47 | AGTR2 | Angiotensin II receptor type 2 | DisGeNET |  |  |  |
| 48 | AHI1 | Abelson Helper Integration Site 1 | GeneCards |  |  |  |
| 49 | AHSA1 | Activator of HSP90 ATPase activity 1 | DisGeNET |  |  |  |
| 50 | AHSG | Alpha 2-HS glycoprotein | DisGeNET |  |  |  |
| 51 | AIFM1 | Apoptosis inducing factor mitochondria associated 1 | DisGeNET |  |  |  |
| 52 | AIMP2 | Aminoacyl tRNA synthetase complex interacting multifunctional protein 2 | DisGeNET |  |  |  |
| 53 | AIRE | Autoimmune Regulator | GeneCards |  |  |  |
| 54 | AKAP1 | A-kinase anchoring protein 1 | DisGeNET |  |  |  |
| 55 | AKR1A1 | Aldo-keto reductase family 1 member A1 | DisGeNET |  |  |  |
| 56 | AKR1B1 | Aldo-Keto Reductase Family 1 Member B | GeneCards | DisGeNET |  |  |
| 57 | AKT1 | AKT Serine/Threonine Kinase 1 | GeneCards | DisGeNET |  |  |
| 58 | AKT2 | AKT Serine/Threonine Kinase 2 | GeneCards | OMIM |  |  |
| 59 | ALB | Albumin | GeneCards | DisGeNET | DrugBank |  |
| 60 | ALCAM | Activated leukocyte cell adhesion molecule | DisGeNET |  |  |  |
| 61 | ALDH2 | Aldehyde dehydrogenase 2 family member | DisGeNET |  |  |  |
| 62 | ALG9 | ALG9 Alpha-1,2-Mannosyltransferase | GeneCards |  |  |  |
| 63 | ALKBH1 | AlkB homolog 1, histone H2A dioxygenase | DisGeNET |  |  |  |
| 64 | ALMS1 | ALMS1 Centrosome And Basal Body Associated Protein | GeneCards |  |  |  |
| 65 | ALOX12 | Arachidonate 12-lipoxygenase, 12S type | DisGeNET |  |  |  |
| 66 | ALPK1 | Alpha kinase 1 | DisGeNET |  |  |  |
| 67 | ALPL | Alkaline Phosphatase, Biomineralization Associated | GeneCards | OMIM |  |  |
| 68 | AMT | Aminomethyltransferase | DisGeNET |  |  |  |
| 69 | ANG | Angiogenin | DisGeNET |  |  |  |
| 70 | ANGPT1 | Angiopoietin 1 | DisGeNET |  |  |  |
| 71 | ANGPT2 | Angiopoietin 2 | DisGeNET |  |  |  |
| 72 | ANGPTL2 | Angiopoietin like 2 | DisGeNET |  |  |  |
| 73 | ANGPTL4 | Angiopoietin like 4 | DisGeNET |  |  |  |
| 74 | ANKRD1 | Ankyrin repeat domain 1 | DisGeNET |  |  |  |
| 75 | ANKS6 | Ankyrin Repeat And Sterile Alpha Motif Domain Containing 6 | GeneCards |  |  |  |
| 76 | ANO1 | Anoctamin 1 | DisGeNET |  |  |  |
| 77 | AOC3 | Membrane copper amine oxidase | TTD | DisGeNET |  |  |
| 78 | APC | APC Regulator Of WNT Signaling Pathway | GeneCards | OMIM |  |  |
| 79 | APEX1 | Apurinic/apyrimidinic endodeoxyribonuclease 1 | DisGeNET |  |  |  |
| 80 | APLN | Apelin | DisGeNET |  |  |  |
| 81 | APLNR | Apelin receptor | DisGeNET |  |  |  |
| 82 | APOA1 | Apolipoprotein A1 | GeneCards | DisGeNET |  |  |
| 83 | APOA4 | Apolipoprotein A4 | DisGeNET |  |  |  |
| 84 | APOA5 | Apolipoprotein A5 | DisGeNET |  |  |  |
| 85 | APOB | Apolipoprotein B | GeneCards | DisGeNET |  |  |
| 86 | APOC1 | Apolipoprotein C1 | DisGeNET |  |  |  |
| 87 | APOC3 | Apolipoprotein C3 | GeneCards | DisGeNET |  |  |
| 88 | APOE | Apolipoprotein E | GeneCards | DisGeNET |  |  |
| 89 | APOH | Apolipoprotein H | DisGeNET |  |  |  |
| 90 | APOL1 | Apolipoprotein L1 | GeneCards | DisGeNET |  |  |
| 91 | APOL3 | Apolipoprotein L3 | DisGeNET |  |  |  |
| 92 | APOM | Apolipoprotein M | DisGeNET |  |  |  |
| 93 | APP | Amyloid Beta Precursor Protein | GeneCards |  |  |  |
| 94 | APPL1 | Adaptor Protein, Phosphotyrosine Interacting With PH Domain And Leucine Zipper 1 | GeneCards | OMIM |  |  |
| 95 | APRT | Adenine phosphoribosyltransferase | DisGeNET |  |  |  |
| 96 | AQP1 | Aquaporin 1 (Colton blood group) | DisGeNET |  |  |  |
| 97 | AQP11 | Aquaporin 11 | DisGeNET |  |  |  |
| 98 | AQP2 | Aquaporin 2 | GeneCards | DisGeNET | OMIM |  |
| 99 | AQP5 | Aquaporin 5 | DisGeNET |  |  |  |
| 100 | AQP5-AS1 | AQP5 And AQP2 Antisense RNA 2 | GeneCards |  |  |  |
| 101 | AR | Androgen Receptor | GeneCards |  |  |  |
| 102 | ARAP1 | ArfGAP with RhoGAP domain, ankyrin repeat and PH domain 1 | DisGeNET |  |  |  |
| 103 | ARAP1-AS1 | ARAP1 antisense RNA 1 | DisGeNET |  |  |  |
| 104 | ARAP1-AS2 | ARAP1 antisense RNA 2 | DisGeNET |  |  |  |
| 105 | ARF6 | ADP ribosylation factor 6 | DisGeNET |  |  |  |
| 106 | ARG1 | Arginase 1 | DisGeNET |  |  |  |
| 107 | ARG2 | arginase 2 | DisGeNET |  |  |  |
| 108 | ARID2 | AT-rich interaction domain 2 | DisGeNET |  |  |  |
| 109 | ARPC1B | Actin-related protein 2/3 complex subunit 1B | OMIM |  |  |  |
| 110 | ARRB1 | Arrestin beta 1 | DisGeNET |  |  |  |
| 111 | ARRB2 | Arrestin beta 2 | DisGeNET |  |  |  |
| 112 | ATF6 | Activating transcription factor 6 | DisGeNET |  |  |  |
| 113 | ATG16L1 | Autophagy-related protein 16-1 | OMIM |  |  |  |
| 114 | ATM | ATM Serine/Threonine Kinase | GeneCards | DisGeNET |  |  |
| 115 | ATN1 | Atrophin 1 | DisGeNET |  |  |  |
| 116 | ATP1A1 | Sodium/potassium-transporting ATPase subunit alpha-1 | OMIM |  |  |  |
| 117 | ATP2A2 | ATPase Sarcoplasmic/Endoplasmic Reticulum Ca2+ Transporting 2 | GeneCards | DisGeNET |  |  |
| 118 | ATP2C1 | Calcium-transporting ATPase type 2C member 1 | OMIM |  |  |  |
| 119 | ATP5F1B | ATP synthase F1 subunit beta | DisGeNET |  |  |  |
| 120 | ATP6AP2 | ATPase H+ transporting accessory protein 2 | DisGeNET |  |  |  |
| 121 | ATP7B | ATPase Copper Transporting Beta | GeneCards |  |  |  |
| 122 | ATXN2 | Ataxin 2 | GeneCards |  |  |  |
| 123 | AVP | Arginine Vasopressin | GeneCards | DisGeNET | OMIM |  |
| 124 | AVPR2 | Arginine Vasopressin Receptor 2 | GeneCards | DisGeNET | OMIM |  |
| 125 | AXIN2 | Axin 2 | DisGeNET |  |  |  |
| 126 | AXL | AXL receptor tyrosine kinase | DisGeNET |  |  |  |
| 127 | AZIN1 | Antizyme inhibitor 1 | DisGeNET |  |  |  |
| 128 | AZU1 | Azurocidin 1 | DisGeNET |  |  |  |
| 129 | B2M | Beta-2-Microglobulin | GeneCards | DisGeNET |  |  |
| 130 | B3GALNT1 | Beta-1,3-N-acetylgalactosaminyltransferase 1 (globoside blood group) | DisGeNET |  |  |  |
| 131 | BACE | Beta-secretase | TTD |  |  |  |
| 132 | BASP1 | Brain abundant membrane attached signal protein 1 | DisGeNET |  |  |  |
| 133 | BBOX1 | Gamma-butyrobetaine hydroxylase 1 | DisGeNET |  |  |  |
| 134 | BBS1 | Bardet-Biedl Syndrome 1 | GeneCards |  |  |  |
| 135 | BBS10 | Bardet-Biedl Syndrome 10 | GeneCards |  |  |  |
| 136 | BBS12 | Bardet-Biedl Syndrome 12 | GeneCards |  |  |  |
| 137 | BBS2 | Bardet-Biedl Syndrome 2 | GeneCards |  |  |  |
| 138 | BBS4 | Bardet-Biedl Syndrome 4 | GeneCards |  |  |  |
| 139 | BCHE | Cholinesterase | DrugBank |  |  |  |
| 140 | BCKDHB | 2-oxoisovalerate dehydrogenase subunit beta, mitochondrial | OMIM |  |  |  |
| 141 | BCL2 | BCL2 apoptosis regulator | DisGeNET |  |  |  |
| 142 | BCR | BCR activator of RhoGEF and GTPase | DisGeNET |  |  |  |
| 143 | BDKRB1 | B1 bradykinin receptor | DrugBank |  |  |  |
| 144 | BDKRB2 | Bradykinin receptor B2 | DisGeNET |  |  |  |
| 145 | BDNF | Brain Derived Neurotrophic Factor | GeneCards | DisGeNET |  |  |
| 146 | BECN1 | Beclin 1 | DisGeNET |  |  |  |
| 147 | BEST1 | Bestrophin 1 | DisGeNET |  |  |  |
| 148 | BGLAP | Bone Gamma-Carboxyglutamate Protein | GeneCards | DisGeNET |  |  |
| 149 | BGN | Biglycan | DisGeNET |  |  |  |
| 150 | BICC1 | BicC Family RNA Binding Protein 1 | GeneCards |  |  |  |
| 151 | BLK | BLK Proto-Oncogene, Src Family Tyrosine Kinase | GeneCards | OMIM |  |  |
| 152 | BMP2 | Bone Morphogenetic Protein 2 | GeneCards | DisGeNET |  |  |
| 153 | BMP4 | Bone Morphogenetic Protein 4 | GeneCards | DisGeNET |  |  |
| 154 | BMP7 | Bone morphogenetic protein 7 | DisGeNET |  |  |  |
| 155 | BMPR2 | Bone morphogenetic protein receptor type-2 | OMIM |  |  |  |
| 156 | BNIP3 | BCL2 interacting protein 3 | DisGeNET |  |  |  |
| 157 | BRAF | B-Raf Proto-Oncogene, Serine/Threonine Kinase | GeneCards |  |  |  |
| 158 | BRCA1 | BRCA1 DNA Repair Associated | GeneCards |  |  |  |
| 159 | BRCA2 | BRCA2 DNA Repair Associated | GeneCards |  |  |  |
| 160 | BRD2 | Bromodomain containing 2 | DisGeNET |  |  |  |
| 161 | BRD4 | Bromodomain containing 4 | DisGeNET |  |  |  |
| 162 | BSCL2 | BSCL2 Lipid Droplet Biogenesis Associated, Seipin | GeneCards |  |  |  |
| 163 | BSG | Basigin (Ok blood group) | DisGeNET |  |  |  |
| 164 | BSND | Barttin CLCNK Type Accessory Subunit Beta | GeneCards |  |  |  |
| 165 | BTRC | Beta-transducin repeat containing E3 ubiquitin protein ligase | DisGeNET |  |  |  |
| 166 | C12orf43 | Chromosome 12 Open Reading Frame 43 | GeneCards |  |  |  |
| 167 | C3 | Complement C3 | GeneCards | DisGeNET |  |  |
| 168 | C3AR1 | Complement C3a receptor 1 | DisGeNET |  |  |  |
| 169 | C4A | Complement C4A (Rodgers Blood Group) | GeneCards |  |  |  |
| 170 | C5 | Complement C5 | DisGeNET |  |  |  |
| 171 | C5AR1 | Complement C5a receptor 1 | DisGeNET |  |  |  |
| 172 | CADM1 | Cell adhesion molecule 1 | DisGeNET |  |  |  |
| 173 | CALB1 | Calbindin 1 | DisGeNET |  |  |  |
| 174 | CALCRL | Calcitonin receptor like receptor | DisGeNET |  |  |  |
| 175 | CALD1 | Caldesmon 1 | DisGeNET |  |  |  |
| 176 | CAMKK2 | Calcium/calmodulin dependent protein kinase kinase 2 | DisGeNET |  |  |  |
| 177 | CAPN10 | Calpain 10 | GeneCards | DisGeNET | OMIM |  |
| 178 | CARS1 | Cysteinyl-tRNA synthetase 1 | DisGeNET |  |  |  |
| 179 | CASC2 | Cancer susceptibility 2 | DisGeNET |  |  |  |
| 180 | CASP12 | Caspase 12 (gene/pseudogene) | DisGeNET |  |  |  |
| 181 | CASP3 | Caspase 3 | GeneCards | DisGeNET |  |  |
| 182 | CASP7 | Caspase 7 | DisGeNET |  |  |  |
| 183 | CASP8 | Caspase 8 | DisGeNET |  |  |  |
| 184 | CASP9 | Caspase 9 | DisGeNET |  |  |  |
| 185 | CASR | Calcium Sensing Receptor | GeneCards |  |  |  |
| 186 | CAT | Catalase | GeneCards | DisGeNET |  |  |
| 187 | CAV1 | Caveolin 1 | GeneCards | DisGeNET |  |  |
| 188 | CAV3 | Caveolin-3 | OMIM |  |  |  |
| 189 | CC2D2A | Coiled-Coil And C2 Domain Containing 2A | GeneCards |  |  |  |
| 190 | CCHCR1 | Coiled-coil alpha-helical rod protein 1 | DisGeNET |  |  |  |
| 191 | CCL1 | C-C motif chemokine ligand 1 | DisGeNET |  |  |  |
| 192 | CCL2 | C-C Motif Chemokine Ligand 2 | GeneCards | DisGeNET |  |  |
| 193 | CCL20 | C-C motif chemokine ligand 20 | DisGeNET |  |  |  |
| 194 | CCL5 | C-C Motif Chemokine Ligand 5 | GeneCards | DisGeNET |  |  |
| 195 | CCN2 | Cellular Communication Network Factor 2 | GeneCards | DisGeNET |  |  |
| 196 | CCND1 | Cyclin D1 | GeneCards |  |  |  |
| 197 | CCNE1 | Cyclin E1 | DisGeNET |  |  |  |
| 198 | CCNG2 | Cyclin G2 | DisGeNET |  |  |  |
| 199 | CCR1 | C-C motif chemokine receptor 1 | DisGeNET |  |  |  |
| 200 | CCR2 | C-C motif chemokine receptor 2 | TTD | DisGeNET |  |  |
| 201 | CCR5 | C-C Motif Chemokine Receptor 5 | TTD | GeneCards | DisGeNET | OMIM |
| 202 | CCR6 | C-C Motif Chemokine Receptor 6 | GeneCards | DisGeNET |  |  |
| 203 | CCT2 | Chaperonin containing TCP1 subunit 2 | DisGeNET |  |  |  |
| 204 | CD151 | CD151 antigen | OMIM |  |  |  |
| 205 | CD163 | CD163 molecule | DisGeNET |  |  |  |
| 206 | CD2AP | CD2 associated protein | DisGeNET |  |  |  |
| 207 | CD36 | CD36 Molecule | GeneCards | DisGeNET | OMIM |  |
| 208 | CD38 | CD38 molecule | DisGeNET |  |  |  |
| 209 | CD4 | CD4 Molecule | GeneCards |  |  |  |
| 210 | CD40LG | CD40 Ligand | GeneCards | DisGeNET |  |  |
| 211 | CD44 | CD44 molecule (Indian blood group) | DisGeNET |  |  |  |
| 212 | CD59 | CD59 molecule (CD59 blood group) | DisGeNET |  |  |  |
| 213 | CD5L | CD5 molecule like | DisGeNET |  |  |  |
| 214 | CD68 | CD68 molecule | DisGeNET |  |  |  |
| 215 | CD74 | CD74 molecule | DisGeNET |  |  |  |
| 216 | CD80 | CD80 molecule | DisGeNET |  |  |  |
| 217 | CDAN1 | Codanin 1 | DisGeNET |  |  |  |
| 218 | CDC42 | Cell division cycle 42 | DisGeNET |  |  |  |
| 219 | CDH1 | Cadherin 1 | GeneCards |  |  |  |
| 220 | CDH13 | Cadherin 13 | DisGeNET |  |  |  |
| 221 | CDH2 | Cadherin 2 | DisGeNET |  |  |  |
| 222 | CDK5 | Cyclin dependent kinase 5 | DisGeNET |  |  |  |
| 223 | CDKAL1 | CDK5 regulatory subunit associated protein 1 like 1 | DisGeNET |  |  |  |
| 224 | CDKN1A | Cyclin dependent kinase inhibitor 1A | DisGeNET |  |  |  |
| 225 | CDKN1B | Cyclin dependent kinase inhibitor 1B | DisGeNET |  |  |  |
| 226 | CDKN1C | Cyclin Dependent Kinase Inhibitor 1C | GeneCards |  |  |  |
| 227 | CDKN2A | Cyclin Dependent Kinase Inhibitor 2A | GeneCards | DisGeNET |  |  |
| 228 | CDKN2B-AS1 | CDKN2B antisense RNA 1 | DisGeNET |  |  |  |
| 229 | CEBPB | CCAAT enhancer binding protein beta | DisGeNET |  |  |  |
| 230 | CEL | Carboxyl Ester Lipase | GeneCards | OMIM |  |  |
| 231 | CELA2A | Chymotrypsin Like Elastase 2A | GeneCards |  |  |  |
| 232 | CEP290 | Centrosomal Protein 290 | GeneCards |  |  |  |
| 233 | CES1 | Liver carboxylesterase 1 | DrugBank |  |  |  |
| 234 | CETP | Cholesteryl Ester Transfer Protein | GeneCards | DisGeNET |  |  |
| 235 | CFD | Complement factor D | DisGeNET |  |  |  |
| 236 | CFH | Complement Factor H | GeneCards |  |  |  |
| 237 | CFHR5 | Complement Factor H Related 5 | GeneCards | OMIM |  |  |
| 238 | CFI | Complement Factor I | GeneCards |  |  |  |
| 239 | CFL1 | Cofilin 1 | DisGeNET |  |  |  |
| 240 | CFTR | CF Transmembrane Conductance Regulator | GeneCards |  |  |  |
| 241 | CHCHD2 | Coiled-coil-helix-coiled-coil-helix domain-containing protein 2 | OMIM |  |  |  |
| 242 | CHI3L1 | Chitinase 3 like 1 | DisGeNET |  |  |  |
| 243 | CHIT1 | Chitinase 1 | DisGeNET |  |  |  |
| 244 | CHN2 | Chimerin 2 | DisGeNET |  |  |  |
| 245 | CHPT1 | Choline phosphotransferase 1 | DisGeNET |  |  |  |
| 246 | CLCN5 | Chloride Voltage-Gated Channel 5 | GeneCards |  |  |  |
| 247 | CLCNKA | Chloride Voltage-Gated Channel Ka | GeneCards | OMIM |  |  |
| 248 | CLCNKB | Chloride Voltage-Gated Channel Kb | GeneCards | OMIM |  |  |
| 249 | CLN3 | CLN3 Lysosomal/Endosomal Transmembrane Protein, Battenin | GeneCards |  |  |  |
| 250 | CLU | Clusterin | DisGeNET |  |  |  |
| 251 | CMKLR1 | Chemerin chemokine-like receptor 1 | DisGeNET |  |  |  |
| 252 | CNBP | CCHC-type zinc finger nucleic acid binding protein | DisGeNET |  |  |  |
| 253 | CNDP1 | Carnosine dipeptidase 1 | DisGeNET |  |  |  |
| 254 | CNDP2 | Carnosine dipeptidase 2 | DisGeNET |  |  |  |
| 255 | CNKSR3 | CNKSR family member 3 | DisGeNET |  |  |  |
| 256 | CNR1 | Cannabinoid receptor 1 | DisGeNET |  |  |  |
| 257 | COL11A2 | Collagen type XI alpha 2 chain | DisGeNET |  |  |  |
| 258 | COL18A1 | Collagen type XVIII alpha 1 chain | DisGeNET |  |  |  |
| 259 | COL1A1 | Collagen Type I Alpha 1 Chain | GeneCards | DisGeNET |  |  |
| 260 | COL1A2 | Collagen type I alpha 2 chain | DisGeNET |  |  |  |
| 261 | COL2A1 | Collagen Type II Alpha 1 Chain | GeneCards |  |  |  |
| 262 | COL4A1 | Collagen type IV alpha 1 chain | DisGeNET | GeneCards | OMIM |  |
| 263 | COL4A3 | Collagen Type IV Alpha 3 Chain | GeneCards |  |  |  |
| 264 | COL4A4 | Collagen Type IV Alpha 4 Chain | GeneCards |  |  |  |
| 265 | COL4A5 | Collagen type IV alpha 5 chain | DisGeNET | GeneCards |  |  |
| 266 | COL6A3 | Collagen type VI alpha 3 chain | DisGeNET |  |  |  |
| 267 | COL8A1 | Collagen type VIII alpha 1 chain | DisGeNET |  |  |  |
| 268 | COL8A2 | Collagen type VIII alpha 2 chain | DisGeNET |  |  |  |
| 269 | COMT | Catechol-O-Methyltransferase | GeneCards |  |  |  |
| 270 | COPA | COPI Coat Complex Subunit Alpha | GeneCards | OMIM |  |  |
| 271 | COPB2 | COPI coat complex subunit beta 2 | DisGeNET |  |  |  |
| 272 | COPD | Coatomer subunit delta | OMIM |  |  |  |
| 273 | CORO2B | Coronin 2B | DisGeNET |  |  |  |
| 274 | COX1 | Cytochrome c oxidase subunit I | DisGeNET |  |  |  |
| 275 | COX2 | Cytochrome c oxidase subunit II | DisGeNET |  |  |  |
| 276 | COX8A | Cytochrome c oxidase subunit 8A | DisGeNET |  |  |  |
| 277 | CP | Ceruloplasmin | GeneCards | DisGeNET |  |  |
| 278 | CPB2 | Carboxypeptidase B2 | DisGeNET |  |  |  |
| 279 | CPT1A | Carnitine palmitoyltransferase 1A | DisGeNET |  |  |  |
| 280 | CPT1B | Carnitine palmitoyltransferase 1B | DisGeNET |  |  |  |
| 281 | CPT2 | Carnitine Palmitoyltransferase 2 | GeneCards | DisGeNET |  |  |
| 282 | CR1 | Complement C3b/C4b receptor 1 (Knops blood group) | DisGeNET |  |  |  |
| 283 | CRB2 | Crumbs Cell Polarity Complex Component 2 | GeneCards |  |  |  |
| 284 | CREB1 | cAMP responsive element binding protein 1 | DisGeNET |  |  |  |
| 285 | CREM | cAMP responsive element modulator | DisGeNET |  |  |  |
| 286 | CRISP2 | Cysteine rich secretory protein 2 | DisGeNET |  |  |  |
| 287 | CRK | CRK proto-oncogene, adaptor protein | DisGeNET |  |  |  |
| 288 | CRMP1 | Collapsin response mediator protein 1 | DisGeNET |  |  |  |
| 289 | CRP | C-Reactive Protein | GeneCards | DisGeNET |  |  |
| 290 | CRTC1 | CREB regulated transcription coactivator 1 | DisGeNET |  |  |  |
| 291 | CRYZ | Crystallin zeta | DisGeNET |  |  |  |
| 292 | CSF1R | Colony stimulating factor 1 receptor | DisGeNET |  |  |  |
| 293 | CSNK2A2 | Casein kinase 2 alpha 2 | DisGeNET |  |  |  |
| 294 | CST2 | Cystatin SA | DisGeNET |  |  |  |
| 295 | CST3 | Cystatin C | GeneCards | DisGeNET |  |  |
| 296 | CTGF | Connective tissue growth factor | TTD |  |  |  |
| 297 | CTLA4 | Cytotoxic T-Lymphocyte Associated Protein 4 | GeneCards | OMIM |  |  |
| 298 | CTNNB1 | Catenin Beta 1 | GeneCards | DisGeNET |  |  |
| 299 | CTNS | Cystinosin, Lysosomal Cystine Transporter | GeneCards |  |  |  |
| 300 | CTSB | Cathepsin B | DisGeNET |  |  |  |
| 301 | CTSD | Cathepsin D | DisGeNET |  |  |  |
| 302 | CUBN | Cubilin | GeneCards | DisGeNET |  |  |
| 303 | CUL5 | Cullin 5 | DisGeNET |  |  |  |
| 304 | CX3CR1 | CX3C chemokine receptor 1 | OMIM |  |  |  |
| 305 | CXCL10 | C-X-C motif chemokine ligand 10 | DisGeNET |  |  |  |
| 306 | CXCL12 | C-X-C Motif Chemokine Ligand 12 | GeneCards | DisGeNET |  |  |
| 307 | CXCL16 | C-X-C motif chemokine ligand 16 | DisGeNET |  |  |  |
| 308 | CXCL6 | C-X-C motif chemokine ligand 6 | DisGeNET |  |  |  |
| 309 | CXCL8 | C-X-C Motif Chemokine Ligand 8 | GeneCards | DisGeNET |  |  |
| 310 | CXCL9 | C-X-C motif chemokine ligand 9 | DisGeNET |  |  |  |
| 311 | CXCR1 | C-X-C motif chemokine receptor 1 | DisGeNET |  |  |  |
| 312 | CXCR2 | C-X-C motif chemokine receptor 2 | DisGeNET |  |  |  |
| 313 | CXCR4 | C-X-C motif chemokine receptor 4 | DisGeNET |  |  |  |
| 314 | CYBA | Cytochrome B-245 Alpha Chain | GeneCards | DisGeNET |  |  |
| 315 | CYBB | Cytochrome B-245 Beta Chain | GeneCards | DisGeNET |  |  |
| 316 | CYLD | CYLD lysine 63 deubiquitinase | DisGeNET |  |  |  |
| 317 | CYP11B2 | Cytochrome P450 family 11 subfamily B member 2 | DisGeNET |  |  |  |
| 318 | CYP19A1 | Cytochrome P450 family 19 subfamily A member 1 | DisGeNET |  |  |  |
| 319 | CYP24A1 | Cytochrome P450 family 24 subfamily A member 1 | DisGeNET |  |  |  |
| 320 | CYP27B1 | Cytochrome P450 Family 27 Subfamily B Member 1 | GeneCards | DisGeNET |  |  |
| 321 | CYP2C19 | Cytochrome P450 2C19 | DrugBank |  |  |  |
| 322 | CYP2C8 | Cytochrome P450 2C8 | DrugBank |  |  |  |
| 323 | CYP2C9 | Cytochrome P450 2C9 | DrugBank |  |  |  |
| 324 | CYP2R1 | Cytochrome P450 family 2 subfamily R member 1 | DisGeNET |  |  |  |
| 325 | CYP3A4 | Cytochrome P450 3A4 | DrugBank |  |  |  |
| 326 | CYP4B1 | Cytochrome P450 family 4 subfamily B member 1 | DisGeNET |  |  |  |
| 327 | D2R | Dopamine D2 receptor | TTD |  |  |  |
| 328 | DAAM1 | Dishevelled associated activator of morphogenesis 1 | DisGeNET |  |  |  |
| 329 | DAPK2 | Death associated protein kinase 2 | DisGeNET |  |  |  |
| 330 | DBA2 | Diamond-Blackfan anemia 2 | DisGeNET |  |  |  |
| 331 | DBP | D-box binding PAR bZIP transcription factor | DisGeNET |  |  |  |
| 332 | DBT | Lipoamide acyltransferase component of branched-chain alpha-keto acid dehydrogenase complex, mitochondrial | OMIM |  |  |  |
| 333 | DCAF17 | DDB1 And CUL4 Associated Factor 17 | GeneCards |  |  |  |
| 334 | DCN | Decorin | DisGeNET |  |  |  |
| 335 | DCTN4 | Dynactin subunit 4 | DisGeNET |  |  |  |
| 336 | DDAH1 | Dimethylarginine dimethylaminohydrolase 1 | DisGeNET |  |  |  |
| 337 | DDAH2 | Dimethylarginine dimethylaminohydrolase 2 | DisGeNET |  |  |  |
| 338 | DDIT3 | DNA damage inducible transcript 3 | DisGeNET |  |  |  |
| 339 | DDOST | Dolichyl-diphosphooligosaccharide--protein glycosyltransferase non-catalytic subunit | DisGeNET |  |  |  |
| 340 | DDT | D-dopachrome tautomerase | DisGeNET |  |  |  |
| 341 | DECR1 | 2,4-dienoyl-CoA reductase 1 | DisGeNET |  |  |  |
| 342 | DEFA1 | Defensin alpha 1 | DisGeNET |  |  |  |
| 343 | DEFA3 | Defensin alpha 3 | DisGeNET |  |  |  |
| 344 | DENR | Density regulated re-initiation and release factor | DisGeNET |  |  |  |
| 345 | DERL2 | Derlin 2 | DisGeNET |  |  |  |
| 346 | DES | Desmin | DisGeNET |  |  |  |
| 347 | DGKA | Diacylglycerol kinase alpha | DisGeNET |  |  |  |
| 348 | DGKH | Diacylglycerol kinase eta | DisGeNET |  |  |  |
| 349 | DHX16 | Pre-mRNA-splicing factor ATP-dependent RNA helicase DHX16 | OMIM |  |  |  |
| 350 | DIANPH | Diabetic nephropathy | DisGeNET |  |  |  |
| 351 | DKK1 | Dickkopf WNT signaling pathway inhibitor 1 | DisGeNET |  |  |  |
| 352 | DMP1 | Dentin matrix acidic phosphoprotein 1 | DisGeNET |  |  |  |
| 353 | DMTF1 | Cyclin D binding myb like transcription factor 1 | DisGeNET |  |  |  |
| 354 | DNAJB11 | DnaJ Heat Shock Protein Family (Hsp40) Member B11 | GeneCards | OMIM |  |  |
| 355 | DNAJC3 | DnaJ homolog subfamily C member 3 | OMIM |  |  |  |
| 356 | DNAJC6 | Putative tyrosine-protein phosphatase auxilin | OMIM |  |  |  |
| 357 | DNM1L | Dynamin 1 like | DisGeNET |  |  |  |
| 358 | DNM2 | Dynamin 2 | GeneCards |  |  |  |
| 359 | DNMT1 | DNA methyltransferase 1 | DisGeNET |  |  |  |
| 360 | DNMT3A | DNA methyltransferase 3 alpha | DisGeNET |  |  |  |
| 361 | DNMT3B | DNA methyltransferase 3 beta | DisGeNET |  |  |  |
| 362 | DPP4 | Dipeptidyl Peptidase 4 | GeneCards | DisGeNET |  |  |
| 363 | DPYD | Dihydropyrimidine dehydrogenase | DisGeNET |  |  |  |
| 364 | DPYS | Dihydropyrimidinase | DisGeNET |  |  |  |
| 365 | DRD3 | Dopamine receptor D3 | DisGeNET |  |  |  |
| 366 | DRG1 | Developmentally regulated GTP binding protein 1 | DisGeNET |  |  |  |
| 367 | DSEL | Dermatan sulfate epimerase like | DisGeNET |  |  |  |
| 368 | DSPP | Dentin sialophosphoprotein | DisGeNET |  |  |  |
| 369 | DSTYK | Dual Serine/Threonine And Tyrosine Protein Kinase | GeneCards | OMIM |  |  |
| 370 | DUOX1 | Dual oxidase 1 | DisGeNET |  |  |  |
| 371 | DUOX2 | Dual oxidase 2 | DisGeNET |  |  |  |
| 372 | DUSP1 | Dual specificity phosphatase 1 | DisGeNET |  |  |  |
| 373 | DUSP26 | Dual specificity phosphatase 26 | DisGeNET |  |  |  |
| 374 | DUSP4 | Dual specificity phosphatase 4 | DisGeNET |  |  |  |
| 375 | DYNC1H1 | Dynein Cytoplasmic 1 Heavy Chain 1 | GeneCards |  |  |  |
| 376 | DYNC2H1 | Dynein Cytoplasmic 2 Heavy Chain 1 | GeneCards |  |  |  |
| 377 | DZIP1L | DAZ Interacting Zinc Finger Protein 1 Like | GeneCards | OMIM |  |  |
| 378 | E2F1 | E2F transcription factor 1 | DisGeNET |  |  |  |
| 379 | E2F3 | E2F transcription factor 3 | DisGeNET |  |  |  |
| 380 | EBI3 | Epstein-Barr virus induced 3 | DisGeNET |  |  |  |
| 381 | ECE1 | Endothelin-converting enzyme 1 | OMIM |  |  |  |
| 382 | ECM1 | Extracellular matrix protein 1 | OMIM |  |  |  |
| 383 | EDA | Ectodysplasin A | DisGeNET |  |  |  |
| 384 | EDN1 | Endothelin 1 | GeneCards | DisGeNET |  |  |
| 385 | EDN3 | Endothelin 3 | GeneCards |  |  |  |
| 386 | EDNRA | Endothelin A receptor | TTD | DisGeNET |  |  |
| 387 | EDNRB | Endothelin Receptor Type B | GeneCards |  |  |  |
| 388 | EFHD2 | EF-hand domain family member D2 | DisGeNET |  |  |  |
| 389 | EFNA1 | Ephrin A1 | DisGeNET |  |  |  |
| 390 | EGF | Epidermal Growth Factor | GeneCards | DisGeNET |  |  |
| 391 | EGFR | Epidermal Growth Factor Receptor | GeneCards | DisGeNET | OMIM |  |
| 392 | EGR1 | Early growth response 1 | DisGeNET |  |  |  |
| 393 | EGR2 | Early Growth Response 2 | GeneCards |  |  |  |
| 394 | EHMT1 | Euchromatic histone lysine methyltransferase 1 | DisGeNET |  |  |  |
| 395 | EIF2AK3 | Eukaryotic Translation Initiation Factor 2 Alpha Kinase 3 | GeneCards | DisGeNET |  |  |
| 396 | EIF3M | Eukaryotic translation initiation factor 3 subunit M | DisGeNET |  |  |  |
| 397 | EIF4G1 | Eukaryotic translation initiation factor 4 gamma 1 | OMIM |  |  |  |
| 398 | ELAVL1 | ELAV like RNA binding protein 1 | DisGeNET |  |  |  |
| 399 | ELAVL2 | ELAV like RNA binding protein 2 | DisGeNET |  |  |  |
| 400 | ELF3 | E74 like ETS transcription factor 3 | DisGeNET |  |  |  |
| 401 | ELMO1 | Engulfment and cell motility 1 | DisGeNET |  |  |  |
| 402 | ELN | Elastin | GeneCards |  |  |  |
| 403 | ELOVL4 | Elongation of very long chain fatty acids protein 4 | OMIM |  |  |  |
| 404 | ENG | Endoglin | GeneCards | DisGeNET |  |  |
| 405 | ENO2 | Enolase 2 | DisGeNET |  |  |  |
| 406 | ENOX1 | Ecto-NOX disulfide-thiol exchanger 1 | DisGeNET |  |  |  |
| 407 | ENPEP | Glutamyl aminopeptidase | DisGeNET |  |  |  |
| 408 | ENPP1 | Ectonucleotide Pyrophosphatase/Phosphodiesterase 1 | GeneCards | DisGeNET | OMIM |  |
| 409 | ENTPD1 | Ectonucleoside triphosphate diphosphohydrolase 1 | DisGeNET |  |  |  |
| 410 | EP300 | E1A Binding Protein P300 | GeneCards | DisGeNET |  |  |
| 411 | EPAS1 | Endothelial PAS domain protein 1 | DisGeNET |  |  |  |
| 412 | EPHA1 | EPH receptor A1 | DisGeNET |  |  |  |
| 413 | EPHB2 | EPH receptor B2 | DisGeNET |  |  |  |
| 414 | EPHX2 | Epoxide hydrolase 2 | DisGeNET |  |  |  |
| 415 | EPO | Erythropoietin | GeneCards | DisGeNET | OMIM |  |
| 416 | ERBB2 | Erb-B2 Receptor Tyrosine Kinase 2 | GeneCards |  |  |  |
| 417 | ERBB4 | Erb-b2 receptor tyrosine kinase 4 | DisGeNET |  |  |  |
| 418 | EREG | Epiregulin | DisGeNET |  |  |  |
| 419 | ERP44 | Endoplasmic reticulum protein 44 | DisGeNET |  |  |  |
| 420 | ERRFI1 | ERBB receptor feedback inhibitor 1 | DisGeNET |  |  |  |
| 421 | ESAM | Endothelial cell adhesion molecule | DisGeNET |  |  |  |
| 422 | ESCO1 | Establishment of sister chromatid cohesion N-acetyltransferase 1 | DisGeNET |  |  |  |
| 423 | ESM1 | Endothelial cell specific molecule 1 | DisGeNET |  |  |  |
| 424 | ESR1 | Estrogen Receptor 1 | GeneCards | DisGeNET |  |  |
| 425 | ETF1 | Eukaryotic translation termination factor 1 | DisGeNET |  |  |  |
| 426 | ETS1 | ETS proto-oncogene 1, transcription factor | DisGeNET |  |  |  |
| 427 | EZH2 | Enhancer of zeste 2 polycomb repressive complex 2 subunit | DisGeNET |  |  |  |
| 428 | EZR | Ezrin | DisGeNET |  |  |  |
| 429 | F10 | Coagulation factor X | DisGeNET |  |  |  |
| 430 | F2 | Coagulation Factor II, Thrombin | GeneCards |  |  |  |
| 431 | F2R | Coagulation factor II thrombin receptor | DisGeNET |  |  |  |
| 432 | F2RL1 | F2R like trypsin receptor 1 | DisGeNET |  |  |  |
| 433 | F3 | Coagulation Factor III, Tissue Factor | GeneCards |  |  |  |
| 434 | F5 | Coagulation Factor V | GeneCards | DisGeNET |  |  |
| 435 | F8 | Coagulation Factor VIII | GeneCards |  |  |  |
| 436 | FABP1 | Fatty acid binding protein 1 | DisGeNET |  |  |  |
| 437 | FABP2 | Fatty acid binding protein 2 | DisGeNET |  |  |  |
| 438 | FABP4 | Fatty acid binding protein 4 | DisGeNET |  |  |  |
| 439 | FAR2 | Fatty acyl-CoA reductase 2 | DisGeNET |  |  |  |
| 440 | FARSB | Phenylalanine--tRNA ligase beta subunit | OMIM |  |  |  |
| 441 | FAS | Fas Cell Surface Death Receptor | GeneCards | DisGeNET |  |  |
| 442 | FASLG | Fas Ligand | GeneCards |  |  |  |
| 443 | FASN | Fatty acid synthase | DisGeNET |  |  |  |
| 444 | FBN1 | Fibrillin 1 | GeneCards |  |  |  |
| 445 | FBXW7 | F-box and WD repeat domain containing 7 | DisGeNET |  |  |  |
| 446 | FCGR2A | Immunoglobulin gamma Fc receptor IIA | TTD | GeneCards |  |  |
| 447 | FFAR2 | Free fatty acid receptor 2 | DisGeNET |  |  |  |
| 448 | FGF1 | Fibroblast growth factor 1 | DisGeNET |  |  |  |
| 449 | FGF11 | Fibroblast growth factor 11 | DisGeNET |  |  |  |
| 450 | FGF13 | Fibroblast growth factor 13 | DisGeNET |  |  |  |
| 451 | FGF2 | Fibroblast Growth Factor 2 | GeneCards | DisGeNET |  |  |
| 452 | FGF21 | Fibroblast growth factor 21 | DisGeNET |  |  |  |
| 453 | FGF23 | Fibroblast Growth Factor 23 | GeneCards | DisGeNET |  |  |
| 454 | FGFR1 | Fibroblast Growth Factor Receptor 1 | GeneCards |  |  |  |
| 455 | FGFR2 | Fibroblast Growth Factor Receptor 2 | GeneCards |  |  |  |
| 456 | FGFR3 | Fibroblast Growth Factor Receptor 3 | GeneCards |  |  |  |
| 457 | FHL2 | Four and a half LIM domains 2 | DisGeNET |  |  |  |
| 458 | FIG4 | FIG4 Phosphoinositide 5-Phosphatase | GeneCards | OMIM |  |  |
| 459 | FIS1 | Fission, mitochondrial 1 | DisGeNET |  |  |  |
| 460 | FITM1 | Fat storage inducing transmembrane protein 1 | DisGeNET |  |  |  |
| 461 | FLT1 | Fms Related Receptor Tyrosine Kinase 1 | GeneCards | DisGeNET |  |  |
| 462 | FLT4 | Fms related receptor tyrosine kinase 4 | DisGeNET |  |  |  |
| 463 | FN1 | Fibronectin 1 | GeneCards | DisGeNET |  |  |
| 464 | FN3K | Fructosamine 3 kinase | DisGeNET |  |  |  |
| 465 | FNDC5 | Fibronectin type III domain containing 5 | DisGeNET |  |  |  |
| 466 | FOXA1 | Forkhead box A1 | DisGeNET |  |  |  |
| 467 | FOXC1 | Forkhead box C1 | DisGeNET |  |  |  |
| 468 | FOXC2 | Forkhead box protein C2 | OMIM |  |  |  |
| 469 | FOXD3 | Forkhead box protein D3 | OMIM |  |  |  |
| 470 | FOXO1 | Forkhead box O1 | DisGeNET |  |  |  |
| 471 | FOXP1 | Forkhead box P1 | DisGeNET |  |  |  |
| 472 | FOXP3 | Forkhead Box P3 | GeneCards | DisGeNET |  |  |
| 473 | FPR2 | Formyl peptide receptor 2 | DisGeNET |  |  |  |
| 474 | FRMD3 | FERM domain containing 3 | DisGeNET |  |  |  |
| 475 | FSCN1 | Fascin actin-bundling protein 1 | DisGeNET |  |  |  |
| 476 | FSD1 | Fibronectin type III and SPRY domain containing 1 | DisGeNET |  |  |  |
| 477 | FSD1L | Fibronectin type III and SPRY domain containing 1 like | DisGeNET |  |  |  |
| 478 | FST | Follistatin | DisGeNET |  |  |  |
| 479 | FTO | FTO alpha-ketoglutarate dependent dioxygenase | DisGeNET |  |  |  |
| 480 | FUT8 | Fucosyltransferase 8 | DisGeNET |  |  |  |
| 481 | G6PC1 | Glucose-6-Phosphatase Catalytic Subunit 1 | GeneCards |  |  |  |
| 482 | G6PD | Glucose-6-phosphate dehydrogenase | DisGeNET |  |  |  |
| 483 | GAA | Alpha Glucosidase | GeneCards |  |  |  |
| 484 | GABPA | GA binding protein transcription factor subunit alpha | DisGeNET |  |  |  |
| 485 | GAD2 | Glutamate Decarboxylase 2 | GeneCards |  |  |  |
| 486 | GALC | Galactosylceramidase | GeneCards |  |  |  |
| 487 | GANAB | Glucosidase II Alpha Subunit | GeneCards |  |  |  |
| 488 | GARS1 | Glycyl-TRNA Synthetase 1 | GeneCards | OMIM |  |  |
| 489 | GAS5 | Growth arrest specific 5 | DisGeNET |  |  |  |
| 490 | GAS6 | Growth arrest specific 6 | DisGeNET |  |  |  |
| 491 | GAST | Gastrin | DisGeNET |  |  |  |
| 492 | GATA3 | GATA Binding Protein 3 | GeneCards |  |  |  |
| 493 | GATA4 | GATA Binding Protein 4 | GeneCards |  |  |  |
| 494 | GATA6 | GATA Binding Protein 6 | GeneCards |  |  |  |
| 495 | GBA | Glucosylceramidase Beta | GeneCards | OMIM |  |  |
| 496 | GBD2 | Dirigent protein | OMIM |  |  |  |
| 497 | GBE1 | 1,4-Alpha-Glucan Branching Enzyme 1 | GeneCards | OMIM |  |  |
| 498 | GC | GC vitamin D binding protein | DisGeNET |  |  |  |
| 499 | GCG | Glucagon | GeneCards | DisGeNET |  |  |
| 500 | GCK | Glucokinase | GeneCards | DisGeNET | OMIM |  |
| 501 | GDAP1 | Ganglioside Induced Differentiation Associated Protein 1 | GeneCards |  |  |  |
| 502 | GDE1 | Glycerophosphodiester phosphodiesterase 1 | DisGeNET |  |  |  |
| 503 | GDF15 | Growth differentiation factor 15 | DisGeNET |  |  |  |
| 504 | GDNF | Glial Cell Derived Neurotrophic Factor | GeneCards | OMIM |  |  |
| 505 | GFAP | Glial Fibrillary Acidic Protein | GeneCards |  |  |  |
| 506 | GFPT1 | Glutamine--fructose-6-phosphate transaminase 1 | DisGeNET |  |  |  |
| 507 | GFPT2 | Glutamine-fructose-6-phosphate transaminase 2 | DisGeNET |  |  |  |
| 508 | GGT1 | Gamma-Glutamyltransferase 1 | GeneCards |  |  |  |
| 509 | GH1 | Growth hormone 1 | DisGeNET |  |  |  |
| 510 | GHR | Growth hormone receptor | DisGeNET |  |  |  |
| 511 | GHRL | Ghrelin And Obestatin Prepropeptide | GeneCards | DisGeNET |  |  |
| 512 | GIGYF2 | GRB10-interacting GYF protein 2 | OMIM |  |  |  |
| 513 | GIPR | Gastric inhibitory polypeptide receptor | DisGeNET |  |  |  |
| 514 | GJA1 | Gap junction protein alpha 1 | DisGeNET |  |  |  |
| 515 | GJB1 | Gap Junction Protein Beta 1 | GeneCards | DisGeNET |  |  |
| 516 | GJB2 | Gap Junction Protein Beta 2 | GeneCards |  |  |  |
| 517 | GLA | Galactosidase Alpha | GeneCards |  |  |  |
| 518 | GLIPR2 | GLI pathogenesis related 2 | DisGeNET |  |  |  |
| 519 | GLIS2 | GLIS Family Zinc Finger 2 | GeneCards |  |  |  |
| 520 | GLIS3 | GLIS Family Zinc Finger 3 | GeneCards | OMIM |  |  |
| 521 | GLO1 | Glyoxalase I | DisGeNET |  |  |  |
| 522 | GLP1R | Glucagon like peptide 1 receptor | DisGeNET |  |  |  |
| 523 | GNAS | GNAS Complex Locus | GeneCards |  |  |  |
| 524 | GNB3 | G Protein Subunit Beta 3 | GeneCards |  |  |  |
| 525 | GNB4 | Guanine nucleotide-binding protein subunit beta-4 | OMIM |  |  |  |
| 526 | GOLGA6A | Golgin A6 family member A | DisGeNET |  |  |  |
| 527 | GORASP1 | Golgi reassembly stacking protein 1 | DisGeNET |  |  |  |
| 528 | GP1BA | Glycoprotein Ib Platelet Subunit Alpha | GeneCards |  |  |  |
| 529 | GPBAR1 | G protein-coupled bile acid receptor 1 | DisGeNET |  |  |  |
| 530 | GPC5 | Glypican 5 | DisGeNET |  |  |  |
| 531 | GPD2 | Glycerol-3-Phosphate Dehydrogenase 2 | GeneCards | OMIM |  |  |
| 532 | GPR158 | G protein-coupled receptor 158 | DisGeNET |  |  |  |
| 533 | GPRC5A | G protein-coupled receptor class C group 5 member A | DisGeNET |  |  |  |
| 534 | GPRC5B | G protein-coupled receptor class C group 5 member B | DisGeNET |  |  |  |
| 535 | GPT | Glutamic--Pyruvic Transaminase | GeneCards |  |  |  |
| 536 | GPX1 | Glutathione peroxidase 1 | DisGeNET |  |  |  |
| 537 | GPX3 | Glutathione peroxidase 3 | DisGeNET |  |  |  |
| 538 | GPX4 | Glutathione peroxidase 4 | DisGeNET |  |  |  |
| 539 | GRAP | GRB2 related adaptor protein | DisGeNET |  |  |  |
| 540 | GRAP2 | GRB2 related adaptor protein 2 | DisGeNET |  |  |  |
| 541 | GRB2 | Growth factor receptor bound protein 2 | DisGeNET |  |  |  |
| 542 | GREM1 | Gremlin 1, DAN family BMP antagonist | DisGeNET |  |  |  |
| 543 | GREM2 | Gremlin 2, DAN family BMP antagonist | DisGeNET |  |  |  |
| 544 | GRK2 | G protein-coupled receptor kinase 2 | DisGeNET |  |  |  |
| 545 | GRK3 | G protein-coupled receptor kinase 3 | DisGeNET |  |  |  |
| 546 | GRK4 | G protein-coupled receptor kinase 4 | DisGeNET |  |  |  |
| 547 | GRK6 | G protein-coupled receptor kinase 6 | DisGeNET |  |  |  |
| 548 | GRN | Granulin precursor | DisGeNET |  |  |  |
| 549 | GSDMD | Gasdermin D | DisGeNET |  |  |  |
| 550 | GSK3B | Glycogen synthase kinase 3 beta | DisGeNET |  |  |  |
| 551 | GSR | Glutathione-disulfide reductase | DisGeNET |  |  |  |
| 552 | GSTK1 | Glutathione S-transferase kappa 1 | DisGeNET |  |  |  |
| 553 | GSTM1 | Glutathione S-transferase mu 1 | DisGeNET |  |  |  |
| 554 | GSTM2 | Glutathione S-transferase mu 2 | DisGeNET |  |  |  |
| 555 | GSTP1 | Glutathione S-transferase pi 1 | DisGeNET |  |  |  |
| 556 | GSTT1 | Glutathione S-transferase theta 1 | DisGeNET |  |  |  |
| 557 | GTF2H1 | General transcription factor IIH subunit 1 | DisGeNET |  |  |  |
| 558 | GUCY1A1 | Guanylate cyclase 1 soluble subunit alpha 1 | DisGeNET |  |  |  |
| 559 | GYG1 | Glycogenin-1 | OMIM |  |  |  |
| 560 | GYS1 | Glycogen Synthase 1 | GeneCards |  |  |  |
| 561 | H19 | H19 Imprinted Maternally Expressed Transcript | GeneCards |  |  |  |
| 562 | H2BS1 | H2B.S histone 1 | DisGeNET |  |  |  |
| 563 | H3P10 | H3 histone pseudogene 10 | DisGeNET |  |  |  |
| 564 | HAMP | Hepcidin Antimicrobial Peptide | GeneCards |  |  |  |
| 565 | HARS1 | Histidine--tRNA ligase, cytoplasmic | OMIM |  |  |  |
| 566 | HAS2 | Hyaluronan synthase 2 | DisGeNET |  |  |  |
| 567 | HAVCR1 | Hepatitis A virus cellular receptor 1 | DisGeNET |  |  |  |
| 568 | HAVCR2 | Hepatitis A virus cellular receptor 2 | DisGeNET |  |  |  |
| 569 | HBA1 | Hemoglobin Subunit Alpha 1 | GeneCards |  |  |  |
| 570 | HBB | Hemoglobin Subunit Beta | GeneCards |  |  |  |
| 571 | HCN1 | Hyperpolarization activated cyclic nucleotide gated potassium channel 1 | DisGeNET |  |  |  |
| 572 | HDAC2 | Histone deacetylase 2 | DisGeNET |  |  |  |
| 573 | HDAC4 | Histone deacetylase 4 | DisGeNET |  |  |  |
| 574 | HDAC5 | Histone deacetylase 5 | DisGeNET |  |  |  |
| 575 | HDLBP | High density lipoprotein binding protein | DisGeNET |  |  |  |
| 576 | HEBP1 | Heme binding protein 1 | DisGeNET |  |  |  |
| 577 | HES1 | Hes family bHLH transcription factor 1 | DisGeNET |  |  |  |
| 578 | HEXA | Hexosaminidase Subunit Alpha | GeneCards |  |  |  |
| 579 | HEXB | Beta-hexosaminidase subunit beta | OMIM |  |  |  |
| 580 | HFE | Homeostatic Iron Regulator | GeneCards | DisGeNET | OMIM |  |
| 581 | HFE-AS1 | HFE Antisense RNA 1 | GeneCards |  |  |  |
| 582 | HGF | Hepatocyte growth factor | DisGeNET |  |  |  |
| 583 | HHIP | Hedgehog interacting protein | DisGeNET |  |  |  |
| 584 | HIF1A | Hypoxia Inducible Factor 1 Subunit Alpha | GeneCards | DisGeNET |  |  |
| 585 | HLA-A | Major Histocompatibility Complex, Class I, A | GeneCards |  |  |  |
| 586 | HLA-B | Major Histocompatibility Complex, Class I, B | GeneCards | DisGeNET |  |  |
| 587 | HLA-DPA1 | Major histocompatibility complex, class II, DP alpha 1 | DisGeNET |  |  |  |
| 588 | HLA-DPB1 | Major Histocompatibility Complex, Class II, DP Beta 1 | GeneCards | OMIM |  |  |
| 589 | HLA-DQA1 | Major Histocompatibility Complex, Class II, DQ Alpha 1 | GeneCards | DisGeNET | OMIM |  |
| 590 | HLA-DQB1 | Major Histocompatibility Complex, Class II, DQ Beta 1 | GeneCards | OMIM |  |  |
| 591 | HLA-DRB1 | Major Histocompatibility Complex, Class II, DR Beta 1 | GeneCards | DisGeNET |  |  |
| 592 | HMCN1 | Hemicentin 1 | DisGeNET |  |  |  |
| 593 | HMGA1 | High Mobility Group AT-Hook 1 | GeneCards | OMIM |  |  |
| 594 | HMGA2 | High mobility group AT-hook 2 | DisGeNET |  |  |  |
| 595 | HMGB1 | High mobility group box 1 | DisGeNET |  |  |  |
| 596 | HMGN1 | High mobility group nucleosome binding domain 1 | DisGeNET |  |  |  |
| 597 | HMOX1 | Heme Oxygenase 1 | GeneCards | DisGeNET |  |  |
| 598 | HNF1A | HNF1 Homeobox A | GeneCards | DisGeNET | OMIM |  |
| 599 | HNF1B | HNF1 Homeobox B | GeneCards | DisGeNET | OMIM |  |
| 600 | HNF4A | Hepatocyte Nuclear Factor 4 Alpha | GeneCards | DisGeNET | OMIM |  |
| 601 | HNMT | Histamine N-methyltransferase | DisGeNET |  |  |  |
| 602 | HNRNPUL2-BSCL2 | HNRNPUL2-BSCL2 Readthrough (NMD Candidate) | GeneCards |  |  |  |
| 603 | HNRPA2B1 | Heterogeneous nuclear ribonucleoproteins A2/B1 | OMIM |  |  |  |
| 604 | HOTAIR | HOX transcript antisense RNA | DisGeNET |  |  |  |
| 605 | HOXD10 | Homeobox protein Hox-D10 | OMIM |  |  |  |
| 606 | HP | Haptoglobin | GeneCards | DisGeNET |  |  |
| 607 | HPGDS | Hematopoietic prostaglandin D synthase | DisGeNET |  |  |  |
| 608 | HPSE | Heparanase | DisGeNET |  |  |  |
| 609 | HPT | Hypoparathyroidism | DisGeNET |  |  |  |
| 610 | HRAS | HRas Proto-Oncogene, GTPase | GeneCards |  |  |  |
| 611 | HSD11B2 | Hydroxysteroid 11-beta dehydrogenase 2 | DisGeNET |  |  |  |
| 612 | HSPA1A | Heat shock protein family A (Hsp70) member 1A | DisGeNET |  |  |  |
| 613 | HSPA1B | Heat shock protein family A (Hsp70) member 1B | DisGeNET |  |  |  |
| 614 | HSPA4 | Heat shock protein family A (Hsp70) member 4 | DisGeNET |  |  |  |
| 615 | HSPA5 | Heat shock protein family A (Hsp70) member 5 | DisGeNET |  |  |  |
| 616 | HSPB1 | Heat Shock Protein Family B (Small) Member 1 | GeneCards | DisGeNET | OMIM |  |
| 617 | HSPB2 | Heat shock protein family B (small) member 2 | DisGeNET |  |  |  |
| 618 | HSPB3 | Heat shock protein family B (small) member 3 | DisGeNET |  |  |  |
| 619 | HSPD1 | Heat Shock Protein Family D (Hsp60) Member 1 | GeneCards |  |  |  |
| 620 | HSPG2 | Heparan sulfate proteoglycan 2 | DisGeNET |  |  |  |
| 621 | HTR2A | 5-hydroxytryptamine receptor 2A | TTD | DisGeNET |  |  |
| 622 | HTRA2 | Serine protease HTRA2, mitochondrial | OMIM |  |  |  |
| 623 | HTT | Huntingtin | GeneCards | OMIM |  |  |
| 624 | HYMAI | Hydatidiform Mole Associated And Imprinted | GeneCards |  |  |  |
| 625 | HYOU1 | Hypoxia up-regulated 1 | DisGeNET |  |  |  |
| 626 | IAPP | Islet Amyloid Polypeptide | GeneCards | DisGeNET | OMIM |  |
| 627 | ICAM1 | Intercellular Adhesion Molecule 1 | GeneCards | DisGeNET |  |  |
| 628 | ID2 | Inhibitor of DNA binding 2 | DisGeNET |  |  |  |
| 629 | IDUA | Alpha-L-iduronidase | DisGeNET |  |  |  |
| 630 | IER3IP1 | Immediate early response 3-interacting protein 1 | OMIM |  |  |  |
| 631 | IFIH1 | Interferon Induced With Helicase C Domain 1 | GeneCards |  |  |  |
| 632 | IFNA1 | Interferon alpha 1 | DisGeNET |  |  |  |
| 633 | IFNA13 | Interferon alpha 13 | DisGeNET |  |  |  |
| 634 | IFNG | Interferon Gamma | GeneCards | DisGeNET |  |  |
| 635 | IFT140 | Intraflagellar Transport 140 | GeneCards |  |  |  |
| 636 | IFT172 | Intraflagellar Transport 172 | GeneCards |  |  |  |
| 637 | IFT88 | Intraflagellar Transport 88 | GeneCards |  |  |  |
| 638 | IGAN1 | IgA nephropathy | DisGeNET | OMIM |  |  |
| 639 | IGF1 | Insulin Like Growth Factor 1 | GeneCards | DisGeNET |  |  |
| 640 | IGF1R | Insulin Like Growth Factor 1 Receptor | TTD | GeneCards | DisGeNET |  |
| 641 | IGF2 | Insulin Like Growth Factor 2 | GeneCards | DisGeNET |  |  |
| 642 | IGF2BP2 | Insulin Like Growth Factor 2 MRNA Binding Protein 2 | GeneCards | DisGeNET | OMIM |  |
| 643 | IGFBP1 | Insulin like growth factor binding protein 1 | DisGeNET |  |  |  |
| 644 | IGFBP3 | Insulin Like Growth Factor Binding Protein 3 | GeneCards | DisGeNET |  |  |
| 645 | IGFBP4 | Insulin like growth factor binding protein 4 | DisGeNET |  |  |  |
| 646 | IGFBP7 | Insulin like growth factor binding protein 7 | DisGeNET |  |  |  |
| 647 | IGHMBP2 | Immunoglobulin Mu DNA Binding Protein 2 | GeneCards |  |  |  |
| 648 | IHG1 | Iris hypoplasia with glaucoma 1 | DisGeNET |  |  |  |
| 649 | IKBKB | Inhibitor of nuclear factor kappa B kinase subunit beta | DisGeNET |  |  |  |
| 650 | IL10 | Interleukin 10 | GeneCards | DisGeNET | OMIM |  |
| 651 | IL10RA | Interleukin 10 Receptor Subunit Alpha | GeneCards |  |  |  |
| 652 | IL12RB1 | Interleukin 12 receptor subunit beta 1 | DisGeNET |  |  |  |
| 653 | IL13 | Interleukin 13 | DisGeNET |  |  |  |
| 654 | IL15 | Interleukin 15 | DisGeNET |  |  |  |
| 655 | IL17A | Interleukin 17A | GeneCards | DisGeNET |  |  |
| 656 | IL17B | Interleukin 17B | DisGeNET |  |  |  |
| 657 | IL18 | Interleukin 18 | GeneCards | DisGeNET |  |  |
| 658 | IL18R1 | Interleukin 18 receptor 1 | DisGeNET |  |  |  |
| 659 | IL19 | Interleukin 19 | DisGeNET |  |  |  |
| 660 | IL1A | Interleukin 1 Alpha | GeneCards | DisGeNET |  |  |
| 661 | IL1B | Interleukin 1 Beta | GeneCards | DisGeNET |  |  |
| 662 | IL1R1 | Interleukin 1 receptor type 1 | DisGeNET |  |  |  |
| 663 | IL1RL1 | Interleukin 1 receptor like 1 | DisGeNET |  |  |  |
| 664 | IL1RN | Interleukin 1 Receptor Antagonist | GeneCards | DisGeNET | OMIM |  |
| 665 | IL2 | Interleukin 2 | GeneCards | DisGeNET |  |  |
| 666 | IL20 | Interleukin 20 | DisGeNET |  |  |  |
| 667 | IL22 | Interleukin 22 | DisGeNET |  |  |  |
| 668 | IL23R | Interleukin 23 Receptor | GeneCards | OMIM |  |  |
| 669 | IL2RA | Interleukin 2 Receptor Subunit Alpha | GeneCards | OMIM |  |  |
| 670 | IL33 | Interleukin 33 | DisGeNET |  |  |  |
| 671 | IL34 | Interleukin 34 | DisGeNET |  |  |  |
| 672 | IL4 | Interleukin 4 | GeneCards | DisGeNET |  |  |
| 673 | IL5 | Interleukin 5 | DisGeNET |  |  |  |
| 674 | IL6 | Interleukin 6 | GeneCards | DisGeNET | OMIM |  |
| 675 | IL6R | Interleukin 6 receptor | DisGeNET |  |  |  |
| 676 | IL6ST | Interleukin 6 signal transducer | DisGeNET |  |  |  |
| 677 | INAVA | Innate immunity activator protein | OMIM |  |  |  |
| 678 | INF2 | Inverted Formin 2 | GeneCards |  |  |  |
| 679 | INHBC | Inhibin subunit beta C | DisGeNET |  |  |  |
| 680 | INPP5E | Inositol Polyphosphate-5-Phosphatase E | GeneCards |  |  |  |
| 681 | INPPL1 | Inositol polyphosphate phosphatase like 1 | DisGeNET |  |  |  |
| 682 | INS | Insulin | GeneCards | DisGeNET | OMIM |  |
| 683 | INS-IGF2 | INS-IGF2 Readthrough | GeneCards |  |  |  |
| 684 | INSR | Insulin Receptor | TTD | GeneCards | DisGeNET |  |
| 685 | INTU | Inturned planar cell polarity protein | DisGeNET |  |  |  |
| 686 | INVS | Inversin | GeneCards |  |  |  |
| 687 | IPCEF1 | Interaction protein for cytohesin exchange factors 1 | DisGeNET |  |  |  |
| 688 | IPPK | Inositol-pentakisphosphate 2-kinase | DisGeNET |  |  |  |
| 689 | IQCB1 | IQ Motif Containing B1 | GeneCards |  |  |  |
| 690 | IQGAP1 | IQ motif containing GTPase activating protein 1 | DisGeNET |  |  |  |
| 691 | IRAK1 | Interleukin 1 receptor associated kinase 1 | DisGeNET |  |  |  |
| 692 | IRAK4 | Interleukin 1 receptor associated kinase 4 | DisGeNET |  |  |  |
| 693 | IRF5 | Interferon regulatory factor 5 | OMIM |  |  |  |
| 694 | IRGM | Immunity-related GTPase family M protein | OMIM |  |  |  |
| 695 | IRS1 | Insulin Receptor Substrate 1 | GeneCards | DisGeNET | OMIM |  |
| 696 | IRS2 | Insulin Receptor Substrate 2 | GeneCards | DisGeNET | OMIM |  |
| 697 | ISYNA1 | Inositol-3-phosphate synthase 1 | DisGeNET |  |  |  |
| 698 | ITGA1 | Integrin subunit alpha 1 | DisGeNET |  |  |  |
| 699 | ITGA2B | Integrin subunit alpha 2b | DisGeNET |  |  |  |
| 700 | ITGAM | Integrin subunit alpha M | DisGeNET |  |  |  |
| 701 | ITGB3 | Integrin subunit beta 3 | DisGeNET |  |  |  |
| 702 | ITPR1 | Inositol 1,4,5-trisphosphate receptor type 1 | DisGeNET |  |  |  |
| 703 | ITPR3 | Inositol 1,4,5-Trisphosphate Receptor Type 3 | GeneCards | OMIM |  |  |
| 704 | JAG1 | Jagged Canonical Notch Ligand 1 | GeneCards | DisGeNET |  |  |
| 705 | JAK1 | Janus kinase 1 | DisGeNET |  |  |  |
| 706 | JAK2 | Janus Kinase 2 | GeneCards | DisGeNET |  |  |
| 707 | JUN | Jun proto-oncogene, AP-1 transcription factor subunit | DisGeNET | DrugBank |  |  |
| 708 | KAAG1 | Kidney-associated antigen 1 | OMIM |  |  |  |
| 709 | KCNH7 | Potassium voltage-gated channel subfamily H member 7 | DisGeNET |  |  |  |
| 710 | KCNJ11 | Potassium Inwardly Rectifying Channel Subfamily J Member 11 | GeneCards | DisGeNET | OMIM |  |
| 711 | KCNQ1 | Potassium Voltage-Gated Channel Subfamily Q Member 1 | GeneCards | DisGeNET |  |  |
| 712 | KDM6A | Lysine demethylase 6A | DisGeNET |  |  |  |
| 713 | KDR | Kinase insert domain receptor | DisGeNET |  |  |  |
| 714 | KEAP1 | Kelch like ECH associated protein 1 | DisGeNET |  |  |  |
| 715 | KHDRBS1 | KH RNA binding domain containing, signal transduction associated 1 | DisGeNET |  |  |  |
| 716 | KHK | Ketohexokinase | DisGeNET |  |  |  |
| 717 | KIF1B | Kinesin Family Member 1B | GeneCards | OMIM |  |  |
| 718 | KIRREL1 | Kirre like nephrin family adhesion molecule 1 | DisGeNET |  |  |  |
| 719 | KIT | KIT Proto-Oncogene, Receptor Tyrosine Kinase | GeneCards |  |  |  |
| 720 | KL | Klotho | GeneCards | DisGeNET |  |  |
| 721 | KLF11 | Kruppel Like Factor 11 | GeneCards | OMIM |  |  |
| 722 | KLF15 | Kruppel like factor 15 | DisGeNET |  |  |  |
| 723 | KLF2 | Kruppel like factor 2 | DisGeNET |  |  |  |
| 724 | KLF4 | Kruppel like factor 4 | DisGeNET |  |  |  |
| 725 | KLF6 | Kruppel like factor 6 | DisGeNET |  |  |  |
| 726 | KLK1 | Kallikrein 1 | DisGeNET |  |  |  |
| 727 | KMT2A | Lysine methyltransferase 2A | DisGeNET |  |  |  |
| 728 | KMT5AP1 | KMT5A pseudogene 1 | DisGeNET |  |  |  |
| 729 | KNG1 | Kininogen 1 | DisGeNET |  |  |  |
| 730 | KPNA2 | Karyopherin subunit alpha 2 | DisGeNET |  |  |  |
| 731 | KRAS | KRAS Proto-Oncogene, GTPase | GeneCards |  |  |  |
| 732 | KRT16 | Keratin 16 | DisGeNET |  |  |  |
| 733 | LAD1 | Ladinin 1 | DisGeNET |  |  |  |
| 734 | LAMB2 | Laminin subunit beta 2 | DisGeNET |  |  |  |
| 735 | LAMP2 | Lysosomal Associated Membrane Protein 2 | GeneCards | DisGeNET |  |  |
| 736 | LARGE1 | LARGE xylosyl- and glucuronyltransferase 1 | DisGeNET |  |  |  |
| 737 | LCAT | Lecithin-Cholesterol Acyltransferase | GeneCards |  |  |  |
| 738 | LCK | LCK proto-oncogene, Src family tyrosine kinase | DisGeNET |  |  |  |
| 739 | LCN1 | Lipocalin 1 | DisGeNET |  |  |  |
| 740 | LCN2 | Lipocalin 2 | GeneCards | DisGeNET |  |  |
| 741 | LDLR | Low Density Lipoprotein Receptor | GeneCards |  |  |  |
| 742 | LEP | Leptin | GeneCards | DisGeNET |  |  |
| 743 | LEPR | Leptin Receptor | GeneCards | DisGeNET |  |  |
| 744 | LGALS1 | Galectin 1 | DisGeNET |  |  |  |
| 745 | LGALS3 | Galectin 3 | DisGeNET |  |  |  |
| 746 | LIMK2 | LIM domain kinase 2 | DisGeNET |  |  |  |
| 747 | LIN28A | Lin-28 homolog A | DisGeNET |  |  |  |
| 748 | LINC00462 | Long intergenic non-protein coding RNA 462 | DisGeNET |  |  |  |
| 749 | LINC00472 | Long intergenic non-protein coding RNA 472 | DisGeNET |  |  |  |
| 750 | LINC00968 | Long intergenic non-protein coding RNA 968 | DisGeNET |  |  |  |
| 751 | LINC01139 | Long intergenic non-protein coding RNA 1139 | DisGeNET |  |  |  |
| 752 | LINC01619 | Long intergenic non-protein coding RNA 1619 | DisGeNET |  |  |  |
| 753 | LIPC | Lipase C, Hepatic Type | GeneCards | DisGeNET | OMIM |  |
| 754 | LMNA | Lamin A/C | GeneCards | DisGeNET | OMIM |  |
| 755 | LMX1B | LIM Homeobox Transcription Factor 1 Beta | GeneCards |  |  |  |
| 756 | LOC102724334 | Histone H2B type F-S-like | DisGeNET |  |  |  |
| 757 | LOC105371049 | Uncharacterized LOC105371049 | GeneCards |  |  |  |
| 758 | LOC106627981 | GBA Recombination Region | GeneCards |  |  |  |
| 759 | LOH19CR1 | Loss of heterozygosity, 19, chromosomal region 1 | DisGeNET |  |  |  |
| 760 | LOXL2 | Lysyl oxidase like 2 | DisGeNET |  |  |  |
| 761 | LPA | Lipoprotein(A) | GeneCards | DisGeNET | OMIM |  |
| 762 | LPAR1 | Lysophosphatidic acid receptor 1 | DisGeNET |  |  |  |
| 763 | LPAR3 | Lysophosphatidic acid receptor 3 | DisGeNET |  |  |  |
| 764 | LPL | Lipoprotein Lipase | GeneCards | DisGeNET |  |  |
| 765 | LRG1 | Leucine rich alpha-2-glycoprotein 1 | DisGeNET |  |  |  |
| 766 | LRP2 | LDL Receptor Related Protein 2 | GeneCards | DisGeNET |  |  |
| 767 | LRP5 | LDL Receptor Related Protein 5 | GeneCards | DisGeNET |  |  |
| 768 | LRP6 | LDL receptor related protein 6 | DisGeNET |  |  |  |
| 769 | LRPAP1 | LDL receptor related protein associated protein 1 | DisGeNET |  |  |  |
| 770 | LRRC7 | Leucine rich repeat containing 7 | DisGeNET |  |  |  |
| 771 | LRRK2 | Leucine Rich Repeat Kinase 2 | GeneCards |  |  |  |
| 772 | LTA | Lymphotoxin alpha | DisGeNET |  |  |  |
| 773 | LTA4H | Leukotriene A-4 hydrolase | DrugBank |  |  |  |
| 774 | LTBP1 | Latent transforming growth factor beta binding protein 1 | DisGeNET |  |  |  |
| 775 | LYZ | Lysozyme | DisGeNET |  |  |  |
| 776 | MAFA | Transcription factor MafA | OMIM |  |  |  |
| 777 | MAK16 | MAK16 homolog | DisGeNET |  |  |  |
| 778 | MALAT1 | Metastasis associated lung adenocarcinoma transcript 1 | DisGeNET |  |  |  |
| 779 | MALT1 | MALT1 paracaspase | DisGeNET |  |  |  |
| 780 | MAP1LC3A | Microtubule associated protein 1 light chain 3 alpha | DisGeNET |  |  |  |
| 781 | MAP2K2 | Mitogen-activated protein kinase kinase 2 | DisGeNET |  |  |  |
| 782 | MAP2K7 | Mitogen-activated protein kinase kinase 7 | DisGeNET |  |  |  |
| 783 | MAP3K1 | Mitogen-activated protein kinase kinase kinase 1 | DisGeNET |  |  |  |
| 784 | MAP3K5 | mitogen-activated protein kinase kinase kinase 5 | TTD | DisGeNET |  |  |
| 785 | MAP3K7 | Mitogen-activated protein kinase kinase kinase 7 | DisGeNET |  |  |  |
| 786 | MAP6 | Microtubule associated protein 6 | DisGeNET |  |  |  |
| 787 | MAPK1 | Mitogen-Activated Protein Kinase 1 | GeneCards | DisGeNET |  |  |
| 788 | MAPK12 | MAP kinase p38 | TTD |  |  |  |
| 789 | MAPK14 | Mitogen-activated protein kinase 14 | DisGeNET |  |  |  |
| 790 | MAPK3 | Mitogen-activated protein kinase 3 | DisGeNET |  |  |  |
| 791 | MAPK8 | Mitogen-activated protein kinase 8 | DisGeNET |  |  |  |
| 792 | MAPK8IP1 | Mitogen-Activated Protein Kinase 8 Interacting Protein 1 | GeneCards | OMIM |  |  |
| 793 | MAPT | Microtubule Associated Protein Tau | GeneCards |  |  |  |
| 794 | MARCKSL1 | MARCKS like 1 | DisGeNET |  |  |  |
| 795 | MARK2 | Microtubule affinity regulating kinase 2 | DisGeNET |  |  |  |
| 796 | MARS1 | Methionyl-TRNA Synthetase 1 | GeneCards |  |  |  |
| 797 | MASP2 | Mannan binding lectin serine peptidase 2 | DisGeNET |  |  |  |
| 798 | MB | Myoglobin | DisGeNET |  |  |  |
| 799 | MBL2 | Mannose binding lectin 2 | DisGeNET |  |  |  |
| 800 | MBL3P | Mannose-binding lectin family member 3, pseudogene | DisGeNET |  |  |  |
| 801 | MBNL2 | Muscleblind like splicing regulator 2 | DisGeNET |  |  |  |
| 802 | MC4R | Melanocortin 4 Receptor | GeneCards |  |  |  |
| 803 | MCAM | Melanoma cell adhesion molecule | DisGeNET |  |  |  |
| 804 | MCF2L2 | MCF.2 cell line derived transforming sequence-like 2 | DisGeNET |  |  |  |
| 805 | MCM3 | Minichromosome maintenance complex component 3 | DisGeNET |  |  |  |
| 806 | MCS+9.7 | RET intron 1 enhancer | DisGeNET |  |  |  |
| 807 | MDK | Midkine | DisGeNET |  |  |  |
| 808 | MDM2 | MDM2 proto-oncogene | DisGeNET |  |  |  |
| 809 | MEFV | MEFV Innate Immuity Regulator, Pyrin | GeneCards | DisGeNET |  |  |
| 810 | MEG3 | Maternally expressed 3 | DisGeNET |  |  |  |
| 811 | MEN1 | Menin 1 | GeneCards |  |  |  |
| 812 | MEP1B | Meprin A subunit beta | DisGeNET |  |  |  |
| 813 | MET | MET Proto-Oncogene, Receptor Tyrosine Kinase | GeneCards |  |  |  |
| 814 | MFAP1 | Microfibril associated protein 1 | DisGeNET |  |  |  |
| 815 | MFHAS1 | Malignant fibrous histiocytoma amplified sequence 1 | DisGeNET |  |  |  |
| 816 | MFN2 | Mitofusin 2 | GeneCards | DisGeNET | OMIM |  |
| 817 | MFT2 | Trichoepithelioma, multiple familial, 2 | DisGeNET |  |  |  |
| 818 | MGAM | Maltase-glucoamylase | DisGeNET |  |  |  |
| 819 | MGP | Matrix Gla protein | DisGeNET |  |  |  |
| 820 | MIA3 | Transport and Golgi organization protein 1 homolog | OMIM |  |  |  |
| 821 | MICOS10-NBL1 | MICOS10-NBL1 readthrough | DisGeNET |  |  |  |
| 822 | MIF | Macrophage migration inhibitory factor | DisGeNET |  |  |  |
| 823 | MINDY4 | MINDY lysine 48 deubiquitinase 4 | DisGeNET |  |  |  |
| 824 | MIOX | Myo-inositol oxygenase | DisGeNET |  |  |  |
| 825 | MIR1207 | MicroRNA 1207 | DisGeNET |  |  |  |
| 826 | MIR122 | MicroRNA 122 | GeneCards |  |  |  |
| 827 | MIR125A | MicroRNA 125a | DisGeNET |  |  |  |
| 828 | MIR126 | MicroRNA 126 | GeneCards | DisGeNET |  |  |
| 829 | MIR130B | MicroRNA 130b | DisGeNET |  |  |  |
| 830 | MIR132 | MicroRNA 132 | GeneCards | DisGeNET |  |  |
| 831 | MIR133B | MicroRNA 133b | DisGeNET |  |  |  |
| 832 | MIR134 | MicroRNA 134 | DisGeNET |  |  |  |
| 833 | MIR137 | MicroRNA 137 | DisGeNET |  |  |  |
| 834 | MIR140 | MicroRNA 140 | GeneCards |  |  |  |
| 835 | MIR141 | MicroRNA 141 | DisGeNET |  |  |  |
| 836 | MIR143 | MicroRNA 143 | GeneCards |  |  |  |
| 837 | MIR145 | MicroRNA 145 | GeneCards | DisGeNET |  |  |
| 838 | MIR146A | MicroRNA 146a | GeneCards | DisGeNET |  |  |
| 839 | MIR150 | MicroRNA 150 | DisGeNET |  |  |  |
| 840 | MIR152 | MicroRNA 152 | DisGeNET |  |  |  |
| 841 | MIR154 | MicroRNA 154 | DisGeNET |  |  |  |
| 842 | MIR155 | MicroRNA 155 | GeneCards | DisGeNET |  |  |
| 843 | MIR15A | MicroRNA 15a | GeneCards | DisGeNET |  |  |
| 844 | MIR15B | MicroRNA 15b | DisGeNET |  |  |  |
| 845 | MIR17 | MicroRNA 17 | GeneCards |  |  |  |
| 846 | MIR181A2 | MicroRNA 181a-2 | DisGeNET |  |  |  |
| 847 | MIR182 | MicroRNA 182 | DisGeNET |  |  |  |
| 848 | MIR184 | MicroRNA 184 | DisGeNET |  |  |  |
| 849 | MIR188 | MicroRNA 188 | DisGeNET |  |  |  |
| 850 | MIR18B | MicroRNA 18b | DisGeNET |  |  |  |
| 851 | MIR192 | MicroRNA 192 | GeneCards | DisGeNET |  |  |
| 852 | MIR199B | MicroRNA 199b | DisGeNET |  |  |  |
| 853 | MIR200B | MicroRNA 200b | DisGeNET |  |  |  |
| 854 | MIR200C | MicroRNA 200c | DisGeNET |  |  |  |
| 855 | MIR203A | MicroRNA 203a | DisGeNET |  |  |  |
| 856 | MIR206 | MicroRNA 206 | DisGeNET |  |  |  |
| 857 | MIR20A | MicroRNA 20a | DisGeNET |  |  |  |
| 858 | MIR20B | MicroRNA 20b | DisGeNET |  |  |  |
| 859 | MIR21 | MicroRNA 21 | GeneCards | DisGeNET |  |  |
| 860 | MIR210 | MicroRNA 210 | DisGeNET |  |  |  |
| 861 | MIR214 | MicroRNA 214 | DisGeNET |  |  |  |
| 862 | MIR217 | MicroRNA 217 | DisGeNET |  |  |  |
| 863 | MIR22 | MicroRNA 22 | DisGeNET |  |  |  |
| 864 | MIR221 | MicroRNA 221 | DisGeNET |  |  |  |
| 865 | MIR223 | MicroRNA 223 | GeneCards | DisGeNET |  |  |
| 866 | MIR23A | MicroRNA 23a | DisGeNET |  |  |  |
| 867 | MIR23B | MicroRNA 23b | DisGeNET |  |  |  |
| 868 | MIR23C | MicroRNA 23c | DisGeNET |  |  |  |
| 869 | MIR25 | MicroRNA 25 | DisGeNET |  |  |  |
| 870 | MIR27A | MicroRNA 27a | DisGeNET |  |  |  |
| 871 | MIR27B | MicroRNA 27b | DisGeNET |  |  |  |
| 872 | MIR29A | MicroRNA 29a | GeneCards | DisGeNET |  |  |
| 873 | MIR29B1 | MicroRNA 29b-1 | DisGeNET |  |  |  |
| 874 | MIR29B2 | MicroRNA 29b-2 | DisGeNET |  |  |  |
| 875 | MIR29C | MicroRNA 29c | GeneCards | DisGeNET |  |  |
| 876 | MIR302A | MicroRNA 302a | DisGeNET |  |  |  |
| 877 | MIR30A | MicroRNA 30a | DisGeNET |  |  |  |
| 878 | MIR30B | MicroRNA 30b | DisGeNET |  |  |  |
| 879 | MIR30C1 | MicroRNA 30c-1 | DisGeNET |  |  |  |
| 880 | MIR30C2 | MicroRNA 30c-2 | DisGeNET |  |  |  |
| 881 | MIR30E | MicroRNA 30e | DisGeNET |  |  |  |
| 882 | MIR31 | MicroRNA 31 | DisGeNET |  |  |  |
| 883 | MIR3196 | MicroRNA 3196 | DisGeNET |  |  |  |
| 884 | MIR320A | MicroRNA 320a | DisGeNET |  |  |  |
| 885 | MIR320E | MicroRNA 320e | DisGeNET |  |  |  |
| 886 | MIR33A | MicroRNA 33a | DisGeNET |  |  |  |
| 887 | MIR342 | MicroRNA 342 | DisGeNET |  |  |  |
| 888 | MIR34A | MicroRNA 34a | GeneCards | DisGeNET |  |  |
| 889 | MIR34B | MicroRNA 34b | DisGeNET |  |  |  |
| 890 | MIR374A | MicroRNA 374a | DisGeNET |  |  |  |
| 891 | MIR377 | MicroRNA 377 | DisGeNET |  |  |  |
| 892 | MIR378A | MicroRNA 378a | DisGeNET |  |  |  |
| 893 | MIR379 | MicroRNA 379 | DisGeNET |  |  |  |
| 894 | MIR382 | MicroRNA 382 | DisGeNET |  |  |  |
| 895 | MIR424 | MicroRNA 424 | DisGeNET |  |  |  |
| 896 | MIR4490 | MicroRNA 4490 | DisGeNET |  |  |  |
| 897 | MIR449A | MicroRNA 449a | DisGeNET |  |  |  |
| 898 | MIR449B | MicroRNA 449b | DisGeNET |  |  |  |
| 899 | MIR451A | MicroRNA 451a | DisGeNET |  |  |  |
| 900 | MIR455 | MicroRNA 455 | DisGeNET |  |  |  |
| 901 | MIR4756 | MicroRNA 4756 | DisGeNET |  |  |  |
| 902 | MIR485 | MicroRNA 485 | DisGeNET |  |  |  |
| 903 | MIR486-1 | MicroRNA 486-1 | DisGeNET |  |  |  |
| 904 | MIR488 | MicroRNA 488 | DisGeNET |  |  |  |
| 905 | MIR497 | MicroRNA 497 | DisGeNET |  |  |  |
| 906 | MIR499A | MicroRNA 499a | DisGeNET |  |  |  |
| 907 | MIR503 | MicroRNA 503 | DisGeNET |  |  |  |
| 908 | MIR544A | MicroRNA 544a | DisGeNET |  |  |  |
| 909 | MIR675 | MicroRNA 675 | DisGeNET |  |  |  |
| 910 | MIR770 | MicroRNA 770 | DisGeNET |  |  |  |
| 911 | MIR874 | MicroRNA 874 | DisGeNET |  |  |  |
| 912 | MIR93 | MicroRNA 93 | DisGeNET |  |  |  |
| 913 | MIR96 | MicroRNA 96 | DisGeNET |  |  |  |
| 914 | MIR98 | MicroRNA 98 | DisGeNET |  |  |  |
| 915 | MIR99B | MicroRNA 99b | DisGeNET |  |  |  |
| 916 | MIRLET7A2 | MicroRNA let-7a-2 | DisGeNET |  |  |  |
| 917 | MIRLET7A3 | MicroRNA let-7a-3 | DisGeNET |  |  |  |
| 918 | MIRLET7B | MicroRNA let-7b | DisGeNET |  |  |  |
| 919 | MIRLET7C | MicroRNA let-7c | DisGeNET |  |  |  |
| 920 | MIXL1 | Mix paired-like homeobox | DisGeNET |  |  |  |
| 921 | MKKS | MKKS Centrosomal Shuttling Protein | GeneCards |  |  |  |
| 922 | MKS1 | MKS Transition Zone Complex Subunit 1 | GeneCards |  |  |  |
| 923 | MLLT3 | MLLT3 super elongation complex subunit | DisGeNET |  |  |  |
| 924 | MLN | Motilin | DisGeNET |  |  |  |
| 925 | MLXIPL | MLX interacting protein like | DisGeNET |  |  |  |
| 926 | MME | Membrane Metalloendopeptidase | GeneCards | DisGeNET | OMIM |  |
| 927 | MMP1 | Matrix Metallopeptidase 1 | GeneCards | DisGeNET |  |  |
| 928 | MMP-1 | Matrix metalloproteinase-1 | TTD |  |  |  |
| 929 | MMP10 | Matrix metallopeptidase 10 | DisGeNET |  |  |  |
| 930 | MMP12 | Matrix metallopeptidase 12 | DisGeNET |  |  |  |
| 931 | MMP2 | Matrix Metallopeptidase 2 | GeneCards | DisGeNET | DrugBank |  |
| 932 | MMP3 | Matrix Metallopeptidase 3 | GeneCards | DisGeNET |  |  |
| 933 | MMP7 | Matrix metallopeptidase 7 | DisGeNET |  |  |  |
| 934 | MMP8 | Matrix metallopeptidase 8 | DisGeNET |  |  |  |
| 935 | MMP9 | Matrix Metallopeptidase 9 | GeneCards | DisGeNET | DrugBank |  |
| 936 | MMRN1 | Multimerin 1 | DisGeNET |  |  |  |
| 937 | MOK | MOK protein kinase | DisGeNET |  |  |  |
| 938 | MPO | Myeloperoxidase | GeneCards | DisGeNET |  |  |
| 939 | MPRIP | Myosin phosphatase Rho interacting protein | DisGeNET |  |  |  |
| 940 | MPV17 | Protein Mpv17 | OMIM |  |  |  |
| 941 | MPZ | Myelin Protein Zero | GeneCards | OMIM |  |  |
| 942 | MR | Mineralocorticoid recepto**r** | TTD |  |  |  |
| 943 | MRAS | Muscle RAS oncogene homolog | DisGeNET |  |  |  |
| 944 | MRTFA | Myocardin related transcription factor A | DisGeNET |  |  |  |
| 945 | MSC | Musculin | DisGeNET |  |  |  |
| 946 | MSN | Moesin | DisGeNET |  |  |  |
| 947 | MSX2 | Msh homeobox 2 | DisGeNET |  |  |  |
| 948 | MT-ATP6 | Mitochondrially Encoded ATP Synthase Membrane Subunit 6 | GeneCards |  |  |  |
| 949 | MT-CO1 | Mitochondrially Encoded Cytochrome C Oxidase I | GeneCards |  |  |  |
| 950 | MTCO2P12 | MT-CO2 pseudogene 12 | DisGeNET |  |  |  |
| 951 | MTDH | Metadherin | DisGeNET |  |  |  |
| 952 | MTHFR | Methylenetetrahydrofolate Reductase | GeneCards | DisGeNET | DrugBank |  |
| 953 | MTHFS | Methenyltetrahydrofolate synthetase | DisGeNET |  |  |  |
| 954 | MTMR2 | Myotubularin Related Protein 2 | GeneCards |  |  |  |
| 955 | MT-ND1 | Mitochondrially Encoded NADH:Ubiquinone Oxidoreductase Core Subunit 1 | GeneCards |  |  |  |
| 956 | MT-ND4 | Mitochondrially Encoded NADH:Ubiquinone Oxidoreductase Core Subunit 4 | GeneCards |  |  |  |
| 957 | MT-ND5 | Mitochondrially Encoded NADH:Ubiquinone Oxidoreductase Core Subunit 5 | GeneCards |  |  |  |
| 958 | MT-ND6 | Mitochondrially Encoded NADH:Ubiquinone Oxidoreductase Core Subunit 6 | GeneCards |  |  |  |
| 959 | MTNR1B | Melatonin Receptor 1B | GeneCards | OMIM |  |  |
| 960 | MTOR | Mechanistic Target Of Rapamycin Kinase | GeneCards | DisGeNET |  |  |
| 961 | MT-TE | Mitochondrially Encoded TRNA-Glu (GAA/G) | GeneCards |  |  |  |
| 962 | MT-TK | Mitochondrially Encoded TRNA-Lys (AAA/G) | GeneCards |  |  |  |
| 963 | MT-TL1 | Mitochondrially Encoded TRNA-Leu (UUA/G) 1 | GeneCards |  |  |  |
| 964 | MUC1 | Mucin 1, Cell Surface Associated | GeneCards | OMIM |  |  |
| 965 | MYC | MYC Proto-Oncogene, BHLH Transcription Factor | GeneCards |  |  |  |
| 966 | MYCN | MYCN proto-oncogene, bHLH transcription factor | DisGeNET |  |  |  |
| 967 | MYD88 | MYD88 innate immune signal transduction adaptor | DisGeNET |  |  |  |
| 968 | MYH2 | Myosin heavy chain 2 | DisGeNET |  |  |  |
| 969 | MYH9 | Myosin Heavy Chain 9 | GeneCards | DisGeNET |  |  |
| 970 | MYOCD | Myocardin | DisGeNET |  |  |  |
| 971 | MZF1 | Myeloid zinc finger 1 | DisGeNET |  |  |  |
| 972 | NAGLU | N-Acetyl-Alpha-Glucosaminidase | GeneCards |  |  |  |
| 973 | NAT2 | N-acetyltransferase 2 | DisGeNET |  |  |  |
| 974 | NAV3 | Neuron navigator 3 | DisGeNET |  |  |  |
| 975 | NBL1 | NBL1, DAN family BMP antagonist | DisGeNET |  |  |  |
| 976 | NCALD | Neurocalcin delta | DisGeNET |  |  |  |
| 977 | NCF1 | Neutrophil cytosolic factor 1 | DisGeNET | OMIM |  |  |
| 978 | NCF2 | Neutrophil Cytosolic Factor 2 | GeneCards | OMIM |  |  |
| 979 | NCK1 | NCK adaptor protein 1 | DisGeNET |  |  |  |
| 980 | NCL | Nucleolin | DisGeNET |  |  |  |
| 981 | NDN | Necdin | DisGeNET |  |  |  |
| 982 | NDRG1 | N-Myc Downstream Regulated 1 | GeneCards |  |  |  |
| 983 | NDUFS3 | NADH:ubiquinone oxidoreductase core subunit S3 | DisGeNET |  |  |  |
| 984 | NEAT1 | Nuclear paraspeckle assembly transcript 1 | DisGeNET |  |  |  |
| 985 | NEFL | Neurofilament Light Chain | GeneCards |  |  |  |
| 986 | NEK1 | NIMA Related Kinase 1 | GeneCards |  |  |  |
| 987 | NEK8 | NIMA Related Kinase 8 | GeneCards |  |  |  |
| 988 | NEUROD1 | Neuronal Differentiation 1 | GeneCards | OMIM |  |  |
| 989 | NF1 | Neurofibromin 1 | GeneCards |  |  |  |
| 990 | NFAT5 | Nuclear factor of activated T cells 5 | DisGeNET |  |  |  |
| 991 | NFE2L2 | Nuclear factor, erythroid 2 like 2 | DisGeNET |  |  |  |
| 992 | NFIA | Nuclear factor I A | DisGeNET |  |  |  |
| 993 | NFIB | Nuclear factor I B | DisGeNET |  |  |  |
| 994 | NFIC | Nuclear factor I C | DisGeNET |  |  |  |
| 995 | NFIX | Nuclear factor I X | DisGeNET |  |  |  |
| 996 | NFKB1 | Nuclear Factor Kappa B Subunit 1 | GeneCards | DisGeNET |  |  |
| 997 | NFKBIA | NFKB inhibitor alpha | DisGeNET |  |  |  |
| 998 | NLRC4 | NLR family CARD domain containing 4 | DisGeNET |  |  |  |
| 999 | NLRC5 | NLR family CARD domain containing 5 | DisGeNET |  |  |  |
| 1000 | NLRP1 | NLR family pyrin domain containing 1 | DisGeNET |  |  |  |
| 1001 | NLRP3 | NLR Family Pyrin Domain Containing 3 | GeneCards | DisGeNET |  |  |
| 1002 | NLRX1 | NLR family member X1 | DisGeNET |  |  |  |
| 1003 | NMUR2 | Neuromedin U receptor 2 | DisGeNET |  |  |  |
| 1004 | NOD2 | Nucleotide Binding Oligomerization Domain Containing 2 | GeneCards | DisGeNET |  |  |
| 1005 | NORAD | Non-coding RNA activated by DNA damage | DisGeNET |  |  |  |
| 1006 | NOS1 | Nitric oxide synthase 1 | DisGeNET |  |  |  |
| 1007 | NOS2 | Nitric-oxide synthase inducible | TTD | GeneCards | DisGeNET |  |
| 1008 | NOS3 | Nitric Oxide Synthase 3 | GeneCards | DisGeNET | OMIM |  |
| 1009 | NOTCH1 | Notch Receptor 1 | GeneCards | DisGeNET |  |  |
| 1010 | NOTCH2 | Notch Receptor 2 | GeneCards | DisGeNET |  |  |
| 1011 | NOTCH2NLC | Notch homolog 2 N-terminal-like protein C | OMIM |  |  |  |
| 1012 | NOTCH3 | Notch receptor 3 | DisGeNET |  |  |  |
| 1013 | NOX1 | NADPH oxidase 1 | DisGeNET |  |  |  |
| 1014 | NOX3 | NADPH oxidase 3 | DisGeNET |  |  |  |
| 1015 | NOX4 | NADPH oxidase 4 | DisGeNET |  |  |  |
| 1016 | NOX5 | NADPH oxidase 5 | DisGeNET |  |  |  |
| 1017 | NPC1 | NPC Intracellular Cholesterol Transporter 1 | GeneCards |  |  |  |
| 1018 | NPC2 | NPC Intracellular Cholesterol Transporter 2 | GeneCards |  |  |  |
| 1019 | NPHP1 | Nephrocystin 1 | GeneCards |  |  |  |
| 1020 | NPHP3 | Nephrocystin 3 | GeneCards |  |  |  |
| 1021 | NPHP4 | Nephrocystin 4 | GeneCards |  |  |  |
| 1022 | NPHS1 | NPHS1 Adhesion Molecule, Nephrin | GeneCards | DisGeNET |  |  |
| 1023 | NPHS2 | NPHS2 Stomatin Family Member, Podocin | GeneCards | DisGeNET |  |  |
| 1024 | NPPA | Natriuretic Peptide A | GeneCards | DisGeNET |  |  |
| 1025 | NPPB | Natriuretic Peptide B | GeneCards | DisGeNET |  |  |
| 1026 | NPY | Neuropeptide Y | DisGeNET |  |  |  |
| 1027 | NQO1 | NAD(P)H quinone dehydrogenase 1 | DisGeNET |  |  |  |
| 1028 | NR0B2 | Nuclear receptor subfamily 0 group B member 2 | DisGeNET |  |  |  |
| 1029 | NR1H3 | Nuclear receptor subfamily 1 group H member 3 | DisGeNET |  |  |  |
| 1030 | NR1H4 | Nuclear receptor subfamily 1 group H member 4 | DisGeNET |  |  |  |
| 1031 | NR1I2 | Nuclear receptor subfamily 1 group I member 2 | DisGeNET |  |  |  |
| 1032 | NR2C2 | Nuclear receptor subfamily 2 group C member 2 | DisGeNET |  |  |  |
| 1033 | NR3C2 | Nuclear receptor subfamily 3 group C member 2 | DisGeNET |  |  |  |
| 1034 | NR4A1 | Nuclear receptor subfamily 4 group A member 1 | DisGeNET |  |  |  |
| 1035 | NR5A1 | Nuclear receptor subfamily 5 group A member 1 | DisGeNET |  |  |  |
| 1036 | NRG4 | Neuregulin 4 | DisGeNET |  |  |  |
| 1037 | NRK | Nik related kinase | DisGeNET |  |  |  |
| 1038 | NSA2 | NSA2 ribosome biogenesis factor | DisGeNET |  |  |  |
| 1039 | NTN1 | Netrin 1 | DisGeNET |  |  |  |
| 1040 | NTRK1 | Neurotrophic receptor tyrosine kinase 1 | DisGeNET |  |  |  |
| 1041 | NTRK2 | Neurotrophic receptor tyrosine kinase 2 | DisGeNET |  |  |  |
| 1042 | NTT | Noncoding transcript in T cells | DisGeNET |  |  |  |
| 1043 | NUAK1 | NUAK family kinase 1 | DisGeNET |  |  |  |
| 1044 | NUP62 | Nucleoporin 62 | DisGeNET |  |  |  |
| 1045 | OCRL | OCRL Inositol Polyphosphate-5-Phosphatase | GeneCards |  |  |  |
| 1046 | OFD1 | OFD1 Centriole And Centriolar Satellite Protein | GeneCards |  |  |  |
| 1047 | OGA | O-GlcNAcase | DisGeNET |  |  |  |
| 1048 | OGN | Osteoglycin | DisGeNET |  |  |  |
| 1049 | OLR1 | Oxidized low density lipoprotein receptor 1 | DisGeNET |  |  |  |
| 1050 | OPTN | Optineurin | DisGeNET |  |  |  |
| 1051 | OR10A4 | Olfactory receptor family 10 subfamily A member 4 | DisGeNET |  |  |  |
| 1052 | ORAI1 | ORAI calcium release-activated calcium modulator 1 | DisGeNET |  |  |  |
| 1053 | ORM1 | Alpha-1-acid glycoprotein 1 | DrugBank |  |  |  |
| 1054 | P2RX7 | Purinergic receptor P2X 7 | DisGeNET |  |  |  |
| 1055 | PACSIN2 | Protein kinase C and casein kinase substrate in neurons 2 | DisGeNET |  |  |  |
| 1056 | PAEP | Progestagen associated endometrial protein | DisGeNET |  |  |  |
| 1057 | PALD1 | Phosphatase domain containing paladin 1 | DisGeNET |  |  |  |
| 1058 | PALLD | Palladin | DisGeNET |  |  |  |
| 1059 | PAQR3 | Progestin and adipoQ receptor family member 3 | DisGeNET |  |  |  |
| 1060 | PARK7 | Parkinsonism Associated Deglycase | GeneCards |  |  |  |
| 1061 | PARN | Poly(A)-specific ribonuclease | DisGeNET |  |  |  |
| 1062 | PARP1 | Poly(ADP-ribose) polymerase 1 | DisGeNET |  |  |  |
| 1063 | PAX2 | Paired Box 2 | GeneCards | DisGeNET |  |  |
| 1064 | PAX4 | Paired Box 4 | GeneCards | OMIM |  |  |
| 1065 | PAX6 | Paired Box 6 | GeneCards |  |  |  |
| 1066 | PBX1 | PBX Homeobox 1 | GeneCards | OMIM |  |  |
| 1067 | PCSK1 | Proprotein convertase subtilisin/kexin type 1 | DisGeNET |  |  |  |
| 1068 | PDCD1 | Programmed Cell Death 1 | GeneCards | OMIM |  |  |
| 1069 | PDE11A | Dual 3',5'-cyclic-AMP and -GMP phosphodiesterase 11A | OMIM |  |  |  |
| 1070 | PDE3A | Phosphodiesterase 3A | DisGeNET |  |  |  |
| 1071 | PDE5A | Phosphodiesterase 5A | DisGeNET |  |  |  |
| 1072 | PDE8B | High affinity cAMP-specific and IBMX-insensitive 3',5'-cyclic phosphodiesterase 8B | OMIM |  |  |  |
| 1073 | PDGFA | Platelet derived growth factor subunit A | DisGeNET |  |  |  |
| 1074 | PDGFRA | Platelet derived growth factor receptor alpha | DisGeNET |  |  |  |
| 1075 | PDGFRB | Platelet derived growth factor receptor beta | DisGeNET |  |  |  |
| 1076 | PDK1 | Pyruvate dehydrogenase kinase 1 | DisGeNET |  |  |  |
| 1077 | PDPN | Podoplanin | DisGeNET |  |  |  |
| 1078 | PDX1 | Pancreatic And Duodenal Homeobox 1 | GeneCards | OMIM |  |  |
| 1079 | PEA15 | Proliferation and apoptosis adaptor protein 15 | DisGeNET |  |  |  |
| 1080 | PEBP1 | Phosphatidylethanolamine binding protein 1 | DisGeNET |  |  |  |
| 1081 | PECAM1 | Platelet and endothelial cell adhesion molecule 1 | DisGeNET |  |  |  |
| 1082 | PES1 | Pescadillo ribosomal biogenesis factor 1 | DisGeNET |  |  |  |
| 1083 | PEX6 | Peroxisomal Biogenesis Factor 6 | GeneCards |  |  |  |
| 1084 | PGAM2 | Phosphoglycerate mutase 2 | OMIM |  |  |  |
| 1085 | PGC | Progastricsin | DisGeNET |  |  |  |
| 1086 | PGR-AS1 | PGR antisense RNA 1 | DisGeNET |  |  |  |
| 1087 | PHLPP1 | PH domain and leucine rich repeat protein phosphatase 1 | DisGeNET |  |  |  |
| 1088 | PHOX2B | Paired mesoderm homeobox protein 2B | OMIM |  |  |  |
| 1089 | PIAS4 | Protein inhibitor of activated STAT 4 | DisGeNET |  |  |  |
| 1090 | PICK1 | Protein interacting with PRKCA 1 | DisGeNET |  |  |  |
| 1091 | PIK3C2B | Phosphatidylinositol-4-phosphate 3-kinase catalytic subunit type 2 beta | DisGeNET |  |  |  |
| 1092 | PIK3CA | Phosphatidylinositol-4,5-Bisphosphate 3-Kinase Catalytic Subunit Alpha | GeneCards | DisGeNET |  |  |
| 1093 | PIK3CB | Phosphatidylinositol-4,5-bisphosphate 3-kinase catalytic subunit beta | DisGeNET |  |  |  |
| 1094 | PIK3CD | Phosphatidylinositol-4,5-bisphosphate 3-kinase catalytic subunit delta | DisGeNET |  |  |  |
| 1095 | PIK3CG | Phosphatidylinositol-4,5-bisphosphate 3-kinase catalytic subunit gamma | DisGeNET |  |  |  |
| 1096 | PIK3R1 | Phosphoinositide-3-Kinase Regulatory Subunit 1 | GeneCards | DisGeNET |  |  |
| 1097 | PIK3R2 | Phosphoinositide-3-kinase regulatory subunit 2 | DisGeNET |  |  |  |
| 1098 | PIN1 | Peptidylprolyl cis/trans isomerase, NIMA-interacting 1 | DisGeNET |  |  |  |
| 1099 | PINK1 | PTEN Induced Kinase 1 | GeneCards | OMIM |  |  |
| 1100 | PITX2 | Paired like homeodomain 2 | DisGeNET |  |  |  |
| 1101 | PKD1 | Polycystin 1, Transient Receptor Potential Channel Interacting | GeneCards | DisGeNET |  |  |
| 1102 | PKD2 | Polycystin 2, Transient Receptor Potential Cation Channel | GeneCards | OMIM |  |  |
| 1103 | PKD2L1 | Polycystin 2 Like 1, Transient Receptor Potential Cation Channel | GeneCards |  |  |  |
| 1104 | PKHD1 | Fibrocystin | GeneCards | OMIM |  |  |
| 1105 | PKM | Pyruvate kinase M1/2 | DisGeNET |  |  |  |
| 1106 | PLA2G15 | Phospholipase A2 group XV | DisGeNET |  |  |  |
| 1107 | PLA2G1B | Phospholipase A2 group IB | DisGeNET |  |  |  |
| 1108 | PLA2G6 | Phospholipase A2 Group VI | GeneCards |  |  |  |
| 1109 | PLA2G7 | Phospholipase A2 group VII | DisGeNET |  |  |  |
| 1110 | PLA2R1 | Phospholipase A2 receptor 1 | DisGeNET |  |  |  |
| 1111 | PLAGL1 | PLAG1 Like Zinc Finger 1 | GeneCards |  |  |  |
| 1112 | PLAU | Plasminogen activator, urokinase | DisGeNET |  |  |  |
| 1113 | PLB1 | Phospholipase B1 | DisGeNET |  |  |  |
| 1114 | PLEKHG5 | Pleckstrin homology domain-containing family G member 5 | OMIM |  |  |  |
| 1115 | PLEKHH2 | Pleckstrin homology, MyTH4 and FERM domain containing H2 | DisGeNET |  |  |  |
| 1116 | PLEKHO1 | Pleckstrin homology domain containing O1 | DisGeNET |  |  |  |
| 1117 | PLG | Plasminogen | DisGeNET |  |  |  |
| 1118 | PLIN2 | Perilipin 2 | DisGeNET |  |  |  |
| 1119 | PLK2 | Polo like kinase 2 | DisGeNET |  |  |  |
| 1120 | PMM2 | Phosphomannomutase 2 | GeneCards |  |  |  |
| 1121 | PMP22 | Peripheral Myelin Protein 22 | GeneCards |  |  |  |
| 1122 | PNO1 | Partner of NOB1 homolog | DisGeNET |  |  |  |
| 1123 | PNPLA2 | Patatin like phospholipase domain containing 2 | DisGeNET |  |  |  |
| 1124 | POGLUT1 | Protein O-glucosyltransferase 1 | OMIM |  |  |  |
| 1125 | POLDIP2 | DNA polymerase delta interacting protein 2 | DisGeNET |  |  |  |
| 1126 | POLG | DNA Polymerase Gamma, Catalytic Subunit | GeneCards |  |  |  |
| 1127 | POMC | Proopiomelanocortin | GeneCards |  |  |  |
| 1128 | PON1 | Paraoxonase 1 | GeneCards | DisGeNET | OMIM |  |
| 1129 | PON2 | Paraoxonase 2 | GeneCards | DisGeNET | OMIM |  |
| 1130 | POSTN | Periostin | DisGeNET |  |  |  |
| 1131 | POTEF | POTE ankyrin domain family member F | DisGeNET |  |  |  |
| 1132 | POU2F3 | POU class 2 homeobox 3 | DisGeNET |  |  |  |
| 1133 | PPARA | Peroxisome Proliferator Activated Receptor Alpha | GeneCards | DisGeNET |  |  |
| 1134 | PPARD | Peroxisome proliferator activated receptor delta | DisGeNET |  |  |  |
| 1135 | PPARG | Peroxisome Proliferator Activated Receptor Gamma | GeneCards | DisGeNET | DrugBank | OMIM |
| 1136 | PPARGC1A | PPARG Coactivator 1 Alpha | GeneCards | DisGeNET |  |  |
| 1137 | PPBP | Pro-platelet basic protein | DisGeNET |  |  |  |
| 1138 | PPIA | Peptidylprolyl isomerase A | DisGeNET |  |  |  |
| 1139 | PPIG | Peptidylprolyl isomerase G | DisGeNET |  |  |  |
| 1140 | PPM1K | Protein phosphatase 1K, mitochondrial | OMIM |  |  |  |
| 1141 | PPP1R3A | Protein Phosphatase 1 Regulatory Subunit 3A | GeneCards |  |  |  |
| 1142 | PPP1R8 | Protein phosphatase 1 regulatory subunit 8 | DisGeNET |  |  |  |
| 1143 | PRKAA1 | Protein kinase AMP-activated catalytic subunit alpha 1 | DisGeNET |  |  |  |
| 1144 | PRKAA2 | Protein kinase AMP-activated catalytic subunit alpha 2 | DisGeNET |  |  |  |
| 1145 | PRKAB1 | Protein kinase AMP-activated non-catalytic subunit beta 1 | DisGeNET |  |  |  |
| 1146 | PRKAG2 | Protein Kinase AMP-Activated Non-Catalytic Subunit Gamma 2 | GeneCards | OMIM |  |  |
| 1147 | PRKAR1A | Protein Kinase CAMP-Dependent Type I Regulatory Subunit Alpha | GeneCards |  |  |  |
| 1148 | PRKCA | Protein kinase C alpha | DisGeNET |  |  |  |
| 1149 | PRKCB | Protein kinase C beta | DisGeNET |  |  |  |
| 1150 | PRKCD | Protein Kinase C Delta | GeneCards |  |  |  |
| 1151 | PRKCE | Protein kinase C epsilon | DisGeNET |  |  |  |
| 1152 | PRKCSH | Protein Kinase C Substrate 80K-H | GeneCards | DisGeNET |  |  |
| 1153 | PRKN | Parkin RBR E3 Ubiquitin Protein Ligase | GeneCards | OMIM |  |  |
| 1154 | PRMT1 | Protein arginine methyltransferase 1 | DisGeNET |  |  |  |
| 1155 | PRMT7 | Protein Arginine Methyltransferase 7 | GeneCards |  |  |  |
| 1156 | PRNP | Prion Protein | GeneCards |  |  |  |
| 1157 | PROC | Protein C, inactivator of coagulation factors Va and VIIIa | DisGeNET |  |  |  |
| 1158 | PROM1 | Prominin 1 | GeneCards | OMIM |  |  |
| 1159 | PROS1 | Protein S | DisGeNET |  |  |  |
| 1160 | PRPS1 | Phosphoribosyl Pyrophosphate Synthetase 1 | GeneCards |  |  |  |
| 1161 | PRSS1 | Serine protease 1 | DisGeNET |  |  |  |
| 1162 | PRSS2 | Serine protease 2 | DisGeNET |  |  |  |
| 1163 | PRSS55 | Serine protease 55 | DisGeNET |  |  |  |
| 1164 | PRTN3 | Proteinase 3 | GeneCards |  |  |  |
| 1165 | PRX | Periaxin | GeneCards |  |  |  |
| 1166 | PSAP | Prosaposin | GeneCards |  |  |  |
| 1167 | PSEN1 | Presenilin 1 | GeneCards |  |  |  |
| 1168 | PSEN2 | Presenilin 2 | GeneCards | OMIM |  |  |
| 1169 | PSMA6 | Proteasome 20S subunit alpha 6 | DisGeNET |  |  |  |
| 1170 | PSMD9 | Proteasome 26S subunit, non-ATPase 9 | DisGeNET |  |  |  |
| 1171 | PTAFR | Platelet activating factor receptor | DisGeNET |  |  |  |
| 1172 | PTBP1 | Polypyrimidine tract binding protein 1 | DisGeNET |  |  |  |
| 1173 | PTEN | Phosphatase And Tensin Homolog | GeneCards | DisGeNET |  |  |
| 1174 | PTF1A | Pancreas Associated Transcription Factor 1a | GeneCards |  |  |  |
| 1175 | PTGDS | Prostaglandin D2 synthase | DisGeNET |  |  |  |
| 1176 | PTGS1 | Prostaglandin-endoperoxide synthase 1 | DisGeNET | DrugBank |  |  |
| 1177 | PTGS2 | Prostaglandin-Endoperoxide Synthase 2 | GeneCards | DisGeNET |  |  |
| 1178 | PTH | Parathyroid hormone | DisGeNET |  |  |  |
| 1179 | PTHLH | Parathyroid hormone like hormone | DisGeNET |  |  |  |
| 1180 | PTK2 | Protein tyrosine kinase 2 | DisGeNET |  |  |  |
| 1181 | PTK2B | Protein tyrosine kinase 2 beta | DisGeNET |  |  |  |
| 1182 | PTPA | Protein phosphatase 2 phosphatase activator | DisGeNET |  |  |  |
| 1183 | PTPN1 | Protein Tyrosine Phosphatase Non-Receptor Type 1 | GeneCards |  |  |  |
| 1184 | PTPN11 | Protein Tyrosine Phosphatase Non-Receptor Type 11 | GeneCards | DisGeNET |  |  |
| 1185 | PTPN2 | Protein tyrosine phosphatase non-receptor type 2 | DisGeNET |  |  |  |
| 1186 | PTPN22 | Protein Tyrosine Phosphatase Non-Receptor Type 22 | GeneCards | OMIM |  |  |
| 1187 | PTPN6 | Protein tyrosine phosphatase non-receptor type 6 | DisGeNET |  |  |  |
| 1188 | PTX3 | Pentraxin 3 | DisGeNET |  |  |  |
| 1189 | PVT1 | Pvt1 oncogene | DisGeNET |  |  |  |
| 1190 | PWAR1 | Prader Willi/Angelman region RNA 1 | DisGeNET |  |  |  |
| 1191 | PYCARD | PYD and CARD domain containing | DisGeNET |  |  |  |
| 1192 | PYGM | Glycogen Phosphorylase, Muscle Associated | GeneCards |  |  |  |
| 1193 | RAB38 | RAB38, member RAS oncogene family | DisGeNET |  |  |  |
| 1194 | RAB3A | RAB3A, member RAS oncogene family | DisGeNET |  |  |  |
| 1195 | RAB7 | Ras-related protein Rab-7a | OMIM |  |  |  |
| 1196 | RABGEF1 | RAB guanine nucleotide exchange factor 1 | DisGeNET |  |  |  |
| 1197 | RAC1 | Rac family small GTPase 1 | DisGeNET |  |  |  |
| 1198 | RAMP2 | Receptor activity modifying protein 2 | DisGeNET |  |  |  |
| 1199 | RAP1A | RAP1A, member of RAS oncogene family | DisGeNET |  |  |  |
| 1200 | RAPGEF5 | Rap guanine nucleotide exchange factor 5 | DisGeNET |  |  |  |
| 1201 | RARRES2 | Retinoic acid receptor responder 2 | DisGeNET |  |  |  |
| 1202 | RB1CC1 | RB1 inducible coiled-coil 1 | DisGeNET |  |  |  |
| 1203 | RBM45 | RNA binding motif protein 45 | DisGeNET |  |  |  |
| 1204 | RBMS3 | RNA binding motif single stranded interacting protein 3 | DisGeNET |  |  |  |
| 1205 | RBP4 | Retinol Binding Protein 4 | GeneCards | DisGeNET |  |  |
| 1206 | RBPJ | Recombination signal binding protein for immunoglobulin kappa J region | DisGeNET |  |  |  |
| 1207 | RCAN1 | Regulator of calcineurin 1 | DisGeNET |  |  |  |
| 1208 | RCBTB1 | RCC1 and BTB domain containing protein 1 | DisGeNET |  |  |  |
| 1209 | RDX | Radixin | DisGeNET |  |  |  |
| 1210 | RELA | RELA proto-oncogene, NF-kB subunit | DisGeNET |  |  |  |
| 1211 | REM1 | RRAD and GEM like GTPase 1 | DisGeNET |  |  |  |
| 1212 | REN | Renin | GeneCards | DisGeNET | DrugBank | OMIM |
| 1213 | RENBP | Renin binding protein | DisGeNET |  |  |  |
| 1214 | RET | Ret Proto-Oncogene | GeneCards |  |  |  |
| 1215 | RETN | Resistin | GeneCards | DisGeNET | OMIM |  |
| 1216 | RFC1 | Replication factor C subunit 1 | DisGeNET |  |  |  |
| 1217 | RFX6 | Regulatory Factor X6 | GeneCards |  |  |  |
| 1218 | RHBDF2 | Rhomboid 5 homolog 2 | DisGeNET |  |  |  |
| 1219 | RHCE | Blood group Rh(CE) polypeptide | OMIM |  |  |  |
| 1220 | RHO | Rhodopsin | GeneCards |  |  |  |
| 1221 | RIPK2 | Receptor interacting serine/threonine kinase 2 | DisGeNET |  |  |  |
| 1222 | RIPK3 | Receptor interacting serine/threonine kinase 3 | DisGeNET |  |  |  |
| 1223 | RMC1 | Regulator of MON1-CCZ1 | DisGeNET |  |  |  |
| 1224 | RMDN1 | Regulator of microtubule dynamics 1 | DisGeNET |  |  |  |
| 1225 | RMDN2 | Regulator of microtubule dynamics 2 | DisGeNET |  |  |  |
| 1226 | RMDN3 | Regulator of microtubule dynamics 3 | DisGeNET |  |  |  |
| 1227 | RN7SL263P | RNA, 7SL, cytoplasmic 263, pseudogene | DisGeNET |  |  |  |
| 1228 | RNASE2 | Ribonuclease A family member 2 | DisGeNET |  |  |  |
| 1229 | RNF185 | Ring finger protein 185 | DisGeNET |  |  |  |
| 1230 | RNF19A | Ring finger protein 19A, RBR E3 ubiquitin protein ligase | DisGeNET |  |  |  |
| 1231 | ROCK1 | Rho-associated protein kinase 1 | TTD | DisGeNET |  |  |
| 1232 | ROCK2 | Rho associated coiled-coil containing protein kinase 2 | DisGeNET |  |  |  |
| 1233 | ROS1 | ROS proto-oncogene 1, receptor tyrosine kinase | DisGeNET |  |  |  |
| 1234 | RPGRIP1L | RPGRIP1 Like | GeneCards |  |  |  |
| 1235 | RPL36A | Ribosomal protein L36a | DisGeNET |  |  |  |
| 1236 | RPLP0 | Ribosomal protein lateral stalk subunit P0 | DisGeNET |  |  |  |
| 1237 | RPPH1 | Ribonuclease P RNA component H1 | DisGeNET |  |  |  |
| 1238 | RPS19 | Ribosomal protein S19 | DisGeNET |  |  |  |
| 1239 | RPS27A | Ribosomal protein S27a | DisGeNET |  |  |  |
| 1240 | RPS6 | Ribosomal protein S6 | DisGeNET |  |  |  |
| 1241 | RRAD | GTP-binding protein RAD | OMIM |  |  |  |
| 1242 | RRAS | RAS related | DisGeNET |  |  |  |
| 1243 | RYR1 | Ryanodine Receptor 1 | GeneCards |  |  |  |
| 1244 | S100A4 | S100 calcium binding protein A4 | DisGeNET |  |  |  |
| 1245 | S100A8 | S100 calcium binding protein A8 | DisGeNET |  |  |  |
| 1246 | S100A9 | S100 calcium binding protein A9 | DisGeNET |  |  |  |
| 1247 | S100B | S100 calcium binding protein B | DisGeNET |  |  |  |
| 1248 | SAA1 | Serum amyloid A1 | DisGeNET |  |  |  |
| 1249 | SAA2 | Serum amyloid A2 | DisGeNET |  |  |  |
| 1250 | SAG | S-Antigen Visual Arrestin | GeneCards | OMIM |  |  |
| 1251 | SAR1B | GTP-binding protein SAR1b | OMIM |  |  |  |
| 1252 | SASH1 | SAM and SH3 domain containing 1 | DisGeNET |  |  |  |
| 1253 | SBF2 | SET Binding Factor 2 | GeneCards |  |  |  |
| 1254 | SCAF4 | SR-related CTD associated factor 4 | DisGeNET |  |  |  |
| 1255 | SCAF8 | SR-related CTD associated factor 8 | DisGeNET |  |  |  |
| 1256 | SCD | Stearoyl-CoA desaturase | DisGeNET |  |  |  |
| 1257 | SDC2 | Syndecan 2 | DisGeNET |  |  |  |
| 1258 | SDCCAG8 | SHH Signaling And Ciliogenesis Regulator SDCCAG8 | GeneCards |  |  |  |
| 1259 | SDHB | Succinate Dehydrogenase Complex Iron Sulfur Subunit B | GeneCards |  |  |  |
| 1260 | SEC61A1 | Protein transport protein Sec61 subunit alpha isoform 1 | OMIM |  |  |  |
| 1261 | SEC63 | SEC63 Homolog, Protein Translocation Regulator | GeneCards | OMIM |  |  |
| 1262 | SELE | Selectin E | GeneCards |  |  |  |
| 1263 | SELENBP1 | Selenium binding protein 1 | DisGeNET |  |  |  |
| 1264 | SELL | Selectin L | DisGeNET |  |  |  |
| 1265 | SELP | Selectin P | DisGeNET |  |  |  |
| 1266 | SEMA3A | Semaphorin 3A | DisGeNET |  |  |  |
| 1267 | SEMA6A | Semaphorin 6A | DisGeNET |  |  |  |
| 1268 | SEPTIN7 | Septin 7 | DisGeNET |  |  |  |
| 1269 | SERPINA1 | Serpin Family A Member 1 | GeneCards |  |  |  |
| 1270 | SERPINA3 | Serpin Family A Member 3 | GeneCards |  |  |  |
| 1271 | SERPINB2 | Serpin family B member 2 | DisGeNET |  |  |  |
| 1272 | SERPINE1 | Serpin Family E Member 1 | GeneCards | DisGeNET |  |  |
| 1273 | SERPINF1 | Serpin family F member 1 | DisGeNET |  |  |  |
| 1274 | SETD7 | SET domain containing 7, histone lysine methyltransferase | DisGeNET |  |  |  |
| 1275 | SFI1 | SFI1 centrin binding protein | DisGeNET |  |  |  |
| 1276 | SFRP4 | Secreted frizzled-related protein 4 | DrugBank | OMIM |  |  |
| 1277 | SGK1 | Serum/glucocorticoid regulated kinase 1 | DisGeNET |  |  |  |
| 1278 | SGLT2 | Sodium/glucose cotransporter 2 | TTD |  |  |  |
| 1279 | SH3TC2 | SH3 Domain And Tetratricopeptide Repeats 2 | GeneCards | OMIM |  |  |
| 1280 | SHBG | Sex hormone binding globulin | DisGeNET |  |  |  |
| 1281 | SHH | Sonic Hedgehog Signaling Molecule | GeneCards |  |  |  |
| 1282 | SI | Sucrase-isomaltase | DisGeNET |  |  |  |
| 1283 | SIRT1 | Sirtuin 1 | DisGeNET |  |  |  |
| 1284 | SIRT3 | Sirtuin 3 | DisGeNET |  |  |  |
| 1285 | SIRT4 | Sirtuin 4 | DisGeNET |  |  |  |
| 1286 | SIRT6 | Sirtuin 6 | DisGeNET |  |  |  |
| 1287 | SIRT7 | Sirtuin 7 | DisGeNET |  |  |  |
| 1288 | SKIL | SKI like proto-oncogene | DisGeNET |  |  |  |
| 1289 | SKP2 | S-phase kinase associated protein 2 | DisGeNET |  |  |  |
| 1290 | SLBP | Stem-loop binding protein | DisGeNET |  |  |  |
| 1291 | SLC12A1 | Solute Carrier Family 12 Member 1 | GeneCards | DisGeNET |  |  |
| 1292 | SLC12A3 | Solute Carrier Family 12 Member 3 | GeneCards | DisGeNET |  |  |
| 1293 | SLC15A1 | Solute carrier family 15 member 1 | DrugBank |  |  |  |
| 1294 | SLC15A2 | Solute carrier family 15 member 2 | DrugBank |  |  |  |
| 1295 | SLC16A3 | Solute carrier family 16 member 3 | DisGeNET |  |  |  |
| 1296 | SLC17A1 | Sodium-dependent phosphate transport protein 1 | OMIM |  |  |  |
| 1297 | SLC17A5 | Solute Carrier Family 17 Member 5 | GeneCards | DisGeNET | OMIM |  |
| 1298 | SLC19A2 | Solute Carrier Family 19 Member 2 | GeneCards |  |  |  |
| 1299 | SLC22A12 | Solute carrier family 22 member 12 | DrugBank |  |  |  |
| 1300 | SLC22A2 | Solute carrier family 22 member 2 | DisGeNET |  |  |  |
| 1301 | SLC22A3 | Solute carrier family 22 member 3 | DisGeNET |  |  |  |
| 1302 | SLC22A6 | Solute carrier family 22 member 6 | DisGeNET | DrugBank |  |  |
| 1303 | SLC22A7 | Solute carrier family 22 member 7 | DrugBank |  |  |  |
| 1304 | SLC22A8 | Solute carrier family 22 member 8 | DisGeNET | DrugBank |  |  |
| 1305 | SLC29A3 | Solute Carrier Family 29 Member 3 | GeneCards |  |  |  |
| 1306 | SLC2A1 | Solute Carrier Family 2 Member 1 | GeneCards | DisGeNET |  |  |
| 1307 | SLC2A12 | Solute carrier family 2 member 12 | DisGeNET |  |  |  |
| 1308 | SLC2A2 | Solute Carrier Family 2 Member 2 | GeneCards | DisGeNET | OMIM |  |
| 1309 | SLC2A4 | Solute Carrier Family 2 Member 4 | GeneCards | DisGeNET |  |  |
| 1310 | SLC2A9 | Solute carrier family 2, facilitated glucose transporter member 9 | DrugBank |  |  |  |
| 1311 | SLC30A7 | Solute carrier family 30 member 7 | DisGeNET |  |  |  |
| 1312 | SLC30A8 | Solute Carrier Family 30 Member 8 | GeneCards | DisGeNET | OMIM |  |
| 1313 | SLC33A1 | Solute carrier family 33 member 1 | DisGeNET |  |  |  |
| 1314 | SLC34A1 | Solute Carrier Family 34 Member 1 | GeneCards |  |  |  |
| 1315 | SLC37A4 | Solute Carrier Family 37 Member 4 | GeneCards |  |  |  |
| 1316 | SLC52A1 | Solute carrier family 52 member 1 | DisGeNET |  |  |  |
| 1317 | SLC52A2 | Solute carrier family 52 member 2 | DisGeNET |  |  |  |
| 1318 | SLC5A1 | Solute carrier family 5 member 1 | DisGeNET |  |  |  |
| 1319 | SLC5A2 | Solute Carrier Family 5 Member 2 | GeneCards | DisGeNET | DrugBank |  |
| 1320 | SLC7A2 | Solute carrier family 7 member 2 | DisGeNET |  |  |  |
| 1321 | SLC9A8 | Solute carrier family 9 member A8 | DisGeNET |  |  |  |
| 1322 | SLCO1A2 | Solute carrier organic anion transporter family member 1A2 | DrugBank |  |  |  |
| 1323 | SLCO1B1 | Solute carrier organic anion transporter family member 1B1 | DrugBank |  |  |  |
| 1324 | SLCO1B3 | Solute carrier organic anion transporter family member 1B3 | DrugBank |  |  |  |
| 1325 | SLCO6A1 | Solute carrier organic anion transporter family member 6A1 | DisGeNET |  |  |  |
| 1326 | SLIT2 | Slit guidance ligand 2 | DisGeNET |  |  |  |
| 1327 | SLPI | Secretory leukocyte peptidase inhibitor | DisGeNET |  |  |  |
| 1328 | SMAD1 | SMAD family member 1 | DisGeNET |  |  |  |
| 1329 | SMAD2 | SMAD family member 2 | DisGeNET |  |  |  |
| 1330 | SMAD3 | SMAD Family Member 3 | GeneCards | DisGeNET |  |  |
| 1331 | SMAD4 | SMAD Family Member 4 | GeneCards |  |  |  |
| 1332 | SMAD5 | SMAD family member 5 | DisGeNET |  |  |  |
| 1333 | SMAD6 | SMAD family member 6 | DisGeNET |  |  |  |
| 1334 | SMAD7 | SMAD family member 7 | DisGeNET |  |  |  |
| 1335 | SMG1 | SMG1 nonsense mediated mRNA decay associated PI3K related kinase | DisGeNET |  |  |  |
| 1336 | SMN1 | Survival of motor neuron 1, telomeric | DisGeNET |  |  |  |
| 1337 | SMN2 | Survival of motor neuron 2, centromeric | DisGeNET |  |  |  |
| 1338 | SMPD1 | Sphingomyelin Phosphodiesterase 1 | GeneCards |  |  |  |
| 1339 | SMPDL3B | Sphingomyelin phosphodiesterase acid like 3B | DisGeNET |  |  |  |
| 1340 | SMURF2 | SMAD specific E3 ubiquitin protein ligase 2 | DisGeNET |  |  |  |
| 1341 | SNAI1 | Snail family transcriptional repressor 1 | DisGeNET |  |  |  |
| 1342 | SNCA | Synuclein Alpha | GeneCards | OMIM |  |  |
| 1343 | SNRNP70 | Small nuclear ribonucleoprotein U1 subunit 70 | DisGeNET |  |  |  |
| 1344 | SOAT1 | Sterol O-acyltransferase 1 | DisGeNET |  |  |  |
| 1345 | SOCS1 | Suppressor of cytokine signaling 1 | DisGeNET |  |  |  |
| 1346 | SOCS2 | Suppressor of cytokine signaling 2 | DisGeNET |  |  |  |
| 1347 | SOCS3 | Suppressor of cytokine signaling 3 | DisGeNET |  |  |  |
| 1348 | SOCS5 | Suppressor of cytokine signaling 5 | DisGeNET |  |  |  |
| 1349 | SOD1 | Superoxide Dismutase 1 | GeneCards | DisGeNET |  |  |
| 1350 | SOD2 | Superoxide Dismutase 2 | GeneCards | DisGeNET | OMIM |  |
| 1351 | SOD3 | Superoxide dismutase 3 | DisGeNET |  |  |  |
| 1352 | SORBS1 | Sorbin and SH3 domain containing 1 | DisGeNET |  |  |  |
| 1353 | SORD | Sorbitol dehydrogenase | DisGeNET |  |  |  |
| 1354 | SOS1 | SOS Ras/Rac guanine nucleotide exchange factor 1 | DisGeNET |  |  |  |
| 1355 | SOST | Sclerostin | DisGeNET |  |  |  |
| 1356 | SOSTDC1 | Sclerostin domain containing 1 | DisGeNET |  |  |  |
| 1357 | SOX10 | SRY-Box Transcription Factor 10 | GeneCards |  |  |  |
| 1358 | SOX2 | SRY-box transcription factor 2 | DisGeNET |  |  |  |
| 1359 | SOX2-OT | SOX2 overlapping transcript | DisGeNET |  |  |  |
| 1360 | SOX6 | SRY-box transcription factor 6 | DisGeNET |  |  |  |
| 1361 | SP1 | Sp1 transcription factor | DisGeNET |  |  |  |
| 1362 | SP110 | Sp110 nuclear body protein | OMIM |  |  |  |
| 1363 | SPARC | Secreted protein acidic and cysteine rich | DisGeNET |  |  |  |
| 1364 | SPHK1 | Sphingosine kinase 1 | DisGeNET |  |  |  |
| 1365 | SPINK1 | Serine Peptidase Inhibitor Kazal Type 1 | GeneCards | DisGeNET | OMIM |  |
| 1366 | SPP1 | Secreted Phosphoprotein 1 | GeneCards | DisGeNET |  |  |
| 1367 | SPRY2 | Protein sprouty homolog 2 | OMIM |  |  |  |
| 1368 | SPZ1 | Spermatogenic leucine zipper 1 | DisGeNET |  |  |  |
| 1369 | SQSTM1 | Sequestosome 1 | GeneCards | DisGeNET | OMIM |  |
| 1370 | SREBF1 | Sterol Regulatory Element Binding Transcription Factor 1 | GeneCards | DisGeNET |  |  |
| 1371 | SREBF2 | Sterol regulatory element binding transcription factor 2 | DisGeNET |  |  |  |
| 1372 | SRGAP2 | SLIT-ROBO Rho GTPase activating protein 2 | DisGeNET |  |  |  |
| 1373 | SRRM2 | Serine/arginine repetitive matrix 2 | DisGeNET |  |  |  |
| 1374 | SRXN1 | Sulfiredoxin 1 | DisGeNET |  |  |  |
| 1375 | SRY | Sex determining region Y | DisGeNET |  |  |  |
| 1376 | SST | Somatostatin | GeneCards |  |  |  |
| 1377 | ST3GAL4 | ST3 beta-galactoside alpha-2,3-sialyltransferase 4 | DisGeNET |  |  |  |
| 1378 | STAM2 | Signal transducing adaptor molecule 2 | DisGeNET |  |  |  |
| 1379 | STAP2 | Signal transducing adaptor family member 2 | DisGeNET |  |  |  |
| 1380 | STAT1 | Signal Transducer And Activator Of Transcription 1 | GeneCards | DisGeNET |  |  |
| 1381 | STAT3 | Signal Transducer And Activator Of Transcription 3 | GeneCards | DisGeNET |  |  |
| 1382 | STAT5A | Signal transducer and activator of transcription 5A | DisGeNET |  |  |  |
| 1383 | STAT5B | Signal transducer and activator of transcription 5B | DisGeNET |  |  |  |
| 1384 | STC1 | Stanniocalcin 1 | DisGeNET |  |  |  |
| 1385 | STIM1 | Stromal interaction molecule 1 | DisGeNET |  |  |  |
| 1386 | STIM2 | Stromal interaction molecule 2 | DisGeNET |  |  |  |
| 1387 | STK11 | Serine/threonine kinase 11 | DisGeNET |  |  |  |
| 1388 | STS | Steroid sulfatase | DisGeNET |  |  |  |
| 1389 | SUMO4 | Small Ubiquitin Like Modifier 4 | GeneCards | DisGeNET | OMIM |  |
| 1390 | SURF1 | SURF1 Cytochrome C Oxidase Assembly Factor | GeneCards |  |  |  |
| 1391 | SUV39H1 | Suppressor of variegation 3-9 homolog 1 | DisGeNET |  |  |  |
| 1392 | SYBU | Syntabulin | DisGeNET |  |  |  |
| 1393 | SYK | Spleen associated tyrosine kinase | DisGeNET |  |  |  |
| 1394 | SYT1 | Synaptotagmin 1 | DisGeNET |  |  |  |
| 1395 | SYVN1 | Synoviolin 1 | DisGeNET |  |  |  |
| 1396 | TALDO1 | Transaldolase 1 | DisGeNET |  |  |  |
| 1397 | TAM | Myeloproliferative syndrome, transient (transient abnormal | DisGeNET |  |  |  |
| 1398 | TAS2R13 | taste 2 receptor member 13 | DisGeNET |  |  |  |
| 1399 | TBC1D31 | TBC1 domain family member 31 | DisGeNET |  |  |  |
| 1400 | TBC1D4 | TBC1 Domain Family Member 4 | GeneCards | OMIM |  |  |
| 1401 | TBP | TATA-box-binding protein | OMIM |  |  |  |
| 1402 | TBX1 | T-box transcription factor 1 | DisGeNET |  |  |  |
| 1403 | TBX18 | T-Box Transcription Factor 18 | GeneCards | OMIM |  |  |
| 1404 | TCF7 | Transcription factor 7 | DisGeNET |  |  |  |
| 1405 | TCF7L2 | Transcription Factor 7 Like 2 | GeneCards | DisGeNET | OMIM |  |
| 1406 | TERC | Telomerase RNA component | DisGeNET |  |  |  |
| 1407 | TERF1 | Telomeric repeat binding factor 1 | DisGeNET |  |  |  |
| 1408 | TERF2IP | TERF2 interacting protein | DisGeNET |  |  |  |
| 1409 | TERT | Telomerase Reverse Transcriptase | GeneCards |  |  |  |
| 1410 | TET2 | Tet methylcytosine dioxygenase 2 | DisGeNET |  |  |  |
| 1411 | TF | Transferrin | DisGeNET |  |  |  |
| 1412 | TFG | Trafficking from ER to golgi regulator | DisGeNET |  |  |  |
| 1413 | TFPI | Tissue factor pathway inhibitor | DisGeNET |  |  |  |
| 1414 | TFRC | Transferrin receptor | DisGeNET |  |  |  |
| 1415 | TG | Thyroglobulin | GeneCards |  |  |  |
| 1416 | TGFA | Transforming growth factor alpha | DisGeNET |  |  |  |
| 1417 | TGFB1 | Transforming Growth Factor Beta 1 | GeneCards | DisGeNET |  |  |
| 1418 | TGFB2 | Transforming growth factor beta 2 | DisGeNET |  |  |  |
| 1419 | TGFB3 | Ttransforming growth factor beta 3 | DisGeNET |  |  |  |
| 1420 | TGFBI | Transforming growth factor beta induced | DisGeNET |  |  |  |
| 1421 | TGFBR1 | transforming growth factor beta receptor 1 | TTD | DisGeNET |  |  |
| 1422 | TGFBR2 | Transforming growth factor beta receptor 2 | DisGeNET |  |  |  |
| 1423 | TGIF1 | TGFB induced factor homeobox 1 | DisGeNET |  |  |  |
| 1424 | TGM2 | Transglutaminase 2 | DisGeNET |  |  |  |
| 1425 | TH | Tyrosine Hydroxylase | GeneCards |  |  |  |
| 1426 | THBD | Thrombomodulin | GeneCards | DisGeNET |  |  |
| 1427 | THBS1 | Thrombospondin 1 | DisGeNET |  |  |  |
| 1428 | THG1L | tRNA-histidine guanylyltransferase 1 like | DisGeNET |  |  |  |
| 1429 | TIMM44 | Translocase of inner mitochondrial membrane 44 | DisGeNET |  |  |  |
| 1430 | TIMP1 | TIMP metallopeptidase inhibitor 1 | DisGeNET |  |  |  |
| 1431 | TIMP2 | TIMP metallopeptidase inhibitor 2 | DisGeNET |  |  |  |
| 1432 | TIMP3 | TIMP metallopeptidase inhibitor 3 | DisGeNET |  |  |  |
| 1433 | TINAG | Tubulointerstitial nephritis antigen | DisGeNET |  |  |  |
| 1434 | TKT | Transketolase | DisGeNET |  |  |  |
| 1435 | TLR2 | Toll Like Receptor 2 | GeneCards | DisGeNET |  |  |
| 1436 | TLR4 | Toll Like Receptor 4 | GeneCards | DisGeNET |  |  |
| 1437 | TLR5 | Toll-like receptor 5 | OMIM |  |  |  |
| 1438 | TMEM216 | Transmembrane Protein 216 | GeneCards |  |  |  |
| 1439 | TMEM231 | Transmembrane Protein 231 | GeneCards |  |  |  |
| 1440 | TMEM67 | Transmembrane Protein 67 | GeneCards |  |  |  |
| 1441 | TNF | Tumor Necrosis Factor | GeneCards | DisGeNET |  |  |
| 1442 | TNFAIP1 | TNF alpha induced protein 1 | DisGeNET |  |  |  |
| 1443 | TNFAIP8 | TNF alpha induced protein 8 | DisGeNET |  |  |  |
| 1444 | TNFRSF10D | TNF receptor superfamily member 10d | DisGeNET |  |  |  |
| 1445 | TNFRSF11A | TNF Receptor Superfamily Member 11a | GeneCards |  |  |  |
| 1446 | TNFRSF11B | TNF Receptor Superfamily Member 11b | GeneCards | DisGeNET |  |  |
| 1447 | TNFRSF1A | TNF Receptor Superfamily Member 1A | GeneCards | DisGeNET |  |  |
| 1448 | TNFRSF1B | TNF receptor superfamily member 1B | DisGeNET |  |  |  |
| 1449 | TNFSF10 | TNF superfamily member 10 | DisGeNET |  |  |  |
| 1450 | TNMD | Tenomodulin | DisGeNET |  |  |  |
| 1451 | TNNI3K | Serine/threonine-protein kinase TNNI3K | OMIM |  |  |  |
| 1452 | TNS2 | Tensin 2 | DisGeNET |  |  |  |
| 1453 | TP53 | Tumor Protein P53 | GeneCards | DisGeNET |  |  |
| 1454 | TP53COR1 | Tumor protein p53 pathway corepressor 1 | DisGeNET |  |  |  |
| 1455 | TPD52 | Tumor protein D52 | DisGeNET |  |  |  |
| 1456 | TRAF5 | TNF receptor associated factor 5 | DisGeNET |  |  |  |
| 1457 | TRAF6 | TNF receptor associated factor 6 | DisGeNET |  |  |  |
| 1458 | TRDN | Triadin | DisGeNET |  |  |  |
| 1459 | TREM1 | Triggering receptor expressed on myeloid cells 1 | DisGeNET |  |  |  |
| 1460 | TRH | Thyrotropin releasing hormone | DisGeNET |  |  |  |
| 1461 | TRIAP1 | TP53 regulated inhibitor of apoptosis 1 | DisGeNET |  |  |  |
| 1462 | TRIB3 | Tribbles pseudokinase 3 | DisGeNET |  |  |  |
| 1463 | TRIM11 | Tripartite motif containing 11 | DisGeNET |  |  |  |
| 1464 | TRIM2 | Tripartite motif-containing protein 2 | OMIM |  |  |  |
| 1465 | TRIP10 | Thyroid hormone receptor interactor 10 | DisGeNET |  |  |  |
| 1466 | TRNT | tRNA | DisGeNET |  |  |  |
| 1467 | TRPC1 | Transient receptor potential cation channel subfamily C member 1 | DisGeNET |  |  |  |
| 1468 | TRPC5 | Transient receptor potential cation channel subfamily C member 5 | DisGeNET |  |  |  |
| 1469 | TRPC6 | Transient receptor potential cation channel subfamily C member 6 | DisGeNET |  |  |  |
| 1470 | TRPV4 | Transient Receptor Potential Cation Channel Subfamily V Member 4 | GeneCards |  |  |  |
| 1471 | TSC1 | TSC Complex Subunit 1 | GeneCards | DisGeNET |  |  |
| 1472 | TSC2 | TSC Complex Subunit 2 | GeneCards |  |  |  |
| 1473 | TSC22D1 | TSC22 domain family member 1 | DisGeNET |  |  |  |
| 1474 | TSPYL2 | TSPY like 2 | DisGeNET |  |  |  |
| 1475 | TTC21B | Tetratricopeptide Repeat Domain 21B | GeneCards |  |  |  |
| 1476 | TTC8 | Tetratricopeptide Repeat Domain 8 | GeneCards |  |  |  |
| 1477 | TTR | Transthyretin | GeneCards |  |  |  |
| 1478 | TUG1 | Taurine up-regulated 1 | DisGeNET |  |  |  |
| 1479 | TWIST1 | Twist family bHLH transcription factor 1 | DisGeNET |  |  |  |
| 1480 | TXN | Thioredoxin | DisGeNET |  |  |  |
| 1481 | TXNIP | Thioredoxin interacting protein | DisGeNET |  |  |  |
| 1482 | TYRO3 | TYRO3 protein tyrosine kinase | DisGeNET |  |  |  |
| 1483 | UBE2V1 | Ubiquitin conjugating enzyme E2 V1 | DisGeNET |  |  |  |
| 1484 | UBE4A | Ubiquitination factor E4A | DisGeNET |  |  |  |
| 1485 | UCA1 | Urothelial cancer associated 1 | DisGeNET |  |  |  |
| 1486 | UCHL1 | Ubiquitin C-Terminal Hydrolase L1 | GeneCards | DisGeNET | OMIM |  |
| 1487 | UCP1 | Uncoupling protein 1 | DisGeNET |  |  |  |
| 1488 | UCP2 | Uncoupling protein 2 | DisGeNET |  |  |  |
| 1489 | UCP3 | Uncoupling protein 3 | DisGeNET | OMIM |  |  |
| 1490 | UGT1-1 | UDP-glucuronosyltransferase 1-1 | DrugBank |  |  |  |
| 1491 | UGT1-10 | UDP-glucuronosyltransferase 1-10 | DrugBank |  |  |  |
| 1492 | UGT1-3 | UDP-glucuronosyltransferase 1-3 | DrugBank |  |  |  |
| 1493 | UGT1-9 | UDP-glucuronosyltransferase 1-9 | DrugBank |  |  |  |
| 1494 | UGT2B17 | UDP-glucuronosyltransferase 2B17 | DrugBank |  |  |  |
| 1495 | UGT2B4 | UDP-glucuronosyltransferase 2B4 | DrugBank |  |  |  |
| 1496 | UGT2B7 | UDP-glucuronosyltransferase 2B7 | DrugBank |  |  |  |
| 1497 | UGT8 | UDP glycosyltransferase 8 | DisGeNET |  |  |  |
| 1498 | ULK1 | Unc-51 like autophagy activating kinase 1 | DisGeNET |  |  |  |
| 1499 | UMOD | Uromodulin | GeneCards | DisGeNET |  |  |
| 1500 | UNC13B | Unc-13 homolog B | DisGeNET |  |  |  |
| 1501 | UTRN | Utrophin | DisGeNET |  |  |  |
| 1502 | UTS2 | Urotensin 2 | DisGeNET |  |  |  |
| 1503 | UTS2R | urotensin 2 receptor | TTD | DisGeNET |  |  |
| 1504 | VAC14 | VAC14 component of PIKFYVE complex | DisGeNET |  |  |  |
| 1505 | VASH1 | Vasohibin 1 | DisGeNET |  |  |  |
| 1506 | VASH2 | Vasohibin 2 | DisGeNET |  |  |  |
| 1507 | VAV1 | Vav guanine nucleotide exchange factor 1 | DisGeNET |  |  |  |
| 1508 | VCAM1 | Vascular Cell Adhesion Molecule 1 | GeneCards | DisGeNET |  |  |
| 1509 | VCAN | Versican | DisGeNET |  |  |  |
| 1510 | VCP | Valosin Containing Protein | GeneCards |  |  |  |
| 1511 | VDAC1 | Voltage dependent anion channel 1 | DisGeNET |  |  |  |
| 1512 | VDAC2 | Voltage dependent anion channel 2 | DisGeNET |  |  |  |
| 1513 | VDR | Vitamin D Receptor | GeneCards | DisGeNET |  |  |
| 1514 | VEGFA | Vascular Endothelial Growth Factor A | GeneCards | DisGeNET | OMIM |  |
| 1515 | VEGFB | Vascular endothelial growth factor B | DisGeNET |  |  |  |
| 1516 | VEGFC | Vascular endothelial growth factor C | DisGeNET |  |  |  |
| 1517 | VHL | Von Hippel-Lindau Tumor Suppressor | GeneCards |  |  |  |
| 1518 | VIM | Vimentin | DisGeNET |  |  |  |
| 1519 | VPS51 | VPS51 subunit of GARP complex | DisGeNET |  |  |  |
| 1520 | VSIG4 | V-set and immunoglobulin domain containing 4 | DisGeNET |  |  |  |
| 1521 | VTN | Vitronectin | DisGeNET |  |  |  |
| 1522 | VWF | Von Willebrand Factor | GeneCards | DisGeNET |  |  |
| 1523 | WARS1 | Tryptophanyl-tRNA synthetase 1 | DisGeNET |  |  |  |
| 1524 | WDR19 | WD Repeat Domain 19 | GeneCards |  |  |  |
| 1525 | WDR83 | WD repeat domain 83 | DisGeNET |  |  |  |
| 1526 | WFS1 | Wolframin ER Transmembrane Glycoprotein | GeneCards | DisGeNET | OMIM |  |
| 1527 | WNK1 | WNK lysine deficient protein kinase 1 | DisGeNET |  |  |  |
| 1528 | WSPAR | WNT signaling pathway activating non-coding RNA | DisGeNET |  |  |  |
| 1529 | WT1 | WT1 Transcription Factor | GeneCards | DisGeNET |  |  |
| 1530 | WWTR1 | WW domain containing transcription regulator 1 | DisGeNET |  |  |  |
| 1531 | XBP1 | X-box binding protein 1 | DisGeNET |  |  |  |
| 1532 | XDH | Xanthine dehydrogenase | DisGeNET |  |  |  |
| 1533 | XIST | X inactive specific transcript | DisGeNET |  |  |  |
| 1534 | XPNPEP3 | Xaa-Pro aminopeptidase 3 | OMIM |  |  |  |
| 1535 | XPR1 | Xenotropic and polytropic retrovirus receptor 1 | DisGeNET |  |  |  |
| 1536 | XRCC1 | X-ray repair cross complementing 1 | DisGeNET |  |  |  |
| 1537 | XYLT1 | Xylosyltransferase 1 | DisGeNET |  |  |  |
| 1538 | XYLT2 | Xylosyltransferase 2 | DisGeNET |  |  |  |
| 1539 | YAP1 | Yes associated protein 1 | DisGeNET |  |  |  |
| 1540 | YARS1 | Tyrosine--tRNA ligase, cytoplasmic | OMIM |  |  |  |
| 1541 | YBX1 | Y-box binding protein 1 | DisGeNET |  |  |  |
| 1542 | YIPF5 | Protein YIPF5 | OMIM |  |  |  |
| 1543 | YWHAZ | Tyrosine 3-monooxygenase/tryptophan 5-monooxygenase activation protein zeta | DisGeNET |  |  |  |
| 1544 | YY1 | YY1 transcription factor | DisGeNET |  |  |  |
| 1545 | ZAP70 | Tyrosine-protein kinase ZAP-70 | OMIM |  |  |  |
| 1546 | ZBTB16 | Zinc finger and BTB domain containing 16 | DisGeNET |  |  |  |
| 1547 | ZEB1 | Zinc finger E-box binding homeobox 1 | DisGeNET |  |  |  |
| 1548 | ZEB2 | Zinc finger E-box binding homeobox 2 | DisGeNET |  |  |  |
| 1549 | ZFP36 | ZFP36 ring finger protein | DisGeNET |  |  |  |
| 1550 | ZFP57 | ZFP57 Zinc Finger Protein | GeneCards | OMIM |  |  |
| 1551 | ZGLP1 | Zinc finger GATA like protein 1 | DisGeNET |  |  |  |
| 1552 | ZNF236 | Zinc finger protein 236 | DisGeNET |  |  |  |
| 1553 | ZNF410 | Zinc finger protein 410 | DisGeNET |  |  |  |
| 1554 | ZNF687 | Zinc finger protein 687 | OMIM |  |  |  |
