# Supplementary material for: Cellular mechanism of action of forsythiaside for the treatment of diabetic kidney disease
Source: Front Pharmacol. 2023 Jan 13;13:1096536. doi: 10.3389/fphar.2022.1096536 (PMC9880420; doi:10.3389/fphar.2022.1096536)
Supplement: Supplementary file 4 [file Table3.DOCX]

**Table S3**. Diabetic podocytopathy related targets.

| **Number** | **Gene name** | **Protein name** | **Database** |
| --- | --- | --- | --- |
| 1 | ABCC8 | ATP-binding cassette, subfamily C, member 8 (sulfonylurea receptor) | OMIM |
| 2 | ACE | Angiotensin I converting enzyme (dipeptidyl carboxypeptidase-1) | OMIM |
| 3 | ACTN4 | Actinin Alpha 4 | GeneCards |
| 4 | AGER | Advanced Glycosylation End-Product Specific Receptor | GeneCards |
| 5 | AKT2 | AKT serine/threonine kinase 2 | OMIM |
| 6 | APOL1 | Apolipoprotein L1 | GeneCards |
| 7 | APPL1 | Adaptor protein containing PH domain, PTB domain, and leucine zipper motif 1 | OMIM |
| 8 | AQP2 | Aquaporin-2 (collecting duct) | OMIM |
| 9 | AVP | Arginine vasopressin (neurophysin II, antidiuretic hormone) | OMIM |
| 10 | AVPR2 | Arginine vasopressin receptor-2 | OMIM |
| 11 | BLK | BLK protooncogene, SRC family tyrosinase kinase | OMIM |
| 12 | CAMK4 | Calcium/Calmodulin Dependent Protein Kinase IV | GeneCards |
| 13 | CAPN10 | Calpain-10 | OMIM |
| 14 | CCR5 | Chemokine (C-C) receptor 5 | OMIM |
| 15 | CEL | Carboxyl-ester lipase (bile-salt stimulated lipase) | OMIM |
| 16 | COL4A3 | Collagen Type IV Alpha 3 Chain | GeneCards |
| 17 | COL4A4 | Collagen Type IV Alpha 4 Chain | GeneCards |
| 18 | CTLA4 | Cytotoxic T-lymphocyte-associated serine esterase-4 | OMIM |
| 19 | DDN | Dendrin | GeneCards |
| 20 | DNAJC3 | DnaJ, E. coli, homolog of, subfamily C, member 3 (protein kinase inhibitor p58) | OMIM |
| 21 | ELAVL1 | ELAV Like RNA Binding Protein 1 | GeneCards |
| 22 | ENPP1 | Ectonucleotide pyrophosphatase/phosphodiesterase 1 (Ly-41 antigen, mouse, homolog of) | OMIM |
| 23 | EPO | Erythropoietin | OMIM |
| 24 | EZR | Ezrin | GeneCards |
| 25 | FHL2 | Four And A Half LIM Domains 2 | GeneCards |
| 26 | FOXC2 | Forkhead box C2 | OMIM |
| 27 | GCK | Glucokinase (hexokinase-4) | OMIM |
| 28 | GLIS3 | GLIS family zinc finger protein 3 | OMIM |
| 29 | GPD2 | Glycerol-3-phosphate dehydrogenase 2 (mitochondrial) | OMIM |
| 30 | HDAC1 | Histone Deacetylase 1 | GeneCards |
| 31 | HDAC2 | Histone Deacetylase 2 | GeneCards |
| 32 | HFE | Homeostatic iron regulator | OMIM |
| 33 | HMGA1 | High-mobility group AT-hook 1 | OMIM |
| 34 | HNF1A | HNF1 homeobox B | OMIM |
| 35 | HNF1B | HNF1 homeobox B (transcription factor 2) | OMIM |
| 36 | HNF4A | Hepatocyte nuclear factor 4, alpha (transcription factor-14) | OMIM |
| 37 | IAPP | Islet amyloid polypeptide (diabetes-associated peptide; amylin) | OMIM |
| 38 | IDDM1 | Insulin-dependent diabetes mellitus-1 | OMIM |
| 39 | IDDM11 | Insulin-dependent diabetes mellitus-11 | OMIM |
| 40 | IDDM13 | Insulin-dependent diabetes mellitus-13 | OMIM |
| 41 | IDDM15 | Insulin-dependent diabetes mellitus-15 | OMIM |
| 42 | IDDM17 | Insulin-dependent diabetes mellitus-17 | OMIM |
| 43 | IDDM18 | Insulin-dependent diabetes mellitus-18 | OMIM |
| 44 | IDDM19 | Diabetes mellitus, insulin-dependent, 19 | OMIM |
| 45 | IDDM21 | Diabetes mellitus, insulin-dependent, 21 | OMIM |
| 46 | IDDM23 | Diabetes mellitus, insulin-dependent, 23 | OMIM |
| 47 | IDDM24 | Diabetes mellitus, insulin-dependent, 24 | OMIM |
| 48 | IDDM3 | Insulin-dependent diabetes mellitus-3 | OMIM |
| 49 | IDDM4 | Insulin-dependent diabetes mellitus-4 | OMIM |
| 50 | IDDM6 | Insulin-dependent diabetes mellitus-6 | OMIM |
| 51 | IDDM7 | Insulin-dependent diabetes mellitus-7 | OMIM |
| 52 | IDDM8 | Insulin-dependent diabetes mellitus-8 | OMIM |
| 53 | IDDMX | Diabetes mellitus, insulin-dependent, X-linked, susceptibility to | OMIM |
| 54 | IER3IP1 | Immediate-early response 3-interacting protein 1 | OMIM |
| 55 | IGF2BP2 | Insulin-like growth factor 2 mRNA-binding protein 2 | OMIM |
| 56 | IL1RN | Interleukin-1 receptor antagonist | OMIM |
| 57 | IL2RA | Interleukin-2 receptor, alpha | OMIM |
| 58 | IL6 | Interleukin-6 (interferon, beta-2) | OMIM |
| 59 | INS | Insulin | OMIM |
| 60 | INSR | Insulin receptor | OMIM |
| 61 | IRS1 | Insulin receptor substrate-1 | OMIM |
| 62 | IRS2 | Insulin receptor substrate 2 | OMIM |
| 63 | ITPR3 | Inositol 1,4,5-triphosphate receptor, type 3 | OMIM |
| 64 | KCNJ11 | Potassium inwardly-rectifying channel, subfamily J, member 11 | OMIM |
| 65 | KLF11 | Kruppel-like factor 11 | OMIM |
| 66 | LIPC | Lipase C, hepatic | OMIM |
| 67 | LMX1B | LIM Homeobox Transcription Factor 1 Beta | GeneCards |
| 68 | MAFA | MAF bZIP transcription factor A | OMIM |
| 69 | MAPK8IP1 | Mitogen-activated protein kinase 8-interacting protein 1 | OMIM |
| 70 | MIA3 | MIA SH3 domain ER export factor 3 | OMIM |
| 71 | MIR193A | MicroRNA 193a | GeneCards |
| 72 | MIR21 | MicroRNA 21 | GeneCards |
| 73 | MIR30A | MicroRNA 30a | GeneCards |
| 74 | MTNR1B | Melatonin receptor 1B | OMIM |
| 75 | MYH9 | Myosin Heavy Chain 9 | GeneCards |
| 76 | NEUROD1 | Neurogenic differentiation 1 | OMIM |
| 77 | NIDDM2 | Diabetes mellitus, noninsulin-dependent, 2 | OMIM |
| 78 | NIDDM4 | Diabetes mellitus, noninsulin-dependent, 4 | OMIM |
| 79 | NOTCH1 | Notch Receptor 1 | GeneCards |
| 80 | NOTCH4 | Notch Receptor 4 | GeneCards |
| 81 | NPHS1 | NPHS1 Adhesion Molecule, Nephrin | GeneCards |
| 82 | NPHS2 | NPHS2 Stomatin Family Member, Podocin | GeneCards |
| 83 | PAX4 | Paired box homeotic gene-4 | OMIM |
| 84 | PBCA | Pancreatic beta cell, agenesis of | OMIM |
| 85 | PDX1 | Pancreas/duodenum homeobox protein 1 | OMIM |
| 86 | PLAUR | Plasminogen Activator, Urokinase Receptor | GeneCards |
| 87 | PON1 | Paraoxonase-1 | OMIM |
| 88 | PPARG | Peroxisome proliferator activated receptor, gamma | OMIM |
| 89 | PRKCA | Protein Kinase C Alpha | GeneCards |
| 90 | PRKCB | Protein Kinase C Beta | GeneCards |
| 91 | PRKCH | Protein Kinase C Eta | GeneCards |
| 92 | PTPN22 | Protein tyrosine phosphatase, nonreceptor-type 22 | OMIM |
| 93 | PTPRO | Protein Tyrosine Phosphatase Receptor Type O | GeneCards |
| 94 | RAB11B | RAB11B, Member RAS Oncogene Family | GeneCards |
| 95 | RETN | Resistin | OMIM |
| 96 | RHOA | Ras Homolog Family Member A | GeneCards |
| 97 | RRAD | Ras-related associated with diabetes | OMIM |
| 98 | SIRT6 | Sirtuin 6 | GeneCards |
| 99 | SLC2A2 | Solute carrier family 2 (facilitated glucose transporter), member 2 | OMIM |
| 100 | SLC30A8 | Solute carrier family 30 (zinc transporter), member 8 | OMIM |
| 101 | SMPDL3B | Sphingomyelin Phosphodiesterase Acid Like 3B | GeneCards |
| 102 | SOD2 | Superoxide dismutase-2, mitochondrial | OMIM |
| 103 | SPINK1 | Serine protease inhibitor, Kazal type I (pancreatic secretory trypsin inhibitor) | OMIM |
| 104 | SUMO4 | Small ubiquitin-like modifier 4 | OMIM |
| 105 | SYNPO | Synaptopodin | GeneCards |
| 106 | T2D3 | Type 2 diabetes mellitus 3 | OMIM |
| 107 | TBC1D4 | TPC1 domain family, member 4 | OMIM |
| 108 | TCF7L2 | Transcription factor 7-like 2 | OMIM |
| 109 | TGFB1 | Transforming Growth Factor Beta 1 | GeneCards |
| 110 | TNDM1 | Transient neonatal diabetes mellitus 1 | OMIM |
| 111 | TNFRSF1B | TNF Receptor Superfamily Member 1B | GeneCards |
| 112 | TRPC1 | Transient Receptor Potential Cation Channel Subfamily C Member 1 | GeneCards |
| 113 | TRPC6 | Transient Receptor Potential Cation Channel Subfamily C Member 6 | GeneCards |
| 114 | UCP3 | Uncoupling protein-3 | OMIM |
| 115 | VEGF | Vascular endothelial growth factor | OMIM |
| 116 | VEGFA | Vascular Endothelial Growth Factor A | GeneCards |
| 117 | WFS1 | Wolframin | OMIM |
| 118 | YIPF5 | Yip1 domain family, member 5 | OMIM |
| 119 | ZFP36 | ZFP36 Ring Finger Protein | GeneCards |
| 120 | ZFP57 | Zinc finger protein 57, mouse, homolog of | OMIM |
