# Supplementary material for: Cellular mechanism of action of forsythiaside for the treatment of diabetic kidney disease
Source: Front Pharmacol. 2023 Jan 13;13:1096536. doi: 10.3389/fphar.2022.1096536 (PMC9880420; doi:10.3389/fphar.2022.1096536)
Supplement: Supplementary file 5 [file Table4.DOCX]

**Table S4**. Diabetic tubulopathy related targets.

| **Number** | **Gene name** | **Protein name** | **Database 1** | **Database 2** |
| --- | --- | --- | --- | --- |
| 1 | [ABCB1](https://www.genecards.org/cgi-bin/carddisp.pl?gene=ABCB1&keywords=diabetic,tubulopathy) | ATP Binding Cassette Subfamily B Member 1 | GeneCards |  |
| 2 | [ABCB11](https://www.genecards.org/cgi-bin/carddisp.pl?gene=ABCB11&keywords=diabetic,tubulopathy) | ATP Binding Cassette Subfamily B Member 11 | GeneCards |  |
| 3 | [ABCB7](https://www.genecards.org/cgi-bin/carddisp.pl?gene=ABCB7&keywords=diabetic,tubulopathy) | ATP Binding Cassette Subfamily B Member 7 | GeneCards |  |
| 4 | [ABCC2](https://www.genecards.org/cgi-bin/carddisp.pl?gene=ABCC2&keywords=diabetic,tubulopathy) | ATP Binding Cassette Subfamily C Member 2 | GeneCards |  |
| 5 | [ABCC4](https://www.genecards.org/cgi-bin/carddisp.pl?gene=ABCC4&keywords=diabetic,tubulopathy) | ATP Binding Cassette Subfamily C Member 4 | GeneCards |  |
| 6 | ABCC8 | ATP-binding cassette, subfamily C, member 8 (sulfonylurea receptor) | OMIM |  |
| 7 | [ACAD9](https://www.genecards.org/cgi-bin/carddisp.pl?gene=ACAD9&keywords=diabetic,tubulopathy) | Acyl-CoA Dehydrogenase Family Member 9 | GeneCards |  |
| 8 | [ACADM](https://www.genecards.org/cgi-bin/carddisp.pl?gene=ACADM&keywords=diabetic,tubulopathy) | Acyl-CoA Dehydrogenase Medium Chain | GeneCards |  |
| 9 | [ACADS](https://www.genecards.org/cgi-bin/carddisp.pl?gene=ACADS&keywords=diabetic,tubulopathy) | Acyl-CoA Dehydrogenase Short Chain | GeneCards |  |
| 10 | [ACADVL](https://www.genecards.org/cgi-bin/carddisp.pl?gene=ACADVL&keywords=diabetic,tubulopathy) | Acyl-CoA Dehydrogenase Very Long Chain | GeneCards |  |
| 11 | [ACE](https://www.genecards.org/cgi-bin/carddisp.pl?gene=ACE&keywords=diabetic,tubulopathy) | Angiotensin I Converting Enzyme | GeneCards | OMIM |
| 12 | [ACO1](https://www.genecards.org/cgi-bin/carddisp.pl?gene=ACO1&keywords=diabetic,tubulopathy) | Aconitase 1 | GeneCards |  |
| 13 | [ACO2](https://www.genecards.org/cgi-bin/carddisp.pl?gene=ACO2&keywords=diabetic,tubulopathy) | Aconitase 2 | GeneCards |  |
| 14 | [ACOT11](https://www.genecards.org/cgi-bin/carddisp.pl?gene=ACOT11&keywords=diabetic,tubulopathy) | Acyl-CoA Thioesterase 11 | GeneCards |  |
| 15 | [ADAM19](https://www.genecards.org/cgi-bin/carddisp.pl?gene=ADAM19&keywords=diabetic,tubulopathy) | ADAM Metallopeptidase Domain 19 | GeneCards |  |
| 16 | [ADAMTS13](https://www.genecards.org/cgi-bin/carddisp.pl?gene=ADAMTS13&keywords=diabetic,tubulopathy) | ADAM Metallopeptidase With Thrombospondin Type 1 Motif 13 | GeneCards |  |
| 17 | [ADAMTS4](https://www.genecards.org/cgi-bin/carddisp.pl?gene=ADAMTS4&keywords=diabetic,tubulopathy) | ADAM Metallopeptidase With Thrombospondin Type 1 Motif 4 | GeneCards |  |
| 18 | [ADGRG6](https://www.genecards.org/cgi-bin/carddisp.pl?gene=ADGRG6&keywords=diabetic,tubulopathy) | Adhesion G Protein-Coupled Receptor G6 | GeneCards |  |
| 19 | [ADH1A](https://www.genecards.org/cgi-bin/carddisp.pl?gene=ADH1A&keywords=diabetic,tubulopathy) | Alcohol Dehydrogenase 1A (Class I), Alpha Polypeptide | GeneCards |  |
| 20 | [ADI1](https://www.genecards.org/cgi-bin/carddisp.pl?gene=ADI1&keywords=diabetic,tubulopathy) | Acireductone Dioxygenase 1 | GeneCards |  |
| 21 | [AGT](https://www.genecards.org/cgi-bin/carddisp.pl?gene=AGT&keywords=diabetic,tubulopathy) | Angiotensinogen | GeneCards |  |
| 22 | [AGTR1](https://www.genecards.org/cgi-bin/carddisp.pl?gene=AGTR1&keywords=diabetic,tubulopathy) | Angiotensin II Receptor Type 1 | GeneCards |  |
| 23 | [AHSP](https://www.genecards.org/cgi-bin/carddisp.pl?gene=AHSP&keywords=diabetic,tubulopathy) | Alpha Hemoglobin Stabilizing Protein | GeneCards |  |
| 24 | [AKAP4](https://www.genecards.org/cgi-bin/carddisp.pl?gene=AKAP4&keywords=diabetic,tubulopathy) | A-Kinase Anchoring Protein 4 | GeneCards |  |
| 25 | AKT2 | AKT serine/threonine kinase 2 | OMIM |  |
| 26 | [ALB](https://www.genecards.org/cgi-bin/carddisp.pl?gene=ALB&keywords=diabetic,tubulopathy) | Albumin | GeneCards |  |
| 27 | [ALDOB](https://www.genecards.org/cgi-bin/carddisp.pl?gene=ALDOB&keywords=diabetic,tubulopathy) | Aldolase, Fructose-Bisphosphate B | GeneCards |  |
| 28 | [ALG1](https://www.genecards.org/cgi-bin/carddisp.pl?gene=ALG1&keywords=diabetic,tubulopathy) | ALG1 Chitobiosyldiphosphodolichol Beta-Mannosyltransferase | GeneCards |  |
| 29 | [ALG14](https://www.genecards.org/cgi-bin/carddisp.pl?gene=ALG14&keywords=diabetic,tubulopathy) | ALG14 UDP-N-Acetylglucosaminyltransferase Subunit | GeneCards |  |
| 30 | [ALG2](https://www.genecards.org/cgi-bin/carddisp.pl?gene=ALG2&keywords=diabetic,tubulopathy) | ALG2 Alpha-1,3/1,6-Mannosyltransferase | GeneCards |  |
| 31 | [ALX1](https://www.genecards.org/cgi-bin/carddisp.pl?gene=ALX1&keywords=diabetic,tubulopathy) | ALX Homeobox 1 | GeneCards |  |
| 32 | [AMBP](https://www.genecards.org/cgi-bin/carddisp.pl?gene=AMBP&keywords=diabetic,tubulopathy) | Alpha-1-Microglobulin/Bikunin Precursor | GeneCards |  |
| 33 | [AMN](https://www.genecards.org/cgi-bin/carddisp.pl?gene=AMN&keywords=diabetic,tubulopathy) | Amnion Associated Transmembrane Protein | GeneCards |  |
| 34 | [AMPD1](https://www.genecards.org/cgi-bin/carddisp.pl?gene=AMPD1&keywords=diabetic,tubulopathy) | Adenosine Monophosphate Deaminase 1 | GeneCards |  |
| 35 | [ANAPC2](https://www.genecards.org/cgi-bin/carddisp.pl?gene=ANAPC2&keywords=diabetic,tubulopathy) | Anaphase Promoting Complex Subunit 2 | GeneCards |  |
| 36 | [ANKRD12](https://www.genecards.org/cgi-bin/carddisp.pl?gene=ANKRD12&keywords=diabetic,tubulopathy) | Ankyrin Repeat Domain 12 | GeneCards |  |
| 37 | [ANKS4B](https://www.genecards.org/cgi-bin/carddisp.pl?gene=ANKS4B&keywords=diabetic,tubulopathy) | Ankyrin Repeat And Sterile Alpha Motif Domain Containing 4B | GeneCards |  |
| 38 | [ANXA5](https://www.genecards.org/cgi-bin/carddisp.pl?gene=ANXA5&keywords=diabetic,tubulopathy) | Annexin A5 | GeneCards |  |
| 39 | [AP1M1](https://www.genecards.org/cgi-bin/carddisp.pl?gene=AP1M1&keywords=diabetic,tubulopathy) | Adaptor Related Protein Complex 1 Subunit Mu 1 | GeneCards |  |
| 40 | [AP3B1](https://www.genecards.org/cgi-bin/carddisp.pl?gene=AP3B1&keywords=diabetic,tubulopathy) | Adaptor Related Protein Complex 3 Subunit Beta 1 | GeneCards |  |
| 41 | [APOB](https://www.genecards.org/cgi-bin/carddisp.pl?gene=APOB&keywords=diabetic,tubulopathy) | Apolipoprotein B | GeneCards |  |
| 42 | [APPL1](https://www.genecards.org/cgi-bin/carddisp.pl?gene=APPL1&keywords=diabetic,tubulopathy) | Adaptor Protein, Phosphotyrosine Interacting With PH Domain And Leucine Zipper 1 | GeneCards | OMIM |
| 43 | [APTX](https://www.genecards.org/cgi-bin/carddisp.pl?gene=APTX&keywords=diabetic,tubulopathy) | Aprataxin | GeneCards |  |
| 44 | [AQP2](https://www.genecards.org/cgi-bin/carddisp.pl?gene=AQP2&keywords=diabetic,tubulopathy) | Aquaporin 2 | GeneCards | OMIM |
| 45 | [ARHGAP1](https://www.genecards.org/cgi-bin/carddisp.pl?gene=ARHGAP1&keywords=diabetic,tubulopathy) | Rho GTPase Activating Protein 1 | GeneCards |  |
| 46 | [ARRDC4](https://www.genecards.org/cgi-bin/carddisp.pl?gene=ARRDC4&keywords=diabetic,tubulopathy) | Arrestin Domain Containing 4 | GeneCards |  |
| 47 | [ATP13A2](https://www.genecards.org/cgi-bin/carddisp.pl?gene=ATP13A2&keywords=diabetic,tubulopathy) | ATPase Cation Transporting 13A2 | GeneCards |  |
| 48 | [ATP5F1E](https://www.genecards.org/cgi-bin/carddisp.pl?gene=ATP5F1E&keywords=diabetic,tubulopathy) | ATP Synthase F1 Subunit Epsilon | GeneCards |  |
| 49 | [ATP5MG](https://www.genecards.org/cgi-bin/carddisp.pl?gene=ATP5MG&keywords=diabetic,tubulopathy) | ATP Synthase Membrane Subunit G | GeneCards |  |
| 50 | [ATP6V0A4](https://www.genecards.org/cgi-bin/carddisp.pl?gene=ATP6V0A4&keywords=diabetic,tubulopathy) | ATPase H+ Transporting V0 Subunit A4 | GeneCards |  |
| 51 | [ATP6V1B1](https://www.genecards.org/cgi-bin/carddisp.pl?gene=ATP6V1B1&keywords=diabetic,tubulopathy) | ATPase H+ Transporting V1 Subunit B1 | GeneCards |  |
| 52 | [AVP](https://www.genecards.org/cgi-bin/carddisp.pl?gene=AVP&keywords=diabetic,tubulopathy) | Arginine Vasopressin | GeneCards | OMIM |
| 53 | [AVPR2](https://www.genecards.org/cgi-bin/carddisp.pl?gene=AVPR2&keywords=diabetic,tubulopathy) | Arginine Vasopressin Receptor 2 | GeneCards | OMIM |
| 54 | [B2M](https://www.genecards.org/cgi-bin/carddisp.pl?gene=B2M&keywords=diabetic,tubulopathy) | Beta-2-Microglobulin | GeneCards |  |
| 55 | [B4GALT1](https://www.genecards.org/cgi-bin/carddisp.pl?gene=B4GALT1&keywords=diabetic,tubulopathy) | Beta-1,4-Galactosyltransferase 1 | GeneCards |  |
| 56 | [BCL11A](https://www.genecards.org/cgi-bin/carddisp.pl?gene=BCL11A&keywords=diabetic,tubulopathy) | BAF Chromatin Remodeling Complex Subunit BCL11A | GeneCards |  |
| 57 | [BCL2L13](https://www.genecards.org/cgi-bin/carddisp.pl?gene=BCL2L13&keywords=diabetic,tubulopathy) | BCL2 Like 13 | GeneCards |  |
| 58 | [BCS1L](https://www.genecards.org/cgi-bin/carddisp.pl?gene=BCS1L&keywords=diabetic,tubulopathy) | BCS1 Homolog, Ubiquinol-Cytochrome C Reductase Complex Chaperone | GeneCards |  |
| 59 | [BGLAP](https://www.genecards.org/cgi-bin/carddisp.pl?gene=BGLAP&keywords=diabetic,tubulopathy) | Bone Gamma-Carboxyglutamate Protein | GeneCards |  |
| 60 | BLK | BLK protooncogene, SRC family tyrosinase kinase | OMIM |  |
| 61 | [BLOC1S1](https://www.genecards.org/cgi-bin/carddisp.pl?gene=BLOC1S1&keywords=diabetic,tubulopathy) | Biogenesis Of Lysosomal Organelles Complex 1 Subunit 1 | GeneCards |  |
| 62 | [BSND](https://www.genecards.org/cgi-bin/carddisp.pl?gene=BSND&keywords=diabetic,tubulopathy) | Barttin CLCNK Type Accessory Subunit Beta | GeneCards |  |
| 63 | [C1orf112](https://www.genecards.org/cgi-bin/carddisp.pl?gene=C1orf112&keywords=diabetic,tubulopathy) | Chromosome 1 Open Reading Frame 112 | GeneCards |  |
| 64 | [CA2](https://www.genecards.org/cgi-bin/carddisp.pl?gene=CA2&keywords=diabetic,tubulopathy) | Carbonic Anhydrase 2 | GeneCards |  |
| 65 | [CABIN1](https://www.genecards.org/cgi-bin/carddisp.pl?gene=CABIN1&keywords=diabetic,tubulopathy) | Calcineurin Binding Protein 1 | GeneCards |  |
| 66 | CAPN10 | Calpain-10 | OMIM |  |
| 67 | [CAPN8](https://www.genecards.org/cgi-bin/carddisp.pl?gene=CAPN8&keywords=diabetic,tubulopathy) | Calpain 8 | GeneCards |  |
| 68 | [CASR](https://www.genecards.org/cgi-bin/carddisp.pl?gene=CASR&keywords=diabetic,tubulopathy) | Calcium Sensing Receptor | GeneCards |  |
| 69 | CCR5 | Chemokine (C-C) receptor 5 | OMIM |  |
| 70 | [CD151](https://www.genecards.org/cgi-bin/carddisp.pl?gene=CD151&keywords=diabetic,tubulopathy) | CD151 Molecule (Raph Blood Group) | GeneCards |  |
| 71 | [CD4](https://www.genecards.org/cgi-bin/carddisp.pl?gene=CD4&keywords=diabetic,tubulopathy) | CD4 Molecule | GeneCards |  |
| 72 | [CD59](https://www.genecards.org/cgi-bin/carddisp.pl?gene=CD59&keywords=diabetic,tubulopathy) | CD59 Molecule (CD59 Blood Group) | GeneCards |  |
| 73 | [CDC42](https://www.genecards.org/cgi-bin/carddisp.pl?gene=CDC42&keywords=diabetic,tubulopathy) | Cell Division Cycle 42 | GeneCards |  |
| 74 | [CDK5RAP1](https://www.genecards.org/cgi-bin/carddisp.pl?gene=CDK5RAP1&keywords=diabetic,tubulopathy) | CDK5 Regulatory Subunit Associated Protein 1 | GeneCards |  |
| 75 | [CDKN3](https://www.genecards.org/cgi-bin/carddisp.pl?gene=CDKN3&keywords=diabetic,tubulopathy) | Cyclin Dependent Kinase Inhibitor 3 | GeneCards |  |
| 76 | CEL | Carboxyl-ester lipase (bile-salt stimulated lipase) | OMIM |  |
| 77 | [CFTR](https://www.genecards.org/cgi-bin/carddisp.pl?gene=CFTR&keywords=diabetic,tubulopathy) | CF Transmembrane Conductance Regulator | GeneCards |  |
| 78 | [CHCHD2](https://www.genecards.org/cgi-bin/carddisp.pl?gene=CHCHD2&keywords=diabetic,tubulopathy) | Coiled-Coil-Helix-Coiled-Coil-Helix Domain Containing 2 | GeneCards |  |
| 79 | [CHIT1](https://www.genecards.org/cgi-bin/carddisp.pl?gene=CHIT1&keywords=diabetic,tubulopathy) | Chitinase 1 | GeneCards |  |
| 80 | [CHKA](https://www.genecards.org/cgi-bin/carddisp.pl?gene=CHKA&keywords=diabetic,tubulopathy) | Choline Kinase Alpha | GeneCards |  |
| 81 | [CKMT1A](https://www.genecards.org/cgi-bin/carddisp.pl?gene=CKMT1A&keywords=diabetic,tubulopathy) | Creatine Kinase, Mitochondrial 1A | GeneCards |  |
| 82 | [CKMT1B](https://www.genecards.org/cgi-bin/carddisp.pl?gene=CKMT1B&keywords=diabetic,tubulopathy) | Creatine Kinase, Mitochondrial 1B | GeneCards |  |
| 83 | [CLCN1](https://www.genecards.org/cgi-bin/carddisp.pl?gene=CLCN1&keywords=diabetic,tubulopathy) | Chloride Voltage-Gated Channel 1 | GeneCards |  |
| 84 | [CLCN3](https://www.genecards.org/cgi-bin/carddisp.pl?gene=CLCN3&keywords=diabetic,tubulopathy) | Chloride Voltage-Gated Channel 3 | GeneCards |  |
| 85 | [CLCN4](https://www.genecards.org/cgi-bin/carddisp.pl?gene=CLCN4&keywords=diabetic,tubulopathy) | Chloride Voltage-Gated Channel 4 | GeneCards |  |
| 86 | [CLCN5](https://www.genecards.org/cgi-bin/carddisp.pl?gene=CLCN5&keywords=diabetic,tubulopathy) | Chloride Voltage-Gated Channel 5 | GeneCards |  |
| 87 | [CLCN6](https://www.genecards.org/cgi-bin/carddisp.pl?gene=CLCN6&keywords=diabetic,tubulopathy) | Chloride Voltage-Gated Channel 6 | GeneCards |  |
| 88 | [CLCN7](https://www.genecards.org/cgi-bin/carddisp.pl?gene=CLCN7&keywords=diabetic,tubulopathy) | Chloride Voltage-Gated Channel 7 | GeneCards |  |
| 89 | [CLCNKA](https://www.genecards.org/cgi-bin/carddisp.pl?gene=CLCNKA&keywords=diabetic,tubulopathy) | Chloride Voltage-Gated Channel Ka | GeneCards |  |
| 90 | [CLCNKB](https://www.genecards.org/cgi-bin/carddisp.pl?gene=CLCNKB&keywords=diabetic,tubulopathy) | Chloride Voltage-Gated Channel Kb | GeneCards |  |
| 91 | [CLDN10](https://www.genecards.org/cgi-bin/carddisp.pl?gene=CLDN10&keywords=diabetic,tubulopathy) | Claudin 10 | GeneCards |  |
| 92 | [CLDN16](https://www.genecards.org/cgi-bin/carddisp.pl?gene=CLDN16&keywords=diabetic,tubulopathy) | Claudin 16 | GeneCards |  |
| 93 | [CLDN19](https://www.genecards.org/cgi-bin/carddisp.pl?gene=CLDN19&keywords=diabetic,tubulopathy) | Claudin 19 | GeneCards |  |
| 94 | [CLTRN](https://www.genecards.org/cgi-bin/carddisp.pl?gene=CLTRN&keywords=diabetic,tubulopathy) | Collectrin, Amino Acid Transport Regulator | GeneCards |  |
| 95 | [CNNM2](https://www.genecards.org/cgi-bin/carddisp.pl?gene=CNNM2&keywords=diabetic,tubulopathy) | Cyclin And CBS Domain Divalent Metal Cation Transport Mediator 2 | GeneCards |  |
| 96 | [CNTN3](https://www.genecards.org/cgi-bin/carddisp.pl?gene=CNTN3&keywords=diabetic,tubulopathy) | Contactin 3 | GeneCards |  |
| 97 | [COA3](https://www.genecards.org/cgi-bin/carddisp.pl?gene=COA3&keywords=diabetic,tubulopathy) | Cytochrome C Oxidase Assembly Factor 3 | GeneCards |  |
| 98 | [COG2](https://www.genecards.org/cgi-bin/carddisp.pl?gene=COG2&keywords=diabetic,tubulopathy) | Component Of Oligomeric Golgi Complex 2 | GeneCards |  |
| 99 | [COG4](https://www.genecards.org/cgi-bin/carddisp.pl?gene=COG4&keywords=diabetic,tubulopathy) | Component Of Oligomeric Golgi Complex 4 | GeneCards |  |
| 100 | [COG6](https://www.genecards.org/cgi-bin/carddisp.pl?gene=COG6&keywords=diabetic,tubulopathy) | Component Of Oligomeric Golgi Complex 6 | GeneCards |  |
| 101 | [COG7](https://www.genecards.org/cgi-bin/carddisp.pl?gene=COG7&keywords=diabetic,tubulopathy) | Component Of Oligomeric Golgi Complex 7 | GeneCards |  |
| 102 | [COPB2](https://www.genecards.org/cgi-bin/carddisp.pl?gene=COPB2&keywords=diabetic,tubulopathy) | COPI Coat Complex Subunit Beta 2 | GeneCards |  |
| 103 | [COQ2](https://www.genecards.org/cgi-bin/carddisp.pl?gene=COQ2&keywords=diabetic,tubulopathy) | Coenzyme Q2, Polyprenyltransferase | GeneCards |  |
| 104 | [COQ3](https://www.genecards.org/cgi-bin/carddisp.pl?gene=COQ3&keywords=diabetic,tubulopathy) | Coenzyme Q3, Methyltransferase | GeneCards |  |
| 105 | [COQ4](https://www.genecards.org/cgi-bin/carddisp.pl?gene=COQ4&keywords=diabetic,tubulopathy) | Coenzyme Q4 | GeneCards |  |
| 106 | [COQ5](https://www.genecards.org/cgi-bin/carddisp.pl?gene=COQ5&keywords=diabetic,tubulopathy) | Coenzyme Q5, Methyltransferase | GeneCards |  |
| 107 | [COQ6](https://www.genecards.org/cgi-bin/carddisp.pl?gene=COQ6&keywords=diabetic,tubulopathy) | Coenzyme Q6, Monooxygenase | GeneCards |  |
| 108 | [COQ7](https://www.genecards.org/cgi-bin/carddisp.pl?gene=COQ7&keywords=diabetic,tubulopathy) | Coenzyme Q7, Hydroxylase | GeneCards |  |
| 109 | [COQ8A](https://www.genecards.org/cgi-bin/carddisp.pl?gene=COQ8A&keywords=diabetic,tubulopathy) | Coenzyme Q8A | GeneCards |  |
| 110 | [COQ8B](https://www.genecards.org/cgi-bin/carddisp.pl?gene=COQ8B&keywords=diabetic,tubulopathy) | Coenzyme Q8B | GeneCards |  |
| 111 | [COQ9](https://www.genecards.org/cgi-bin/carddisp.pl?gene=COQ9&keywords=diabetic,tubulopathy) | Coenzyme Q9 | GeneCards |  |
| 112 | [COX10](https://www.genecards.org/cgi-bin/carddisp.pl?gene=COX10&keywords=diabetic,tubulopathy) | Cytochrome C Oxidase Assembly Factor Heme A:Farnesyltransferase COX10 | GeneCards |  |
| 113 | [COX15](https://www.genecards.org/cgi-bin/carddisp.pl?gene=COX15&keywords=diabetic,tubulopathy) | Cytochrome C Oxidase Assembly Homolog COX15 | GeneCards |  |
| 114 | [COX4I1](https://www.genecards.org/cgi-bin/carddisp.pl?gene=COX4I1&keywords=diabetic,tubulopathy) | Cytochrome C Oxidase Subunit 4I1 | GeneCards |  |
| 115 | [COX5A](https://www.genecards.org/cgi-bin/carddisp.pl?gene=COX5A&keywords=diabetic,tubulopathy) | Cytochrome C Oxidase Subunit 5A | GeneCards |  |
| 116 | [COX5B](https://www.genecards.org/cgi-bin/carddisp.pl?gene=COX5B&keywords=diabetic,tubulopathy) | Cytochrome C Oxidase Subunit 5B | GeneCards |  |
| 117 | [COX6B1](https://www.genecards.org/cgi-bin/carddisp.pl?gene=COX6B1&keywords=diabetic,tubulopathy) | Cytochrome C Oxidase Subunit 6B1 | GeneCards |  |
| 118 | [COX6C](https://www.genecards.org/cgi-bin/carddisp.pl?gene=COX6C&keywords=diabetic,tubulopathy) | Cytochrome C Oxidase Subunit 6C | GeneCards |  |
| 119 | [COX7C](https://www.genecards.org/cgi-bin/carddisp.pl?gene=COX7C&keywords=diabetic,tubulopathy) | Cytochrome C Oxidase Subunit 7C | GeneCards |  |
| 120 | [COX8A](https://www.genecards.org/cgi-bin/carddisp.pl?gene=COX8A&keywords=diabetic,tubulopathy) | Cytochrome C Oxidase Subunit 8A | GeneCards |  |
| 121 | [CPT1B](https://www.genecards.org/cgi-bin/carddisp.pl?gene=CPT1B&keywords=diabetic,tubulopathy) | Carnitine Palmitoyltransferase 1B | GeneCards |  |
| 122 | [CPT2](https://www.genecards.org/cgi-bin/carddisp.pl?gene=CPT2&keywords=diabetic,tubulopathy) | Carnitine Palmitoyltransferase 2 | GeneCards |  |
| 123 | [CREB3L4](https://www.genecards.org/cgi-bin/carddisp.pl?gene=CREB3L4&keywords=diabetic,tubulopathy) | CAMP Responsive Element Binding Protein 3 Like 4 | GeneCards |  |
| 124 | [CRP](https://www.genecards.org/cgi-bin/carddisp.pl?gene=CRP&keywords=diabetic,tubulopathy) | C-Reactive Protein | GeneCards |  |
| 125 | [CS](https://www.genecards.org/cgi-bin/carddisp.pl?gene=CS&keywords=diabetic,tubulopathy) | Citrate Synthase | GeneCards |  |
| 126 | [CST3](https://www.genecards.org/cgi-bin/carddisp.pl?gene=CST3&keywords=diabetic,tubulopathy) | Cystatin C | GeneCards |  |
| 127 | CTLA4 | Cytotoxic T-lymphocyte-associated serine esterase-4 | OMIM |  |
| 128 | [CTNNB1](https://www.genecards.org/cgi-bin/carddisp.pl?gene=CTNNB1&keywords=diabetic,tubulopathy) | Catenin Beta 1 | GeneCards |  |
| 129 | [CTNS](https://www.genecards.org/cgi-bin/carddisp.pl?gene=CTNS&keywords=diabetic,tubulopathy) | Cystinosin, Lysosomal Cystine Transporter | GeneCards |  |
| 130 | [CTRL](https://www.genecards.org/cgi-bin/carddisp.pl?gene=CTRL&keywords=diabetic,tubulopathy) | Chymotrypsin Like | GeneCards |  |
| 131 | [CUBN](https://www.genecards.org/cgi-bin/carddisp.pl?gene=CUBN&keywords=diabetic,tubulopathy) | Cubilin | GeneCards |  |
| 132 | [CWF19L1](https://www.genecards.org/cgi-bin/carddisp.pl?gene=CWF19L1&keywords=diabetic,tubulopathy) | CWF19 Like Cell Cycle Control Factor 1 | GeneCards |  |
| 133 | [CXCL8](https://www.genecards.org/cgi-bin/carddisp.pl?gene=CXCL8&keywords=diabetic,tubulopathy) | C-X-C Motif Chemokine Ligand 8 | GeneCards |  |
| 134 | [CYC1](https://www.genecards.org/cgi-bin/carddisp.pl?gene=CYC1&keywords=diabetic,tubulopathy) | Cytochrome C1 | GeneCards |  |
| 135 | [CYCS](https://www.genecards.org/cgi-bin/carddisp.pl?gene=CYCS&keywords=diabetic,tubulopathy) | Cytochrome C, Somatic | GeneCards |  |
| 136 | [DACT3](https://www.genecards.org/cgi-bin/carddisp.pl?gene=DACT3&keywords=diabetic,tubulopathy) | Dishevelled Binding Antagonist Of Beta Catenin 3 | GeneCards |  |
| 137 | [DALRD3](https://www.genecards.org/cgi-bin/carddisp.pl?gene=DALRD3&keywords=diabetic,tubulopathy) | DALR Anticodon Binding Domain Containing 3 | GeneCards |  |
| 138 | [DARS2](https://www.genecards.org/cgi-bin/carddisp.pl?gene=DARS2&keywords=diabetic,tubulopathy) | Aspartyl-TRNA Synthetase 2, Mitochondrial | GeneCards |  |
| 139 | [DDAH2](https://www.genecards.org/cgi-bin/carddisp.pl?gene=DDAH2&keywords=diabetic,tubulopathy) | Dimethylarginine Dimethylaminohydrolase 2 | GeneCards |  |
| 140 | [DENND4B](https://www.genecards.org/cgi-bin/carddisp.pl?gene=DENND4B&keywords=diabetic,tubulopathy) | DENN Domain Containing 4B | GeneCards |  |
| 141 | [DGUOK](https://www.genecards.org/cgi-bin/carddisp.pl?gene=DGUOK&keywords=diabetic,tubulopathy) | Deoxyguanosine Kinase | GeneCards |  |
| 142 | [DMP1](https://www.genecards.org/cgi-bin/carddisp.pl?gene=DMP1&keywords=diabetic,tubulopathy) | Dentin Matrix Acidic Phosphoprotein 1 | GeneCards |  |
| 143 | [DNAH10OS](https://www.genecards.org/cgi-bin/carddisp.pl?gene=DNAH10OS&keywords=diabetic,tubulopathy) | Dynein Axonemal Heavy Chain 10 Opposite Strand | GeneCards |  |
| 144 | DNAJC3 | DnaJ, E. coli, homolog of, subfamily C, member 3 (protein kinase inhibitor p58) | OMIM |  |
| 145 | [EBF3](https://www.genecards.org/cgi-bin/carddisp.pl?gene=EBF3&keywords=diabetic,tubulopathy) | EBF Transcription Factor 3 | GeneCards |  |
| 146 | [EDARADD](https://www.genecards.org/cgi-bin/carddisp.pl?gene=EDARADD&keywords=diabetic,tubulopathy) | EDAR Associated Death Domain | GeneCards |  |
| 147 | [EGID-105180392](https://www.genecards.org/cgi-bin/carddisp.pl?gene=EGID-105180392&keywords=diabetic,tubulopathy) | Beta-Globin 3' Hypersensitive Site 1 | GeneCards |  |
| 148 | [EHHADH](https://www.genecards.org/cgi-bin/carddisp.pl?gene=EHHADH&keywords=diabetic,tubulopathy) | Enoyl-CoA Hydratase And 3-Hydroxyacyl CoA Dehydrogenase | GeneCards |  |
| 149 | [EIF2AK1](https://www.genecards.org/cgi-bin/carddisp.pl?gene=EIF2AK1&keywords=diabetic,tubulopathy) | Eukaryotic Translation Initiation Factor 2 Alpha Kinase 1 | GeneCards |  |
| 150 | [EIF2S1](https://www.genecards.org/cgi-bin/carddisp.pl?gene=EIF2S1&keywords=diabetic,tubulopathy) | Eukaryotic Translation Initiation Factor 2 Subunit Alpha | GeneCards |  |
| 151 | [ELAC2](https://www.genecards.org/cgi-bin/carddisp.pl?gene=ELAC2&keywords=diabetic,tubulopathy) | ElaC Ribonuclease Z 2 | GeneCards |  |
| 152 | [ENPP1](https://www.genecards.org/cgi-bin/carddisp.pl?gene=ENPP1&keywords=diabetic,tubulopathy) | Ectonucleotide Pyrophosphatase/Phosphodiesterase 1 | GeneCards |  |
| 153 | [ENTPD1](https://www.genecards.org/cgi-bin/carddisp.pl?gene=ENTPD1&keywords=diabetic,tubulopathy) | Ectonucleoside Triphosphate Diphosphohydrolase 1 | GeneCards |  |
| 154 | [EPO](https://www.genecards.org/cgi-bin/carddisp.pl?gene=EPO&keywords=diabetic,tubulopathy) | Erythropoietin | GeneCards | OMIM |
| 155 | [EPOR](https://www.genecards.org/cgi-bin/carddisp.pl?gene=EPOR&keywords=diabetic,tubulopathy) | Erythropoietin Receptor | GeneCards |  |
| 156 | [EPRS1](https://www.genecards.org/cgi-bin/carddisp.pl?gene=EPRS1&keywords=diabetic,tubulopathy) | Glutamyl-Prolyl-TRNA Synthetase 1 | GeneCards |  |
| 157 | [ERCC1](https://www.genecards.org/cgi-bin/carddisp.pl?gene=ERCC1&keywords=diabetic,tubulopathy) | ERCC Excision Repair 1, Endonuclease Non-Catalytic Subunit | GeneCards |  |
| 158 | [ERCC8](https://www.genecards.org/cgi-bin/carddisp.pl?gene=ERCC8&keywords=diabetic,tubulopathy) | ERCC Excision Repair 8, CSA Ubiquitin Ligase Complex Subunit | GeneCards |  |
| 159 | [ERFE](https://www.genecards.org/cgi-bin/carddisp.pl?gene=ERFE&keywords=diabetic,tubulopathy) | Erythroferrone | GeneCards |  |
| 160 | [ETFA](https://www.genecards.org/cgi-bin/carddisp.pl?gene=ETFA&keywords=diabetic,tubulopathy) | Electron Transfer Flavoprotein Subunit Alpha | GeneCards |  |
| 161 | [ETFB](https://www.genecards.org/cgi-bin/carddisp.pl?gene=ETFB&keywords=diabetic,tubulopathy) | Electron Transfer Flavoprotein Subunit Beta | GeneCards |  |
| 162 | [ETFDH](https://www.genecards.org/cgi-bin/carddisp.pl?gene=ETFDH&keywords=diabetic,tubulopathy) | Electron Transfer Flavoprotein Dehydrogenase | GeneCards |  |
| 163 | [F2](https://www.genecards.org/cgi-bin/carddisp.pl?gene=F2&keywords=diabetic,tubulopathy) | Coagulation Factor II, Thrombin | GeneCards |  |
| 164 | [F5](https://www.genecards.org/cgi-bin/carddisp.pl?gene=F5&keywords=diabetic,tubulopathy) | Coagulation Factor V | GeneCards |  |
| 165 | [F9](https://www.genecards.org/cgi-bin/carddisp.pl?gene=F9&keywords=diabetic,tubulopathy) | Coagulation Factor IX | GeneCards |  |
| 166 | [FAAP100](https://www.genecards.org/cgi-bin/carddisp.pl?gene=FAAP100&keywords=diabetic,tubulopathy) | FA Core Complex Associated Protein 100 | GeneCards |  |
| 167 | [FAH](https://www.genecards.org/cgi-bin/carddisp.pl?gene=FAH&keywords=diabetic,tubulopathy) | Fumarylacetoacetate Hydrolase | GeneCards |  |
| 168 | [FAM186B](https://www.genecards.org/cgi-bin/carddisp.pl?gene=FAM186B&keywords=diabetic,tubulopathy) | Family With Sequence Similarity 186 Member B | GeneCards |  |
| 169 | [FAM71E2](https://www.genecards.org/cgi-bin/carddisp.pl?gene=FAM71E2&keywords=diabetic,tubulopathy) | Family With Sequence Similarity 71 Member E2 | GeneCards |  |
| 170 | [FANCA](https://www.genecards.org/cgi-bin/carddisp.pl?gene=FANCA&keywords=diabetic,tubulopathy) | FA Complementation Group A | GeneCards |  |
| 171 | [FANCB](https://www.genecards.org/cgi-bin/carddisp.pl?gene=FANCB&keywords=diabetic,tubulopathy) | FA Complementation Group B | GeneCards |  |
| 172 | [FANCD2](https://www.genecards.org/cgi-bin/carddisp.pl?gene=FANCD2&keywords=diabetic,tubulopathy) | FA Complementation Group D2 | GeneCards |  |
| 173 | [FANCE](https://www.genecards.org/cgi-bin/carddisp.pl?gene=FANCE&keywords=diabetic,tubulopathy) | FA Complementation Group E | GeneCards |  |
| 174 | [FANCG](https://www.genecards.org/cgi-bin/carddisp.pl?gene=FANCG&keywords=diabetic,tubulopathy) | FA Complementation Group G | GeneCards |  |
| 175 | [FANCI](https://www.genecards.org/cgi-bin/carddisp.pl?gene=FANCI&keywords=diabetic,tubulopathy) | FA Complementation Group I | GeneCards |  |
| 176 | [FANCL](https://www.genecards.org/cgi-bin/carddisp.pl?gene=FANCL&keywords=diabetic,tubulopathy) | FA Complementation Group L | GeneCards |  |
| 177 | [FANCM](https://www.genecards.org/cgi-bin/carddisp.pl?gene=FANCM&keywords=diabetic,tubulopathy) | FA Complementation Group M | GeneCards |  |
| 178 | [FARS2](https://www.genecards.org/cgi-bin/carddisp.pl?gene=FARS2&keywords=diabetic,tubulopathy) | Phenylalanyl-TRNA Synthetase 2, Mitochondrial | GeneCards |  |
| 179 | [FASTKD2](https://www.genecards.org/cgi-bin/carddisp.pl?gene=FASTKD2&keywords=diabetic,tubulopathy) | FAST Kinase Domains 2 | GeneCards |  |
| 180 | [FAT1](https://www.genecards.org/cgi-bin/carddisp.pl?gene=FAT1&keywords=diabetic,tubulopathy) | FAT Atypical Cadherin 1 | GeneCards |  |
| 181 | [FBXL4](https://www.genecards.org/cgi-bin/carddisp.pl?gene=FBXL4&keywords=diabetic,tubulopathy) | F-Box And Leucine Rich Repeat Protein 4 | GeneCards |  |
| 182 | [FCGR3A](https://www.genecards.org/cgi-bin/carddisp.pl?gene=FCGR3A&keywords=diabetic,tubulopathy) | Fc Fragment Of IgG Receptor IIIa | GeneCards |  |
| 183 | [FGF21](https://www.genecards.org/cgi-bin/carddisp.pl?gene=FGF21&keywords=diabetic,tubulopathy) | Fibroblast Growth Factor 21 | GeneCards |  |
| 184 | [FGF23](https://www.genecards.org/cgi-bin/carddisp.pl?gene=FGF23&keywords=diabetic,tubulopathy) | Fibroblast Growth Factor 23 | GeneCards |  |
| 185 | [FHL5](https://www.genecards.org/cgi-bin/carddisp.pl?gene=FHL5&keywords=diabetic,tubulopathy) | Four And A Half LIM Domains 5 | GeneCards |  |
| 186 | [FLAD1](https://www.genecards.org/cgi-bin/carddisp.pl?gene=FLAD1&keywords=diabetic,tubulopathy) | Flavin Adenine Dinucleotide Synthetase 1 | GeneCards |  |
| 187 | FOXC2 | Forkhead box C2 | OMIM |  |
| 188 | [FOXL2](https://www.genecards.org/cgi-bin/carddisp.pl?gene=FOXL2&keywords=diabetic,tubulopathy) | Forkhead Box L2 | GeneCards |  |
| 189 | [FOXRED1](https://www.genecards.org/cgi-bin/carddisp.pl?gene=FOXRED1&keywords=diabetic,tubulopathy) | FAD Dependent Oxidoreductase Domain Containing 1 | GeneCards |  |
| 190 | [FTH1](https://www.genecards.org/cgi-bin/carddisp.pl?gene=FTH1&keywords=diabetic,tubulopathy) | Ferritin Heavy Chain 1 | GeneCards |  |
| 191 | [FTL](https://www.genecards.org/cgi-bin/carddisp.pl?gene=FTL&keywords=diabetic,tubulopathy) | Ferritin Light Chain | GeneCards |  |
| 192 | [FXN](https://www.genecards.org/cgi-bin/carddisp.pl?gene=FXN&keywords=diabetic,tubulopathy) | Frataxin | GeneCards |  |
| 193 | [FXYD2](https://www.genecards.org/cgi-bin/carddisp.pl?gene=FXYD2&keywords=diabetic,tubulopathy) | FXYD Domain Containing Ion Transport Regulator 2 | GeneCards |  |
| 194 | [FXYD7](https://www.genecards.org/cgi-bin/carddisp.pl?gene=FXYD7&keywords=diabetic,tubulopathy) | FXYD Domain Containing Ion Transport Regulator 7 | GeneCards |  |
| 195 | [G6PD](https://www.genecards.org/cgi-bin/carddisp.pl?gene=G6PD&keywords=diabetic,tubulopathy) | Glucose-6-Phosphate Dehydrogenase | GeneCards |  |
| 196 | [GAMT](https://www.genecards.org/cgi-bin/carddisp.pl?gene=GAMT&keywords=diabetic,tubulopathy) | Guanidinoacetate N-Methyltransferase | GeneCards |  |
| 197 | [GARS1](https://www.genecards.org/cgi-bin/carddisp.pl?gene=GARS1&keywords=diabetic,tubulopathy) | Glycyl-TRNA Synthetase 1 | GeneCards |  |
| 198 | [GAS1](https://www.genecards.org/cgi-bin/carddisp.pl?gene=GAS1&keywords=diabetic,tubulopathy) | Growth Arrest Specific 1 | GeneCards |  |
| 199 | [GATA1](https://www.genecards.org/cgi-bin/carddisp.pl?gene=GATA1&keywords=diabetic,tubulopathy) | GATA Binding Protein 1 | GeneCards |  |
| 200 | [GATA2](https://www.genecards.org/cgi-bin/carddisp.pl?gene=GATA2&keywords=diabetic,tubulopathy) | GATA Binding Protein 2 | GeneCards |  |
| 201 | [GATM](https://www.genecards.org/cgi-bin/carddisp.pl?gene=GATM&keywords=diabetic,tubulopathy) | Glycine Amidinotransferase | GeneCards |  |
| 202 | [GC](https://www.genecards.org/cgi-bin/carddisp.pl?gene=GC&keywords=diabetic,tubulopathy) | GC Vitamin D Binding Protein | GeneCards |  |
| 203 | [GCDH](https://www.genecards.org/cgi-bin/carddisp.pl?gene=GCDH&keywords=diabetic,tubulopathy) | Glutaryl-CoA Dehydrogenase | GeneCards |  |
| 204 | GCK | Glucokinase (hexokinase-4) | OMIM |  |
| 205 | [GDF15](https://www.genecards.org/cgi-bin/carddisp.pl?gene=GDF15&keywords=diabetic,tubulopathy) | Growth Differentiation Factor 15 | GeneCards |  |
| 206 | [GGT1](https://www.genecards.org/cgi-bin/carddisp.pl?gene=GGT1&keywords=diabetic,tubulopathy) | Gamma-Glutamyltransferase 1 | GeneCards |  |
| 207 | [GIGYF2](https://www.genecards.org/cgi-bin/carddisp.pl?gene=GIGYF2&keywords=diabetic,tubulopathy) | GRB10 Interacting GYF Protein 2 | GeneCards |  |
| 208 | [GJA1](https://www.genecards.org/cgi-bin/carddisp.pl?gene=GJA1&keywords=diabetic,tubulopathy) | Gap Junction Protein Alpha 1 | GeneCards |  |
| 209 | GLIS3 | GLIS family zinc finger protein 3 | OMIM |  |
| 210 | GPD2 | Glycerol-3-phosphate dehydrogenase 2 (mitochondrial) | OMIM |  |
| 211 | [GPR22](https://www.genecards.org/cgi-bin/carddisp.pl?gene=GPR22&keywords=diabetic,tubulopathy) | G Protein-Coupled Receptor 22 | GeneCards |  |
| 212 | [GPT](https://www.genecards.org/cgi-bin/carddisp.pl?gene=GPT&keywords=diabetic,tubulopathy) | Glutamic--Pyruvic Transaminase | GeneCards |  |
| 213 | [GRHPR](https://www.genecards.org/cgi-bin/carddisp.pl?gene=GRHPR&keywords=diabetic,tubulopathy) | Glyoxylate And Hydroxypyruvate Reductase | GeneCards |  |
| 214 | [GRM1](https://www.genecards.org/cgi-bin/carddisp.pl?gene=GRM1&keywords=diabetic,tubulopathy) | Glutamate Metabotropic Receptor 1 | GeneCards |  |
| 215 | [GSR](https://www.genecards.org/cgi-bin/carddisp.pl?gene=GSR&keywords=diabetic,tubulopathy) | Glutathione-Disulfide Reductase | GeneCards |  |
| 216 | [GSTM1](https://www.genecards.org/cgi-bin/carddisp.pl?gene=GSTM1&keywords=diabetic,tubulopathy) | Glutathione S-Transferase Mu 1 | GeneCards |  |
| 217 | [GSTP1](https://www.genecards.org/cgi-bin/carddisp.pl?gene=GSTP1&keywords=diabetic,tubulopathy) | Glutathione S-Transferase Pi 1 | GeneCards |  |
| 218 | [GSTT1](https://www.genecards.org/cgi-bin/carddisp.pl?gene=GSTT1&keywords=diabetic,tubulopathy) | Glutathione S-Transferase Theta 1 | GeneCards |  |
| 219 | [GTPBP3](https://www.genecards.org/cgi-bin/carddisp.pl?gene=GTPBP3&keywords=diabetic,tubulopathy) | GTP Binding Protein 3, Mitochondrial | GeneCards |  |
| 220 | [H1-1](https://www.genecards.org/cgi-bin/carddisp.pl?gene=H1-1&keywords=diabetic,tubulopathy) | H1.1 Linker Histone, Cluster Member | GeneCards |  |
| 221 | [H2AC7](https://www.genecards.org/cgi-bin/carddisp.pl?gene=H2AC7&keywords=diabetic,tubulopathy) | H2A Clustered Histone 7 | GeneCards |  |
| 222 | [HADH](https://www.genecards.org/cgi-bin/carddisp.pl?gene=HADH&keywords=diabetic,tubulopathy) | Hydroxyacyl-CoA Dehydrogenase | GeneCards |  |
| 223 | [HADHA](https://www.genecards.org/cgi-bin/carddisp.pl?gene=HADHA&keywords=diabetic,tubulopathy) | Hydroxyacyl-CoA Dehydrogenase Trifunctional Multienzyme Complex Subunit Alpha | GeneCards |  |
| 224 | [HAMP](https://www.genecards.org/cgi-bin/carddisp.pl?gene=HAMP&keywords=diabetic,tubulopathy) | Hepcidin Antimicrobial Peptide | GeneCards |  |
| 225 | [HBA1](https://www.genecards.org/cgi-bin/carddisp.pl?gene=HBA1&keywords=diabetic,tubulopathy) | Hemoglobin Subunit Alpha 1 | GeneCards |  |
| 226 | [HBA2](https://www.genecards.org/cgi-bin/carddisp.pl?gene=HBA2&keywords=diabetic,tubulopathy) | Hemoglobin Subunit Alpha 2 | GeneCards |  |
| 227 | [HBB](https://www.genecards.org/cgi-bin/carddisp.pl?gene=HBB&keywords=diabetic,tubulopathy) | Hemoglobin Subunit Beta | GeneCards |  |
| 228 | [HBB-LCR](https://www.genecards.org/cgi-bin/carddisp.pl?gene=HBB-LCR&keywords=diabetic,tubulopathy) | Beta-Globin Locus Control Region | GeneCards |  |
| 229 | [HBD](https://www.genecards.org/cgi-bin/carddisp.pl?gene=HBD&keywords=diabetic,tubulopathy) | Hemoglobin Subunit Delta | GeneCards |  |
| 230 | [HBE1](https://www.genecards.org/cgi-bin/carddisp.pl?gene=HBE1&keywords=diabetic,tubulopathy) | Hemoglobin Subunit Epsilon 1 | GeneCards |  |
| 231 | [HBG1](https://www.genecards.org/cgi-bin/carddisp.pl?gene=HBG1&keywords=diabetic,tubulopathy) | Hemoglobin Subunit Gamma 1 | GeneCards |  |
| 232 | [HBG2](https://www.genecards.org/cgi-bin/carddisp.pl?gene=HBG2&keywords=diabetic,tubulopathy) | Hemoglobin Subunit Gamma 2 | GeneCards |  |
| 233 | [HBS1L](https://www.genecards.org/cgi-bin/carddisp.pl?gene=HBS1L&keywords=diabetic,tubulopathy) | HBS1 Like Translational GTPase | GeneCards |  |
| 234 | [HEY2](https://www.genecards.org/cgi-bin/carddisp.pl?gene=HEY2&keywords=diabetic,tubulopathy) | Hes Related Family BHLH Transcription Factor With YRPW Motif 2 | GeneCards |  |
| 235 | [HFE](https://www.genecards.org/cgi-bin/carddisp.pl?gene=HFE&keywords=diabetic,tubulopathy) | Homeostatic Iron Regulator | GeneCards | OMIM |
| 236 | [HJV](https://www.genecards.org/cgi-bin/carddisp.pl?gene=HJV&keywords=diabetic,tubulopathy) | Hemojuvelin BMP Co-Receptor | GeneCards |  |
| 237 | HMGA1 | High-mobility group AT-hook 1 | OMIM |  |
| 238 | HNF1A | HNF1 homeobox B | OMIM |  |
| 239 | [HNF1B](https://www.genecards.org/cgi-bin/carddisp.pl?gene=HNF1B&keywords=diabetic,tubulopathy) | HNF1 Homeobox B | GeneCards | OMIM |
| 240 | [HNF4A](https://www.genecards.org/cgi-bin/carddisp.pl?gene=HNF4A&keywords=diabetic,tubulopathy) | Hepatocyte Nuclear Factor 4 Alpha | GeneCards | OMIM |
| 241 | [HOGA1](https://www.genecards.org/cgi-bin/carddisp.pl?gene=HOGA1&keywords=diabetic,tubulopathy) | 4-Hydroxy-2-Oxoglutarate Aldolase 1 | GeneCards |  |
| 242 | [HOXA2](https://www.genecards.org/cgi-bin/carddisp.pl?gene=HOXA2&keywords=diabetic,tubulopathy) | Homeobox A2 | GeneCards |  |
| 243 | [HPD](https://www.genecards.org/cgi-bin/carddisp.pl?gene=HPD&keywords=diabetic,tubulopathy) | 4-Hydroxyphenylpyruvate Dioxygenase | GeneCards |  |
| 244 | [HSPA1L](https://www.genecards.org/cgi-bin/carddisp.pl?gene=HSPA1L&keywords=diabetic,tubulopathy) | Heat Shock Protein Family A (Hsp70) Member 1 Like | GeneCards |  |
| 245 | [HTRA2](https://www.genecards.org/cgi-bin/carddisp.pl?gene=HTRA2&keywords=diabetic,tubulopathy) | HtrA Serine Peptidase 2 | GeneCards |  |
| 246 | [HYDIN](https://www.genecards.org/cgi-bin/carddisp.pl?gene=HYDIN&keywords=diabetic,tubulopathy) | HYDIN Axonemal Central Pair Apparatus Protein | GeneCards |  |
| 247 | IAPP | Islet amyloid polypeptide (diabetes-associated peptide; amylin) | OMIM |  |
| 248 | [IARS2](https://www.genecards.org/cgi-bin/carddisp.pl?gene=IARS2&keywords=diabetic,tubulopathy) | Isoleucyl-TRNA Synthetase 2, Mitochondrial | GeneCards |  |
| 249 | [ICAM1](https://www.genecards.org/cgi-bin/carddisp.pl?gene=ICAM1&keywords=diabetic,tubulopathy) | Intercellular Adhesion Molecule 1 | GeneCards |  |
| 250 | IDDM1 | Insulin-dependent diabetes mellitus-1 | OMIM |  |
| 251 | IDDM11 | Insulin-dependent diabetes mellitus-11 | OMIM |  |
| 252 | IDDM13 | Insulin-dependent diabetes mellitus-13 | OMIM |  |
| 253 | IDDM15 | Insulin-dependent diabetes mellitus-15 | OMIM |  |
| 254 | IDDM17 | Insulin-dependent diabetes mellitus-17 | OMIM |  |
| 255 | IDDM18 | Insulin-dependent diabetes mellitus-18 | OMIM |  |
| 256 | IDDM19 | Diabetes mellitus, insulin-dependent, 19 | OMIM |  |
| 257 | IDDM21 | Diabetes mellitus, insulin-dependent, 21 | OMIM |  |
| 258 | IDDM23 | Diabetes mellitus, insulin-dependent, 23 | OMIM |  |
| 259 | IDDM24 | Diabetes mellitus, insulin-dependent, 24 | OMIM |  |
| 260 | IDDM3 | Insulin-dependent diabetes mellitus-3 | OMIM |  |
| 261 | IDDM4 | Insulin-dependent diabetes mellitus-4 | OMIM |  |
| 262 | IDDM6 | Insulin-dependent diabetes mellitus-6 | OMIM |  |
| 263 | IDDM7 | Insulin-dependent diabetes mellitus-7 | OMIM |  |
| 264 | IDDM8 | Insulin-dependent diabetes mellitus-8 | OMIM |  |
| 265 | IDDMX | Diabetes mellitus, insulin-dependent, X-linked, susceptibility to | OMIM |  |
| 266 | [IDH3A](https://www.genecards.org/cgi-bin/carddisp.pl?gene=IDH3A&keywords=diabetic,tubulopathy) | Isocitrate Dehydrogenase (NAD(+)) 3 Catalytic Subunit Alpha | GeneCards |  |
| 267 | IER3IP1 | Immediate-early response 3-interacting protein 1 | OMIM |  |
| 268 | [IGF1](https://www.genecards.org/cgi-bin/carddisp.pl?gene=IGF1&keywords=diabetic,tubulopathy) | Insulin Like Growth Factor 1 | GeneCards |  |
| 269 | IGF2BP2 | Insulin-like growth factor 2 mRNA-binding protein 2 | OMIM |  |
| 270 | [IGF2R](https://www.genecards.org/cgi-bin/carddisp.pl?gene=IGF2R&keywords=diabetic,tubulopathy) | Insulin Like Growth Factor 2 Receptor | GeneCards |  |
| 271 | [IGFBP3](https://www.genecards.org/cgi-bin/carddisp.pl?gene=IGFBP3&keywords=diabetic,tubulopathy) | Insulin Like Growth Factor Binding Protein 3 | GeneCards |  |
| 272 | [IGKC](https://www.genecards.org/cgi-bin/carddisp.pl?gene=IGKC&keywords=diabetic,tubulopathy) | Immunoglobulin Kappa Constant | GeneCards |  |
| 273 | [IL18](https://www.genecards.org/cgi-bin/carddisp.pl?gene=IL18&keywords=diabetic,tubulopathy) | Interleukin 18 | GeneCards |  |
| 274 | IL1RN | Interleukin-1 receptor antagonist | OMIM |  |
| 275 | IL2RA | Interleukin-2 receptor, alpha | OMIM |  |
| 276 | [IL3](https://www.genecards.org/cgi-bin/carddisp.pl?gene=IL3&keywords=diabetic,tubulopathy) | Interleukin 3 | GeneCards |  |
| 277 | [IL6](https://www.genecards.org/cgi-bin/carddisp.pl?gene=IL6&keywords=diabetic,tubulopathy) | Interleukin 6 | GeneCards | OMIM |
| 278 | [INCENP](https://www.genecards.org/cgi-bin/carddisp.pl?gene=INCENP&keywords=diabetic,tubulopathy) | Inner Centromere Protein | GeneCards |  |
| 279 | [INPP5B](https://www.genecards.org/cgi-bin/carddisp.pl?gene=INPP5B&keywords=diabetic,tubulopathy) | Inositol Polyphosphate-5-Phosphatase B | GeneCards |  |
| 280 | [INPP5E](https://www.genecards.org/cgi-bin/carddisp.pl?gene=INPP5E&keywords=diabetic,tubulopathy) | Inositol Polyphosphate-5-Phosphatase E | GeneCards |  |
| 281 | [INS](https://www.genecards.org/cgi-bin/carddisp.pl?gene=INS&keywords=diabetic,tubulopathy) | Insulin | GeneCards | OMIM |
| 282 | INSR | Insulin receptor | OMIM |  |
| 283 | [IREB2](https://www.genecards.org/cgi-bin/carddisp.pl?gene=IREB2&keywords=diabetic,tubulopathy) | Iron Responsive Element Binding Protein 2 | GeneCards |  |
| 284 | IRS1 | Insulin receptor substrate-1 | OMIM |  |
| 285 | IRS2 | Insulin receptor substrate 2 | OMIM |  |
| 286 | [ITGB1](https://www.genecards.org/cgi-bin/carddisp.pl?gene=ITGB1&keywords=diabetic,tubulopathy) | Integrin Subunit Beta 1 | GeneCards |  |
| 287 | ITPR3 | Inositol 1,4,5-triphosphate receptor, type 3 | OMIM |  |
| 288 | [IVD](https://www.genecards.org/cgi-bin/carddisp.pl?gene=IVD&keywords=diabetic,tubulopathy) | Isovaleryl-CoA Dehydrogenase | GeneCards |  |
| 289 | [JAK2](https://www.genecards.org/cgi-bin/carddisp.pl?gene=JAK2&keywords=diabetic,tubulopathy) | Janus Kinase 2 | GeneCards |  |
| 290 | [KCNA1](https://www.genecards.org/cgi-bin/carddisp.pl?gene=KCNA1&keywords=diabetic,tubulopathy) | Potassium Voltage-Gated Channel Subfamily A Member 1 | GeneCards |  |
| 291 | [KCNJ1](https://www.genecards.org/cgi-bin/carddisp.pl?gene=KCNJ1&keywords=diabetic,tubulopathy) | Potassium Inwardly Rectifying Channel Subfamily J Member 1 | GeneCards |  |
| 292 | [KCNJ10](https://www.genecards.org/cgi-bin/carddisp.pl?gene=KCNJ10&keywords=diabetic,tubulopathy) | Potassium Inwardly Rectifying Channel Subfamily J Member 10 | GeneCards |  |
| 293 | [KCNJ11](https://www.genecards.org/cgi-bin/carddisp.pl?gene=KCNJ11&keywords=diabetic,tubulopathy) | Potassium Inwardly Rectifying Channel Subfamily J Member 11 | GeneCards | OMIM |
| 294 | [KCNJ12](https://www.genecards.org/cgi-bin/carddisp.pl?gene=KCNJ12&keywords=diabetic,tubulopathy) | Potassium Inwardly Rectifying Channel Subfamily J Member 12 | GeneCards |  |
| 295 | [KCNJ13](https://www.genecards.org/cgi-bin/carddisp.pl?gene=KCNJ13&keywords=diabetic,tubulopathy) | Potassium Inwardly Rectifying Channel Subfamily J Member 13 | GeneCards |  |
| 296 | [KCNJ15](https://www.genecards.org/cgi-bin/carddisp.pl?gene=KCNJ15&keywords=diabetic,tubulopathy) | Potassium Inwardly Rectifying Channel Subfamily J Member 15 | GeneCards |  |
| 297 | [KCNJ16](https://www.genecards.org/cgi-bin/carddisp.pl?gene=KCNJ16&keywords=diabetic,tubulopathy) | Potassium Inwardly Rectifying Channel Subfamily J Member 16 | GeneCards |  |
| 298 | [KCNJ18](https://www.genecards.org/cgi-bin/carddisp.pl?gene=KCNJ18&keywords=diabetic,tubulopathy) | Potassium Inwardly Rectifying Channel Subfamily J Member 18 | GeneCards |  |
| 299 | [KCNJ3](https://www.genecards.org/cgi-bin/carddisp.pl?gene=KCNJ3&keywords=diabetic,tubulopathy) | Potassium Inwardly Rectifying Channel Subfamily J Member 3 | GeneCards |  |
| 300 | [KCNJ4](https://www.genecards.org/cgi-bin/carddisp.pl?gene=KCNJ4&keywords=diabetic,tubulopathy) | Potassium Inwardly Rectifying Channel Subfamily J Member 4 | GeneCards |  |
| 301 | [KCNJ9](https://www.genecards.org/cgi-bin/carddisp.pl?gene=KCNJ9&keywords=diabetic,tubulopathy) | Potassium Inwardly Rectifying Channel Subfamily J Member 9 | GeneCards |  |
| 302 | [KCNMA1](https://www.genecards.org/cgi-bin/carddisp.pl?gene=KCNMA1&keywords=diabetic,tubulopathy) | Potassium Calcium-Activated Channel Subfamily M Alpha 1 | GeneCards |  |
| 303 | [KCNQ1](https://www.genecards.org/cgi-bin/carddisp.pl?gene=KCNQ1&keywords=diabetic,tubulopathy) | Potassium Voltage-Gated Channel Subfamily Q Member 1 | GeneCards |  |
| 304 | [KCNV2](https://www.genecards.org/cgi-bin/carddisp.pl?gene=KCNV2&keywords=diabetic,tubulopathy) | Potassium Voltage-Gated Channel Modifier Subfamily V Member 2 | GeneCards |  |
| 305 | [KCTD1](https://www.genecards.org/cgi-bin/carddisp.pl?gene=KCTD1&keywords=diabetic,tubulopathy) | Potassium Channel Tetramerization Domain Containing 1 | GeneCards |  |
| 306 | [KHK](https://www.genecards.org/cgi-bin/carddisp.pl?gene=KHK&keywords=diabetic,tubulopathy) | Ketohexokinase | GeneCards |  |
| 307 | [KL](https://www.genecards.org/cgi-bin/carddisp.pl?gene=KL&keywords=diabetic,tubulopathy) | Klotho | GeneCards |  |
| 308 | [KLF1](https://www.genecards.org/cgi-bin/carddisp.pl?gene=KLF1&keywords=diabetic,tubulopathy) | Kruppel Like Factor 1 | GeneCards |  |
| 309 | [KLF11](https://www.genecards.org/cgi-bin/carddisp.pl?gene=KLF11&keywords=diabetic,tubulopathy) | Kruppel Like Factor 11 | GeneCards | OMIM |
| 310 | [KLHL3](https://www.genecards.org/cgi-bin/carddisp.pl?gene=KLHL3&keywords=diabetic,tubulopathy) | Kelch Like Family Member 3 | GeneCards |  |
| 311 | [KLK4](https://www.genecards.org/cgi-bin/carddisp.pl?gene=KLK4&keywords=diabetic,tubulopathy) | Kallikrein Related Peptidase 4 | GeneCards |  |
| 312 | [LAP3](https://www.genecards.org/cgi-bin/carddisp.pl?gene=LAP3&keywords=diabetic,tubulopathy) | Leucine Aminopeptidase 3 | GeneCards |  |
| 313 | [LARS2](https://www.genecards.org/cgi-bin/carddisp.pl?gene=LARS2&keywords=diabetic,tubulopathy) | Leucyl-TRNA Synthetase 2, Mitochondrial | GeneCards |  |
| 314 | [LCN1](https://www.genecards.org/cgi-bin/carddisp.pl?gene=LCN1&keywords=diabetic,tubulopathy) | Lipocalin 1 | GeneCards |  |
| 315 | [LCN2](https://www.genecards.org/cgi-bin/carddisp.pl?gene=LCN2&keywords=diabetic,tubulopathy) | Lipocalin 2 | GeneCards |  |
| 316 | [LIPA](https://www.genecards.org/cgi-bin/carddisp.pl?gene=LIPA&keywords=diabetic,tubulopathy) | Lipase A, Lysosomal Acid Type | GeneCards |  |
| 317 | LIPC | Lipase C, hepatic | OMIM |  |
| 318 | [LMBRD1](https://www.genecards.org/cgi-bin/carddisp.pl?gene=LMBRD1&keywords=diabetic,tubulopathy) | LMBR1 Domain Containing 1 | GeneCards |  |
| 319 | [LOC106099062](https://www.genecards.org/cgi-bin/carddisp.pl?gene=LOC106099062&keywords=diabetic,tubulopathy) | HBB Recombination Region | GeneCards |  |
| 320 | [LOC107133510](https://www.genecards.org/cgi-bin/carddisp.pl?gene=LOC107133510&keywords=diabetic,tubulopathy) | Origin Of Replication At HBB | GeneCards |  |
| 321 | [LOC109951029](https://www.genecards.org/cgi-bin/carddisp.pl?gene=LOC109951029&keywords=diabetic,tubulopathy) | Delta-Globin 5' Regulatory Region | GeneCards |  |
| 322 | [LOC110006319](https://www.genecards.org/cgi-bin/carddisp.pl?gene=LOC110006319&keywords=diabetic,tubulopathy) | Beta-Globin Gene 3' Regulatory Region | GeneCards |  |
| 323 | [LOC112081413](https://www.genecards.org/cgi-bin/carddisp.pl?gene=LOC112081413&keywords=diabetic,tubulopathy) | Sharpr-MPRA Regulatory Region 5992 | GeneCards |  |
| 324 | [LOC112552175](https://www.genecards.org/cgi-bin/carddisp.pl?gene=LOC112552175&keywords=diabetic,tubulopathy) | Sharpr-MPRA Regulatory Region 9916 | GeneCards |  |
| 325 | [LRBA](https://www.genecards.org/cgi-bin/carddisp.pl?gene=LRBA&keywords=diabetic,tubulopathy) | LPS Responsive Beige-Like Anchor Protein | GeneCards |  |
| 326 | [LRP2](https://www.genecards.org/cgi-bin/carddisp.pl?gene=LRP2&keywords=diabetic,tubulopathy) | LDL Receptor Related Protein 2 | GeneCards |  |
| 327 | [LRP8](https://www.genecards.org/cgi-bin/carddisp.pl?gene=LRP8&keywords=diabetic,tubulopathy) | LDL Receptor Related Protein 8 | GeneCards |  |
| 328 | [LRRK2](https://www.genecards.org/cgi-bin/carddisp.pl?gene=LRRK2&keywords=diabetic,tubulopathy) | Leucine Rich Repeat Kinase 2 | GeneCards |  |
| 329 | [MACO1](https://www.genecards.org/cgi-bin/carddisp.pl?gene=MACO1&keywords=diabetic,tubulopathy) | Macoilin 1 | GeneCards |  |
| 330 | [MAD2L2](https://www.genecards.org/cgi-bin/carddisp.pl?gene=MAD2L2&keywords=diabetic,tubulopathy) | Mitotic Arrest Deficient 2 Like 2 | GeneCards |  |
| 331 | MAFA | MAF bZIP transcription factor A | OMIM |  |
| 332 | [MAGED2](https://www.genecards.org/cgi-bin/carddisp.pl?gene=MAGED2&keywords=diabetic,tubulopathy) | MAGE Family Member D2 | GeneCards |  |
| 333 | [MAN1B1](https://www.genecards.org/cgi-bin/carddisp.pl?gene=MAN1B1&keywords=diabetic,tubulopathy) | Mannosidase Alpha Class 1B Member 1 | GeneCards |  |
| 334 | MAPK8IP1 | Mitogen-activated protein kinase 8-interacting protein 1 | OMIM |  |
| 335 | [MAPT](https://www.genecards.org/cgi-bin/carddisp.pl?gene=MAPT&keywords=diabetic,tubulopathy) | Microtubule Associated Protein Tau | GeneCards |  |
| 336 | [MAVS](https://www.genecards.org/cgi-bin/carddisp.pl?gene=MAVS&keywords=diabetic,tubulopathy) | Mitochondrial Antiviral Signaling Protein | GeneCards |  |
| 337 | [MED13L](https://www.genecards.org/cgi-bin/carddisp.pl?gene=MED13L&keywords=diabetic,tubulopathy) | Mediator Complex Subunit 13L | GeneCards |  |
| 338 | [MGME1](https://www.genecards.org/cgi-bin/carddisp.pl?gene=MGME1&keywords=diabetic,tubulopathy) | Mitochondrial Genome Maintenance Exonuclease 1 | GeneCards |  |
| 339 | [MIA2](https://www.genecards.org/cgi-bin/carddisp.pl?gene=MIA2&keywords=diabetic,tubulopathy) | MIA SH3 Domain ER Export Factor 2 | GeneCards |  |
| 340 | MIA3 | MIA SH3 domain ER export factor 3 | OMIM |  |
| 341 | [MIOX](https://www.genecards.org/cgi-bin/carddisp.pl?gene=MIOX&keywords=diabetic,tubulopathy) | Myo-Inositol Oxygenase | GeneCards |  |
| 342 | [MIR9-1](https://www.genecards.org/cgi-bin/carddisp.pl?gene=MIR9-1&keywords=diabetic,tubulopathy) | MicroRNA 9-1 | GeneCards |  |
| 343 | [MKX](https://www.genecards.org/cgi-bin/carddisp.pl?gene=MKX&keywords=diabetic,tubulopathy) | Mohawk Homeobox | GeneCards |  |
| 344 | [MOGS](https://www.genecards.org/cgi-bin/carddisp.pl?gene=MOGS&keywords=diabetic,tubulopathy) | Mannosyl-Oligosaccharide Glucosidase | GeneCards |  |
| 345 | [MPV17](https://www.genecards.org/cgi-bin/carddisp.pl?gene=MPV17&keywords=diabetic,tubulopathy) | Mitochondrial Inner Membrane Protein MPV17 | GeneCards |  |
| 346 | [MRM2](https://www.genecards.org/cgi-bin/carddisp.pl?gene=MRM2&keywords=diabetic,tubulopathy) | Mitochondrial RRNA Methyltransferase 2 | GeneCards |  |
| 347 | [MRPL18](https://www.genecards.org/cgi-bin/carddisp.pl?gene=MRPL18&keywords=diabetic,tubulopathy) | Mitochondrial Ribosomal Protein L18 | GeneCards |  |
| 348 | [MRPL36](https://www.genecards.org/cgi-bin/carddisp.pl?gene=MRPL36&keywords=diabetic,tubulopathy) | Mitochondrial Ribosomal Protein L36 | GeneCards |  |
| 349 | [MRPL44](https://www.genecards.org/cgi-bin/carddisp.pl?gene=MRPL44&keywords=diabetic,tubulopathy) | Mitochondrial Ribosomal Protein L44 | GeneCards |  |
| 350 | [MRPS22](https://www.genecards.org/cgi-bin/carddisp.pl?gene=MRPS22&keywords=diabetic,tubulopathy) | Mitochondrial Ribosomal Protein S22 | GeneCards |  |
| 351 | [MRRF](https://www.genecards.org/cgi-bin/carddisp.pl?gene=MRRF&keywords=diabetic,tubulopathy) | Mitochondrial Ribosome Recycling Factor | GeneCards |  |
| 352 | [MSTO1](https://www.genecards.org/cgi-bin/carddisp.pl?gene=MSTO1&keywords=diabetic,tubulopathy) | Misato Mitochondrial Distribution And Morphology Regulator 1 | GeneCards |  |
| 353 | [MT-ATP6](https://www.genecards.org/cgi-bin/carddisp.pl?gene=MT-ATP6&keywords=diabetic,tubulopathy) | Mitochondrially Encoded ATP Synthase Membrane Subunit 6 | GeneCards |  |
| 354 | [MT-ATP8](https://www.genecards.org/cgi-bin/carddisp.pl?gene=MT-ATP8&keywords=diabetic,tubulopathy) | Mitochondrially Encoded ATP Synthase Membrane Subunit 8 | GeneCards |  |
| 355 | [MT-CO1](https://www.genecards.org/cgi-bin/carddisp.pl?gene=MT-CO1&keywords=diabetic,tubulopathy) | Mitochondrially Encoded Cytochrome C Oxidase I | GeneCards |  |
| 356 | [MT-CO2](https://www.genecards.org/cgi-bin/carddisp.pl?gene=MT-CO2&keywords=diabetic,tubulopathy) | Mitochondrially Encoded Cytochrome C Oxidase II | GeneCards |  |
| 357 | [MT-CO3](https://www.genecards.org/cgi-bin/carddisp.pl?gene=MT-CO3&keywords=diabetic,tubulopathy) | Mitochondrially Encoded Cytochrome C Oxidase III | GeneCards |  |
| 358 | [MT-CYB](https://www.genecards.org/cgi-bin/carddisp.pl?gene=MT-CYB&keywords=diabetic,tubulopathy) | Mitochondrially Encoded Cytochrome B | GeneCards |  |
| 359 | [MTERF1](https://www.genecards.org/cgi-bin/carddisp.pl?gene=MTERF1&keywords=diabetic,tubulopathy) | Mitochondrial Transcription Termination Factor 1 | GeneCards |  |
| 360 | [MTFMT](https://www.genecards.org/cgi-bin/carddisp.pl?gene=MTFMT&keywords=diabetic,tubulopathy) | Mitochondrial Methionyl-TRNA Formyltransferase | GeneCards |  |
| 361 | [MTHFD2](https://www.genecards.org/cgi-bin/carddisp.pl?gene=MTHFD2&keywords=diabetic,tubulopathy) | Methylenetetrahydrofolate Dehydrogenase (NADP+ Dependent) 2, Methenyltetrahydrofolate Cyclohydrolase | GeneCards |  |
| 362 | [MTHFR](https://www.genecards.org/cgi-bin/carddisp.pl?gene=MTHFR&keywords=diabetic,tubulopathy) | Methylenetetrahydrofolate Reductase | GeneCards |  |
| 363 | [MT-ND1](https://www.genecards.org/cgi-bin/carddisp.pl?gene=MT-ND1&keywords=diabetic,tubulopathy) | Mitochondrially Encoded NADH:Ubiquinone Oxidoreductase Core Subunit 1 | GeneCards |  |
| 364 | [MT-ND2](https://www.genecards.org/cgi-bin/carddisp.pl?gene=MT-ND2&keywords=diabetic,tubulopathy) | Mitochondrially Encoded NADH:Ubiquinone Oxidoreductase Core Subunit 2 | GeneCards |  |
| 365 | [MT-ND3](https://www.genecards.org/cgi-bin/carddisp.pl?gene=MT-ND3&keywords=diabetic,tubulopathy) | Mitochondrially Encoded NADH:Ubiquinone Oxidoreductase Core Subunit 3 | GeneCards |  |
| 366 | [MT-ND4](https://www.genecards.org/cgi-bin/carddisp.pl?gene=MT-ND4&keywords=diabetic,tubulopathy) | Mitochondrially Encoded NADH:Ubiquinone Oxidoreductase Core Subunit 4 | GeneCards |  |
| 367 | [MT-ND4L](https://www.genecards.org/cgi-bin/carddisp.pl?gene=MT-ND4L&keywords=diabetic,tubulopathy) | Mitochondrially Encoded NADH:Ubiquinone Oxidoreductase Core Subunit 4L | GeneCards |  |
| 368 | [MT-ND5](https://www.genecards.org/cgi-bin/carddisp.pl?gene=MT-ND5&keywords=diabetic,tubulopathy) | Mitochondrially Encoded NADH:Ubiquinone Oxidoreductase Core Subunit 5 | GeneCards |  |
| 369 | [MT-ND6](https://www.genecards.org/cgi-bin/carddisp.pl?gene=MT-ND6&keywords=diabetic,tubulopathy) | Mitochondrially Encoded NADH:Ubiquinone Oxidoreductase Core Subunit 6 | GeneCards |  |
| 370 | MTNR1B | Melatonin receptor 1B | OMIM |  |
| 371 | [MTO1](https://www.genecards.org/cgi-bin/carddisp.pl?gene=MTO1&keywords=diabetic,tubulopathy) | Mitochondrial TRNA Translation Optimization 1 | GeneCards |  |
| 372 | [MT-RNR1](https://www.genecards.org/cgi-bin/carddisp.pl?gene=MT-RNR1&keywords=diabetic,tubulopathy) | Mitochondrially Encoded 12S RRNA | GeneCards |  |
| 373 | [MT-RNR2](https://www.genecards.org/cgi-bin/carddisp.pl?gene=MT-RNR2&keywords=diabetic,tubulopathy) | Mitochondrially Encoded 16S RRNA | GeneCards |  |
| 374 | [MT-TA](https://www.genecards.org/cgi-bin/carddisp.pl?gene=MT-TA&keywords=diabetic,tubulopathy) | Mitochondrially Encoded TRNA-Ala (GCN) | GeneCards |  |
| 375 | [MT-TD](https://www.genecards.org/cgi-bin/carddisp.pl?gene=MT-TD&keywords=diabetic,tubulopathy) | Mitochondrially Encoded TRNA-Asp (GAU/C) | GeneCards |  |
| 376 | [MT-TE](https://www.genecards.org/cgi-bin/carddisp.pl?gene=MT-TE&keywords=diabetic,tubulopathy) | Mitochondrially Encoded TRNA-Glu (GAA/G) | GeneCards |  |
| 377 | [MT-TF](https://www.genecards.org/cgi-bin/carddisp.pl?gene=MT-TF&keywords=diabetic,tubulopathy) | Mitochondrially Encoded TRNA-Phe (UUU/C) | GeneCards |  |
| 378 | [MT-TG](https://www.genecards.org/cgi-bin/carddisp.pl?gene=MT-TG&keywords=diabetic,tubulopathy) | Mitochondrially Encoded TRNA-Gly (GGN) | GeneCards |  |
| 379 | [MT-TH](https://www.genecards.org/cgi-bin/carddisp.pl?gene=MT-TH&keywords=diabetic,tubulopathy) | Mitochondrially Encoded TRNA-His (CAU/C) | GeneCards |  |
| 380 | [MT-TI](https://www.genecards.org/cgi-bin/carddisp.pl?gene=MT-TI&keywords=diabetic,tubulopathy) | Mitochondrially Encoded TRNA-Ile (AUU/C) | GeneCards |  |
| 381 | [MT-TK](https://www.genecards.org/cgi-bin/carddisp.pl?gene=MT-TK&keywords=diabetic,tubulopathy) | Mitochondrially Encoded TRNA-Lys (AAA/G) | GeneCards |  |
| 382 | [MT-TL1](https://www.genecards.org/cgi-bin/carddisp.pl?gene=MT-TL1&keywords=diabetic,tubulopathy) | Mitochondrially Encoded TRNA-Leu (UUA/G) 1 | GeneCards |  |
| 383 | [MT-TL2](https://www.genecards.org/cgi-bin/carddisp.pl?gene=MT-TL2&keywords=diabetic,tubulopathy) | Mitochondrially Encoded TRNA-Leu (CUN) 2 | GeneCards |  |
| 384 | [MT-TM](https://www.genecards.org/cgi-bin/carddisp.pl?gene=MT-TM&keywords=diabetic,tubulopathy) | Mitochondrially Encoded TRNA-Met (AUA/G) | GeneCards |  |
| 385 | [MT-TN](https://www.genecards.org/cgi-bin/carddisp.pl?gene=MT-TN&keywords=diabetic,tubulopathy) | Mitochondrially Encoded TRNA-Asn (AAU/C) | GeneCards |  |
| 386 | [MT-TP](https://www.genecards.org/cgi-bin/carddisp.pl?gene=MT-TP&keywords=diabetic,tubulopathy) | Mitochondrially Encoded TRNA-Pro (CCN) | GeneCards |  |
| 387 | [MT-TQ](https://www.genecards.org/cgi-bin/carddisp.pl?gene=MT-TQ&keywords=diabetic,tubulopathy) | Mitochondrially Encoded TRNA-Gln (CAA/G) | GeneCards |  |
| 388 | [MT-TR](https://www.genecards.org/cgi-bin/carddisp.pl?gene=MT-TR&keywords=diabetic,tubulopathy) | Mitochondrially Encoded TRNA-Arg (CGN) | GeneCards |  |
| 389 | [MT-TS1](https://www.genecards.org/cgi-bin/carddisp.pl?gene=MT-TS1&keywords=diabetic,tubulopathy) | Mitochondrially Encoded TRNA-Ser (UCN) 1 | GeneCards |  |
| 390 | [MT-TS2](https://www.genecards.org/cgi-bin/carddisp.pl?gene=MT-TS2&keywords=diabetic,tubulopathy) | Mitochondrially Encoded TRNA-Ser (AGU/C) 2 | GeneCards |  |
| 391 | [MT-TT](https://www.genecards.org/cgi-bin/carddisp.pl?gene=MT-TT&keywords=diabetic,tubulopathy) | Mitochondrially Encoded TRNA-Thr (ACN) | GeneCards |  |
| 392 | [MT-TV](https://www.genecards.org/cgi-bin/carddisp.pl?gene=MT-TV&keywords=diabetic,tubulopathy) | Mitochondrially Encoded TRNA-Val (GUN) | GeneCards |  |
| 393 | [MT-TW](https://www.genecards.org/cgi-bin/carddisp.pl?gene=MT-TW&keywords=diabetic,tubulopathy) | Mitochondrially Encoded TRNA-Trp (UGA/G) | GeneCards |  |
| 394 | [MYB](https://www.genecards.org/cgi-bin/carddisp.pl?gene=MYB&keywords=diabetic,tubulopathy) | MYB Proto-Oncogene, Transcription Factor | GeneCards |  |
| 395 | [MYO5B](https://www.genecards.org/cgi-bin/carddisp.pl?gene=MYO5B&keywords=diabetic,tubulopathy) | Myosin VB | GeneCards |  |
| 396 | [NAGLU](https://www.genecards.org/cgi-bin/carddisp.pl?gene=NAGLU&keywords=diabetic,tubulopathy) | N-Acetyl-Alpha-Glucosaminidase | GeneCards |  |
| 397 | [NCOR1](https://www.genecards.org/cgi-bin/carddisp.pl?gene=NCOR1&keywords=diabetic,tubulopathy) | Nuclear Receptor Corepressor 1 | GeneCards |  |
| 398 | [NDUFA1](https://www.genecards.org/cgi-bin/carddisp.pl?gene=NDUFA1&keywords=diabetic,tubulopathy) | NADH:Ubiquinone Oxidoreductase Subunit A1 | GeneCards |  |
| 399 | [NDUFA10](https://www.genecards.org/cgi-bin/carddisp.pl?gene=NDUFA10&keywords=diabetic,tubulopathy) | NADH:Ubiquinone Oxidoreductase Subunit A10 | GeneCards |  |
| 400 | [NDUFA11](https://www.genecards.org/cgi-bin/carddisp.pl?gene=NDUFA11&keywords=diabetic,tubulopathy) | NADH:Ubiquinone Oxidoreductase Subunit A11 | GeneCards |  |
| 401 | [NDUFA12](https://www.genecards.org/cgi-bin/carddisp.pl?gene=NDUFA12&keywords=diabetic,tubulopathy) | NADH:Ubiquinone Oxidoreductase Subunit A12 | GeneCards |  |
| 402 | [NDUFA13](https://www.genecards.org/cgi-bin/carddisp.pl?gene=NDUFA13&keywords=diabetic,tubulopathy) | NADH:Ubiquinone Oxidoreductase Subunit A13 | GeneCards |  |
| 403 | [NDUFA2](https://www.genecards.org/cgi-bin/carddisp.pl?gene=NDUFA2&keywords=diabetic,tubulopathy) | NADH:Ubiquinone Oxidoreductase Subunit A2 | GeneCards |  |
| 404 | [NDUFA6](https://www.genecards.org/cgi-bin/carddisp.pl?gene=NDUFA6&keywords=diabetic,tubulopathy) | NADH:Ubiquinone Oxidoreductase Subunit A6 | GeneCards |  |
| 405 | [NDUFA8](https://www.genecards.org/cgi-bin/carddisp.pl?gene=NDUFA8&keywords=diabetic,tubulopathy) | NADH:Ubiquinone Oxidoreductase Subunit A8 | GeneCards |  |
| 406 | [NDUFA9](https://www.genecards.org/cgi-bin/carddisp.pl?gene=NDUFA9&keywords=diabetic,tubulopathy) | NADH:Ubiquinone Oxidoreductase Subunit A9 | GeneCards |  |
| 407 | [NDUFAF1](https://www.genecards.org/cgi-bin/carddisp.pl?gene=NDUFAF1&keywords=diabetic,tubulopathy) | NADH:Ubiquinone Oxidoreductase Complex Assembly Factor 1 | GeneCards |  |
| 408 | [NDUFAF2](https://www.genecards.org/cgi-bin/carddisp.pl?gene=NDUFAF2&keywords=diabetic,tubulopathy) | NADH:Ubiquinone Oxidoreductase Complex Assembly Factor 2 | GeneCards |  |
| 409 | [NDUFAF3](https://www.genecards.org/cgi-bin/carddisp.pl?gene=NDUFAF3&keywords=diabetic,tubulopathy) | NADH:Ubiquinone Oxidoreductase Complex Assembly Factor 3 | GeneCards |  |
| 410 | [NDUFAF4](https://www.genecards.org/cgi-bin/carddisp.pl?gene=NDUFAF4&keywords=diabetic,tubulopathy) | NADH:Ubiquinone Oxidoreductase Complex Assembly Factor 4 | GeneCards |  |
| 411 | [NDUFAF5](https://www.genecards.org/cgi-bin/carddisp.pl?gene=NDUFAF5&keywords=diabetic,tubulopathy) | NADH:Ubiquinone Oxidoreductase Complex Assembly Factor 5 | GeneCards |  |
| 412 | [NDUFAF6](https://www.genecards.org/cgi-bin/carddisp.pl?gene=NDUFAF6&keywords=diabetic,tubulopathy) | NADH:Ubiquinone Oxidoreductase Complex Assembly Factor 6 | GeneCards |  |
| 413 | [NDUFAF8](https://www.genecards.org/cgi-bin/carddisp.pl?gene=NDUFAF8&keywords=diabetic,tubulopathy) | NADH:Ubiquinone Oxidoreductase Complex Assembly Factor 8 | GeneCards |  |
| 414 | [NDUFB10](https://www.genecards.org/cgi-bin/carddisp.pl?gene=NDUFB10&keywords=diabetic,tubulopathy) | NADH:Ubiquinone Oxidoreductase Subunit B10 | GeneCards |  |
| 415 | [NDUFB11](https://www.genecards.org/cgi-bin/carddisp.pl?gene=NDUFB11&keywords=diabetic,tubulopathy) | NADH:Ubiquinone Oxidoreductase Subunit B11 | GeneCards |  |
| 416 | [NDUFB3](https://www.genecards.org/cgi-bin/carddisp.pl?gene=NDUFB3&keywords=diabetic,tubulopathy) | NADH:Ubiquinone Oxidoreductase Subunit B3 | GeneCards |  |
| 417 | [NDUFB6](https://www.genecards.org/cgi-bin/carddisp.pl?gene=NDUFB6&keywords=diabetic,tubulopathy) | NADH:Ubiquinone Oxidoreductase Subunit B6 | GeneCards |  |
| 418 | [NDUFB8](https://www.genecards.org/cgi-bin/carddisp.pl?gene=NDUFB8&keywords=diabetic,tubulopathy) | NADH:Ubiquinone Oxidoreductase Subunit B8 | GeneCards |  |
| 419 | [NDUFB9](https://www.genecards.org/cgi-bin/carddisp.pl?gene=NDUFB9&keywords=diabetic,tubulopathy) | NADH:Ubiquinone Oxidoreductase Subunit B9 | GeneCards |  |
| 420 | [NDUFC2](https://www.genecards.org/cgi-bin/carddisp.pl?gene=NDUFC2&keywords=diabetic,tubulopathy) | NADH:Ubiquinone Oxidoreductase Subunit C2 | GeneCards |  |
| 421 | [NDUFS1](https://www.genecards.org/cgi-bin/carddisp.pl?gene=NDUFS1&keywords=diabetic,tubulopathy) | NADH:Ubiquinone Oxidoreductase Core Subunit S1 | GeneCards |  |
| 422 | [NDUFS2](https://www.genecards.org/cgi-bin/carddisp.pl?gene=NDUFS2&keywords=diabetic,tubulopathy) | NADH:Ubiquinone Oxidoreductase Core Subunit S2 | GeneCards |  |
| 423 | [NDUFS3](https://www.genecards.org/cgi-bin/carddisp.pl?gene=NDUFS3&keywords=diabetic,tubulopathy) | NADH:Ubiquinone Oxidoreductase Core Subunit S3 | GeneCards |  |
| 424 | [NDUFS4](https://www.genecards.org/cgi-bin/carddisp.pl?gene=NDUFS4&keywords=diabetic,tubulopathy) | NADH:Ubiquinone Oxidoreductase Subunit S4 | GeneCards |  |
| 425 | [NDUFS5](https://www.genecards.org/cgi-bin/carddisp.pl?gene=NDUFS5&keywords=diabetic,tubulopathy) | NADH:Ubiquinone Oxidoreductase Subunit S5 | GeneCards |  |
| 426 | [NDUFS6](https://www.genecards.org/cgi-bin/carddisp.pl?gene=NDUFS6&keywords=diabetic,tubulopathy) | NADH:Ubiquinone Oxidoreductase Subunit S6 | GeneCards |  |
| 427 | [NDUFS7](https://www.genecards.org/cgi-bin/carddisp.pl?gene=NDUFS7&keywords=diabetic,tubulopathy) | NADH:Ubiquinone Oxidoreductase Core Subunit S7 | GeneCards |  |
| 428 | [NDUFS8](https://www.genecards.org/cgi-bin/carddisp.pl?gene=NDUFS8&keywords=diabetic,tubulopathy) | NADH:Ubiquinone Oxidoreductase Core Subunit S8 | GeneCards |  |
| 429 | [NDUFV1](https://www.genecards.org/cgi-bin/carddisp.pl?gene=NDUFV1&keywords=diabetic,tubulopathy) | NADH:Ubiquinone Oxidoreductase Core Subunit V1 | GeneCards |  |
| 430 | [NDUFV2](https://www.genecards.org/cgi-bin/carddisp.pl?gene=NDUFV2&keywords=diabetic,tubulopathy) | NADH:Ubiquinone Oxidoreductase Core Subunit V2 | GeneCards |  |
| 431 | [NDUFV2-AS1](https://www.genecards.org/cgi-bin/carddisp.pl?gene=NDUFV2-AS1&keywords=diabetic,tubulopathy) | NDUFV2 Antisense RNA 1 | GeneCards |  |
| 432 | NEUROD1 | Neurogenic differentiation 1 | OMIM |  |
| 433 | [NEXN](https://www.genecards.org/cgi-bin/carddisp.pl?gene=NEXN&keywords=diabetic,tubulopathy) | Nexilin F-Actin Binding Protein | GeneCards |  |
| 434 | [NFIB](https://www.genecards.org/cgi-bin/carddisp.pl?gene=NFIB&keywords=diabetic,tubulopathy) | Nuclear Factor I B | GeneCards |  |
| 435 | NIDDM2 | Diabetes mellitus, noninsulin-dependent, 2 | OMIM |  |
| 436 | NIDDM4 | Diabetes mellitus, noninsulin-dependent, 4 | OMIM |  |
| 437 | [NKX2-5](https://www.genecards.org/cgi-bin/carddisp.pl?gene=NKX2-5&keywords=diabetic,tubulopathy) | NK2 Homeobox 5 | GeneCards |  |
| 438 | [NLRX1](https://www.genecards.org/cgi-bin/carddisp.pl?gene=NLRX1&keywords=diabetic,tubulopathy) | NLR Family Member X1 | GeneCards |  |
| 439 | [NR3C2](https://www.genecards.org/cgi-bin/carddisp.pl?gene=NR3C2&keywords=diabetic,tubulopathy) | Nuclear Receptor Subfamily 3 Group C Member 2 | GeneCards |  |
| 440 | [NUBPL](https://www.genecards.org/cgi-bin/carddisp.pl?gene=NUBPL&keywords=diabetic,tubulopathy) | Nucleotide Binding Protein Like | GeneCards |  |
| 441 | [OCRL](https://www.genecards.org/cgi-bin/carddisp.pl?gene=OCRL&keywords=diabetic,tubulopathy) | OCRL Inositol Polyphosphate-5-Phosphatase | GeneCards |  |
| 442 | [OR4L1](https://www.genecards.org/cgi-bin/carddisp.pl?gene=OR4L1&keywords=diabetic,tubulopathy) | Olfactory Receptor Family 4 Subfamily L Member 1 | GeneCards |  |
| 443 | [OSGEP](https://www.genecards.org/cgi-bin/carddisp.pl?gene=OSGEP&keywords=diabetic,tubulopathy) | O-Sialoglycoprotein Endopeptidase | GeneCards |  |
| 444 | [OSGEPL1](https://www.genecards.org/cgi-bin/carddisp.pl?gene=OSGEPL1&keywords=diabetic,tubulopathy) | O-Sialoglycoprotein Endopeptidase Like 1 | GeneCards |  |
| 445 | [OXSR1](https://www.genecards.org/cgi-bin/carddisp.pl?gene=OXSR1&keywords=diabetic,tubulopathy) | Oxidative Stress Responsive Kinase 1 | GeneCards |  |
| 446 | [PARK7](https://www.genecards.org/cgi-bin/carddisp.pl?gene=PARK7&keywords=diabetic,tubulopathy) | Parkinsonism Associated Deglycase | GeneCards |  |
| 447 | [PAX2](https://www.genecards.org/cgi-bin/carddisp.pl?gene=PAX2&keywords=diabetic,tubulopathy) | Paired Box 2 | GeneCards |  |
| 448 | PAX4 | Paired box homeotic gene-4 | OMIM |  |
| 449 | PBCA | Pancreatic beta cell, agenesis of | OMIM |  |
| 450 | [PCBD1](https://www.genecards.org/cgi-bin/carddisp.pl?gene=PCBD1&keywords=diabetic,tubulopathy) | Pterin-4 Alpha-Carbinolamine Dehydratase 1 | GeneCards |  |
| 451 | [PDHA1](https://www.genecards.org/cgi-bin/carddisp.pl?gene=PDHA1&keywords=diabetic,tubulopathy) | Pyruvate Dehydrogenase E1 Subunit Alpha 1 | GeneCards |  |
| 452 | [PDSS1](https://www.genecards.org/cgi-bin/carddisp.pl?gene=PDSS1&keywords=diabetic,tubulopathy) | Decaprenyl Diphosphate Synthase Subunit 1 | GeneCards |  |
| 453 | [PDSS2](https://www.genecards.org/cgi-bin/carddisp.pl?gene=PDSS2&keywords=diabetic,tubulopathy) | Decaprenyl Diphosphate Synthase Subunit 2 | GeneCards |  |
| 454 | PDX1 | Pancreas/duodenum homeobox protein 1 | OMIM |  |
| 455 | [PET100](https://www.genecards.org/cgi-bin/carddisp.pl?gene=PET100&keywords=diabetic,tubulopathy) | PET100 Cytochrome C Oxidase Chaperone | GeneCards |  |
| 456 | [PET117](https://www.genecards.org/cgi-bin/carddisp.pl?gene=PET117&keywords=diabetic,tubulopathy) | PET117 Cytochrome C Oxidase Chaperone | GeneCards |  |
| 457 | [PHETA1](https://www.genecards.org/cgi-bin/carddisp.pl?gene=PHETA1&keywords=diabetic,tubulopathy) | PH Domain Containing Endocytic Trafficking Adaptor 1 | GeneCards |  |
| 458 | [PHEX](https://www.genecards.org/cgi-bin/carddisp.pl?gene=PHEX&keywords=diabetic,tubulopathy) | Phosphate Regulating Endopeptidase Homolog X-Linked | GeneCards |  |
| 459 | [PINK1](https://www.genecards.org/cgi-bin/carddisp.pl?gene=PINK1&keywords=diabetic,tubulopathy) | PTEN Induced Kinase 1 | GeneCards |  |
| 460 | [PKD2](https://www.genecards.org/cgi-bin/carddisp.pl?gene=PKD2&keywords=diabetic,tubulopathy) | Polycystin 2, Transient Receptor Potential Cation Channel | GeneCards |  |
| 461 | [PLA2G6](https://www.genecards.org/cgi-bin/carddisp.pl?gene=PLA2G6&keywords=diabetic,tubulopathy) | Phospholipase A2 Group VI | GeneCards |  |
| 462 | [PMM2](https://www.genecards.org/cgi-bin/carddisp.pl?gene=PMM2&keywords=diabetic,tubulopathy) | Phosphomannomutase 2 | GeneCards |  |
| 463 | [PMPCA](https://www.genecards.org/cgi-bin/carddisp.pl?gene=PMPCA&keywords=diabetic,tubulopathy) | Peptidase, Mitochondrial Processing Subunit Alpha | GeneCards |  |
| 464 | [POLG](https://www.genecards.org/cgi-bin/carddisp.pl?gene=POLG&keywords=diabetic,tubulopathy) | DNA Polymerase Gamma, Catalytic Subunit | GeneCards |  |
| 465 | [PON1](https://www.genecards.org/cgi-bin/carddisp.pl?gene=PON1&keywords=diabetic,tubulopathy) | Paraoxonase 1 | GeneCards | OMIM |
| 466 | [POU6F2](https://www.genecards.org/cgi-bin/carddisp.pl?gene=POU6F2&keywords=diabetic,tubulopathy) | POU Class 6 Homeobox 2 | GeneCards |  |
| 467 | [PPARG](https://www.genecards.org/cgi-bin/carddisp.pl?gene=PPARG&keywords=diabetic,tubulopathy) | Peroxisome Proliferator Activated Receptor Gamma | GeneCards | OMIM |
| 468 | [PRIMPOL](https://www.genecards.org/cgi-bin/carddisp.pl?gene=PRIMPOL&keywords=diabetic,tubulopathy) | Primase And DNA Directed Polymerase | GeneCards |  |
| 469 | [PRKD2](https://www.genecards.org/cgi-bin/carddisp.pl?gene=PRKD2&keywords=diabetic,tubulopathy) | Protein Kinase D2 | GeneCards |  |
| 470 | [PRKN](https://www.genecards.org/cgi-bin/carddisp.pl?gene=PRKN&keywords=diabetic,tubulopathy) | Parkin RBR E3 Ubiquitin Protein Ligase | GeneCards |  |
| 471 | [PROCR](https://www.genecards.org/cgi-bin/carddisp.pl?gene=PROCR&keywords=diabetic,tubulopathy) | Protein C Receptor | GeneCards |  |
| 472 | [PRODH](https://www.genecards.org/cgi-bin/carddisp.pl?gene=PRODH&keywords=diabetic,tubulopathy) | Proline Dehydrogenase 1 | GeneCards |  |
| 473 | [PTCHD1-AS](https://www.genecards.org/cgi-bin/carddisp.pl?gene=PTCHD1-AS&keywords=diabetic,tubulopathy) | PTCHD1 Antisense RNA (Head To Head) | GeneCards |  |
| 474 | [PTGER1](https://www.genecards.org/cgi-bin/carddisp.pl?gene=PTGER1&keywords=diabetic,tubulopathy) | Prostaglandin E Receptor 1 | GeneCards |  |
| 475 | [PTGER3](https://www.genecards.org/cgi-bin/carddisp.pl?gene=PTGER3&keywords=diabetic,tubulopathy) | Prostaglandin E Receptor 3 | GeneCards |  |
| 476 | [PTGER4](https://www.genecards.org/cgi-bin/carddisp.pl?gene=PTGER4&keywords=diabetic,tubulopathy) | Prostaglandin E Receptor 4 | GeneCards |  |
| 477 | [PTGES](https://www.genecards.org/cgi-bin/carddisp.pl?gene=PTGES&keywords=diabetic,tubulopathy) | Prostaglandin E Synthase | GeneCards |  |
| 478 | [PTGS1](https://www.genecards.org/cgi-bin/carddisp.pl?gene=PTGS1&keywords=diabetic,tubulopathy) | Prostaglandin-Endoperoxide Synthase 1 | GeneCards |  |
| 479 | [PTH](https://www.genecards.org/cgi-bin/carddisp.pl?gene=PTH&keywords=diabetic,tubulopathy) | Parathyroid Hormone | GeneCards |  |
| 480 | PTPN22 | Protein tyrosine phosphatase, nonreceptor-type 22 | OMIM |  |
| 481 | [PVALB](https://www.genecards.org/cgi-bin/carddisp.pl?gene=PVALB&keywords=diabetic,tubulopathy) | Parvalbumin | GeneCards |  |
| 482 | [RAB5A](https://www.genecards.org/cgi-bin/carddisp.pl?gene=RAB5A&keywords=diabetic,tubulopathy) | RAB5A, Member RAS Oncogene Family | GeneCards |  |
| 483 | [RAB8A](https://www.genecards.org/cgi-bin/carddisp.pl?gene=RAB8A&keywords=diabetic,tubulopathy) | RAB8A, Member RAS Oncogene Family | GeneCards |  |
| 484 | [RAC1](https://www.genecards.org/cgi-bin/carddisp.pl?gene=RAC1&keywords=diabetic,tubulopathy) | Rac Family Small GTPase 1 | GeneCards |  |
| 485 | [RARS2](https://www.genecards.org/cgi-bin/carddisp.pl?gene=RARS2&keywords=diabetic,tubulopathy) | Arginyl-TRNA Synthetase 2, Mitochondrial | GeneCards |  |
| 486 | [RBM48](https://www.genecards.org/cgi-bin/carddisp.pl?gene=RBM48&keywords=diabetic,tubulopathy) | RNA Binding Motif Protein 48 | GeneCards |  |
| 487 | [RBP4](https://www.genecards.org/cgi-bin/carddisp.pl?gene=RBP4&keywords=diabetic,tubulopathy) | Retinol Binding Protein 4 | GeneCards |  |
| 488 | [RCOR1](https://www.genecards.org/cgi-bin/carddisp.pl?gene=RCOR1&keywords=diabetic,tubulopathy) | REST Corepressor 1 | GeneCards |  |
| 489 | [REN](https://www.genecards.org/cgi-bin/carddisp.pl?gene=REN&keywords=diabetic,tubulopathy) | Renin | GeneCards |  |
| 490 | RETN | Resistin | OMIM |  |
| 491 | [RFK](https://www.genecards.org/cgi-bin/carddisp.pl?gene=RFK&keywords=diabetic,tubulopathy) | Riboflavin Kinase | GeneCards |  |
| 492 | [RFWD3](https://www.genecards.org/cgi-bin/carddisp.pl?gene=RFWD3&keywords=diabetic,tubulopathy) | Ring Finger And WD Repeat Domain 3 | GeneCards |  |
| 493 | [RHD](https://www.genecards.org/cgi-bin/carddisp.pl?gene=RHD&keywords=diabetic,tubulopathy) | Rh Blood Group D Antigen | GeneCards |  |
| 494 | [RNF220](https://www.genecards.org/cgi-bin/carddisp.pl?gene=RNF220&keywords=diabetic,tubulopathy) | Ring Finger Protein 220 | GeneCards |  |
| 495 | [RPS27A](https://www.genecards.org/cgi-bin/carddisp.pl?gene=RPS27A&keywords=diabetic,tubulopathy) | Ribosomal Protein S27a | GeneCards |  |
| 496 | RRAD | Ras-related associated with diabetes | OMIM |  |
| 497 | [RRM2B](https://www.genecards.org/cgi-bin/carddisp.pl?gene=RRM2B&keywords=diabetic,tubulopathy) | Ribonucleotide Reductase Regulatory TP53 Inducible Subunit M2B | GeneCards | OMIM |
| 498 | [RTN4IP1](https://www.genecards.org/cgi-bin/carddisp.pl?gene=RTN4IP1&keywords=diabetic,tubulopathy) | Reticulon 4 Interacting Protein 1 | GeneCards |  |
| 499 | [SARS1](https://www.genecards.org/cgi-bin/carddisp.pl?gene=SARS1&keywords=diabetic,tubulopathy) | Seryl-TRNA Synthetase 1 | GeneCards |  |
| 500 | [SARS2](https://www.genecards.org/cgi-bin/carddisp.pl?gene=SARS2&keywords=diabetic,tubulopathy) | Seryl-TRNA Synthetase 2, Mitochondrial | GeneCards |  |
| 501 | [SCGN](https://www.genecards.org/cgi-bin/carddisp.pl?gene=SCGN&keywords=diabetic,tubulopathy) | Secretagogin, EF-Hand Calcium Binding Protein | GeneCards |  |
| 502 | [SCNN1A](https://www.genecards.org/cgi-bin/carddisp.pl?gene=SCNN1A&keywords=diabetic,tubulopathy) | Sodium Channel Epithelial 1 Subunit Alpha | GeneCards |  |
| 503 | [SCNN1B](https://www.genecards.org/cgi-bin/carddisp.pl?gene=SCNN1B&keywords=diabetic,tubulopathy) | Sodium Channel Epithelial 1 Subunit Beta | GeneCards |  |
| 504 | [SCNN1G](https://www.genecards.org/cgi-bin/carddisp.pl?gene=SCNN1G&keywords=diabetic,tubulopathy) | Sodium Channel Epithelial 1 Subunit Gamma | GeneCards |  |
| 505 | [SCO1](https://www.genecards.org/cgi-bin/carddisp.pl?gene=SCO1&keywords=diabetic,tubulopathy) | Synthesis Of Cytochrome C Oxidase 1 | GeneCards |  |
| 506 | [SCO2](https://www.genecards.org/cgi-bin/carddisp.pl?gene=SCO2&keywords=diabetic,tubulopathy) | Synthesis Of Cytochrome C Oxidase 2 | GeneCards |  |
| 507 | [SDHA](https://www.genecards.org/cgi-bin/carddisp.pl?gene=SDHA&keywords=diabetic,tubulopathy) | Succinate Dehydrogenase Complex Flavoprotein Subunit A | GeneCards |  |
| 508 | [SDHC](https://www.genecards.org/cgi-bin/carddisp.pl?gene=SDHC&keywords=diabetic,tubulopathy) | Succinate Dehydrogenase Complex Subunit C | GeneCards |  |
| 509 | [SDHD](https://www.genecards.org/cgi-bin/carddisp.pl?gene=SDHD&keywords=diabetic,tubulopathy) | Succinate Dehydrogenase Complex Subunit D | GeneCards |  |
| 510 | [SERPINC1](https://www.genecards.org/cgi-bin/carddisp.pl?gene=SERPINC1&keywords=diabetic,tubulopathy) | Serpin Family C Member 1 | GeneCards |  |
| 511 | [SHH](https://www.genecards.org/cgi-bin/carddisp.pl?gene=SHH&keywords=diabetic,tubulopathy) | Sonic Hedgehog Signaling Molecule | GeneCards |  |
| 512 | [SIRT3](https://www.genecards.org/cgi-bin/carddisp.pl?gene=SIRT3&keywords=diabetic,tubulopathy) | Sirtuin 3 | GeneCards |  |
| 513 | [SLC12A1](https://www.genecards.org/cgi-bin/carddisp.pl?gene=SLC12A1&keywords=diabetic,tubulopathy) | Solute Carrier Family 12 Member 1 | GeneCards |  |
| 514 | [SLC12A2](https://www.genecards.org/cgi-bin/carddisp.pl?gene=SLC12A2&keywords=diabetic,tubulopathy) | Solute Carrier Family 12 Member 2 | GeneCards |  |
| 515 | [SLC12A3](https://www.genecards.org/cgi-bin/carddisp.pl?gene=SLC12A3&keywords=diabetic,tubulopathy) | Solute Carrier Family 12 Member 3 | GeneCards |  |
| 516 | [SLC12A4](https://www.genecards.org/cgi-bin/carddisp.pl?gene=SLC12A4&keywords=diabetic,tubulopathy) | Solute Carrier Family 12 Member 4 | GeneCards |  |
| 517 | [SLC12A5](https://www.genecards.org/cgi-bin/carddisp.pl?gene=SLC12A5&keywords=diabetic,tubulopathy) | Solute Carrier Family 12 Member 5 | GeneCards |  |
| 518 | [SLC12A6](https://www.genecards.org/cgi-bin/carddisp.pl?gene=SLC12A6&keywords=diabetic,tubulopathy) | Solute Carrier Family 12 Member 6 | GeneCards |  |
| 519 | [SLC17A1](https://www.genecards.org/cgi-bin/carddisp.pl?gene=SLC17A1&keywords=diabetic,tubulopathy) | Solute Carrier Family 17 Member 1 | GeneCards |  |
| 520 | [SLC17A3](https://www.genecards.org/cgi-bin/carddisp.pl?gene=SLC17A3&keywords=diabetic,tubulopathy) | Solute Carrier Family 17 Member 3 | GeneCards |  |
| 521 | [SLC22A11](https://www.genecards.org/cgi-bin/carddisp.pl?gene=SLC22A11&keywords=diabetic,tubulopathy) | Solute Carrier Family 22 Member 11 | GeneCards |  |
| 522 | [SLC22A5](https://www.genecards.org/cgi-bin/carddisp.pl?gene=SLC22A5&keywords=diabetic,tubulopathy) | Solute Carrier Family 22 Member 5 | GeneCards |  |
| 523 | [SLC22A6](https://www.genecards.org/cgi-bin/carddisp.pl?gene=SLC22A6&keywords=diabetic,tubulopathy) | Solute Carrier Family 22 Member 6 | GeneCards |  |
| 524 | [SLC25A10](https://www.genecards.org/cgi-bin/carddisp.pl?gene=SLC25A10&keywords=diabetic,tubulopathy) | Solute Carrier Family 25 Member 10 | GeneCards |  |
| 525 | [SLC25A20](https://www.genecards.org/cgi-bin/carddisp.pl?gene=SLC25A20&keywords=diabetic,tubulopathy) | Solute Carrier Family 25 Member 20 | GeneCards |  |
| 526 | [SLC25A24](https://www.genecards.org/cgi-bin/carddisp.pl?gene=SLC25A24&keywords=diabetic,tubulopathy) | Solute Carrier Family 25 Member 24 | GeneCards |  |
| 527 | [SLC25A32](https://www.genecards.org/cgi-bin/carddisp.pl?gene=SLC25A32&keywords=diabetic,tubulopathy) | Solute Carrier Family 25 Member 32 | GeneCards |  |
| 528 | [SLC26A3](https://www.genecards.org/cgi-bin/carddisp.pl?gene=SLC26A3&keywords=diabetic,tubulopathy) | Solute Carrier Family 26 Member 3 | GeneCards |  |
| 529 | [SLC26A4](https://www.genecards.org/cgi-bin/carddisp.pl?gene=SLC26A4&keywords=diabetic,tubulopathy) | Solute Carrier Family 26 Member 4 | GeneCards |  |
| 530 | [SLC2A2](https://www.genecards.org/cgi-bin/carddisp.pl?gene=SLC2A2&keywords=diabetic,tubulopathy) | Solute Carrier Family 2 Member 2 | GeneCards | OMIM |
| 531 | [SLC2A5](https://www.genecards.org/cgi-bin/carddisp.pl?gene=SLC2A5&keywords=diabetic,tubulopathy) | Solute Carrier Family 2 Member 5 | GeneCards |  |
| 532 | SLC30A8 | Solute carrier family 30 (zinc transporter), member 8 | OMIM |  |
| 533 | [SLC34A1](https://www.genecards.org/cgi-bin/carddisp.pl?gene=SLC34A1&keywords=diabetic,tubulopathy) | Solute Carrier Family 34 Member 1 | GeneCards |  |
| 534 | [SLC34A3](https://www.genecards.org/cgi-bin/carddisp.pl?gene=SLC34A3&keywords=diabetic,tubulopathy) | Solute Carrier Family 34 Member 3 | GeneCards |  |
| 535 | [SLC35C1](https://www.genecards.org/cgi-bin/carddisp.pl?gene=SLC35C1&keywords=diabetic,tubulopathy) | Solute Carrier Family 35 Member C1 | GeneCards |  |
| 536 | [SLC4A1](https://www.genecards.org/cgi-bin/carddisp.pl?gene=SLC4A1&keywords=diabetic,tubulopathy) | Solute Carrier Family 4 Member 1 (Diego Blood Group) | GeneCards |  |
| 537 | [SLC4A4](https://www.genecards.org/cgi-bin/carddisp.pl?gene=SLC4A4&keywords=diabetic,tubulopathy) | Solute Carrier Family 4 Member 4 | GeneCards |  |
| 538 | [SLC52A1](https://www.genecards.org/cgi-bin/carddisp.pl?gene=SLC52A1&keywords=diabetic,tubulopathy) | Solute Carrier Family 52 Member 1 | GeneCards |  |
| 539 | [SLC52A2](https://www.genecards.org/cgi-bin/carddisp.pl?gene=SLC52A2&keywords=diabetic,tubulopathy) | Solute Carrier Family 52 Member 2 | GeneCards |  |
| 540 | [SLC52A3](https://www.genecards.org/cgi-bin/carddisp.pl?gene=SLC52A3&keywords=diabetic,tubulopathy) | Solute Carrier Family 52 Member 3 | GeneCards |  |
| 541 | [SLC6A3](https://www.genecards.org/cgi-bin/carddisp.pl?gene=SLC6A3&keywords=diabetic,tubulopathy) | Solute Carrier Family 6 Member 3 | GeneCards |  |
| 542 | [SLC8A1](https://www.genecards.org/cgi-bin/carddisp.pl?gene=SLC8A1&keywords=diabetic,tubulopathy) | Solute Carrier Family 8 Member A1 | GeneCards |  |
| 543 | [SLC9A1](https://www.genecards.org/cgi-bin/carddisp.pl?gene=SLC9A1&keywords=diabetic,tubulopathy) | Solute Carrier Family 9 Member A1 | GeneCards |  |
| 544 | [SLC9A2](https://www.genecards.org/cgi-bin/carddisp.pl?gene=SLC9A2&keywords=diabetic,tubulopathy) | Solute Carrier Family 9 Member A2 | GeneCards |  |
| 545 | [SLC9A3](https://www.genecards.org/cgi-bin/carddisp.pl?gene=SLC9A3&keywords=diabetic,tubulopathy) | Solute Carrier Family 9 Member A3 | GeneCards |  |
| 546 | [SLX4](https://www.genecards.org/cgi-bin/carddisp.pl?gene=SLX4&keywords=diabetic,tubulopathy) | SLX4 Structure-Specific Endonuclease Subunit | GeneCards |  |
| 547 | [SMAD3](https://www.genecards.org/cgi-bin/carddisp.pl?gene=SMAD3&keywords=diabetic,tubulopathy) | SMAD Family Member 3 | GeneCards |  |
| 548 | [SMAD7](https://www.genecards.org/cgi-bin/carddisp.pl?gene=SMAD7&keywords=diabetic,tubulopathy) | SMAD Family Member 7 | GeneCards |  |
| 549 | [SNCA](https://www.genecards.org/cgi-bin/carddisp.pl?gene=SNCA&keywords=diabetic,tubulopathy) | Synuclein Alpha | GeneCards |  |
| 550 | [SOD2](https://www.genecards.org/cgi-bin/carddisp.pl?gene=SOD2&keywords=diabetic,tubulopathy) | Superoxide Dismutase 2 | GeneCards | OMIM |
| 551 | [SP1](https://www.genecards.org/cgi-bin/carddisp.pl?gene=SP1&keywords=diabetic,tubulopathy) | Sp1 Transcription Factor | GeneCards |  |
| 552 | [SPG7](https://www.genecards.org/cgi-bin/carddisp.pl?gene=SPG7&keywords=diabetic,tubulopathy) | SPG7 Matrix AAA Peptidase Subunit, Paraplegin | GeneCards |  |
| 553 | [SPINK1](https://www.genecards.org/cgi-bin/carddisp.pl?gene=SPINK1&keywords=diabetic,tubulopathy) | Serine Peptidase Inhibitor Kazal Type 1 | GeneCards | OMIM |
| 554 | [SPTA1](https://www.genecards.org/cgi-bin/carddisp.pl?gene=SPTA1&keywords=diabetic,tubulopathy) | Spectrin Alpha, Erythrocytic 1 | GeneCards |  |
| 555 | [SPTB](https://www.genecards.org/cgi-bin/carddisp.pl?gene=SPTB&keywords=diabetic,tubulopathy) | Spectrin Beta, Erythrocytic | GeneCards |  |
| 556 | [STK24](https://www.genecards.org/cgi-bin/carddisp.pl?gene=STK24&keywords=diabetic,tubulopathy) | Serine/Threonine Kinase 24 | GeneCards |  |
| 557 | [STK33](https://www.genecards.org/cgi-bin/carddisp.pl?gene=STK33&keywords=diabetic,tubulopathy) | Serine/Threonine Kinase 33 | GeneCards |  |
| 558 | [STK39](https://www.genecards.org/cgi-bin/carddisp.pl?gene=STK39&keywords=diabetic,tubulopathy) | Serine/Threonine Kinase 39 | GeneCards |  |
| 559 | [STRA6](https://www.genecards.org/cgi-bin/carddisp.pl?gene=STRA6&keywords=diabetic,tubulopathy) | Signaling Receptor And Transporter Of Retinol STRA6 | GeneCards |  |
| 560 | [STX10](https://www.genecards.org/cgi-bin/carddisp.pl?gene=STX10&keywords=diabetic,tubulopathy) | Syntaxin 10 | GeneCards |  |
| 561 | [SUCLG1](https://www.genecards.org/cgi-bin/carddisp.pl?gene=SUCLG1&keywords=diabetic,tubulopathy) | Succinate-CoA Ligase GDP/ADP-Forming Subunit Alpha | GeneCards |  |
| 562 | SUMO4 | Small ubiquitin-like modifier 4 | OMIM |  |
| 563 | [SURF1](https://www.genecards.org/cgi-bin/carddisp.pl?gene=SURF1&keywords=diabetic,tubulopathy) | SURF1 Cytochrome C Oxidase Assembly Factor | GeneCards |  |
| 564 | [SYMPK](https://www.genecards.org/cgi-bin/carddisp.pl?gene=SYMPK&keywords=diabetic,tubulopathy) | Symplekin | GeneCards |  |
| 565 | [SYT10](https://www.genecards.org/cgi-bin/carddisp.pl?gene=SYT10&keywords=diabetic,tubulopathy) | Synaptotagmin 10 | GeneCards |  |
| 566 | T2D3 | Type 2 diabetes mellitus 3 | OMIM |  |
| 567 | [TACO1](https://www.genecards.org/cgi-bin/carddisp.pl?gene=TACO1&keywords=diabetic,tubulopathy) | Translational Activator Of Cytochrome C Oxidase I | GeneCards |  |
| 568 | [TALDO1](https://www.genecards.org/cgi-bin/carddisp.pl?gene=TALDO1&keywords=diabetic,tubulopathy) | Transaldolase 1 | GeneCards |  |
| 569 | [TANGO2](https://www.genecards.org/cgi-bin/carddisp.pl?gene=TANGO2&keywords=diabetic,tubulopathy) | Transport And Golgi Organization 2 Homolog | GeneCards |  |
| 570 | TBC1D4 | TPC1 domain family, member 4 | OMIM |  |
| 571 | TCF7L2 | Transcription factor 7-like 2 | OMIM |  |
| 572 | [TCIRG1](https://www.genecards.org/cgi-bin/carddisp.pl?gene=TCIRG1&keywords=diabetic,tubulopathy) | T Cell Immune Regulator 1, ATPase H+ Transporting V0 Subunit A3 | GeneCards |  |
| 573 | [TEAD2](https://www.genecards.org/cgi-bin/carddisp.pl?gene=TEAD2&keywords=diabetic,tubulopathy) | TEA Domain Transcription Factor 2 | GeneCards |  |
| 574 | [TF](https://www.genecards.org/cgi-bin/carddisp.pl?gene=TF&keywords=diabetic,tubulopathy) | Transferrin | GeneCards |  |
| 575 | [TFAM](https://www.genecards.org/cgi-bin/carddisp.pl?gene=TFAM&keywords=diabetic,tubulopathy) | Transcription Factor A, Mitochondrial | GeneCards |  |
| 576 | [TFB1M](https://www.genecards.org/cgi-bin/carddisp.pl?gene=TFB1M&keywords=diabetic,tubulopathy) | Transcription Factor B1, Mitochondrial | GeneCards |  |
| 577 | [TFR2](https://www.genecards.org/cgi-bin/carddisp.pl?gene=TFR2&keywords=diabetic,tubulopathy) | Transferrin Receptor 2 | GeneCards |  |
| 578 | [TFRC](https://www.genecards.org/cgi-bin/carddisp.pl?gene=TFRC&keywords=diabetic,tubulopathy) | Transferrin Receptor | GeneCards |  |
| 579 | [TGFB1](https://www.genecards.org/cgi-bin/carddisp.pl?gene=TGFB1&keywords=diabetic,tubulopathy) | Transforming Growth Factor Beta 1 | GeneCards |  |
| 580 | [TIMMDC1](https://www.genecards.org/cgi-bin/carddisp.pl?gene=TIMMDC1&keywords=diabetic,tubulopathy) | Translocase Of Inner Mitochondrial Membrane Domain Containing 1 | GeneCards |  |
| 581 | [TMCO6](https://www.genecards.org/cgi-bin/carddisp.pl?gene=TMCO6&keywords=diabetic,tubulopathy) | Transmembrane And Coiled-Coil Domains 6 | GeneCards |  |
| 582 | [TMEM126B](https://www.genecards.org/cgi-bin/carddisp.pl?gene=TMEM126B&keywords=diabetic,tubulopathy) | Transmembrane Protein 126B | GeneCards |  |
| 583 | [TMEM70](https://www.genecards.org/cgi-bin/carddisp.pl?gene=TMEM70&keywords=diabetic,tubulopathy) | Transmembrane Protein 70 | GeneCards |  |
| 584 | [TMPRSS6](https://www.genecards.org/cgi-bin/carddisp.pl?gene=TMPRSS6&keywords=diabetic,tubulopathy) | Transmembrane Serine Protease 6 | GeneCards |  |
| 585 | TNDM1 | Transient neonatal diabetes mellitus 1 | OMIM |  |
| 586 | [TNFSF11](https://www.genecards.org/cgi-bin/carddisp.pl?gene=TNFSF11&keywords=diabetic,tubulopathy) | TNF Superfamily Member 11 | GeneCards |  |
| 587 | [TOMM20](https://www.genecards.org/cgi-bin/carddisp.pl?gene=TOMM20&keywords=diabetic,tubulopathy) | Translocase Of Outer Mitochondrial Membrane 20 | GeneCards |  |
| 588 | [TOMM20L](https://www.genecards.org/cgi-bin/carddisp.pl?gene=TOMM20L&keywords=diabetic,tubulopathy) | Translocase Of Outer Mitochondrial Membrane 20 Like | GeneCards |  |
| 589 | [TRIM38](https://www.genecards.org/cgi-bin/carddisp.pl?gene=TRIM38&keywords=diabetic,tubulopathy) | Tripartite Motif Containing 38 | GeneCards |  |
| 590 | [TRIT1](https://www.genecards.org/cgi-bin/carddisp.pl?gene=TRIT1&keywords=diabetic,tubulopathy) | TRNA Isopentenyltransferase 1 | GeneCards |  |
| 591 | [TRL-AAG2-3](https://www.genecards.org/cgi-bin/carddisp.pl?gene=TRL-AAG2-3&keywords=diabetic,tubulopathy) | TRNA-Leu (Anticodon AAG) 2-3 | GeneCards |  |
| 592 | [TRMT5](https://www.genecards.org/cgi-bin/carddisp.pl?gene=TRMT5&keywords=diabetic,tubulopathy) | TRNA Methyltransferase 5 | GeneCards |  |
| 593 | [TRMT61B](https://www.genecards.org/cgi-bin/carddisp.pl?gene=TRMT61B&keywords=diabetic,tubulopathy) | TRNA Methyltransferase 61B | GeneCards |  |
| 594 | [TRMU](https://www.genecards.org/cgi-bin/carddisp.pl?gene=TRMU&keywords=diabetic,tubulopathy) | TRNA Mitochondrial 2-Thiouridylase | GeneCards |  |
| 595 | [TRPM6](https://www.genecards.org/cgi-bin/carddisp.pl?gene=TRPM6&keywords=diabetic,tubulopathy) | Transient Receptor Potential Cation Channel Subfamily M Member 6 | GeneCards |  |
| 596 | [TRPM7](https://www.genecards.org/cgi-bin/carddisp.pl?gene=TRPM7&keywords=diabetic,tubulopathy) | Transient Receptor Potential Cation Channel Subfamily M Member 7 | GeneCards |  |
| 597 | [TRPV5](https://www.genecards.org/cgi-bin/carddisp.pl?gene=TRPV5&keywords=diabetic,tubulopathy) | Transient Receptor Potential Cation Channel Subfamily V Member 5 | GeneCards |  |
| 598 | [TSEN54](https://www.genecards.org/cgi-bin/carddisp.pl?gene=TSEN54&keywords=diabetic,tubulopathy) | TRNA Splicing Endonuclease Subunit 54 | GeneCards |  |
| 599 | [TSFM](https://www.genecards.org/cgi-bin/carddisp.pl?gene=TSFM&keywords=diabetic,tubulopathy) | Ts Translation Elongation Factor, Mitochondrial | GeneCards |  |
| 600 | [TWNK](https://www.genecards.org/cgi-bin/carddisp.pl?gene=TWNK&keywords=diabetic,tubulopathy) | Twinkle MtDNA Helicase | GeneCards |  |
| 601 | [TYMP](https://www.genecards.org/cgi-bin/carddisp.pl?gene=TYMP&keywords=diabetic,tubulopathy) | Thymidine Phosphorylase | GeneCards |  |
| 602 | [UBE2T](https://www.genecards.org/cgi-bin/carddisp.pl?gene=UBE2T&keywords=diabetic,tubulopathy) | Ubiquitin Conjugating Enzyme E2 T | GeneCards |  |
| 603 | [UBL4A](https://www.genecards.org/cgi-bin/carddisp.pl?gene=UBL4A&keywords=diabetic,tubulopathy) | Ubiquitin Like 4A | GeneCards |  |
| 604 | UCP3 | Uncoupling protein-3 | OMIM |  |
| 605 | [UGT1A1](https://www.genecards.org/cgi-bin/carddisp.pl?gene=UGT1A1&keywords=diabetic,tubulopathy) | UDP Glucuronosyltransferase Family 1 Member A1 | GeneCards |  |
| 606 | [UMOD](https://www.genecards.org/cgi-bin/carddisp.pl?gene=UMOD&keywords=diabetic,tubulopathy) | Uromodulin | GeneCards |  |
| 607 | [UNC80](https://www.genecards.org/cgi-bin/carddisp.pl?gene=UNC80&keywords=diabetic,tubulopathy) | Unc-80 Homolog, NALCN Channel Complex Subunit | GeneCards |  |
| 608 | [UQCC2](https://www.genecards.org/cgi-bin/carddisp.pl?gene=UQCC2&keywords=diabetic,tubulopathy) | Ubiquinol-Cytochrome C Reductase Complex Assembly Factor 2 | GeneCards |  |
| 609 | [UQCR10](https://www.genecards.org/cgi-bin/carddisp.pl?gene=UQCR10&keywords=diabetic,tubulopathy) | Ubiquinol-Cytochrome C Reductase, Complex III Subunit X | GeneCards |  |
| 610 | [UQCRB](https://www.genecards.org/cgi-bin/carddisp.pl?gene=UQCRB&keywords=diabetic,tubulopathy) | Ubiquinol-Cytochrome C Reductase Binding Protein | GeneCards |  |
| 611 | [UQCRC2](https://www.genecards.org/cgi-bin/carddisp.pl?gene=UQCRC2&keywords=diabetic,tubulopathy) | Ubiquinol-Cytochrome C Reductase Core Protein 2 | GeneCards |  |
| 612 | [UQCRFS1](https://www.genecards.org/cgi-bin/carddisp.pl?gene=UQCRFS1&keywords=diabetic,tubulopathy) | Ubiquinol-Cytochrome C Reductase, Rieske Iron-Sulfur Polypeptide 1 | GeneCards |  |
| 613 | [UQCRQ](https://www.genecards.org/cgi-bin/carddisp.pl?gene=UQCRQ&keywords=diabetic,tubulopathy) | Ubiquinol-Cytochrome C Reductase Complex III Subunit VII | GeneCards |  |
| 614 | [USH1C](https://www.genecards.org/cgi-bin/carddisp.pl?gene=USH1C&keywords=diabetic,tubulopathy) | USH1 Protein Network Component Harmonin | GeneCards |  |
| 615 | [VDR](https://www.genecards.org/cgi-bin/carddisp.pl?gene=VDR&keywords=diabetic,tubulopathy) | Vitamin D Receptor | GeneCards |  |
| 616 | VEGFA | Vascular endothelial growth factor | OMIM |  |
| 617 | [VLDLR](https://www.genecards.org/cgi-bin/carddisp.pl?gene=VLDLR&keywords=diabetic,tubulopathy) | Very Low Density Lipoprotein Receptor | GeneCards |  |
| 618 | [WARS2](https://www.genecards.org/cgi-bin/carddisp.pl?gene=WARS2&keywords=diabetic,tubulopathy) | Tryptophanyl TRNA Synthetase 2, Mitochondrial | GeneCards |  |
| 619 | [WASHC4](https://www.genecards.org/cgi-bin/carddisp.pl?gene=WASHC4&keywords=diabetic,tubulopathy) | WASH Complex Subunit 4 | GeneCards |  |
| 620 | [WASHC5](https://www.genecards.org/cgi-bin/carddisp.pl?gene=WASHC5&keywords=diabetic,tubulopathy) | WASH Complex Subunit 5 | GeneCards |  |
| 621 | [WDR6](https://www.genecards.org/cgi-bin/carddisp.pl?gene=WDR6&keywords=diabetic,tubulopathy) | WD Repeat Domain 6 | GeneCards |  |
| 622 | WFS1 | Wolframin | OMIM |  |
| 623 | [WNK1](https://www.genecards.org/cgi-bin/carddisp.pl?gene=WNK1&keywords=diabetic,tubulopathy) | WNK Lysine Deficient Protein Kinase 1 | GeneCards |  |
| 624 | [WNK3](https://www.genecards.org/cgi-bin/carddisp.pl?gene=WNK3&keywords=diabetic,tubulopathy) | WNK Lysine Deficient Protein Kinase 3 | GeneCards |  |
| 625 | [WNK4](https://www.genecards.org/cgi-bin/carddisp.pl?gene=WNK4&keywords=diabetic,tubulopathy) | WNK Lysine Deficient Protein Kinase 4 | GeneCards |  |
| 626 | [YARS2](https://www.genecards.org/cgi-bin/carddisp.pl?gene=YARS2&keywords=diabetic,tubulopathy) | Tyrosyl-TRNA Synthetase 2 | GeneCards |  |
| 627 | YIPF5 | Yip1 domain family, member 5 | OMIM |  |
| 628 | [YRDC](https://www.genecards.org/cgi-bin/carddisp.pl?gene=YRDC&keywords=diabetic,tubulopathy) | YrdC N6-Threonylcarbamoyltransferase Domain Containing | GeneCards |  |
| 629 | ZFP57 | Zinc finger protein 57, mouse, homolog of | OMIM |  |
| 630 | [ZFPM2](https://www.genecards.org/cgi-bin/carddisp.pl?gene=ZFPM2&keywords=diabetic,tubulopathy) | Zinc Finger Protein, FOG Family Member 2 | GeneCards |  |
| 631 | [ZFYVE21](https://www.genecards.org/cgi-bin/carddisp.pl?gene=ZFYVE21&keywords=diabetic,tubulopathy) | Zinc Finger FYVE-Type Containing 21 | GeneCards |  |
| 632 | [ZNF483](https://www.genecards.org/cgi-bin/carddisp.pl?gene=ZNF483&keywords=diabetic,tubulopathy) | Zinc Finger Protein 483 | GeneCards |  |
| 633 | [ZNF780B](https://www.genecards.org/cgi-bin/carddisp.pl?gene=ZNF780B&keywords=diabetic,tubulopathy) | Zinc Finger Protein 780B | GeneCards |  |
